# Sexual selection, feather wear, and time constraints on the pre‐basic molt explain the acquisition of the pre‐alternate molt in European passerines

**DOI:** 10.1002/ece3.9260

**Published:** 2022-09-06

**Authors:** José J. Cuervo, Judith Morales, Juan J. Soler, Juan Moreno

**Affiliations:** ^1^ Department of Evolutionary Ecology Museo Nacional de Ciencias Naturales (MNCN‐CSIC) Madrid Spain; ^2^ Department of Functional and Evolutionary Ecology Estación Experimental de Zonas Áridas (EEZA‐CSIC) Almería Spain

**Keywords:** birds, feather wear, pre‐alternate molt, sexual selection, social selection, time constraints

## Abstract

Avian feathers need to be replaced periodically to fulfill their functions, with natural, social, and sexual selection presumably driving the evolution of molting strategies. In temperate birds, a common pattern is to molt feathers immediately after the breeding season, the pre‐basic molt. However, some species undergo another molt in winter‐spring, the pre‐alternate molt. Using a sample of 188 European passerine species, Bayesian phylogenetic mixed models, and correlated evolution analyses, we tested whether the occurrence of the pre‐alternate molt was positively associated with proxies for sexual selection (sexual selection hypothesis) and nonsexual social selection (social selection hypothesis), and with factors related to feather wear (feather wear hypothesis) and time constraints on the pre‐basic molt (time constraints hypothesis). We found that the pre‐alternate molt was more frequent in migratory and less gregarious species inhabiting open/xeric habitats and feeding on the wing, and marginally more frequent in species with strong sexual selection and those showing a winter territorial behavior. Moreover, an increase in migratory behavior and sexual selection intensity preceded the acquisition of the pre‐alternate molt. These results provide support for the feather wear hypothesis, partial support for the sexual selection and time constraints hypotheses, and no support for the social selection hypothesis.

## Graphical abstract

Using a sample of 188 European passerine species, we tested whether the occurrence of the pre‐alternate (winter‐spring) molt was positively associated with proxies for sexual selection and nonsexual social selection, and with factors related to feather wear and time constraints on the pre‐basic (summer‐autumn) molt. We found that the pre‐alternate molt was more frequent in migratory and less gregarious species inhabiting open/xeric habitats and feeding on the wing, and marginally more frequent in species with strong sexual selection and those showing a winter territorial behavior. The occurrence of the pre‐alternate molt appears to be promoted by feather wear, sexual selection, and time constraints on the pre‐basic molt, but not by nonsexual social selection.

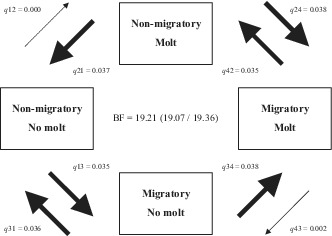

## 1 INTRODUCTION

Sexual and natural selection can interact to shape trait evolution, with the former generally favoring an exaggeration of secondary sexual characters and the latter, a reduction in their expression (Andersson, 1994). However, sexual and natural selection can also have additive effects on phenotypic traits (e.g., Simmons & Scheepers, 1996) that might also be affected by certain types of selection (e.g., nonsexual social selection; Tobias et al., 2012). Disentangling the effects of different types of selection on a trait is a pivotal challenge in evolutionary biology.

A study system particularly well suited to investigate the relative roles of sexual and natural selection, including nonsexual social selection, is the molting pattern of avian feathers. These selection pressures are hypothesized to affect its evolution via a variety of mechanisms (Guallar & Figuerola, 2016; McQueen et al., 2019; Terrill et al., 2020). Feathers are nonliving integumentary structures that cover most of a bird's body and fulfill important functions, such as thermoregulation (Wolf & Walsberg, 2000), flight (Hedenström, 2002), and social signaling (Chaine et al., 2013). Due to degradation by mechanical abrasion and microorganisms (Azcárate‐García et al., 2020), or the need to adjust plumage to seasonal changes (Beltran et al., 2018), feathers need to be replaced regularly through molting to maintain their functionality. Molt is costly because it requires energy and other resources to grow feathers (Lindström, Visser, et al., 1993; Moreno‐Rueda, 2010; Murphy & King, 1992; Sanz et al., 2004) and because plumage function (e.g., flight ability) may be impaired during the molting process (Swaddle & Witter, 1997). A common pattern in birds from temperate regions is to molt immediately after the breeding season, the pre‐basic (summer‐autumn) molt, which results in the basic plumage (Howell et al., 2003; Wolfe et al., 2014). In addition, some species experience a second molt, the pre‐alternate (winter‐spring) molt, which results in alternate plumage (Howell et al., 2003; Wolfe et al., 2014). Basic and alternate plumage may differ in coloration, but most often, they do not (McQueen et al., 2019). A single (pre‐basic) molt in the annual cycle is considered the ancestral state in birds (Hall & Tullberg, 2004; Howell et al., 2003; but see Kiat et al., 2019). However, the evolutionary forces that drive the acquisition of a second (pre‐alternate) molt in some species are not fully understood.

The sexual selection hypothesis (also called variable pressures hypothesis; Terrill et al., 2020), for instance, posits that the pre‐alternate molt evolved in response to differential relative levels of sexual and natural selection throughout the year. Pre‐alternate molt generally occurs just before the start of the breeding season, and some studies have suggested that it evolved to develop a conspicuous plumage important for sexual selection (McQueen et al., 2019). Although conspicuous plumage can be useful during the mating season to attract mates or compete for reproductive resources (Andersson, 1994; Hill, 2006), it is costly because of increased predation risk (Huhta et al., 2003), especially for small‐sized species (Cohen et al., 1993). In this context, the seasonal color change would be the best strategy, with the pre‐alternate molt producing a conspicuous alternate plumage involved in sexual signaling during the breeding season and the pre‐basic molt producing a cryptic basic plumage appropriate for concealment during the nonbreeding season (McQueen et al., 2019; Tökölyi et al., 2008). According to this hypothesis, the presence of a pre‐alternate molt would be more common in sexually dichromatic species because sexual dichromatism is regarded as a proxy for the intensity of sexual selection (Dale et al., 2015; Dunn et al., 2015; Price & Eaton, 2014). Sexual dichromatism, however, might not be a good proxy for overall sexual selection (e.g., Cooney et al., 2018; Mason et al., 2014), but only for sexual selection operating on plumage color. To test whether the evolution of the pre‐alternate molt is driven by overall sexual selection, proxies other than sexual dichromatism (e.g., behavioral ones; Dale et al., 2015) need to be considered.

The feather wear hypothesis states that the pre‐alternate molt evolved to replace worn feathers and keep plumage functional (Terrill et al., 2020). Migration is thought to increase feather wear in birds because of their long flight periods (Schreiber et al., 2006) or prolonged exposure to ultraviolet radiation in low latitude wintering areas (Bergman, 1982; Froehlich et al., 2005; Pageau et al., 2021; Terrill et al., 2020). Under this hypothesis, the presence of the pre‐alternate molt would be positively related to migratory behavior, as has been shown in previous studies (Figuerola & Jovani, 2001; Guallar & Figuerola, 2016; Hall & Tullberg, 2004; Pageau et al., 2021; Svensson & Hedenström, 1999; Terrill et al., 2020). Another factor that might also affect feather wear is habitat type. Two main mechanisms have been proposed to account for this relationship. In the first, open habitats provide fewer opportunities for shade than forested ones, thus birds in these habitats are exposed to higher levels of ultraviolet radiation (Bergman, 1982; Froehlich et al., 2005; Terrill et al., 2020). In the second, xeric (i.e., arid) habitats are considered more abrasive for feathers than mesic (i.e., moist) ones due to either greater exposure to windblown dust or the more abrasive vegetation that is prevalent in dry environments (e.g., grasses) (Rohwer et al., 2005; Willoughby, 1991). According to these mechanisms, we would expect the pre‐alternate molt to be more frequent in species living in open/xeric habitats than in forested/mesic ones. However, feathers can also be degraded by keratinolytic bacteria, which are more active in humid environments (Burtt & Ichida, 2004). Therefore, the opposite effect is also possible.

Nonsexual social selection may have also favored the evolution of the pre‐alternate molt (Guallar & Figuerola, 2016) as the signaling function of the plumage is not restricted to the breeding period. Indeed, many bird species display badges of status, which are important when competing for nonsexual resources such as food or shelter (Chaine et al., 2013; Rohwer, 1975, 1982; Senar, 2006). Under this hypothesis, which we term the social selection hypothesis (although it refers exclusively to nonsexual social selection), the expression of at least some badges of status would be facilitated by the pre‐alternate molt. As gregariousness implies higher social complexity and more information exchange (Pacala et al., 1996), this hypothesis predicts that the pre‐alternate molt would be more frequent in species that are more gregarious in winter (as shown in Motacillidae; Guallar & Figuerola, 2016).

Time constraints on the pre‐basic molt have also been suggested to promote the acquisition of the pre‐alternate molt (Rohwer et al., 2005). When there is a shortage of time to complete the pre‐basic molt, the molt of some feathers might be delayed until winter. This time constraints hypothesis is particularly relevant for migratory species as some studies show that the pre‐basic molt in these species is already under time constraints (de la Hera et al., 2009; Kiat & Sapir, 2017). One of the factors proposed to cause time constraints is aerial foraging, which heavily depends on flight performance. Consequently, avian species feeding on the wing must molt slowly to minimize foraging costs (Rohwer et al., 2005). Slow feather replacement, in turn, may have a strong influence on molting schedules: if aerial foragers do not complete the pre‐basic molt in the breeding grounds, some feathers will have to be molted in the wintering grounds after migration (Rohwer et al., 2005). Winter territoriality may also cause time constraints on the pre‐basic molt. Some species may be under selective pressure to arrive early to the wintering grounds to secure high‐quality territories (Lindström, Pearson, et al., 1993; Sealy & Biermann, 1983). Although this argument has been dismissed for North American passerines wintering in the Neotropics (Rohwer et al., 2005), it might still be valid for other species or geographical areas. Under the time constraints hypothesis, the occurrence of the pre‐alternate molt would be associated with aerial foraging and/or winter territoriality. To complicate the matter, some authors argue that a molt after autumn migration that is driven by time constraints is not a real pre‐alternate molt, but a delayed pre‐basic molt (Pageau et al., 2021). In either case, we would expect this molt to occur early in the winter season. Consequently, if an early winter molt is considered pre‐basic instead of pre‐alternate, we would not expect the pre‐alternate molt to be associated with aerial foraging or winter territoriality.

The pre‐alternate molt varies by species and can be complete, in which all the feathers are replaced, or partial, in which only part of the plumage is replaced. Within European passerine species, for instance, both pre‐alternate molt strategies are relatively common (Jenni & Winkler, 1994). The selective forces driving a partial pre‐alternate molt in some species but a complete one in others are not well understood. However, the hypotheses described above for the acquisition of the pre‐alternate molt might play a role in this context. For example, some studies have suggested that species that migrate long distances have a more extensive pre‐alternate molt than those that migrate shorter distances (Kiat et al., 2019; Terrill et al., 2020). Therefore, we would expect a complete pre‐alternate molt to be more frequent in species with migratory habits, which are more affected by feather wear, than in resident species. The sexual selection might also drive the extent of pre‐alternate molt by favoring a molt episode prior to reproduction to replace cryptic feathers with conspicuous ones (McQueen et al., 2019). If this were the case, there would be no functional reason to change all the feathers but only those that are the target of sexual selection. In this context, we would expect species under strong sexual selection to present a partial pre‐alternate molt more frequently than a complete one.

In this study, we tested four nonmutually exclusive hypotheses to explain the evolution of the pre‐alternate molt: (1) the sexual selection hypothesis, (2) the feather wear hypothesis, (3) the social selection hypothesis, and (4) the time constraints hypothesis. These hypotheses differ in the selective forces involved (sexual selection, nonsexual social selection, and nonsocial natural selection) and the timing of selection within the annual cycle. To test the four hypotheses, we first examined the relationship across species between the presence of the pre‐alternate molt and (1) sexual plumage dichromatism and the intensity of sexual selection, (2) migratory habit and type of habitat, (3) winter gregariousness, and (4) foraging mode and winter territoriality. Second, in only the species with a pre‐alternate molt, we examined the relationship between the extent of the pre‐alternate molt (partial or complete) and the species‐specific traits mentioned above. When a relationship was significant, we also investigated the pattern of correlated evolution between the two characters (Pagel, 1994). In this study, we focused exclusively on factors promoting the acquisition of the pre‐alternate molt and did not address those that may make this molt less probable, such as spring molt constraints (e.g., time and energy limitations) (Froehlich et al., 2005). Moreover, we only considered hypotheses that likely apply to a wide set of species. For example, we did not consider complete biannual molts due to snow‐related seasonality in camouflage requirements (Beltran et al., 2018) as it only applies to three ptarmigan species (genus *Lagopus*; Zimova et al., 2018).

The hypotheses were tested in European breeding species of passerine birds (order Passeriformes), which show a variety of molt strategies (Jenni & Winkler, 1994). Some molt‐related traits, such as seasonal color change by molt, differ between temperate and tropical passerines and among continents (McQueen et al., 2019). By restricting the study to passerines from a single continent (Europe) that is entirely located in one biogeographic realm (Palearctic), the dataset analyzed was more homogeneous in terms of, for example, the climatic conditions experienced by the birds, which, in this case, are predominantly temperate and seasonal.

## 2 MATERIALS AND METHODS

### 2.1 Data collection

Information on the pre‐alternate molt in European breeding passerines was obtained from Busse (1984). A single source of information was used to avoid inter‐observer variability and to have more homogeneous and comparable data. Species were scored as 1 if they have a pre‐alternate (winterspring) molt in the second year of life or 0 if they do not. A pre‐alternate molt in the first year was not considered because it involves a change from immature to adult plumage, which was outside the scope of the present study. In species with a pre‐alternate molt, we also determined whether this molt is complete (scored as 1) or partial (scored as 0), and the feather tracts that are molted. Intraspecific variability was simplified to dichotomous variables according to the most frequent molt pattern shown by European breeding populations. All the species in our dataset show a similar molt pattern in males and females except *Oriolus oriolus*, whose pre‐alternate molt is complete in males but partial in females. The winter season was defined as including November and December, following Busse (1984). However, it could be argued that these are autumn months. Moreover, selective forces driving molt evolution might differ depending on whether molt occurs in early versus late winter (see Section 1). To investigate these issues, we created a second variable for the occurrence of the pre‐alternate molt in which a molt taking place in November or December was considered pre‐basic instead of pre‐alternate.

Information on (log_10_‐transformed) body mass and sexual dichromatism during the breeding season was obtained from McQueen et al. (2019). Body mass was considered in all analyses because body size is related to almost every ecological and life‐history trait of a species (Peters, 1983) and can be an important confounding factor. McQueen et al. (2019) estimated sexual dichromatism from scanned plates of the Handbook of the Birds of the World (del Hoyo et al., 2003–2011). Specifically, sexual dichromatism scores were mean Euclidean distances between red‐green‐blue (RGB) values of homologous plumage patches in males and females of the same species (for further details, see Dale et al., 2015; McQueen et al., 2019). An index of the intensity of sexual selection in each species, obtained from Dale et al. (2015), consisted of the first component of a phylogenetic principal component analysis that included sexual size dimorphism, social mating system, and paternal care (for detailed information, see Dale et al., 2015). Sexual dichromatism was considered as a proxy for sexual selection operating on plumage color, and intensity of sexual selection, as a proxy for overall sexual selection (see Section 1). However, these two parameters were not significantly related in our sample of European passerines (linear regression; *F* = 0.265, df = 1186, *r* ^2^ = .0014, *p* = .607).

Information on the rest of the predictor variables (i.e., winter gregariousness, migratory habit, foraging behavior, and habitat type) was obtained from the Birds of the Western Palearctic (Cramp & Perrins, 1988–1994) and the Handbook of the Birds of the World Alive (del Hoyo et al., 2020). Additional data related to winter gregariousness of some species were gathered from other references (Table S1). Migratory strategies were classified into five categories that reflected migration intensity following Moreno and Soler (2011): resident (scored as 1); resident to short‐distance, resident to partially migratory, or resident to eruptive (scored as 2); altitudinal, short‐distance, partial migrant, or resident to migratory (scored as 3); migratory to short‐distance, or migratory to resident (scored as 4); and migratory (scored as 5). Eruptive movements refer to irregular (not annual) departures from a species' breeding range. Habitat type was scored as one of six classes, from open/xeric to forested/mesic, following the classification used by Moreno and Soler (2011): desert (scored as 1); savannah, steppe, cliff, or high mountain (scored as 2); scrub, tundra, or grassland (scored as 3); riparian area, groves, or wetland (scored as 4); open woodland (scored as 5); and forest (scored as 6). Foraging behavior was classified into three categories according to the importance of aerial foraging, including flycatching and hovering: unimportant (scored as 0), important (scored as 1), and the most important foraging technique (scored as 2). Gregariousness during the nonbreeding season was categorized, following Moreno and Soler (2011), as territorial (individuals defend territories; scored as 1), nongregarious (individuals neither defend territories nor form groups; scored as 2), moderately gregarious (flocks are small or the species is only sometimes gregarious; scored as 3), or gregarious (the species is always gregarious and flocks are large; scored as 4). This categorization of gregariousness also allowed us to study two other strongly related traits: territoriality (in which species are classified as territorial or nonterritorial) and gregariousness without the confounding effect of territoriality (i.e., excluding territorial species).

All 188 species (from 30 families) with detailed information on pre‐alternate molt available in Busse (1984) were included in the study. The complete dataset is shown in Table S2. The specific feather tracts molted in species with a pre‐alternate molt are shown in Table S3.

### 2.2 Statistical analyses

Possible relationships between the occurrence of the pre‐alternate molt and our variables of interest were tested with Bayesian phylogenetic mixed model analyses implemented in the R statistical environment (R Core Team, 2016). We used the MCMCglmm package (Hadfield, 2010) and the libraries “MCMCglmm,” “ape,” “MASS,” and “mvtnorm” (Paradis et al., 2004; Venables & Ripley, 2002). This analysis allows the inclusion of phylogeny as a design matrix that is considered a random effect (Currie & Meade, 2014; Genz & Bretz, 2011). To account for phylogenetic uncertainty, the models were run on 100 random phylogenetic trees based on the Ericson All Species backbone phylogeny downloaded from Birdtree.org (Jetz et al., 2012; Appendix S1A). Tree branch lengths were not transformed as there were no model convergence issues (see Section 3). Residual variance was fixed to one, and the prior distribution of the phylogenetic random effect adjusted to a χ^2^ with one degree of freedom (list[G = list[G1 = list[V = 1, nu = 100, alfa.mu = 0, alfa.V = 1]], R = list[V = 1, fix = 1]]), as recommended for dichotomous dependent variables (de Villemereuil et al., 2013). The MCMC algorithm was run for 2,000,000 iterations, with a burn‐in period of 100,000 and thinning interval of 2000. For each independent variable considered in the model, and for every phylogenetic tree, the analysis provided the estimate and its lower (LCI) and upper (UCI) 95% credibility interval values, effective sample size (ESS), level of autocorrelation, *z*‐score of the Geweke's convergence diagnostic, and pMCMC value. Geweke's convergence diagnostic (Geweke, 1992) with the absolute value of the *z*‐score < 1.96 at the 0.05 alpha level denotes convergence (Zhang, 2016). The pMCMC value is twice the posterior probability that the estimate is positive or negative, using whichever probability is smallest (Hadfield et al., 2013) and can be interpreted in a similar way to a *p* value. The random effect of phylogeny was reported as heritability (*h* ^2^) (Hadfield, 2010), which is a measure of the phylogenetic signal that ranges from zero (nonphylogenetic signal) to 1 (high phylogenetic signal). The average values for all parameters across the 100 models (corresponding to the 100 phylogenetic trees) were then calculated.

When a significant relationship between the occurrence of the pre‐alternate molt and winter gregariousness was found, we performed two additional analyses to elucidate the role of territoriality in this relationship. In the first, territoriality instead of gregariousness was included as a predictor in the model, with species classified as territorial (group 1) or nonterritorial (groups 2, 3, and 4; see Section 2.1 for a definition of categories). In the second, the relationship between the occurrence of pre‐alternate molt and winter gregariousness without the confounding effect of territoriality was tested by removing the 26 territorial species from the sample. For this analysis, gregariousness was reduced to three categories (groups 2, 3, and 4). A new set of 100 phylogenetic trees without the territorial species was downloaded from Birdtree.org (Appendix S1B).

Bayesian phylogenetic mixed model analyses were also used to test for possible relationships between the extent of the pre‐alternate molt (partial or complete) and our variables of interest. Only species with pre‐alternate molt were included in these analyses. Depending on whether a molt in November or December was considered pre‐alternate or pre‐basic, the number of species with a pre‐alternate molt was 83 or 67, respectively (Table S2). Two corresponding sets of 100 random phylogenetic trees were downloaded from Birdtree.org and used for these analyses (Appendix S1C,D). A problem of “separation,” which occurs when one or more independent variables perfectly predict a binary outcome (Zorn, 2005), was detected for the variable migration because all of the nonfully migratory species in our dataset have a partial pre‐alternate molt (see Section 3). We solved the problem by adding a B element to the prior that imposed zero‐mean normality on the predictors (Bolker, 2018; B = list(mu = rep(0, k), V = diag(9, k)); where k is the number of fixed effects in the model, including the intercept). All analyses on the extent of the pre‐alternate molt were performed using the male molt pattern of *Oriolus oriolus*.

When a significant relationship between the presence or the extent of the pre‐alternate molt and a predictor variable was found with the Bayesian phylogenetic mixed model analyses, we investigated the pattern of correlated evolution using Pagel's (1994) discrete analyses. Specifically, we used MCMC modeling and reversible jump, as implemented in BayesTraits (version 3.0) for discrete traits (Pagel & Meade, 2006). As these analyses require dichotomous variables, the predictors were transformed into traits with two states (0 and 1). For the categorical variables, as we had no a priori expectations about the most appropriate cut point to group categories, all possible cut points were used, and the analyses were repeated for every dataset obtained from the different cut points. For the continuous variables (sexual selection and sexual dichromatism), four cut points were chosen (mean, median, upper quartile, and lower quartile), and analyses were repeated for every dataset. Two models in BayesTraits were fitted to the data: the independent model, in which rates of change in one character do not depend on the state of the other character, and the dependent model, in which rates of change in one character depending on the state of the other character. Models were run over a sample of 100 phylogenetic trees with untransformed branch lengths (Appendix S1A,C) using 2,000,000 iterations, a burn‐in period of 100,000, and a thinning interval of 2000. When the autocorrelation coefficient (*r*) of the log‐likelihood in the dependent model was too high (i.e., *r* ≥ .1; sensu Hadfield, 2017, p. 22), the analyses were repeated using a larger thinning interval (to reduce autocorrelation) and a larger number of iterations (to maintain the same number of sampled points) until an acceptable level of autocorrelation was reached (number of iterations, thinning intervals, and autocorrelation coefficients are shown in Figures S1–S6). Marginal likelihoods of the dependent and independent models of evolution were calculated by means of stepping‐stone sampling with 100 stones and 10,000 iterations per stone (Xie et al., 2011). We used a hyper‐exponential prior for all rate parameters, which seeded the mean of the exponential prior from a uniform distribution on the interval 0 to 10. To check for consistency in our estimations and inferences, we ran the dependent and independent models 10 times. We then calculated the Bayes factor (BF), which compares the marginal likelihoods of two models (Kass & Raftery, 1995). The BF was estimated as 2(log[marginal likelihood of dependent model] − log[marginal likelihood of independent model]). Given that we ran the models 10 times, we were able to combine the marginal means and obtain 100 BF estimates. The average and 95% confidence interval (CI) of the BF values associated with each comparison was then calculated. BF values < 2 were interpreted as no evidence of the difference between the tested models, between 2 and 5 as positive evidence, and > 5 as strong evidence (Pagel & Meade, 2006).

When a difference between the two models was detected, we inferred the direction of the evolutionary process by examining the rates of change. Given there are four possibilities of co‐occurrence for two dichotomous characters, the dependent model considers eight transition rate parameters that describe the probability of all possible single changes among the four states (Pagel, 1994). To test predictions on the rate of change from one state to another, we first calculated the marginal likelihoods of two dependent models: one that allows all transition rates to vary and another that restricts two transition rates to be equal. Marginal likelihoods were calculated on 100 phylogenetic trees using BayesTraits (see above). Then, the BF was estimated as 2(log[marginal likelihood of unrestricted model] − log[marginal likelihood of restricted model]). As described above, the models were run 10 times, 100 BF estimates were obtained for each comparison, and the average and 95% CI of the BF values were calculated. Contingent change tests and temporal order tests (Pagel, 1994) were used to test specific directional hypotheses. For more information on these tests, see Figure 3 and Table 2.

## 3 RESULTS

### 3.1 Traits associated with the occurrence of the pre‐alternate molt

**FIGURE 1:**
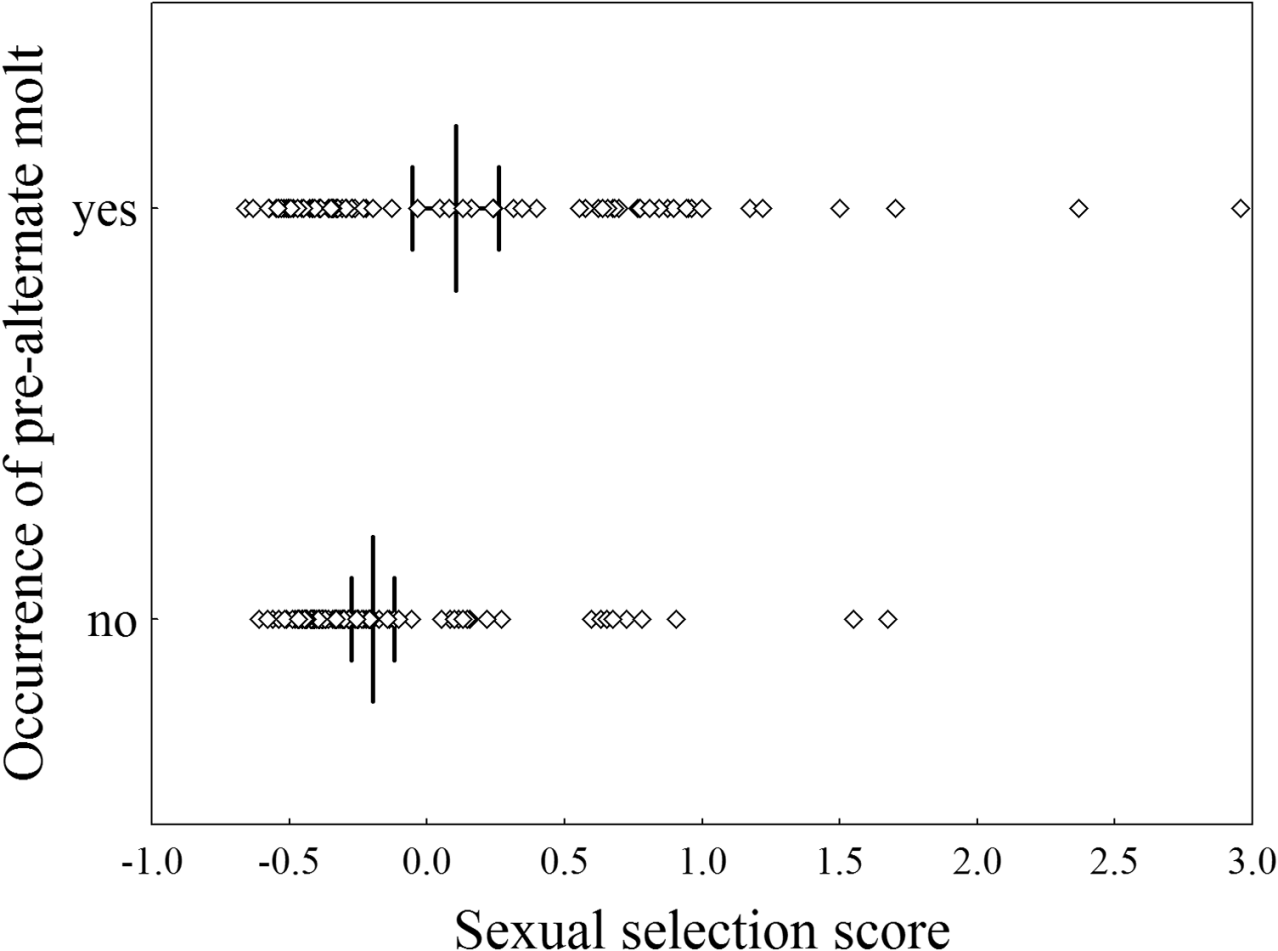
Sexual selection scores for 188 European passerine species with and without pre‐alternate molt. Every dot represents a species. Mean ± 95% CI values are denoted with vertical lines. Feather molt in November or December was considered pre‐alternate.

Eighty‐three out of the 188 species in our sample of European passerines (44%) have a pre‐alternate molt when a molt in November or December was considered as pre‐alternate (Table S2). The intensity of sexual selection was significantly (although marginally) stronger in species with a pre‐alternate molt compared to those without one (Table 1, Figure 1). Correlated evolution between pre‐alternate molt and dichotomous sexual selection was stronger when the mean was chosen as the cut point, with values below and above the mean scored as 0 and 1, respectively (Figure 3a). We found evidence that an increase in the strength of sexual selection precedes the acquisition of the pre‐alternate molt (Table 2, Figure 3a). Sexual dichromatism was not significantly associated with the occurrence of pre‐alternate molts (Table 1). Of the 83 species with a pre‐alternate molt, only 15 (18%) undergo seasonal color change (according to McQueen et al., 2019; Table S3).

**TABLE 1:** MCMCglmm model with the occurrence of the pre‐alternate molt as the dichotomous response variable and sexual selection, sexual dichromatism, migratory behavior, type of habitat, aerial foraging, and winter gregariousness as predictor variables (*n* = 188 species).

Model	Estimate	95% LCI	95% UCI	ESS	Autocorrelation	*z*‐score	pMCMC
(Intercept)	−0.092	−3.927	3.717	951	0.005	0.112	0.903
Body mass	−0.234	−2.249	1.768	964	−0.000	0.028	0.787
Sexual selection	0.958	0.005	1.918	970	0.001	0.089	**0.046**
Sexual dichromatism	0.005	−0.012	0.021	942	0.001	−0.069	0.560
Migration	1.212	0.742	1.689	964	−0.002	−0.130	**0.001**
Type of habitat	−0.519	−0.941	−0.096	943	0.001	0.009	**0.017**
Aerial foraging	1.348	0.287	2.446	994	−0.004	−0.038	**0.014**
Gregariousness	−1.069	−1.694	−0.448	976	−0.002	−0.037	**0.001**
Heritability (*h* ^2^)	0.522	0.454	0.597				

*Note*: Feather molt in November or December was considered pre‐alternate. Gregariousness includes four categories (territorial, nongregarious, moderately gregarious, and gregarious). Log_10_‐transformed body mass was included in the model as a confounding factor. The model was run on 100 random phylogenetic trees, and for each independent variable, we show the average of the following parameters: estimate, lower (LCI) and upper (UCI) 95% credibility interval of the estimate, effective sample size (ESS), level of autocorrelation, *z*‐score of the Geweke's convergence diagnostic, and pMCMC value. Heritability (*h* ^2^) represents the phylogenetic signal, and we show the average of the estimate and the average of 95% LCI and UCI. pMCMC < 0.05 (shown in bold) denotes statistical significance.

**TABLE 2:** Contingent change and temporal order tests for correlated evolution between the occurrence of the pre‐alternate molt and the strength of sexual selection, migratory behavior, aerial foraging, and winter territoriality.

Predictor	Result	Meaning	BF (95% CI)
Sexual selection	*q*12 < *q*34	Acquisition of pre‐alternate molt is more frequent in species with strong sexual selection	**6.93 (6.65/7.20)**
	*q*21 < *q*43	Loss of pre‐alternate molt is more frequent in species with strong sexual selection	**4.37 (4.13/4.61)**
	*q*13 ≈ *q*24	Increase in sexual selection does not depend on pre‐alternate molt	−1.21 (−1.54/−0.88)
	*q*31 ≈ *q*42	Reduction in sexual selection does not depend on pre‐alternate molt	1.74 (1.49/1.99)
	*q*12 < *q*13	Increase in sexual selection precedes acquisition of pre‐alternate molt	**5.07 (4.54/5.60)**
	*q*42 ≈ *q*43	No clear order in reduction in sexual selection and loss of pre‐alternate molt	0.39 (0.09/0.69)
Migration	*q*12 < *q*34	Acquisition of pre‐alternate molt is more frequent in migratory species	**18.22 (18.08/18.37)**
	*q*21 > *q*43	Loss of pre‐alternate molt is more frequent in nonmigratory species	**3.88 (3.76/3.99)**
	*q*13 ≈ *q*24	Acquisition of migration does not depend on pre‐alternate molt	−1.37 (−1.45/−1.29)
	*q*31 ≈ *q*42	Loss of migration does not depend on pre‐alternate molt	−1.84 (−1.92/−1.77)
	*q*12 < *q*13	Acquisition of migration precedes acquisition of pre‐alternate molt	**13.20 (13.04/13.35)**
	*q*42 > *q*43	Loss of migration precedes loss of pre‐alternate molt	**3.54 (3.47/3.61)**
Aerial foraging	*q*12 ≈ *q*34	Acquisition of pre‐alternate molt does not depend on aerial foraging	−1.50 (−1.57/−1.42)
	*q*21 ≈ *q*43	Loss of pre‐alternate molt does not depend on aerial foraging	−1.86 (−1.94/−1.78)
	*q*13 < *q*24	Acquisition of aerial foraging is more frequent in species with pre‐alternate molt	**8.00 (7.91/8.09)**
	*q*31 > *q*42	Loss of aerial foraging is more frequent in species without pre‐alternate molt	**2.94 (2.82/3.06)**
	*q*12 > *q*13	Acquisition of pre‐alternate molt precedes acquisition of aerial foraging	**8.05 (7.96/8.14)**
	*q*42 < *q*43	Loss of pre‐alternate molt precedes loss of aerial foraging	**3.59 (3.51/3.66)**
Territoriality	*q*12 ≈ *q*34	Acquisition of pre‐alternate molt does not depend on territoriality	−0.54 (−0.62/−0.46)
	*q*21 ≈ *q*43	Loss of pre‐alternate molt does not depend on territoriality	−1.76 (−1.85/−1.68)
	*q*13 ≈ *q*24	Loss of territoriality does not depend on pre‐alternate molt	0.96 (0.84/1.09)
	*q*31 < *q*42	Acquisition of territoriality is more frequent in species with pre‐alternate molt	**10.34 (10.24/10.44)**
	*q*31 < *q*34	Acquisition of pre‐alternate molt precedes acquisition of territoriality	**10.71 (10.61/10.81)**
	*q*21 ≈ *q*24	No clear order in loss of pre‐alternate molt and territoriality	−0.95 (−1.05/−0.84)

*Note*: Feather molt in November or December was considered pre‐alternate. Contingent change tests explore whether acquisition or loss of the first variable depends on the state of the second variable (*q*12 ≠ *q*34 or *q*21 ≠ *q*43, respectively) and whether acquisition or loss of the second variable depends on the state of the first variable (*q*13 ≠ *q*24 or *q*31 ≠ *q*42, respectively). Temporal order tests explore whether acquisition of the first variable precedes or follows acquisition of the second variable (*q*12 ≠ *q*13) and whether loss of the first variable precedes or follows loss of the second variable (*q*42 ≠ *q*43). In the case of winter territoriality and the occurrence of the pre‐alternate molt, state 0 in one character is associated with state 1 in the other, so temporal order tests change accordingly (*q*31 ≠ *q*34 and *q*21 ≠ *q*24). For graphic representation of transitions, see Figure 3. Bayes factors (BF) ≥ 2 (in bold) show evidence that transition rates differ.

**FIGURE 2:**
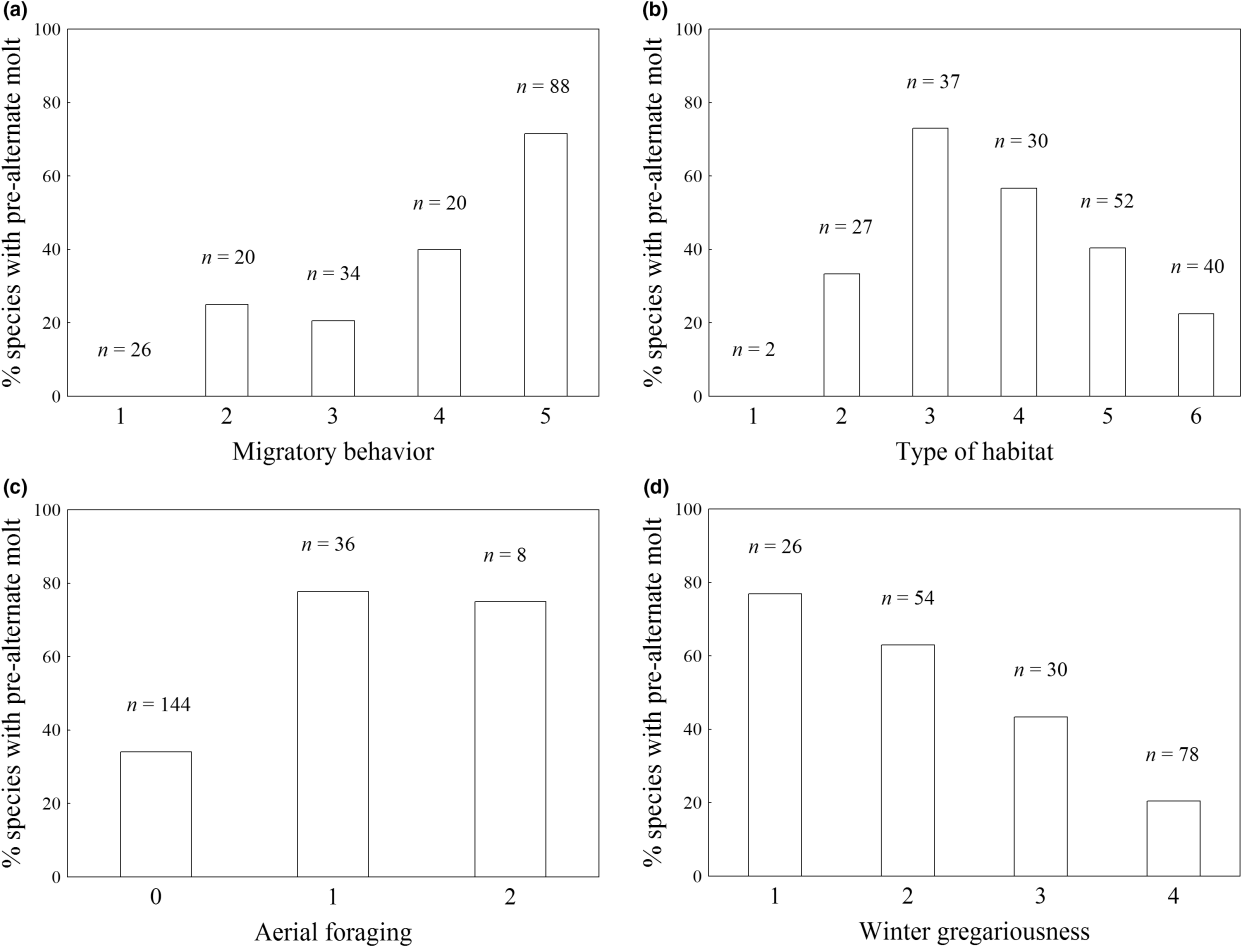
Percentage of European passerine species with pre‐alternate molt in relation to (a) migratory behavior (1 = resident; 2 = resident to short‐distance, resident to partially migratory, or resident to eruptive; 3 = altitudinal, short‐distance, partial migrant, or resident to migratory; 4 = migratory to short‐distance, or migratory to resident; 5 = migratory), (b) type of habitat (1 = desert; 2 = savannah, steppe, cliffs, or high mountain; 3 = scrub, tundra, or grassland; 4 = riparian area, groves, or wetland; 5 = open woodland; 6 = forest), (c) aerial foraging (0 = unimportant; 1 = important; 2 = the most important foraging technique), and (d) winter gregariousness (1 = territorial; 2 = nongregarious; 3 = moderately gregarious; 4 = gregarious). Feather molt in November or December was considered pre‐alternate.

**FIGURE 3:**
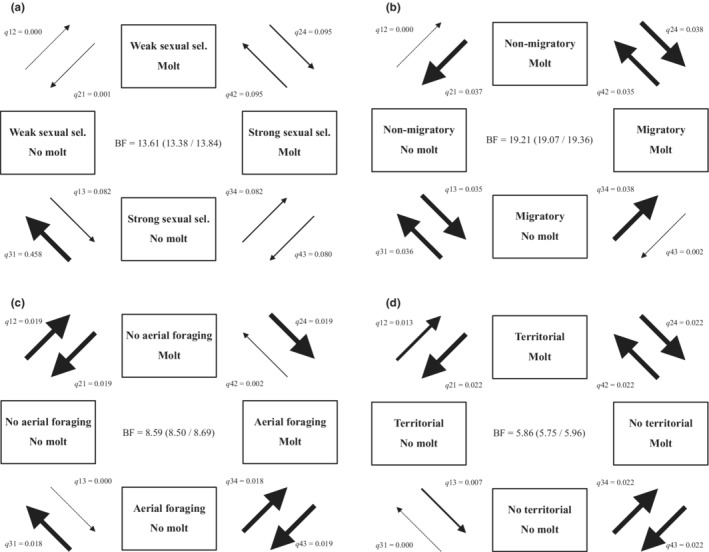
Flow diagrams showing correlated evolution between the occurrence of the pre‐alternate molt and (a) the strength of sexual selection, (b) migratory behavior, (c) aerial foraging, and (d) winter territoriality in European passerine species. Feather molt in November or December was considered pre‐alternate. For information on dichotomization of variables, see main text. Bayes factors (BF (95% CI)) indicate the probability of correlated evolution between the two traits. Transitions of one dichotomous character holding the state of the other constant are represented by arrows and named as *q*(*ij*). The associated mean rate parameter for each transition is shown. Arrow width is proportional to the magnitude of the mean rate parameter within each diagram. For two dichotomous characters, there are four possibilities of co‐occurrence: 00 (1), 01 (2), 10 (3), and 11 (4). The number in parentheses is the identity of each combination of characters (1, 2, 3, or 4), the first number within each pair (0 or 1) represents the state of one character, and the second number the state of the other character. The dependent model considers eight transition rate parameters that describe the probability of all possible single changes among the four states: *q*12, *q*21, *q*13, *q*31, *q*24, *q*42, *q*34, and *q*43. For example, *q*12 refers to the transition from 00 to 01 and *q*43 to the transition from 11 to 10. Posterior distribution and percentage of visits assigned as zero (*Z*‐value) for every rate parameter are shown in Figures S1–S4.

Migratory behavior also explained interspecific variation in the presence of the pre‐alternate molt (Table 1). Specifically, species with more migratory behaviors showed the pre‐alternate molt more frequently than less migratory species (Figure 2a). Correlated evolution between pre‐alternate molt and dichotomous migratory habits was stronger when groups 1, 2, and 3 were classified as nonmigratory and groups 4 and 5, as migratory (Figure 3b). Contingent change tests showed that acquisition of the pre‐alternate molt occurs most frequently in the migratory species (Table 2, Figure 3b).

Pre‐alternate molt was also associated with habitat type (Table 1) This molt was more frequent in species living in open/xeric habitats than those in forested/mesic ones (Figure 2b). However, only a relatively small proportion of the species living in the most xeric habitats (groups 1 and 2) have a pre‐alternate molt (Figure 2b). The association between pre‐alternate molt and dichotomous habitat type was strongest when forest species (group 6) were compared with nonforest ones (groups 1–5). However, the results of this analysis did not reach the threshold level to suggest correlated evolution (BF (95% CI) = 1.55 (1.47/1.63)).

Pre‐alternate molt was also associated with aerial foraging (Table 1, Figure 2c). Moreover, correlated evolution between the occurrence of the pre‐alternate molt and dichotomous foraging behavior was strongest when species in which aerial foraging is important or the most important foraging technique (groups 1 and 2) were pooled together (Figure 3c). We found that acquisition of aerial foraging occurs more frequently in species with a pre‐alternate molt (Table 2, Figure 3c), but that acquisition of this molt does not appear to depend on aerial foraging (Table 2, Figure 3c).

Another trait explaining interspecific variation in pre‐alternate molt was winter gregariousness (Table 1). Contrary to expectations, the pre‐alternate molt was less frequent in the more gregarious species (Figure 2d). When territorial species were excluded from the analysis, the association between pre‐alternate molt and gregariousness remained qualitatively identical (estimate = −1.161, pMCMC = 0.006; Model 1 in Table S4). When gregariousness was replaced with territoriality in the model, a (marginally) significant association with pre‐alternate molt was observed (estimate = −1.681, pMCMC = 0.042; Model 2 in Table S4), with the pre‐alternate molt being more frequent in territorial than nonterritorial species (Figure 2d). With respect to gregariousness as a dichotomous variable, only classifying species as territorial or nonterritorial suggested correlated evolution with pre‐alternate molt (Figure 3d). We found that acquisition of territoriality occurs more frequently in species with a pre‐alternate molt, but that acquisition of this molt does not appear to depend on territoriality (Table 2, Figure 3d).

When a molt in November or December was considered pre‐basic instead of pre‐alternate, 67 of the 188 European passerines in our sample (36%) were classified as having a pre‐alternate molt (Table S2). Under this classification, the relationship between the occurrence of the pre‐alternate molt and the different variables of interest remained largely the same (Table 3), except in two cases. First, aerial foraging was no longer significantly associated with pre‐alternate molt (Table 3). Second, although the occurrence of the pre‐alternate molt was still associated with winter gregariousness when territorial species were excluded from the analysis (estimate = −1.375, pMCMC = 0.001; Model 3 in Table S4), the relationship with territoriality was no longer significant (estimate = −0.536, pMCMC = 0.437; Model 4 in Table S4).

**TABLE 3:** MCMCglmm model with the occurrence of the pre‐alternate molt as the dichotomous response variable and sexual selection, sexual dichromatism, migratory behavior, type of habitat, aerial foraging, and winter gregariousness as predictor variables (*n* = 188 species).

Model	Estimate	95% LCI	95% UCI	ESS	Autocorrelation	*z*‐Score	pMCMC
(Intercept)	1.498	−2.184	5.294	980	−0.004	−0.051	0.437
Body mass	−1.097	−3.063	0.872	959	−0.002	0.049	0.281
Sexual selection	0.840	0.036	1.654	964	−0.005	−0.056	**0.040**
Sexual dichromatism	0.009	−0.007	0.023	943	−0.002	0.027	0.271
Migration	0.728	0.299	1.162	966	−0.004	0.145	**0.001**
Type of habitat	−0.459	−0.856	−0.063	966	−0.005	−0.017	**0.024**
Aerial foraging	0.569	−0.375	1.507	970	−0.007	0.035	0.246
Gregariousness	−0.914	−1.475	−0.355	974	−0.001	−0.036	**0.002**
Heritability (*h* ^2^)	0.520	0.452	0.594				

*Note*: Feather molt in November or December was considered pre‐basic. Gregariousness includes four categories (territorial, nongregarious, moderately gregarious, and gregarious). Log_10_‐transformed body mass was included in all models as a confounding factor. pMCMC < 0.05 (shown in bold) denotes statistical significance. Remaining information as in Table 1.

### 3.2 Traits associated with the extent of the pre‐alternate molt

Of the 83 passerine species with a pre‐alternate molt in our dataset when a molt in November or December was considered pre‐alternate, 48 (58%) have a partial molt and 35 (42%) have a complete one (Table S2). Although several of the species with a partial pre‐alternate molt change some flight feathers (i.e., primaries, secondaries, tertials, or rectrices), most (37 species, 77%) change only nonflight feathers (i.e., wing covers, head feathers, or body feathers; Table S3).

**FIGURE 4:**
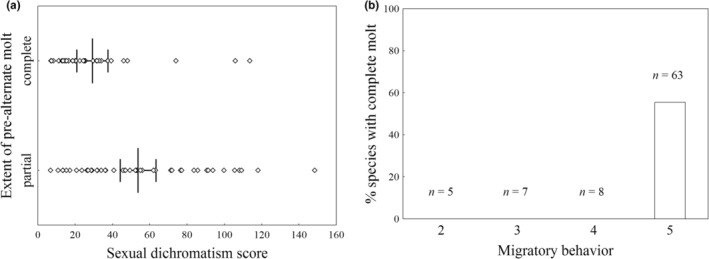
(a) Sexual dichromatism scores for 83 European passerine species with partial or complete pre‐alternate molt. Every dot represents a species. Mean ± 95% CI values are denoted with vertical lines. (b) Percentage of European passerine species with complete (as opposed to partial) pre‐alternate molt in relation to migratory behavior (2 = resident to short‐distance, resident to partially migratory, or resident to eruptive; 3 = altitudinal, short‐distance, partial migrant, or resident to migratory; 4 = migratory to short‐distance, or migratory to resident; 5 = migratory). In both figures, feather molt in November or December was considered pre‐alternate.

**FIGURE 5:**
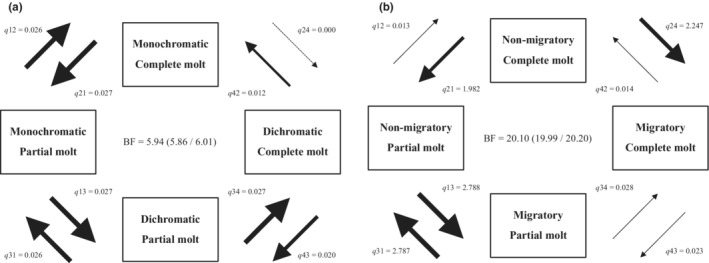
Flow diagrams showing correlated evolution between the extent of the pre‐alternate molt (partial/complete) and (a) sexual plumage dichromatism or (b) migratory behavior in European passerine species. Feather molt in November or December was considered pre‐alternate. For information on dichotomization of variables, see main text. Posterior distribution and percentage of visits assigned as zero (*Z*‐value) for every rate parameter are shown in Figures S5 and S6 and remaining information as in Figure 3.

We found that the extent of the pre‐alternate molt was associated with sexual dichromatism (Table 4). Specifically, species with a partial pre‐alternate molt were more dichromatic than those with a complete one (Figure 4a). Correlated evolution between the extent of this molt and dichotomous sexual dichromatism was strongest when the upper quartile was chosen as the cut point, with values below and above this quartile scored as 0 (monochromatic) and 1 (dichromatic), respectively (Figure 5a). We found strong evidence suggesting that the transition from a complete to partial pre‐alternate molt precedes the change from mono‐ to dichromatism (Table 5, Figure 5a).

**TABLE 4:** MCMCglmm model with the extent of the pre‐alternate molt (partial or complete) as the dichotomous response variable and sexual selection, sexual dichromatism, migratory behavior, type of habitat, aerial foraging, and winter gregariousness as predictor variables.

	Estimate	95% LCI	95% UCI	ESS	Autocorrelation	*z*‐Score	pMCMC
(Intercept)	−5.071	−9.680	−0.493	968	0.001	0.070	**0.029**
Body mass	−0.553	−3.241	2.015	975	0.004	−0.132	0.691
Sexual selection	0.145	−0.819	1.117	966	−0.002	−0.070	0.776
Sexual dichromatism	−0.038	−0.068	−0.010	958	0.003	0.084	**0.006**
Migration	1.702	0.692	2.758	957	0.000	−0.095	**0.001**
Type of habitat	−0.180	−0.809	0.442	964	−0.004	0.063	0.580
Aerial foraging	0.057	−1.112	1.235	951	0.001	−0.013	0.926
Gregariousness	−0.174	−0.928	0.577	962	0.002	0.279	0.655
Heritability (*h* ^2^)	0.510	0.444	0.583				

*Note*: Only species with pre‐alternate molt are included in the analysis (*n* = 83 species). Feather molt in November or December was considered pre‐alternate. Gregariousness includes four categories (territorial, nongregarious, moderately gregarious, and gregarious). Log_10_‐transformed body mass was included in the model as a confounding factor. pMCMC < 0.05 (shown in bold) denotes statistical significance. Remaining information as in Table 1.

**TABLE 5:** Contingent change and temporal order tests for correlated evolution between the extent of the pre‐alternate molt (partial/complete) and sexual plumage dichromatism or migratory behavior.

Predictor	Result	Meaning	BF (95% CI)
Sex. dichromatism	*q*12 ≈ *q*34	Transition from partial to complete pre‐alternate molt does not depend on dichromatism	−1.70 (−1.76/−1.64)
	*q*21 ≈ *q*43	Transition from complete to partial pre‐alternate molt does not depend on dichromatism	−1.23 (−1.32/−1.15)
	*q*13 > *q*24	Transition from mono‐ to dichromatism is more frequent in species with partial pre‐alternate molt	**8.73 (8.66/8.79)**
	*q*31 ≈ *q*42	Transition from di‐ to monochromatism does not depend on the extent of pre‐alternate molt	−0.06 (−0.14/0.03)
	*q*31 ≈ *q*34	No clear order in transitions from partial to complete pre‐alternate molt and from di‐ to monochromatism	−1.09 (−1.16/−1.01)
	*q*21 > *q*24	Transition from complete to partial pre‐alternate molt precedes transition from mono‐ to dichromatism	**9.12 (9.04/9.21)**
Migration	*q*12 ≈ *q*34	Transition from partial to complete pre‐alternate molt does not depend on migration	−0.26 (−0.42/−0.09)
	*q*21 ≈ *q*43	Transition from complete to partial pre‐alternate molt does not depend on migration	−0.25 (−0.36/−0.14)
	*q*13 ≈ *q*24	Acquisition of migration does not depend on the extent of pre‐alternate molt	−1.68 (−1.80/−1.55)
	*q*31 > *q*42	Loss of migration is more frequent in species with partial pre‐alternate molt	**4.68 (4.54/4.82)**
	*q*12 < *q*13	Acquisition of migration precedes transition from partial to complete pre‐alternate molt	**4.36 (3.94/4.78)**
	*q*42 ≈ *q*43	No clear order in loss of migration and transition from complete to partial pre‐alternate molt	0.50 (0.25/0.75)

*Note*: Feather molt in November or December was considered pre‐alternate. Meaning of contingent change and temporal order tests as in Table 2. In the case of sexual dichromatism and the extent of the pre‐alternate molt, state 0 in one character is associated with state 1 in the other, so temporal order tests change accordingly (*q*31 ≠ *q*34 and *q*21 ≠ *q*24). For graphic representation of transitions, see Figure 5. Bayes factors (BF) ≥ 2 (in bold) show evidence that transition rates differ. In the case of migratory behavior and the extent of the pre‐alternate molt, rate parameters *q*13, *q*21, *q*24, and *q*31 showed widely scattered values (Figure S6), suggesting that their means do not accurately represent the samples. Therefore, and despite the marked difference in proportion of zeros between *q*31 (0.0%) and *q*42 (61.1%), or between *q*12 (61.2%) and *q*13 (1.4%), the differences found between these rate parameters should be considered with caution.

The only other trait associated with the extent of the pre‐alternate molt was migration (Table 4), with about half of the fully migratory species (group 5) having a complete pre‐alternate molt and none of the species in the other migratory categories showing such molt pattern (Figure 4b). Correlated evolution between the extent of the pre‐alternate molt and dichotomous migratory habits was strongest when groups 2, 3, and 4 were classified as nonmigratory and group 5, as migratory (Figure 5b). The results of the temporal order tests showed that acquisition of migratory behavior precedes the transition from a partial to complete pre‐alternate molt (Table 5, Figure 5b). However, this finding should be considered with caution due to the widely scattered values of some of the rate parameters (see footnotes of Table 5).

Of the 67 species classified as having a pre‐alternate molt when a molt in November or December was considered pre‐basic, 47 (70%) have a partial molt and 20 (30%) have a complete one (Table S2). As in the previous case (in which a molt in November or December was considered pre‐alternate), only migratory behavior and sexual dichromatism were significantly associated with the extent of the pre‐alternate molt (Model 5 in Table S4).

## 4 DISCUSSION

### 4.1 Sexual selection hypothesis

In accordance with the sexual selection hypothesis, we found an association between the intensity of sexual selection and the occurrence of the pre‐alternate molt in European passerine species. Moreover, an increase in the intensity of sexual selection precedes the acquisition of the pre‐alternate molt, a result that is consistent with a causal relationship in the direction suggested by the hypothesis. These results are in line with previous studies showing that pre‐alternate molts leading to seasonal color change are more common in species with polygynous mating systems (McQueen et al., 2019; Tökölyi et al., 2008). However, they must be interpreted with caution because they rely heavily on a marginally significant relationship (see Table 1). Therefore, we conclude that our results provide only partial support for the sexual selection hypothesis. With respect to sexual dichromatism, we found that this variable was not significantly related to the occurrence of the pre‐alternate molt, a result that confirms those obtained in previous studies (Figuerola & Jovani, 2001; Guallar & Figuerola, 2016). Therefore, we have a scenario in which a proxy for overall sexual selection is related to the occurrence of pre‐alternate molt, but one for sexual selection operating on plumage color is not. This could call into question the argument that acquiring ornamental coloration is the main reason for sexual selection to promote feather replacement. We can speculate that plumage plays an important role in sexual selection beyond its color, for example in aerial sexual displays (Menezes & Santos, 2020).

With respect to the relationship between sexual dichromatism and the occurrence of the pre‐alternate molt, at least three factors might have weakened this association. First, seasonal color change by pre‐alternate molts has been shown to be more common in sexually dichromatic species (McQueen et al., 2019). However, in our sample, only a very small number of species with pre‐alternate molts undergo seasonal color change (15 out of 83 species). Second, in some passerine species, sexual selection can favor seasonal color change by feather abrasion instead of pre‐alternate molts (Tökölyi et al., 2008). And third, loss of female ornamentation instead of the acquisition of male ornamentation (i.e., natural instead of sexual selection) seems to be the cause of sexual dichromatism in some passerines (Hofmann et al., 2008; Simpson et al., 2015; Soler & Moreno, 2012).

Our results also suggest that partial molts are predominant in sexually dichromatic species with a pre‐alternate molt. The association between sexual dichromatism and a partial pre‐alternate molt seems to stem from the fact that (i) these molts generally only replace nonflight feathers (Jenni & Winkler, 1994; Table S3) and (ii) coloration of nonflight feathers is particularly important in visual sexual signaling (Dale et al., 2015; McQueen et al., 2019). According to this explanation, sexual selection favors the evolution of partial pre‐alternate molts through the replacement of nonflight feathers that gives rise to seasonal, sexually dichromatic color patches. However, we found that transitions from a complete to partial pre‐alternate molt precede transitions from mono‐ to dichromatism, suggesting an alternative evolutionary pathway. Perhaps, when flight feathers stop molting during the pre‐alternate molt in sexually monochromatic species (e.g., because of changes in feather wear), nonflight feathers continue being replaced and eventually develop a sexually dichromatic ornamental breeding plumage.

### 4.2 Feather wear hypothesis

As predicted by the feather wear hypothesis, we found that the pre‐alternate molt was more frequent in migratory species. An association between the occurrence or extent of the pre‐alternate molt and migratory habits has already been proposed for several passerine families such as Sylviidae (Hall & Tullberg, 2004; Svensson & Hedenström, 1999), Motacillidae (Guallar & Figuerola, 2016), and Parulidae (Terrill et al., 2020). Moreover, studies considering Western Palearctic passerines from different families have shown that the frequency of the pre‐alternate molt increases with migration distance (Figuerola & Jovani, 2001; Pageau et al., 2021). The mechanism proposed to explain this association is based on the understanding that migration favors the pre‐alternate molt. Our study supports this scenario because these two traits evolve correlatively, with the acquisition of the pre‐alternate molt being more frequent in migratory species. This order of evolutionary events (migratory habits evolve first, followed by pre‐alternate molt) has been suggested for the family Parulidae (Terrill et al., 2020). Our study now broadens the number of species and passerine families that seem to follow this order.

In the case of migratory species that breed in the Nearctic and Western Palearctic, photoperiod in the wintering grounds has been shown to be positively related to the occurrence of the pre‐alternate molt (Pageau et al., 2021). For European breeding passerines, interspecific variation in migratory behavior should be intrinsically associated with variation in winter photoperiod, given the predominantly north–south direction of migration (Busse, 2001) and the pronounced change in photoperiod with latitude during winter in the Northern Hemisphere. If this were the case, the association between migration and pre‐alternate molt would be consistent with that between winter photoperiod and this molt.

Although it should be considered with caution, we found that a change from a moderate to fully migratory behavior precedes the transition from a partial to complete pre‐alternate molt. This might not only be due to fully migratory habits imposing more intense feather wear but also a stronger selection on flight performance, possibly with an emphasis on spring migration (Kokko, 1999; Nilsson et al., 2013). In either case, the replacement of abraded flight feathers with newly grown ones would improve flight performance (Bridge, 2008). Therefore, if a species with a partial pre‐alternate molt, which usually consists of the replacement of nonflight feathers, became migratory, it could begin to also molt worn flight feathers during winter to improve flight performance. As a result, this species would develop a complete pre‐alternate molt.

Also in agreement with the feather wear hypothesis, the pre‐alternate molt was more frequent in species living in open/xeric habitats. Apart from deserts, where only two species included in the study live, forests showed the lowest percentage of species with a pre‐alternate molt. This is consistent with the idea that shade provided by trees decreases exposure to ultraviolet radiation, thus reducing feather damage (Bergman, 1982) and making additional molts less necessary (Froehlich et al., 2005; Terrill et al., 2020). Contrary to a recent study reporting water habitats as having the highest percentage of species with a pre‐alternate molt (Pageau et al., 2021), in our study, it was the habitat category including grasslands. Our finding is consistent with a scenario in which abrasive vegetation, such as grasses, increases feather wear, ultimately promoting additional molting (Rohwer et al., 2005; Willoughby, 1991). Aridity itself, however, might play a minor role, as the category including steppes and savannahs, although drier than grassland or scrub, contained a lower proportion of species with a pre‐alternate molt. Alternatively, the role of aridity on feather wear might be obscured by the activity of feather‐degrading bacteria that thrive in mesic habitats (Burtt & Ichida, 2004). In any case, all these factors are related to one another, and further studies are needed to disentangle their specific effects on feather wear and replacement. Overall, our results showing the association of migratory behavior and habitat type with pre‐alternate molt support the feather wear hypothesis.

### 4.3 Social selection hypothesis

Contrary to the prediction of the social selection hypothesis, the results of this study suggest that passerine species with a pre‐alternate molt are less gregarious during winter. When territorial species were excluded from the analyses, the results remained qualitatively identical, implying that territoriality was not responsible for this association. Therefore, our results do not support the social selection hypothesis. To our knowledge, this is the first study to report a negative relationship between the occurrence of the pre‐alternate molt and winter gregariousness. Guallar and Figuerola (2016) found evidence of correlated evolution between the two traits in Motacillidae, but they also observed a nonsignificant relationship between them in their phylogenetic generalized least squares analyses. Such evidence of correlated evolution should thus be interpreted with caution (Maddison & FitzJohn, 2015).

A possible explanation for the negative association between winter gregariousness and pre‐alternate molt is that plumage signals used in sexual contexts during mating are already present during the nonbreeding period in gregarious species as badges of status (e.g., Mennill et al., 2003). In fact, plumage signaling has been shown to play a similarly important role during breeding and nonbreeding periods (Santos et al., 2011). One reason to replace feathers before the breeding season might be to develop sexual plumage signals (McQueen et al., 2019; Tökölyi et al., 2008), but such molt would not be necessary if those signals were already present. Badges of status would be particularly important, thus more frequent, in gregarious than nongregarious species because of their higher social complexity (Tibbetts & Safran, 2009). Moreover, the evolution of badges of status in birds is associated with winter (but not summer) gregariousness (Tibbetts & Safran, 2009). Assuming winter gregariousness as a proxy for the intensity of nonsexual social selection, our results suggest that the pre‐alternate molt is less likely to evolve when strong social selection is operating during the nonbreeding season.

Some caveats, however, should be noted regarding the relationship between winter gregariousness and the pre‐alternate molt. First, for many species, particularly those wintering in sub‐Saharan Africa, little is known about nonbreeding gregariousness, so this may limit data quality. Second, some flocking bird species appear not to compete directly over food (Yates et al., 2000), so gregariousness might not be a good proxy for social selection, although other forms of competition are also possible (e.g., for particular positions within roosts; McGowan et al., 2006). And third, winter gregariousness is associated with other factors that might also affect molt evolution, such as foraging efficiency (Beauchamp, 1998) and predation risk (Whitfield, 2003). All these potential limitations make us cautious in interpreting the results. Despite the negative association between winter gregariousness and the presence of the pre‐alternate molt, we limit ourselves to concluding that our results do not suggest a positive association between the two traits and thus do not support the social selection hypothesis.

### 4.4 Time constraints hypothesis

As predicted by the time constraints hypothesis, we found that the pre‐alternate molt is more frequent in species that feed on the wing and are territorial in winter. Moreover, if time constraints were the only reason for a pre‐alternate molt to occur, it should happen in early winter instead of late winter or spring. In agreement with this prediction, when a molt in early winter (November to December) was considered pre‐basic instead of pre‐alternate, both foraging type and winter territoriality were no longer significantly associated with the occurrence of the pre‐alternate molt. This implies that when aerial foragers and winter territorial species develop a pre‐alternate molt, it mainly occurs in early winter. Migratory behavior has also been suggested to impose time constraints on the pre‐basic molt (de la Hera et al., 2009; Kiat & Sapir, 2017). Therefore, the association between the occurrence of the pre‐alternate molt and migration might be interpreted as support for the time constraints hypothesis. However, when a molt in early winter (November to December) was considered pre‐basic instead of pre‐alternate, the association remained robust, implying that a number of migratory species with a pre‐alternate molt do not molt in early winter. Therefore, although we cannot rule out that time constraints owing to migratory behavior promote the pre‐alternate molt, other selective forces related to migration (e.g., feather wear) must be involved.

Our analyses of correlated evolution showed that acquisition of both aerial foraging and winter territoriality occurs more frequently in species with a pre‐alternate molt, although the reverse order of events would be expected according to the time constraints hypothesis. Apparently, feather replacement in early winter enables some species to become territorial or aerial foragers. For example, molting might allow some species to develop conspicuous plumage for territorial defense during the nonbreeding season, although this explanation has been dismissed for North American passerines (Froehlich et al., 2005). An additional molt might also improve flight performance (Bridge, 2008) and hence aerial foraging efficiency. However, we see no reason why this molt should occur in early winter and not later in the season. As some of the results agree with the predictions (e.g., pre‐alternate molt is more frequent in species that feed on the wing and are territorial in winter), but others do not (e.g., acquisition of the pre‐alternate molt precedes that of aerial foraging or winter territoriality), we conclude that this study provides only partial support for the time constraints hypothesis.

### 4.5 Possible differences with other geographical areas

Although the results of this study refer exclusively to European breeding passerines, we suggest they could be extrapolated to other geographical areas, bearing in mind that the relative importance of some factors may differ dramatically among regions or continents. For example, as migration is less frequent in species breeding at lower latitudes (Garamszegi et al., 2008), migratory behavior likely has less influence on the molt patterns of tropical bird species compared to those from the Palearctic and the Nearctic (i.e., temperate regions). Similarly, North American passerines seem to be more colorful than their European counterparts (Willson & von Neumann, 1972), possibly because the sexual selection is more intense in those species. If this were the case, we would expect sexual selection to play a more important role in molt evolution in North America than in European passerines. Another difference between the two continents is the wintering habitat of migratory species, mainly tropical forest for North American species and open savannah for European ones, and its possible implications on feather wear and molt patterns (Froehlich et al., 2005; Rohwer et al., 2005). Similar studies in different biogeographic realms or continents would allow us to obtain a more comprehensive picture of the factors that promote the pre‐alternate molt in passerines.

## 5 CONCLUSION

Our results suggest that sexual selection, feather wear, and time constraints play a role in the acquisition of the pre‐alternate molt in European passerines. By contrast, nonsexual social selection during the nonbreeding season may have the opposite effect, making this molt less probable. In short, the occurrence of the pre‐alternate molt appears to be influenced by different selective pressures, some with opposite effects, operating throughout the year. This study provides support for the feather wear hypothesis, partial support for the sexual selection and time constraints hypotheses, and no support for the social selection hypothesis to explain the evolution of the pre‐alternate molt.

## AUTHOR CONTRIBUTIONS

**José J. Cuervo:** Conceptualization (equal); data curation (equal); formal analysis (lead); funding acquisition (supporting); investigation (lead); methodology (equal); writing – original draft (lead); writing – review and editing (lead). **Judith Morales:** Conceptualization (equal); data curation (equal); formal analysis (supporting); funding acquisition (lead); investigation (supporting); methodology (equal); writing – original draft (supporting); writing – review and editing (supporting). **Juan J. Soler:** Conceptualization (equal); data curation (equal); formal analysis (supporting); funding acquisition (supporting); investigation (supporting); methodology (equal); writing – original draft (supporting); writing – review and editing (supporting). **Juan Moreno:** Conceptualization (equal); data curation (equal); funding acquisition (supporting); investigation (supporting); methodology (equal); writing – original draft (supporting); writing – review and editing (supporting).

## CONFLICT OF INTEREST

The authors have no conflict of interest to declare.

## Supporting information


Appendix S1

Figures S1–S6

Table S1

Table S2

Table S3

Table S4

## Data Availability

The data that support the findings of this study are available in the Supporting information files and are also archived in Dryad (https://doi.org/10.5061/dryad.m905qfv4c).
