# Supplementary material for: Sexual selection, feather wear, and time constraints on the pre‐basic molt explain the acquisition of the pre‐alternate molt in European passerines
Source: Ecol Evol. 2022 Sep 6;12(9):e9260. doi: 10.1002/ece3.9260 (PMC9448967; doi:10.1002/ece3.9260)
Supplement: Supplementary file 1 — Appendix S1 [file ECE3-12-e9260-s002.docx]

Appendix S1. One hundred random phylogenetic trees (downloaded from <http://birdtree.org/> and based on the Ericson All Species backbone phylogeny) for (A) the 188 passerine species included in the study (pages 1-69), (B) the 162 species showing non-territorial winter behavior (pages 70-129), (C) the 83 species with pre-alternate molt when molt in November or December is considered pre-alternate (pages 130-161), and (D) the 67 species with pre-alternate molt when molt in November or December is considered pre-basic (pages 162-187).

(A) One hundred phylogenetic trees for all passerine species included in the study (188 species).

#NEXUS

BEGIN TAXA;

TITLE Taxa;

DIMENSIONS NTAX=188;

TAXLABELS

Cyanopica_cyanus Perisoreus_infaustus Garrulus_glandarius Nucifraga_caryocatactes Corvus_monedula Corvus_dauuricus Corvus_frugilegus Corvus_corax Corvus_corone Pica_pica Pyrrhocorax_pyrrhocorax Pyrrhocorax_graculus Lanius_collurio Lanius_minor Lanius_senator Lanius_nubicus Lanius_excubitor Oriolus_oriolus Phylloscopus_sibilatrix Phylloscopus_bonelli Phylloscopus_trochilus Phylloscopus_collybita Phylloscopus_inornatus Phylloscopus_proregulus Phylloscopus_trochiloides Phylloscopus_borealis Cettia_cetti Aegithalos_caudatus Riparia_riparia Delichon_urbicum Hirundo_rustica Hirundo_rupestris Hippolais_icterina Hippolais_polyglotta Hippolais_olivetorum Acrocephalus_scirpaceus Acrocephalus_dumetorum Acrocephalus_agricola Acrocephalus_schoenobaenus Acrocephalus_melanopogon Acrocephalus_paludicola Acrocephalus_arundinaceus Acrocephalus_aedon Hippolais_caligata Hippolais_pallida Locustella_certhiola Locustella_fasciolata Locustella_fluviatilis Locustella_luscinioides Locustella_naevia Locustella_lanceolata Acrocephalus_palustris Sylvia_nana Sylvia_nisoria Sylvia_curruca Sylvia_hortensis Sylvia_cantillans Sylvia_melanocephala Sylvia_rueppelli Sylvia_conspicillata Sylvia_sarda Sylvia_undata Sylvia_communis Sylvia_atricapilla Sylvia_borin Panurus_biarmicus Galerida_cristata Galerida_theklae Alauda_arvensis Lullula_arborea Calandrella_rufescens Calandrella_brachydactyla Eremophila_alpestris Melanocorypha_bimaculata Melanocorypha_leucoptera Melanocorypha_yeltoniensis Melanocorypha_calandra Remiz_pendulinus Parus_caeruleus Parus_cyanus Parus_montanus Parus_cinctus Parus_lugubris Parus_ater Parus_cristatus Parus_major Parus_palustris Troglodytes_troglodytes Tichodroma_muraria Certhia_brachydactyla Certhia_familiaris Sitta_europaea Turdus_iliacus Turdus_merula Turdus_torquatus Turdus_ruficollis Turdus_pilaris Turdus_obscurus Turdus_philomelos Turdus_viscivorus Zoothera_sibirica Zoothera_dauma Luscinia_svecica Luscinia_luscinia Luscinia_megarhynchos Saxicola_rubetra Saxicola_torquatus Oenanthe_deserti Oenanthe_pleschanka Oenanthe_hispanica Oenanthe_oenanthe Oenanthe_isabellina Oenanthe_leucura Monticola_saxatilis Monticola_solitarius Phoenicurus_ochruros Phoenicurus_phoenicurus Luscinia_calliope Ficedula_albicollis Ficedula_hypoleuca Ficedula_parva Erithacus_rubecula Erythropygia_galactotes Muscicapa_dauurica Muscicapa_striata Tarsiger_cyanurus Cinclus_cinclus Sturnus_roseus Sturnus_vulgaris Bombycilla_garrulus Regulus_ignicapilla Regulus_regulus Motacilla_flava Motacilla_citreola Motacilla_alba Motacilla_cinerea Anthus_richardi Anthus_campestris Anthus_spinoletta Anthus_pratensis Anthus_cervinus Anthus_trivialis Fringilla_montifringilla Fringilla_coelebs Pinicola_enucleator Pyrrhula_pyrrhula Carpodacus_roseus Carpodacus_erythrinus Carduelis_carduelis Carduelis_citrinella Loxia_leucoptera Loxia_curvirostra Carduelis_flammea Carduelis_hornemanni Carduelis_cannabina Carduelis_flavirostris Carduelis_spinus Serinus_serinus Carduelis_chloris Bucanetes_githagineus Coccothraustes_coccothraustes Calcarius_lapponicus Plectrophenax_nivalis Emberiza_cirlus Emberiza_citrinella Emberiza_leucocephalos Emberiza_hortulana Emberiza_caesia Emberiza_cia Emberiza_cioides Miliaria_calandra Emberiza_melanocephala Emberiza_bruniceps Emberiza_rutila Emberiza_aureola Emberiza_spodocephala Emberiza_pusilla Emberiza_chrysophrys Emberiza_rustica Emberiza_schoeniclus Emberiza_pallasi Montifringilla_nivalis Petronia_petronia Passer_hispaniolensis Passer_domesticus Passer_montanus Prunella_modularis Prunella_collaris;

END;

BEGIN TREES;

Title 'Trees from "output.nex"';

ID 017684a99e8f1;

LINK Taxa = Taxa;

TRANSLATE

[0] 1 Cyanopica_cyanus,

[1] 2 Perisoreus_infaustus,

[2] 3 Garrulus_glandarius,

[3] 4 Nucifraga_caryocatactes,

[4] 5 Corvus_monedula,

[5] 6 Corvus_dauuricus,

[6] 7 Corvus_frugilegus,

[7] 8 Corvus_corax,

[8] 9 Corvus_corone,

[9] 10 Pica_pica,

[10] 11 Pyrrhocorax_pyrrhocorax,

[11] 12 Pyrrhocorax_graculus,

[12] 13 Lanius_collurio,

[13] 14 Lanius_minor,

[14] 15 Lanius_senator,

[15] 16 Lanius_nubicus,

[16] 17 Lanius_excubitor,

[17] 18 Oriolus_oriolus,

[18] 19 Phylloscopus_sibilatrix,

[19] 20 Phylloscopus_bonelli,

[20] 21 Phylloscopus_trochilus,

[21] 22 Phylloscopus_collybita,

[22] 23 Phylloscopus_inornatus,

[23] 24 Phylloscopus_proregulus,

[24] 25 Phylloscopus_trochiloides,

[25] 26 Phylloscopus_borealis,

[26] 27 Cettia_cetti,

[27] 28 Aegithalos_caudatus,

[28] 29 Riparia_riparia,

[29] 30 Delichon_urbicum,

[30] 31 Hirundo_rustica,

[31] 32 Hirundo_rupestris,

[32] 33 Hippolais_icterina,

[33] 34 Hippolais_polyglotta,

[34] 35 Hippolais_olivetorum,

[35] 36 Acrocephalus_scirpaceus,

[36] 37 Acrocephalus_dumetorum,

[37] 38 Acrocephalus_agricola,

[38] 39 Acrocephalus_schoenobaenus,

[39] 40 Acrocephalus_melanopogon,

[40] 41 Acrocephalus_paludicola,

[41] 42 Acrocephalus_arundinaceus,

[42] 43 Acrocephalus_aedon,

[43] 44 Hippolais_caligata,

[44] 45 Hippolais_pallida,

[45] 46 Locustella_certhiola,

[46] 47 Locustella_fasciolata,

[47] 48 Locustella_fluviatilis,

[48] 49 Locustella_luscinioides,

[49] 50 Locustella_naevia,

[50] 51 Locustella_lanceolata,

[51] 52 Acrocephalus_palustris,

[52] 53 Sylvia_nana,

[53] 54 Sylvia_nisoria,

[54] 55 Sylvia_curruca,

[55] 56 Sylvia_hortensis,

[56] 57 Sylvia_cantillans,

[57] 58 Sylvia_melanocephala,

[58] 59 Sylvia_rueppelli,

[59] 60 Sylvia_conspicillata,

[60] 61 Sylvia_sarda,

[61] 62 Sylvia_undata,

[62] 63 Sylvia_communis,

[63] 64 Sylvia_atricapilla,

[64] 65 Sylvia_borin,

[65] 66 Panurus_biarmicus,

[66] 67 Galerida_cristata,

[67] 68 Galerida_theklae,

[68] 69 Alauda_arvensis,

[69] 70 Lullula_arborea,

[70] 71 Calandrella_rufescens,

[71] 72 Calandrella_brachydactyla,

[72] 73 Eremophila_alpestris,

[73] 74 Melanocorypha_bimaculata,

[74] 75 Melanocorypha_leucoptera,

[75] 76 Melanocorypha_yeltoniensis,

[76] 77 Melanocorypha_calandra,

[77] 78 Remiz_pendulinus,

[78] 79 Parus_caeruleus,

[79] 80 Parus_cyanus,

[80] 81 Parus_montanus,

[81] 82 Parus_cinctus,

[82] 83 Parus_lugubris,

[83] 84 Parus_ater,

[84] 85 Parus_cristatus,

[85] 86 Parus_major,

[86] 87 Parus_palustris,

[87] 88 Troglodytes_troglodytes,

[88] 89 Tichodroma_muraria,

[89] 90 Certhia_brachydactyla,

[90] 91 Certhia_familiaris,

[91] 92 Sitta_europaea,

[92] 93 Turdus_iliacus,

[93] 94 Turdus_merula,

[94] 95 Turdus_torquatus,

[95] 96 Turdus_ruficollis,

[96] 97 Turdus_pilaris,

[97] 98 Turdus_obscurus,

[98] 99 Turdus_philomelos,

[99] 100 Turdus_viscivorus,

[100] 101 Zoothera_sibirica,

[101] 102 Zoothera_dauma,

[102] 103 Luscinia_svecica,

[103] 104 Luscinia_luscinia,

[104] 105 Luscinia_megarhynchos,

[105] 106 Saxicola_rubetra,

[106] 107 Saxicola_torquatus,

[107] 108 Oenanthe_deserti,

[108] 109 Oenanthe_pleschanka,

[109] 110 Oenanthe_hispanica,

[110] 111 Oenanthe_oenanthe,

[111] 112 Oenanthe_isabellina,

[112] 113 Oenanthe_leucura,

[113] 114 Monticola_saxatilis,

[114] 115 Monticola_solitarius,

[115] 116 Phoenicurus_ochruros,

[116] 117 Phoenicurus_phoenicurus,

[117] 118 Luscinia_calliope,

[118] 119 Ficedula_albicollis,

[119] 120 Ficedula_hypoleuca,

[120] 121 Ficedula_parva,

[121] 122 Erithacus_rubecula,

[122] 123 Erythropygia_galactotes,

[123] 124 Muscicapa_dauurica,

[124] 125 Muscicapa_striata,

[125] 126 Tarsiger_cyanurus,

[126] 127 Cinclus_cinclus,

[127] 128 Sturnus_roseus,

[128] 129 Sturnus_vulgaris,

[129] 130 Bombycilla_garrulus,

[130] 131 Regulus_ignicapilla,

[131] 132 Regulus_regulus,

[132] 133 Motacilla_flava,

[133] 134 Motacilla_citreola,

[134] 135 Motacilla_alba,

[135] 136 Motacilla_cinerea,

[136] 137 Anthus_richardi,

[137] 138 Anthus_campestris,

[138] 139 Anthus_spinoletta,

[139] 140 Anthus_pratensis,

[140] 141 Anthus_cervinus,

[141] 142 Anthus_trivialis,

[142] 143 Fringilla_montifringilla,

[143] 144 Fringilla_coelebs,

[144] 145 Pinicola_enucleator,

[145] 146 Pyrrhula_pyrrhula,

[146] 147 Carpodacus_roseus,

[147] 148 Carpodacus_erythrinus,

[148] 149 Carduelis_carduelis,

[149] 150 Carduelis_citrinella,

[150] 151 Loxia_leucoptera,

[151] 152 Loxia_curvirostra,

[152] 153 Carduelis_flammea,

[153] 154 Carduelis_hornemanni,

[154] 155 Carduelis_cannabina,

[155] 156 Carduelis_flavirostris,

[156] 157 Carduelis_spinus,

[157] 158 Serinus_serinus,

[158] 159 Carduelis_chloris,

[159] 160 Bucanetes_githagineus,

[160] 161 Coccothraustes_coccothraustes,

[161] 162 Calcarius_lapponicus,

[162] 163 Plectrophenax_nivalis,

[163] 164 Emberiza_cirlus,

[164] 165 Emberiza_citrinella,

[165] 166 Emberiza_leucocephalos,

[166] 167 Emberiza_hortulana,

[167] 168 Emberiza_caesia,

[168] 169 Emberiza_cia,

[169] 170 Emberiza_cioides,

[170] 171 Miliaria_calandra,

[171] 172 Emberiza_melanocephala,

[172] 173 Emberiza_bruniceps,

[173] 174 Emberiza_rutila,

[174] 175 Emberiza_aureola,

[175] 176 Emberiza_spodocephala,

[176] 177 Emberiza_pusilla,

[177] 178 Emberiza_chrysophrys,

[178] 179 Emberiza_rustica,

[179] 180 Emberiza_schoeniclus,

[180] 181 Emberiza_pallasi,

[181] 182 Montifringilla_nivalis,

[182] 183 Petronia_petronia,

[183] 184 Passer_hispaniolensis,

[184] 185 Passer_domesticus,

[185] 186 Passer_montanus,

[186] 187 Prunella_modularis,

[187] 188 Prunella_collaris;

TREE tree_1759 = ((((((1:18.4535718,2:18.453571795000002):1.846585121,(3:17.333779511,((4:13.746323641,((5:3.659689514,6:3.6596895136000005):7.890920663,((7:6.727986877,8:6.727986876299999):1.4115800173,9:8.139566894000001):3.4110432828999997):2.1957134634999997):1.745949953,10:15.492273595999999):1.8415059166999999):2.9663774059000003):1.612720497,(11:10.67460342,12:10.67460342):11.23827399):6.419522699,(((13:8.706034821100001,(14:5.663370714,15:5.663370714):3.0426641071):1.022032765,16:9.728067586):1.372451206,17:11.1005187919):17.231881323699998):4.2407367869,18:32.5731369006):20.701229939199997,((((((((((((19:5.317418303,20:5.317418302499999):9.690103063,(21:6.625662729,22:6.6256627287):8.381858636):0.2492374218,(23:7.79061806961,24:7.7906180688):7.466140719):1.573358932,(25:12.159739141,26:12.159739136599999):4.6703785820000006):6.282185109,27:23.112302827):2.837014109,28:25.949316938):5.6863431627,(29:18.841313285,(30:16.528259208,(31:13.5659261153,32:13.565926114):2.9623330930000003):2.3130540760000002):12.794346817000001):2.713749591,((((33:6.42284621,34:6.42284620988):3.506048349,35:9.9288945592):6.949088359,(((((36:7.560581751,37:7.5605817517):1.308809068,38:8.86939082):2.863042478,((39:6.690184572,40:6.690184572):2.299876762,41:8.9900613328):2.742371964):1.901395773,42:13.63382907):2.311506847,(43:15.03403906,(44:9.345568384,45:9.3455683838):5.688470676900001):0.9112968577):0.93264700081):14.17348037,(((46:13.5252411911,47:13.5252411946):4.945391909,((48:6.806531139,49:6.806531139):4.077400847,(50:10.405283623019999,51:10.405283622999999):0.478648363):7.5867011146):3.7897539030000003,52:22.260386999899996):8.791076283999999):3.2979464115):0.4697804042,((53:11.40048201,((54:10.21731673,(55:7.688556772,56:7.6885567728):2.528759954):1.164187927,(((57:3.022598707,58:3.022598706):4.013016325,59:7.0356150307):1.042422126,((60:6.447758174,(61:3.875362292,62:3.875362292):2.572395881):0.7667458299,63:7.214504004):0.8635331534):3.303467495):0.01897735475):2.500355312,(64:10.98179564,65:10.981795635):2.919041684):20.918352782):1.6960447233,(66:26.60058714,((((67:5.139886611,68:5.13988661105):4.364643743,69:9.5045303539):1.465030788,70:10.96956114):1.5324054007,(((71:8.844801673,72:8.844801672300001):0.3391782759,73:9.183979948000001):2.533390799,(74:5.851773749,(75:2.486243602,(76:0.965602503,77:0.965602503):1.5206410996):3.365530147):5.8655969986):0.7845957948):14.098620597):9.914647683):10.06201294,(78:39.117382425,((79:2.663662542,80:2.663662542):16.33496999,(((((81:11.449362779000001,82:11.449362779500001):1.082491511,83:12.5318542894):2.195334778,84:14.727189068999998):0.2534246367,85:14.980613706):0.5504993576,(86:9.871370401,87:9.871370401):5.6597426625):3.467519473):20.118749891):7.4598653324):1.310422499,(((((((88:34.082488983,89:34.08248899):0.1188375918,(90:9.636266973,91:9.636266972):24.565059609000002):2.962120647,92:37.163447223):7.193001533,(((((((((93:10.35670026869,94:10.356700268):0.2805742962,(((95:4.060317814,96:4.060317814):1.885992255,97:5.946310069):1.1587454560700001,98:7.105055525699999):3.5322190395999997):3.760806133,99:14.398080699000001):2.175626687,100:16.57370738):3.1321032162,101:19.7058106044):0.9705782272,102:20.67638882711):10.789940093,(((((103:14.62688284,(104:3.3155818,105:3.3155818):11.3113010462):4.053044079,((((((106:11.248595,107:11.2485950012):3.795024338,(((108:7.582979264,(109:0.1628276023,110:0.1628276023):7.420151662199999):1.70333114,(111:6.184306402,112:6.184306402):3.102004002):1.905425436,113:11.1917358403):3.8518834986):2.063234409,(114:2.956582726,115:2.956582726):14.150271020999998):0.3274096733,(116:6.479054937,117:6.4790549372):10.955208483):0.8641906593,118:18.29845408):0.08586503018,((119:1.923415966,120:1.923415966):7.540814536999999,121:9.4642305032):8.9200886075):0.29560781410000003):0.8463410191,122:19.526267945620003):0.9057632855,(123:16.0623652751,(124:9.6774083709,125:9.677408370799998):6.3849569045):4.3696659538):0.4329442261,126:20.864975455):10.6013534661):2.503859056,127:33.970187984):1.427072494,(128:10.090260854,129:10.090260853499998):25.306999615600002):8.959188287):1.67194835,130:46.028397113):0.2403272615,(131:28.48393669,132:28.48393668):17.784787685799998):1.209872798,(((((((133:1.90862615,134:1.90862615):0.6448946836,135:2.553520833):0.5974752716,136:3.1509961049):20.7036740317,((137:5.3974725368000005,138:5.3974725371):15.635283753,(((139:3.571743433,140:3.571743433):3.087102614,141:6.658846047599999):5.136741213,142:11.7955872609):9.23716903):2.821913845):9.580357304,(((143:8.049667459,144:8.04966746):22.29986699,(((145:12.520427497,146:12.520427497):7.555777606,(((147:16.54350745,148:16.5435074492):2.3464851048,((((149:6.963678521,150:6.963678521):2.372211198,((151:1.8707122319000002,152:1.8707122321):5.202898682,(153:0.4926477261,154:0.4926477261):6.580963187):2.262278806):0.4131383966,(((155:4.087930771,156:4.0879307715):3.926863001,157:8.014793771999999):1.115837123,158:9.130630895300001):0.6183972208):2.724031056,159:12.473059172200001):6.416933382):0.5736040328,160:19.463596586999998):0.6126085156):2.109030217,161:22.185235319):8.164299126):0.1535683937,((162:8.846586951,163:8.846586951):15.34460662,((((((164:6.347896634,(165:0.3125122291,166:0.3125122291):6.0353844049):1.190463375,(167:1.871556096,168:1.871556096):5.666803914):1.507435603,(169:1.82181286,170:1.8218128601369998):7.2239827523999995):1.046364896,171:10.09216051):1.984246751,(172:4.028818886,173:4.028818886):8.047588374):0.2922224835,((((((174:4.231716857,175:4.231716857):0.4205140058,176:4.6522308627):0.7399637231,177:5.3921945849999995):0.4506711792,178:5.842865764):0.5403955582,179:6.383261323):2.259119228,(180:5.606147057,181:5.606147057):3.0362334940999998):3.726249192):11.8225638236067):6.311909276):2.931924601):1.317406932,((182:18.674908199999997,183:18.6749082):2.935679046,((184:4.5929913014,185:4.5929913006):4.410612569,186:9.00360387):12.606983376):13.141847133999999):3.048571505,(187:0.4567359935,188:0.4567359935):37.3442698814225):9.677591287999999):0.4090730886):5.386696586199999);

TREE tree_4172 = (((((11:8.769136958,12:8.769136958):9.159385480000001,((1:15.94311933,2:15.943119326):0.3592237923,(((4:11.855285489,((5:2.330430475,6:2.3304304753):6.004980279,((8:4.2659507015,7:4.2659507014999996):0.9686097992,9:5.234560499800001):3.1008502535):3.519874734):1.204768647,10:13.060054136):0.3851899759,3:13.445244112000001):2.857099006):1.6261793217):5.488260564,((((14:4.754279483,15:4.75427948257):2.950770607,13:7.705050089923):0.9635908909,16:8.668640981):0.2192668174,17:8.887907798):14.5288752068):2.3634587064,18:25.780241707800002):20.2623281154,(((((((((((23:6.050695886000001,24:6.05069588648):5.476379974,(21:4.095060661,22:4.095060662):7.432015196799999):0.2923552761,(19:3.464132115,20:3.4641321144):8.355299021):1.34613164,(25:7.346387464,26:7.346387464):5.8191753117000005):3.583258467,27:16.748821241999998):3.0196926635,28:19.768513907000003):5.179910874,((30:13.365770779999998,(31:11.8003595847,32:11.800359585):1.5654111956999999):2.5377110963,29:15.9034818762):9.044942902999999):3.621150388,(((64:8.1583989501,65:8.15839895):1.685849227,(((((61:2.232840504,62:2.2328405047):1.938808856,(60:3.597190684,63:3.597190684):0.5744586766):0.5481984094,((57:2.75582051,58:2.7558205095):0.9364647225,59:3.6922852322):1.027562538):1.824621865,53:6.544469635):0.05338449385,((55:4.165721233,56:4.1657212321):2.0215054202,54:6.187226653):0.4106274761):3.246394048):9.750382271,(((((34:2.920880601,33:2.920880601):1.130244734,35:4.0511253359):3.905569036,((((39:3.000553994,40:3.000553993):1.534597212,41:4.5351512052):1.562127517,((36:3.7782060339999997,37:3.7782060341):0.7012426054,38:4.4794486393):1.617830083):0.4249726222,42:6.5222513446):1.434443027):0.5369749406,(43:7.605731172,(45:6.3304160121,44:6.3304160110000005):1.27531516):0.8879381400999999):8.866185702,(((47:6.583044768000001,46:6.5830447673):2.099740722,((51:6.032482674,50:6.0324826742):0.07861285712,(49:3.387913427,48:3.387913427):2.723182104):2.5716899580000003):2.8695784803,52:11.552363971):5.807491043):2.2347754355):8.9749447136):3.7681768673000002,(66:24.25145327,(((72:9.373913570575,71:9.37391357):0.8082987399,73:10.182212310999999):0.3556408397,(((69:7.868358387,(67:3.6730417859999998,68:3.6730417859999998):4.19531660054):0.009382577487,70:7.877740964):1.702225035,(((75:4.102805831,76:4.1028058313):1.954322518,74:6.057128349):0.4017448592,77:6.458873208):3.12109279051):0.9578871508):13.7136001194):8.086298766999999):8.176239263,(78:34.408669399000004,((80:2.580552662,79:2.5805526617):15.75047863,(((((82:9.5277559823,81:9.5277559814):1.648437937,83:11.176193916999999):2.636555991,84:13.812749909899999):0.1129746688,85:13.925724577999999):0.3187765997,(86:7.949647219,87:7.949647219):6.294853959000001):4.086530113):16.077638105):6.105321904):0.5759187519,(((((((((101:15.48600356185,((100:10.95738082,99:10.95738082):1.727916732,((94:8.2066262305,93:8.206626230200001):0.3673081834,(((95:3.589644215,96:3.589644216):1.741636001,97:5.3312802158):0.4690811637,98:5.800361379800001):2.7735730334):4.111363141):2.800706006):0.5374335531,102:16.0234371141):5.821083066,((((((((((106:8.680431344,107:8.680431343271):3.5157550109999995,(((108:5.7738194935,(109:0.3646441602,110:0.3646441602):5.409175332799999):0.8906541814,(111:3.499151477,112:3.4991514771000003):3.165322197):1.986579686,113:8.651053360399999):3.5451329945000003):0.8996812466,(115:3.130233579,114:3.130233579):9.965634023):0.9234480004,(117:6.3257040807,116:6.32570408):7.693611521):0.8193943322,118:14.838709934):0.3059706445,(121:10.0090194723,(119:1.51701302,120:1.51701302):8.4920064522):5.1356611069):0.58494047986,((105:3.154106189,104:3.154106189):9.883055216,103:13.037161404999999):2.6924596525999998):0.2435074726,122:15.9731285346):0.9533720479,((125:6.7540904717,124:6.7540904718):6.02814936287,123:12.78223983565):4.144260744):0.005334884508,126:16.9318354647):4.912684715899999):1.593256382,127:23.437776555299997):1.042140882,(128:6.3309661033,129:6.330966103180001):18.14895134647):11.3730406,((((90:6.661727423,91:6.6617274226):17.498324814,89:24.160052243000003):3.41703053,88:27.577082771500002):4.585129638,92:32.162212404):3.690745642):2.968172788,130:38.821130835):0.7606959693,(131:25.379003445000002,132:25.379003449000002):14.202823350000001):0.05902245673,(((((((139:4.0891203529,140:4.0891203531):0.7796466538,141:4.868767007):3.3142444060000003,142:8.183011411999999):6.799143577599999,(137:4.2434570721,138:4.243457073):10.738697917):1.1721983836,((135:2.548768088,(133:1.217421221,134:1.217421221):1.331346866):0.9469934067,136:3.495761494):12.658591879):12.21396868,((((143:7.565617391,144:7.565617391):15.1291701,((((((151:2.1944416739,152:2.1944416735):3.482757548,(154:0.8496397041,153:0.8496397041):4.827559518):2.471723477,((149:5.948689498,150:5.948689498499999):1.340511958,(((155:2.962057759,156:2.962057759):1.94794673,157:4.9100044877):2.142509516,158:7.0525140052):0.2366874514):0.8597212424):2.451362192,159:10.60028489):4.417767648,((160:13.673681177399999,(148:12.3464160398,147:12.346416039100001):1.3272651387):0.3086649167,(145:10.7576080648,146:10.75760806197):3.224738033):1.035706444):1.354957233,161:16.373009772):6.321777719):0.1219698288,((162:17.840579511,163:17.8405795047):3.035848473,(((171:9.464766663,(((164:6.06506841,(165:0.4203132027,166:0.4203132027):5.644755207):1.500787857,(168:1.896531937,167:1.896531937):5.66932433):0.5997573662,(169:1.643466926,170:1.64346692587):6.522146707):1.29915303):2.217498098,(172:4.850892506,173:4.850892506):6.831372255):0.003368573916,((((((174:3.373277491,177:3.3732774912):0.6746245543,175:4.0479020454):0.5297379625,176:4.577640008):0.7216200942,178:5.299260102):2.068364045,179:7.367624147):0.3975380844,(180:4.93185673,181:4.93185673):2.833305502):3.9204711031):9.1907946472962):1.940329332):3.454778192,((183:13.8456188657,182:13.845618865499999):6.866720428,((184:2.774828596,185:2.77482859652):2.523244943,186:5.29807354):15.414265754399999):5.5591962163551):2.096786543):3.642771106,(187:1.033599141,188:1.033599141):30.977494015799998):7.629756103):1.449060788):4.9526597782);

TREE tree_9521 = ((18:28.359639501799997,(((11:8.037188284,12:8.037188284):10.578171741,((1:14.57279287,2:14.572792872):2.494582948,(((4:11.74963535,((6:2.57743271558,5:2.57743271553):5.285768302,((7:4.59164971031,8:4.5916497105600005):1.56417523,9:6.1558249408):1.7073760765900001):3.8864343324999995):1.464887729,10:13.2145230798):0.9254178061,3:14.139940885):2.9274349347):1.5479842051000001):5.556066846,(((13:7.3462056264,(14:4.258535343,15:4.258535343):3.0876702823):0.7582616622,16:8.1044672879):0.7143976709,17:8.8188649593):15.352561911999999):4.188212628):22.0934066532,(((((((28:27.054221272,(((((21:4.514185177,22:4.514185177):10.1726361063,(23:7.9203795148000005,24:7.9203795151000005):6.766441768):0.5731459309,(19:5.895892416,20:5.895892416400001):9.364074798):2.923468213,(26:11.156536223540002,25:11.156536223):7.026899204):6.482943026,27:24.6663784516):2.3878428193):4.075431107,(((32:13.171970791,31:13.17197079077):2.53780664367,30:15.709777435):2.0252463488999997,29:17.73502378379):13.394628596):0.5997003175,((((47:12.02347399,46:12.023473988):2.7408174042,((50:9.5833458834,51:9.583345884):0.3727051285,(48:5.404511282,49:5.404511282):4.55153973041):4.80824037961):3.9401273459999997,52:18.704418742399998):11.5238563306,((((45:9.1466487589,44:9.146648759):2.325218688,43:11.47186745):1.934510889,((((37:6.099484611399999,36:6.099484611):1.132889917,38:7.232374527999999):3.686839631,((40:5.530039135,39:5.530039135):2.55812724457,41:8.0881663792):2.831047779):0.9457812106,42:11.86499537):1.5413829669):0.447911747,((33:2.842758323,34:2.8427583223):5.624966143,35:8.467724466):5.386565618):16.373984985):1.501077631527):0.3169104092,((64:10.69849699,65:10.6984969857):1.816683198,(((((58:4.429147787,57:4.429147787):1.132516897,59:5.561664684):1.018464848,((60:4.046823212,63:4.046823212):1.247180413,(61:2.490491504,62:2.490491504):2.803512121):1.286125907):2.409438953,53:8.989568484):0.8824991696,(54:9.465188098,(55:5.29488421,56:5.294884211):4.170303887399999):0.4068795556):2.64311253):19.531082919699998):2.9113358398,(66:28.64050166,((((70:13.94763846,((68:6.768094403,67:6.768094402999999):5.533997192,69:12.302091595):1.645546868):0.5031327421,((74:9.818450649,(77:3.92418399,75:3.92418399):5.8942666589999995):3.255961711,76:13.07441236):1.3763588454):1.160119512,(71:14.94516091,72:14.94516091):0.6657298071):0.6055525128,73:16.216443229):12.4240584305):6.317097287):4.608975296,(78:30.416240754999997,((79:2.632540546,80:2.632540546):13.87273511,(((84:12.074040469,85:12.074040469000002):0.4315699366,((81:9.772410648000001,82:9.772410648):0.6815554562,83:10.4539661049):2.051644302):0.6503320643,(87:5.802185607,86:5.802185607):7.3537568637):3.349333187):13.91096510274):9.15033348):4.442603635,((((((((102:15.9250723087,(101:15.192235971499999,(100:12.39431295,(((93:8.3820006644,94:8.382000664):0.2208053901,((97:4.426356087,(95:2.920183737,96:2.9201837369):1.506172351):1.35700371725,98:5.7833598052):2.8194462498):3.528608654,99:12.13141471):0.2628982388):2.797923024):0.7328363361):5.945561803,(126:16.331094495000002,(((((105:2.625989818,104:2.625989818):9.231726221,103:11.8577160347):2.638668313,((((((106:9.121908966,107:9.121908965700001):2.194728513,((((109:0.1574675351,110:0.1574675351):4.6545742754,108:4.8120418104):1.504712639,(111:3.535460631,112:3.5354606304000002):2.781293818):2.014958993,113:8.3317134411):2.9849240373):1.038063012,(114:2.201900227,115:2.2019002275):10.1528002635):0.1319621469,(117:4.9908445056,116:4.990844506):7.495818132):1.406608584,((120:1.43967419,119:1.43967419):6.4291558746,121:7.8688300643):6.0244411571):0.2531197286,118:14.146390950699999):0.34999340090000003):0.1734896842,122:14.66987404):0.98567862074,(123:12.464093199899999,(125:7.286511208099999,124:7.28651120775):5.1775819923):3.1914594565):0.6755418388):5.5395396148):0.8355832969,127:22.7062174105):0.1988269805,(128:6.9089000392,129:6.9089000385):15.996144351599998):14.9615159,(((89:19.054265097,(90:6.3022629689,91:6.302262968):12.7520021283):6.057390641,88:25.11165573665):4.897384165,92:30.009039907000002):7.857520388):3.598618993,130:41.4651792721):1.100819805,(131:23.186254638,132:23.186254644):19.379744447):0.1017031567,((((((143:7.462534944,144:7.462534944):15.65849417,((((145:12.150743267000001,146:12.150743267100001):3.751576759,(160:15.362359975,(147:12.7594510982,148:12.7594510982):2.60290887709):0.5399600522):0.04342550908,(159:10.552468787,(((155:4.8301788685,156:4.830178869):1.969626128,157:6.799804997):1.320018053,((((153:0.244758573,154:0.244758573):4.123472186,(151:1.9923950511,152:1.9923950509000001):2.375835708):3.102869357,(149:5.526474692,150:5.526474692):1.9446254235):0.3861050224,158:7.85720513807):0.2626179111):2.432645737302):5.393276749):2.63151236,161:18.577257896):4.5437712206):1.453931843,((162:18.7683938532,163:18.768393850000002):3.985962851,((((((164:4.794987876,(165:0.5050866827,166:0.5050866827):4.2899011931):1.715290355,(168:2.275399684,167:2.2753996834):4.234878547):1.346911898,(170:2.310221463,169:2.3102214638):5.546968665):0.8664518185,171:8.723641948):1.100388089,(173:3.977019174,172:3.977019174):5.847010862):0.7417089323,((179:6.450562938,(((174:5.337133364,(176:3.545374445,175:3.545374445):1.7917589186):0.09453562013,177:5.431668984):0.574083807,178:6.0057527914):0.4448101465):0.6646504715,(181:2.624815124,180:2.624815124):4.4903982846999995):3.450525559):12.1886177297487):1.820604264):3.559746814,((((134:3.146105504,133:3.146105504):0.6651795892,135:3.811285093):0.6964286685,136:4.507713760800001):15.871788744100002,((((140:3.93524087,139:3.9352408700000003):1.1603743106,141:5.095615181):5.765918839,142:10.8615340188):7.064403681,(137:4.4456278412,138:4.44562784):13.480309855):2.4535648073):7.755205262):0.7929730998,((183:13.3982428772,182:13.3982428725):4.418312532,((184:2.005451469,185:2.0054514688):4.7274162049,186:6.7328676728):11.083687731000001):11.111125465):4.510760275,(188:1.001852194,187:1.001852194):32.4365889494):9.2292610953):1.341475632):6.443868274);

TREE tree_0526 = ((18:30.7096901204,(((11:9.118227448,12:9.118227448):11.860057751,((1:15.29596607,2:15.295966065):2.337975682,(((((5:2.9270168605,6:2.9270168610000002):6.610950183,(9:5.570152923,(7:4.2327789165,8:4.2327789162):1.3373740061000001):3.9678141212):1.834832316,4:11.37279936):1.740956606,10:13.113755965):1.044342265,3:14.1580982314):3.4758435159000003):3.344343452):3.990131843,((16:9.2010548216,17:9.2010548224):0.4702971263,((15:4.302447866,14:4.302447866):2.9485938735,13:7.251041740000001):2.420310209):15.297065088999998):5.7412730787):22.269346380099996,((((66:33.40955163,(((71:11.367752863,72:11.367752864):1.951431133,((((68:6.264277921,67:6.26427792021):3.125078886,69:9.389356808):1.485781561,70:10.87513837):0.8399978732,(74:9.584020566,((77:3.966858537,76:3.9668585371000002):4.797413476,75:8.764272013):0.8197485538):2.1311156765):1.6040477536400002):3.576474363,73:16.8956583565):16.513893272):8.327409662,(((((31:14.3521663368,32:14.352166336):1.9914474396,30:16.343613775999998):2.293761317,29:18.6373750932):12.456030957900001,(((((19:4.583449752,20:4.5834497517):11.17444803,((21:6.594309678,22:6.594309678):9.110943768,(23:9.9763929209,24:9.9763929208):5.7288605265):0.05264433618):2.417001245,(25:11.9839672737,26:11.983967274):6.190931754899999):5.457899771,27:23.632798796):1.789847709,28:25.422646508):5.6707595416):4.962862019,(((64:12.67843649,65:12.6784364917):7.015153109,(53:14.8105172,((54:11.823204,(55:7.066233737,56:7.066233735999999):4.7569702629999995):0.4202467836,(((57:4.401303355,58:4.401303355):3.042347598,((61:4.329577593,62:4.329577593):1.805177811,(63:5.748029797,60:5.748029797):0.3867256075):1.308895549):1.403065601,59:8.8467165543):3.396734229):2.567066413):4.883072406):8.660781443000001,((((47:8.882205462,46:8.882205462):3.023998427,((50:8.2342068146,51:8.234206815):0.1397684732,(49:5.932868497,48:5.93286849738):2.441106791):3.5322286011):5.567495017,52:17.4736989033):7.7427101689999995,((((((37:6.393473415,36:6.3934734148):0.4238172875,38:6.8172907017):2.769227813,(41:6.922553199,(40:5.456748025,39:5.456748025):1.465805175):2.6639653152):0.6220028814,42:10.2085213965):1.720461964,(43:10.24442496,(44:8.506639004,45:8.5066390044):1.737785955):1.6845584016):0.9119323832,((34:3.992090486,33:3.9920904850000003):3.222137993,35:7.214228479):5.626687264):12.3754933355):3.1379619749):7.701897024):5.68069322):4.035501377,(78:42.939168981,((79:3.912693323,80:3.912693323):16.29449226,(((83:13.25229321,(82:10.2993785808,81:10.299378582000001):2.9529146260099997):3.588832938,(85:16.066892968,84:16.0668929682):0.7742331771):0.8958229751,(86:8.828378756,87:8.828378756):8.908570364):2.470236459):22.731983396):2.83329369):1.923865916,((((((((90:9.526885271,91:9.526885270769998):22.317904351,89:31.844789623):0.8633837429,88:32.7081733612):4.211005323,92:36.919178679):5.584391926,(((((((99:13.23079998,((93:9.3093350914,94:9.3093350916):0.1048697365,(((95:3.438233675,96:3.4382336748):2.524399382,97:5.962633057435825):1.019911678,98:6.982544734499999):2.4316600936):3.816595149):1.192583607,100:14.423383586):2.0759331999999997,101:16.49931678):0.8574946917,102:17.356811476700003):7.326692223,(((((((121:6.8306458779,(119:1.105944996,120:1.105944996):5.7247008823000005):7.8598462466,118:14.690492125):0.07785549684,((((106:7.748517356,107:7.7485173561):4.563582122,(((111:4.295091896,112:4.295091896821):2.777425043,((109:0.1694014327,110:0.1694014327):6.5780377668,108:6.7474391998000005):0.3250777395):2.05587694339,113:9.128393883389998):3.1837055950999997):0.7539106511,(114:3.060055577,115:3.060055577):10.0059545533):0.5183084764,(116:5.6632085989,117:5.6632086):7.921110006000001):1.184029016):0.9922626543299999,((105:2.233295952,104:2.233295952):10.69666659,103:12.929962538999998):2.8306477366):0.405648258,122:16.1662585372):0.941589296,(123:12.905471899,(124:9.252229485,125:9.2522294852):3.6532424136999997):4.2023759304):0.8205848326,126:17.928432662):6.7550710377000005):2.124682891,127:26.808186598):2.755063898,(129:8.7859988647,128:8.785998864):20.7772516258):12.94032012):1.841149376,130:44.344719989):1.594223835,(132:40.397453181,131:40.39745318):5.541490641999999):0.8774697921,((((((138:10.369674715,137:10.369674714999999):13.1141350779,(142:19.0020072192,((140:6.345301006,139:6.345301006):5.009954805,141:11.355255810200001):7.6467514125000005):4.481802572):4.921464342,(((133:5.415444504,134:5.415444504):1.22818032,135:6.643624824):3.736203774,136:10.379828597):18.025445538):7.176313953,(((143:9.956604008,144:9.956604008):20.39237216,(((((((151:2.5538829402,152:2.5538829396):3.607605298,(153:0.3796650874,154:0.3796650874):5.78182315):3.087724703,(150:5.904953255,149:5.904953255):3.3442596852):1.782734588,(((155:5.529248216,156:5.5292482168):3.458802139,157:8.9880503552):1.786618771,158:10.7746691276):0.2572784018):3.468252701,159:14.50020023):7.812748209,(((146:17.50028002747,145:17.5002800284):3.862536259,(147:17.090676174000002,148:17.0906761735):4.272140111600001):0.7105936515,160:22.073409933):0.2395385017):2.611918739,161:24.924867178):5.4241089865):0.5220467452,((162:21.946472314,163:21.94647232116):4.615066983,(((171:12.04324124,(((164:7.154071422,(165:0.914260002,166:0.914260002):6.239811421):1.34303167,(167:2.270062077,168:2.270062077):6.227041015):2.580367202,(169:3.51513644,170:3.5151364403):7.562333853):0.9657709479):3.815598313,(173:5.848598282,172:5.848598282):10.0102412727):0.5415561824,(((((175:5.581534366,176:5.581534366):1.5097664255,(174:5.920412927,177:5.920412927):1.1708878645):0.33534705,178:7.426647842):0.7817899904,179:8.208437832000001):1.190581099,(181:4.120126927,180:4.120126926499999):5.2788920048):7.001376805):10.161143567000002):4.309483607):4.710565184):0.7699799319,((183:22.3443195269,182:22.344319530999996):1.712270643,(186:5.239543696,(185:2.6223959449,184:2.62239594475):2.617147752):18.81704647622):12.294977857):3.058398396,(187:0.05687415324,188:0.05687415324):39.3530922696):7.4064471918):0.8799149681):5.282707915);

TREE tree_1894 = ((18:30.346836600753,(((11:9.187932955,12:9.187932955):11.903028349,((1:17.5694569,2:17.569456902):2.300249178,(((((6:1.79984676,5:1.7998467603000001):8.26861,((7:5.0870018542,8:5.0870018543):0.8063838474,9:5.8933857021):4.1750710590999995):3.26010217,4:13.32855893):2.635026172,10:15.963585102):1.207109133,3:17.170694239):2.6990118450000002):1.2212552236):5.356435137,((((14:5.008278081,15:5.00827808):4.057767213,13:9.066045292999998):0.5659443465,16:9.631989639):0.0632085,17:9.69519814017):16.7521982985):3.8994401609000002):20.8016142522,((((66:26.14444476,((((71:10.0751171384,72:10.075117138):0.7374327059,(((68:4.447568993,67:4.447568994):3.2632175097999996,69:7.71078650284):0.9229756132,70:8.633762117):2.178787727):0.08366201332,((74:6.526543248,77:6.526543247999999):4.345850077,(76:7.687883282,75:7.6878832820000005):3.184510043):0.02381853258):1.146049397,73:12.042261251):14.102183506):15.01339501,(((((((19:3.188877373,20:3.1888773729):8.568834047,((21:4.278422274,22:4.2784222750000005):7.2835592332,(24:6.6970960358,23:6.697096036):4.864885471):0.1957299129):1.396445915,(25:8.730209110699999,26:8.73020911111):4.423948224):3.366181633,27:16.520338967300003):2.6484688298,28:19.168807798):6.674316032,(29:17.442574369,(30:14.982222306,(32:12.380307265999999,31:12.380307265410002):2.6019150408):2.4603520633000002):8.400549461):8.697536165,(((64:8.659086858,65:8.6590868581):4.693954358,(54:9.898210978,((53:7.266214444,((((61:3.300654782,62:3.300654782):1.001073156,63:4.301727938):0.03311510605,60:4.3348430446):1.072053605,((57:4.064406897,58:4.0644068959999995):1.33810675,59:5.402513646):0.004383002619):1.859317795):1.903193134,(55:5.2352635344000005,56:5.235263535):3.9341440433):0.7288034):3.454830238):15.4240987435,((((34:4.343457139,33:4.343457139):3.11189774,35:7.455354878):4.580717551,(((45:8.2371714681,44:8.237171469):1.902001748,43:10.1391732161):1.159360637,((((40:3.994253987,39:3.994253987):1.58486707,41:5.5791210573):3.46195422,((37:5.239620908,36:5.239620908100001):0.5636043078,38:5.803225214999999):3.237850062):0.8465665763,42:9.887641853000002):1.4108920003999998):0.7375385756):14.894109714999999,(52:18.801372711899997,((51:9.186964568,(50:9.0275671789,(48:5.357635155,49:5.3576351542000005):3.6699320243):0.1593973886):3.134377879,(47:10.3516015963,46:10.351601596):1.969740851):6.4800302647):8.128809436000001):1.8469578123000001):5.7635200348):6.617179774):3.754267365,(((80:1.853746623,79:1.853746623):18.22754853,((((81:10.9616480739,82:10.9616480748):2.2644230213,83:13.2260710967):1.854881513,(84:15.029964907,85:15.0299649102):0.05098769768):1.838850802,(87:9.906895016,86:9.906895016):7.0129083941):3.161491744):17.944435857000002,78:38.025731006):6.886376119):1.752472526,(((((((89:25.796596625,(90:5.490282093,91:5.490282093):20.306314528):3.426136531,88:29.2227331564):5.004322094,92:34.2270552486):6.807346833,((((((((((((95:4.657936491,96:4.65793649):0.8565440764,97:5.514480567):1.38483280215,98:6.8993133692):2.6019787713,93:9.501292141100002):0.4203391233,94:9.921631264):3.940973718,99:13.86260498):0.2997623049,100:14.16236729):3.5339114259999995,101:17.69627871):0.3926887014,102:18.0889674146):8.688414077000001,((((((((((106:9.194330394,107:9.194330394899998):4.7276084515,(((111:4.695118866,112:4.6951188659):3.75967371,(108:6.388138322350001,(109:0.2485649548,110:0.2485649548):6.1395733667):2.066654254):1.220489786,113:9.67528236114):4.2466564841):1.165467108,(114:3.855855352,115:3.855855352):11.231550602999999):0.6035298904,(117:6.5720206040199995,116:6.5720206044):9.11891524):0.9928682177,118:16.683804063):0.6695415763,(121:9.3272869318,(120:1.500719837,119:1.500719837):7.8265670940000005):8.026058707299999):0.5487657374,((104:3.414198733,105:3.414198733):11.06979553,103:14.483994259):3.4181171160000003):0.2708486158,122:18.172959991000003):1.11456666,((125:9.1006098288,124:9.1006098285):5.75037361049,123:14.850983441):4.4365432116):0.2729342334,126:19.560460884):7.216920605):2.482637864,127:29.260019358):0.3659189169,(129:8.7484750259,128:8.748475026):20.8774632434):11.40846381):2.495039876,130:43.529441955):2.501109089,(132:20.502774318,131:20.502774318):25.527776730999996):0.3159312398,((((((135:3.05662272,(133:1.370104593,134:1.370104593):1.6865181277):1.247245772,136:4.3038684923999995):18.901720520999998,(((141:5.536579809,(140:3.9126793298999996,139:3.9126793296):1.62390048):7.6114830809,142:13.14806289):6.4070016962,(137:4.40899619369,138:4.4089961934):15.1460683967):3.6505244240000003):8.87411049,(((143:8.882236539,144:8.882236539):19.86700011,((((((150:8.6595099745,149:8.659509974999999):0.5765074794,((151:1.6877315057,152:1.6877315059999998):4.374067446,(154:0.4818446587,153:0.4818446586):5.5799542936):3.174218502):0.3927920262,(((156:3.439745404,155:3.4397454039):4.480889759,157:7.920635163):1.544784254,158:9.465419417600001):0.163390063):2.320138638,159:11.948948118):8.217112164,(((146:14.2001047616,145:14.200104762):3.808353998,160:18.008458759):1.894277296,(148:16.602141546000002,147:16.6021415471):3.3005945092):0.2633242258):2.682199122,161:22.8482594):5.90097725):1.180119197,((162:19.187073968,163:19.1870739659):3.427534686,(((171:9.291926767,(((164:4.821604144,(165:0.251652836,166:0.251652836):4.569951308):2.814685638,(168:3.050403217,167:3.050403217):4.5858865648):0.7578547842,(169:1.855079551,170:1.85507955056):6.539065016):0.8977822008):1.828435924,(172:3.905926985,173:3.905926985):7.2144357056):0.2384511587,((179:7.446187905,((174:4.834413886,((176:3.99558654,175:3.9955865398999997):0.5724693095,177:4.5680558499999995):0.2663580367):0.7159113264,178:5.550325213000001):1.895862693):0.7544197591,(181:4.971108029,180:4.971108029):3.229499635):3.158206185):11.2557948012346):7.314747201):2.150343655):1.323743115,((183:16.904951348,182:16.904951348):7.259435571,(186:6.636587122,(185:2.8215800281,184:2.8215800281):3.8150070947000003):17.527799797):9.239055701):6.345333348,(188:1.307509876,187:1.307509876):38.4412660876):6.597706318):0.3180973708):4.4838711945);

TREE tree_2810 = ((18:24.8300817089,(((11:6.958687245,12:6.958687245):9.58558519,((1:13.58118894,2:13.581188935):0.7697792458,(3:12.3866677161,((((6:2.382901349,5:2.3829013485):5.89697379368,((7:4.69618034,8:4.696180341):0.4869718947,9:5.183152235170001):3.0967229074000002):1.532266326,4:9.812141469):1.808303368,10:11.620444835999999):0.7662228799):1.9643004663):2.1933042533):3.555844788,((((14:3.629750624,15:3.629750624):2.5257596866,13:6.1555103110000005):0.4982640154,16:6.653774326):0.1606087853,17:6.814383111900001):13.285734111700002):4.7299644858136):25.1176900177,((((((((64:12.67971542,65:12.6797154202):4.79051987,((((63:8.39324349,(60:7.363167401,(61:3.43956473,62:3.43956473):3.923602672):1.030076089):0.6919013678,((57:5.573944288,58:5.573944288):2.237672046,59:7.811616334):1.273528523):4.974996832,53:14.06014169):0.9180088248,(54:12.39779229,(55:7.129192585,56:7.1291925846):5.268599706):2.580358224):2.492084775):11.2825499334,((((((19:4.891544667,20:4.8915446676):9.77328845,(23:9.761577171999999,24:9.7615771711):4.9032559459):1.169814461,(21:6.856329058,22:6.856329057):8.9783185213):1.478817759,(25:9.34914042137,26:9.34914042):7.964324915700001):6.661716195,27:23.9751815318):2.948138347,28:26.92331988):1.8294653514):5.949780065,((52:20.91638003777,(((48:8.078138131,49:8.078138131):3.270947411,(50:10.941375815899999,51:10.941375816999999):0.407709726):4.371635115,(47:12.636470419,46:12.6364704237):3.0842502339999998):5.1956593808000004):10.926737431,(((34:5.34102846,33:5.34102846):3.912113975,35:9.253142434):6.830877717,((((41:9.868108966,(40:6.998818508,39:6.998818508):2.8692904573):2.251859255,((37:6.873584114600001,36:6.8735841145):0.6536701209,38:7.527254235):4.592713985):0.4894750764,42:12.609443296600002):2.051195505,((45:10.611617737400001,44:10.611617737):2.998504277,43:13.610122013000002):1.0505167881):1.423381349):15.7590973151):2.8594478218):0.4253189531,(((31:17.7917748956,32:17.791774892):2.7476669699,30:20.539441867):2.064917807,29:22.604359672099996):12.523524568):3.512192943,(66:29.23144741,((((71:12.397934716199998,72:12.397934716):0.3071337465,(74:11.18818788,(75:6.655562556,(77:1.504163713,76:1.504163713):5.151398843):4.532625322):1.516880584):0.4581147063,(70:11.10514379,(69:8.933249455,(68:6.151404908,67:6.1514049074):2.781844547):2.171894338):2.05803937539):2.182509705,73:15.345692874):13.885754535999999):9.40862978):3.51160651,(78:33.961086963,((80:3.923118252,79:3.923118252):14.08507016,(((((81:11.050324431,82:11.0503244313):0.6592394385,83:11.70956386872):1.617044486,85:13.326608355000001):0.5872090153,84:13.9138173712):0.6333099912,(86:8.403910661,87:8.4039106607):6.1432167014):3.4610610449):15.9528985571):8.190596735):1.554350106,(((((((((((((((((107:14.0712968452,106:14.071296839999999):3.178710474,(((108:7.8291842053,(109:0.3947585928,110:0.3947585928):7.434425613):2.385967309,(111:5.186680249,112:5.1866802488):5.028471266):3.50036435,113:13.7155158643):3.5344914539000003):1.047032001,(114:3.83578102,115:3.83578102):14.4612583):1.492146034,(117:10.227811366,116:10.2278113669):9.561373987):3.001289015,118:22.79047437):0.2686107967,(121:10.2668021517,(119:1.58216574,120:1.58216574):8.6846364116):12.792283014199999):0.8477649536,((105:4.491104882,104:4.491104881999999):12.37430297,103:16.865407844):7.041442271999999):0.2040768328,122:24.110926957):1.213336183,((124:14.64344009517,125:14.643440092899999):4.121434240539999,123:18.764874333399998):6.559388803):0.57567136287,126:25.899934498):5.982397031,(102:21.6840300804,(101:20.0598465328,((100:15.6241629,99:15.6241629):3.036238024,((94:12.179310389900001,93:12.1793103854):0.03440153677,(98:7.3667985144,(97:5.717982461,(95:3.997396103,96:3.997396103):1.720586358):1.64881605306):4.84691341074):6.446688997):1.3994456083):1.6241835497000001):10.198301448999999):2.453358045,127:34.335689566):0.640459242,(129:11.1782186209,128:11.1782186145):23.7979301927):3.524964672,((((90:6.664430761,91:6.664430762):18.81882400685,89:25.483254767):2.824978896,88:28.3082336619):2.890385339,92:31.198619002999997):7.30249449):1.219212025,130:39.72032550590001):2.789464788,(132:27.506191892,131:27.506191899999997):15.003598404):0.1863521266,(((((((141:5.5547489848,(140:3.399819235,139:3.3998192351):2.1549297499):4.523946116,142:10.078695099699999):10.15822323,(137:6.396935283,138:6.3969352826999994):13.8399830485):2.1315869209,((135:2.693995352,(134:1.549125131,133:1.549125131):1.144870221):1.386511222,136:4.0805065745):18.287998678):9.039012789,(((143:8.394516551,144:8.394516551):19.52028862,(((((148:15.111059414,147:15.111059414):1.886626427,160:16.997685841):0.9306257747,(146:14.3321517931,145:14.332151793000001):3.596159822):1.601759181,(((((152:2.4778453848,151:2.477845385):4.620952944,(154:0.5693109113,153:0.5693109113):6.529487418):3.096406702,(150:5.833350818,149:5.833350818):4.361854213):0.5944509472,(((155:4.628041442200001,156:4.6280414424):4.294361987,157:8.922403429):1.136635913,158:10.0590393426):0.7306166353):2.946147328,159:13.735803306400001):5.79426749):2.06386497,161:21.593935767):6.320869408):0.6360729767,((162:22.9432570481,163:22.9432570509):3.108736029,((((((164:6.343799582,(166:0.7404182909,165:0.7404182909):5.603381291):3.670576475,(168:2.426584061,167:2.426584061):7.587791996):1.673745664,(169:3.736963823,170:3.7369638228):7.951157898):0.3842455267,171:12.07236725):3.645895734,(172:5.244383738,173:5.244383738):10.473879244):0.1805563451,((179:8.909823056,((177:6.2424834460000005,(176:3.643154093,(175:3.506498358,174:3.506498358):0.1366557348):2.5993293529):0.5432119238,178:6.785695369999999):2.124127686):3.069864705,(180:6.073587195,181:6.073587195):5.906100565):3.9191315667):10.1531737565):2.498885062):2.856639896):0.7776391893,((182:14.250074729900001,183:14.250074730000001):7.14095589,((185:1.874425146,184:1.8744251459999999):4.431933824,186:6.3063589704):15.08467165035):10.79412661):3.499517447,(188:0.198280063,187:0.198280063):35.4863946167375):7.011467746999999):1.009891379):6.2417379142);

TREE tree_7445 = (((((11:9.163521036,12:9.163521036):15.556880031999999,((1:18.42108824,2:18.421088239):3.040238135,(((((5:3.018108336,6:3.0181083369):9.164120199,((7:7.423158218999999,8:7.423158219499999):1.02058362,9:8.4437418385):3.7384866968):2.865068041,4:15.047296572):2.648066822,10:17.695363399):1.7906185615,3:19.485981961):1.9753444126000002):3.259074695):6.066942119,((((15:6.172898247,14:6.172898247):2.7439158412999998,13:8.916814088):0.874774943,16:9.7915890309):0.8085549744,17:10.6001440047):20.1871991821):4.629390952,18:35.416734145902694):16.8841687916,((((((29:20.400036742999998,((31:15.4922870458,32:15.492287046000001):1.7549779406,30:17.247264981):3.1527717561):11.915755768,(((((19:6.660825824,20:6.660825824):11.28531989,((23:10.025553004999999,24:10.0255530055):6.900331535,(21:6.304922311,22:6.304922311):10.62096223):1.020261175):1.776961735,(26:11.925244965000001,25:11.925244964000001):7.7978624864):5.982521337,27:25.705628787999995):2.203557642,28:27.909186431):4.4066060794510005):2.692987902,((((54:8.436077069,(55:4.020042056,56:4.0200420566999995):4.416035013):0.2968020473,(53:7.192908829,(((60:3.49119345,((61:2.035489541,62:2.0354895414):0.3917716178,63:2.427261159):1.063932291):1.884941298,59:5.376134748):0.4106793747,(57:3.64018038,58:3.64018038):2.146633743):1.406094706):1.539970287):3.390004068,(64:8.866802396,65:8.8668023957):3.256080789):11.359824613999999,((((33:3.400388252,34:3.4003882516000004):3.103850376,35:6.504238628):4.637450798,(((((36:4.2456915058,37:4.2456915064):0.1816457857,38:4.427337292):3.240208964,((39:4.504466493,40:4.504466493):1.425551928,41:5.9300184208):1.737527835):0.4907143398,42:8.1582605958):1.852214595,(43:7.92269012,(45:6.786939682,44:6.7869396822):1.135750438):2.08778507178):1.131214235):10.744495435,((((51:6.963561272,50:6.9635612725):0.06481889303,(49:4.879877466,48:4.879877466):2.148502699):2.916305826,(47:7.77497624,46:7.774976240300001):2.169709752):3.7755005137,52:13.72018650963):8.165998355):1.5965229391000002):11.526072616):3.2008240185,(66:34.42468332,((((72:12.383544356,71:12.383544355999998):2.131263828,73:14.514808180000001):0.7226781999,(((77:2.901852584,75:2.9018525836):1.568517564,74:4.470370148):0.6307069953,76:5.101077143):10.13640924):5.071049969,(70:17.43119828,(69:15.617551450499999,(68:6.049922172,67:6.0499221720000005):9.567629278):1.813646831):2.8773380719):14.1161469666):3.7849211059999996):4.16200099,(((79:2.377728831,80:2.377728831):17.81287903,((((83:14.86592328,(82:13.04664961361,81:13.046649614):1.8192736605):1.161686214,84:16.027609488499998):0.2331112958,85:16.2607207853):1.349048465,(86:9.676348758,87:9.6763487586):7.933420491699999):2.580838609):16.005491161000002,78:36.196099018):6.175506399):1.209998327,(((((((((101:16.36654515,((99:11.64791872,100:11.64791872):1.052421664,((((97:5.407683641,(95:3.292876861,96:3.292876861):2.1148067804):0.4550093022,98:5.862692943400001):4.1823154678,94:10.0450084111):0.05520278254,93:10.1002111946):2.60012919):3.66620477086):0.7778808980047001,102:17.1444260527):8.365569818,((((((105:2.09179972,104:2.09179972):9.591724158,103:11.683523877999999):3.548962673,((((((107:9.5020619499,106:9.50206195):2.876396294,((((109:0.154792042,110:0.154792042):6.149711708200001,108:6.3045037494):1.093658747,(111:4.446123103,112:4.446123103000001):2.952039394):1.823191203,113:9.2213536993):3.157104544):0.9318083758,(115:1.956501063,114:1.9565010634):11.353765557):0.8095882829,(117:5.941176336,116:5.9411763361):8.1786785667):0.6080319233,118:14.7278868259):0.2105390193,(121:8.5916522867,(119:2.299405466,120:2.299405466):6.292246821100001):6.3467735577):0.294060706):0.3360592245,122:15.5685457731):1.422205728,((124:9.1681573513,125:9.1681573522):4.201042703120001,123:13.369200054379998):3.6215514488):0.2148592481,126:17.2056107508):8.304385117899999):3.193841085,127:28.703836948):1.919051321,(128:8.403599309,129:8.4035993088):22.21928896167):5.651304098,((((90:5.064139022,91:5.0641390218):20.570331166000003,89:25.634470194000002):0.538957474,88:26.1734276643):2.907905275,92:29.081332939000003):7.192859431):2.64169302,130:38.915885385799996):2.879741076,(132:33.671721655,131:33.67172166):8.123904813):0.9245903739,((((((143:9.579304396,144:9.579304396):18.15267053,(((((((155:4.1542936371999994,156:4.1542936374):3.065864878,157:7.2201585141):1.751161646,158:8.971320161600001):0.2662685725,(((152:1.944243356,151:1.9442433556):3.756606192,(154:0.309443332,153:0.309443332):5.391406216):2.653790539,(150:5.166095408,149:5.166095408):3.1885446793999996):0.8829486468):2.040908096,159:11.27849683):8.546832039,((145:14.463815979,146:14.463815978899998):4.001631701,((147:15.017715774800001,148:15.017715776000001):1.3505812862,160:16.368297062):2.097150618):1.359881188):3.150203908,161:22.975532773300003):4.756442152):1.592880291,((162:22.239373941,163:22.239373937):4.443304084,((((((164:5.655501936,(166:0.6136250968,165:0.6136250968):5.0418768400000005):1.271009486,(168:2.09541618,167:2.0954161805):4.831095242):1.275429946,(170:2.411639935,169:2.411639935):5.790301433):0.8514261393,171:9.053367508):1.671670225,(173:3.8010394396000002,172:3.8010394401):6.923998291999999):0.1868592482,((((((175:3.638729092,174:3.638729092):0.403751844,176:4.042480936):0.5656733289,177:4.6081542654):0.4479183843,178:5.056072649):1.120849089,179:6.176921739):1.262832661,(180:4.973129653,181:4.973129653):2.466624746):3.4721425802):15.770781040389):2.642177196):2.281997726,((((134:2.636988281,133:2.636988281):1.343920954,135:3.980909235):0.5801610707,136:4.5610703060999995):18.1117417164,(((141:5.576301849,(140:2.869650896,139:2.8696508955):2.706650952):1.902334027,142:7.478635874999999):10.13323023987,(137:4.254989738,138:4.25498973792):13.3568763798):5.0609459097):8.934040914):1.323206956,((186:6.613719117,(185:1.9253059393999998,184:1.9253059397999999):4.6884131777):18.204734835300002,(182:17.1406953752,183:17.140695374):7.6777585793):8.111605946000001):2.073717063,(188:0.7286447477,187:0.7286447477):34.275132214477196):7.716439876):0.8613869092):8.719299186);

TREE tree_1331 = ((18:27.468104550299998,((((1:15.11107541,2:15.111075406):1.515963974,(((4:11.073060123000001,((6:2.442974365,5:2.442974365):5.359963739,((8:5.03722188761,7:5.0372218882):0.4599180481,9:5.4971399356):2.3057981685):3.2701220197999996):1.798499154,10:12.871559278):1.001473998,3:13.8730332757):2.754006104):1.6389646315,(11:6.483214757,12:6.483214757):11.782789255):4.517636759,((((15:4.488450354,14:4.4884503535):2.740797968,13:7.2292483214):0.9280855903,16:8.157333912):0.7285637259,17:8.8858976366):13.897743131899999):4.684463781):19.8693051559,((((((((30:16.518970615,(32:13.800182353,31:13.800182353299999):2.7187882618):1.427657505,29:17.94662812):9.3021573594,((((((24:8.794166867500001,23:8.794166868):5.320245407,(21:5.551578741,22:5.551578741):8.562833534):0.7909856649,(19:5.012146996,20:5.0121469953):9.893250944):1.801629882,(25:11.714908732,26:11.714908732400001):4.9921190894):4.546403844,27:21.2534316654):1.367394274,28:22.620825939):4.627959540599999):1.543322353,((64:8.067533469,65:8.067533469):3.365165311,((54:7.96208005,(55:4.889042217,56:4.88904221679):3.0730378324):0.6196952998,(((((61:3.240032806,62:3.2400328056):1.078870589,63:4.318903395):0.1812219199,60:4.500125315):0.512457556,((57:3.345363905,58:3.345363904):1.414841512,59:4.760205416):0.2523774549):2.320429591,53:7.333012462):1.248762888):2.85092343):17.35940906314):0.26837475,((((((48:5.649818011,49:5.649818010000001):3.429201109,50:9.0790191201):0.3267299544,51:9.405749074300001):3.249395874,(46:9.026158494,47:9.026158493):3.628986454):4.786659608,52:17.44180455587):9.507081335999999,((((((37:5.7889935359999996,36:5.7889935365):0.4872429099,38:6.276236446):2.80307864,((39:5.034340586,40:5.0343405853):2.080232889,41:7.1145734759999995):1.964741611):0.8701847855,42:9.949499871419999):1.207555183,((45:8.6152275342,44:8.615227533):1.482337458,43:10.0975649906):1.0594900642):0.8613594878,((33:3.5742017625,34:3.574201763):3.739134317,35:7.313336079499999):4.705078464):14.93047135):2.1115966969):5.290474775,(66:26.46217336,((((((68:4.259891077,67:4.259891078000001):4.590514496,69:8.850405573):2.52912995,70:11.37953552):1.19952014,((74:0.4474314266,76:0.4474314266):6.3662733320000005,(75:1.324761359,77:1.324761359):5.4889434):5.7653509043):0.6125491991,(71:11.73752684367,72:11.737526844000001):1.454078018):0.4349257122,73:13.626530573):12.835642785700001):7.888784007):5.153146696,(((80:1.613771601,79:1.613771601):15.95262006,(((84:14.555613409,85:14.555613408000001):0.1769949887,((81:10.79630369,82:10.7963036924):1.4883587925000001,83:12.284662484499998):2.447945912):1.191996182,(86:8.818094733,87:8.818094733):7.106509846):1.641787083):14.2747898248,78:31.8411814905):7.662922572):3.087898896,(((((((((101:18.502524297,(((93:11.896181028,94:11.896181027799999):0.00966468875,((97:6.331768987,(95:3.788073052,96:3.7880730513999996):2.543695936):0.5857637277000001,98:6.917532715799999):4.988313001749999):3.520184875,(99:13.62776338,100:13.62776338):1.798267212):3.0764937058):1.1667373207,102:19.669261618):10.5765748821,((((((105:4.102297753,104:4.102297753):10.63349837,103:14.735796125):3.1776836037,((((119:1.875117592,120:1.875117592):7.8578699503000005,121:9.73298754216):7.4994170413,118:17.232404583):0.1865825304,((((106:10.2696459,107:10.269645899499999):4.618045056,(((108:6.2260743849999995,(109:0.2896844358,110:0.2896844358):5.9363899485000005):2.647825796,(111:5.675486821,112:5.6754868223):3.198413359):2.358614206,113:11.2325143862):3.6551765701):0.5356956348,(114:2.505492989,115:2.505492989):12.917893602):0.5819703737,(117:6.596537621,116:6.59653762052):9.408819344000001):1.413630148):0.4944926172):0.4955091993,122:18.4089889332):0.9590403667999999,(123:15.7511660251,(124:8.752972493,125:8.7529724927):6.99819353215):3.6168632709999997):0.54439124223,126:19.912420539):10.333415961899998):1.034429389,127:31.280265889):1.106383691,(129:8.388597848,128:8.3885978482):23.99805173259):3.752746794,((((90:5.61642809,91:5.616428089799999):16.7574826082,89:22.373910701):2.011372739,88:24.385283441300004):4.450801436,92:28.836084878):7.303311502):2.711656091,130:38.85105247690001):0.9404065833,(132:33.141675033,131:33.14167503):6.6497840207):2.127331602,((((((((140:4.79909262,139:4.799092621):0.8908603098,141:5.6899529296):7.1875307159999995,142:12.877483646000002):8.978941666599999,(137:8.569466617,138:8.5694666175):13.286958700000001):5.827044057999999,(((133:1.997018822,134:1.997018822):0.6632948175,135:2.660313639):1.228020352,136:3.8883339909999997):23.795135376300003):1.621565569,(((143:9.821842402,144:9.821842403000002):15.05846314,((((((((156:3.678941348,155:3.678941348):3.907449369,157:7.5863907162):1.599663929,158:9.18605464573):0.2434607819,((149:7.7952705586,150:7.7952705577):0.9239424024,((151:1.754505869,152:1.75450586856):3.167047502,(153:0.518982917,154:0.518982917):4.4025704534):3.79765959):0.7103024675):0.8663337044799999,159:10.295849132):5.893114713,160:16.188963849):0.3150371993,((145:11.250039409,146:11.2500394092):4.351109053,(148:13.8783554951,147:13.87835549524):1.7227929669):0.902852582):2.689359466,161:19.1933605101):5.686945036):1.010881973,((162:16.0544806521,163:16.054480657):8.181863904,(((171:9.728575054,((((165:0.2726792394,166:0.2726792394):5.1910449252,164:5.4637241648999995):2.090571954,(167:1.99872977,168:1.99872977):5.55556635):0.5119190161,(170:1.058774278,169:1.0587742787):7.007440857000001):1.662359919):1.857168672,(173:3.005715773,172:3.005715773):8.580027953):1.213451515,((181:4.081167658,180:4.081167658):4.42084197,((((177:4.940482535,174:4.940482534699999):0.435997355,(175:5.1505438534,176:5.150543853):0.2259360363):0.5292722211,178:5.905752111):2.252986374,179:8.158738485):0.3432711426):4.2971856127):11.4371493191):1.654842965):3.413847416):0.876252119,((183:13.576556837000002,182:13.576556837):7.109027689,((185:3.090056955,184:3.0900569547):4.154024241,186:7.244081195):13.441503329700002):9.495702534):2.505425206,(188:0.08741790797,187:0.08741790797):32.59929435998):9.232078392):0.6732122989):4.7454067514);

TREE tree_3820 = ((((((1:19.01004145,2:19.010041442000002):1.307700082,(((4:13.50535983,(((7:5.9796385999999995,8:5.9796385994):1.6971340325000002,9:7.676772632):1.848116397,(6:3.291065328,5:3.2910653286000002):6.233823701):3.980470802):1.331370446,10:14.836730271999999):1.08547388437,3:15.922204156):4.3955373672):2.2294246568,(11:8.30300316,12:8.30300316):14.244163024):4.643537701,((((15:7.046347025,14:7.046347025):2.335201444,13:9.381548469):0.9414714687,16:10.3230199385):0.8921521822,17:11.215172120199998):15.9755317648):5.5565649458,18:32.7472688298417):22.858289221200003,((((66:24.8039459,((((70:12.44844859,(69:10.7021961805,(67:7.9734554499999994,68:7.9734554499999994):2.7287407310000003):1.746252405):2.220252546,(((76:0.3071658271,75:0.3071658271):3.749669242,77:4.056835069):5.647903016,74:9.704738085):4.963963046):0.1500775127,73:14.818778639000001):0.5909626059,(71:12.275626096,72:12.275626096):3.134115154):9.3942046507):8.17510355,(((((((19:2.735900199,20:2.7359001989):5.604770482,((24:4.864627833,23:4.8646278335):3.1345078231,(21:3.6461867,22:3.6461867000000003):4.3529489562000006):0.3415350243):1.913242211,(25:6.150457734300001,26:6.150457735500001):4.1034551574):2.681420011,27:12.9353329031):0.9948074468,28:13.930140349999999):5.1360525499,((30:10.291650385,(31:8.661786832699999,32:8.661786833):1.629863552):1.7983198096,29:12.089970194091999):6.976222705):11.68852542,(((64:10.71018011,65:10.7101801169):3.000938855,(((((57:4.258320803,58:4.258320803):1.741212712,59:5.9995335143):0.6612268476,(63:5.307334633,(60:4.178733126,(61:2.229764874,62:2.2297648741):1.948968252):1.128601507):1.35342573):3.331725489,(56:4.7949017425000005,55:4.7949017430000005):5.197584108999999):0.6292964765,(54:9.260664672,53:9.260664672):1.361117657):3.089336639):10.800417313099999,((52:15.03184190347,(((50:7.8780958429,51:7.8780958420000005):0.7597096008,(49:4.908445713,48:4.908445712):3.7293597301999997):2.312324804,(46:8.695911713,47:8.6959117131):2.254218535):4.0817116586):8.063189348,((((44:6.917647141,45:6.9176471412):1.956098052,43:8.873745193):1.9573947375,((((39:4.876626389,40:4.876626389):1.680589933,41:6.5572163215999995):2.453901664,((36:5.733468608,37:5.733468608000001):0.4673375134,38:6.200806121399999):2.810311864):0.7409667627,42:9.752084748):1.0790551821999999):0.6415224325,((33:4.094893702,34:4.094893702):2.501679299,35:6.5965730006):4.876089362):11.6223688881):1.4165050259):6.2431820377):2.2243311290000003):12.51952492,(78:40.105528074,((79:3.034992711,80:3.034992711):18.47143231,(((((81:12.035259319,82:12.0352593183):0.7975989451,83:12.832858259):3.438560395,85:16.271418658199998):0.2762270763,84:16.547645735):0.7439031323,(86:10.52302466,87:10.52302466):6.76852421):4.214876156):18.599103051):5.393046296):4.477543567,(((((((((101:20.3529752098,((99:12.81228454,100:12.81228454):2.827697101,(((((95:5.040322273,96:5.040322272):1.324291064,97:6.364613337):1.7049894775999999,98:8.069602816):3.5653998199999997,94:11.635002634):0.1345578374,93:11.769560472190001):3.870421166):4.71299357):0.1991405223,102:20.5521157285):6.113045841,((((((105:1.727798404,104:1.727798404):10.10598187,103:11.833780276999999):4.7119000628,((((((106:9.094255852,107:9.0942558519):3.251703642,(((108:6.387448996,(109:0.3408952188,110:0.3408952188):6.0465537772):1.132600357,(111:4.025859969,112:4.025859969):3.494189384):1.865477697,113:9.38552705048):2.9604324437):1.134171256,(115:2.950395299,114:2.950395299):10.529735451):0.6772496685,(116:6.541171562,117:6.5411715619):7.616208856):0.6725924421,118:14.82997286):1.068077925,(121:9.5452164591,(120:2.390350106,119:2.390350106):7.1548663535):6.352834326):0.6476295529):0.5212927453,122:17.0669730821):1.332223261,(123:14.34618859142,(125:7.532489286999999,124:7.532489286):6.81369930505):4.0530077532):0.9052997771,126:19.304496121):7.360665449):2.515957807,127:29.18111936833):1.629279538,(128:8.696804635,129:8.6968046358):22.1135942748):12.2543027,(((89:27.23891495,(90:7.615901448700001,91:7.615901449):19.623013498000002):5.834709755,88:33.0736247043):2.949974662,92:36.023599362):7.041102247):2.768617037,130:45.8333186515):2.898577507,(131:30.6488808,132:30.64888079):18.083015357):1.052188161,((((136:7.333779075000001,(135:3.279995532,(133:2.38268033,134:2.38268033):0.8973152022):4.053783544):20.4740457933,(((141:8.449802926,(140:4.257221004,139:4.2572210045):4.1925819220000005):5.921812689,142:14.371615615):9.429103847,(137:6.48762523,138:6.4876252308):17.3130942278):4.00710540757):9.75093056,((((143:8.442449611,144:8.442449611):20.10288027,(((((((156:4.113433874,155:4.113433874):3.309045212,157:7.422479086):1.824495754,((150:5.647326012,149:5.647326012):2.681402851,((153:0.5387853295,154:0.5387853294):6.046988772,(152:2.6610045847999997,151:2.6610045843):3.924769517):1.742954762):0.9182459776):0.5201960336,158:9.7671708741):2.346221646,159:12.113392519):7.399896546,((160:18.606368367,(148:17.27349579322,147:17.27349579):1.3328725767599998):0.4642734957,(146:12.77373051,145:12.773730505):6.296911354):0.4426472033):2.631361133,161:22.144650196):6.400679676999999):2.067088964,((162:16.904266497280002,163:16.904266489999998):5.994362425,(((((((166:0.2900974849,165:0.2900974849):7.067168982,164:7.3572664670000005):0.4074330748,(168:2.674476733,167:2.674476733):5.090222809):1.624216041,(169:2.326372723,170:2.3263727228):7.062542859):0.6563220985,171:10.045237681):2.964219631,(172:3.393498439,173:3.393498439):9.615958873):0.1469319498,((181:5.684262844,180:5.684262844):3.433988608,((178:6.268962099,(((174:3.831702102,175:3.831702102):1.319954065,176:5.151656167):0.7610750034,177:5.9127311702):0.35623092893999997):1.093426235,179:7.362388334):1.755863118):4.0381378103):9.7422396590175):7.713789911):6.125387714,((183:17.77385031,182:17.773850313):11.60992685,((184:1.523988853,185:1.5239888526):7.250256706549999,186:8.774245559):20.6095316054):7.3540293830655):0.8209488812):3.51284929,(188:0.2952553112,187:0.2952553112):40.7763494139862):8.712479594000001):0.192033617):5.629440116);

TREE tree_1636 = ((18:32.9219902997,(((11:11.21598598,12:11.21598598):12.259944499,((1:21.8871994,2:21.887199407):0.08458145431,(((4:14.515797977,((5:3.677051909,6:3.677051909):6.591652939,((8:6.5108858758,7:6.510885875):0.8120852909,9:7.322971166):2.9457336826000002):4.2470931279999995):2.685443725,10:17.201241698):2.1024374378,3:19.303679143):2.668101717):1.5041496233):4.91205519,(((13:9.050420840000001,(14:4.405099636,15:4.405099636):4.6453212063):0.7335930488,16:9.784013889999999):0.768172671,17:10.5521865618):17.8357991069):4.5340046302):26.946252886000003,(((((((((((49:8.81613483,48:8.81613483):4.116060954,51:12.932195784):0.5056180263,50:13.43781381):6.484966366,(47:14.36575998,46:14.365759979):5.557020197):5.295962295000001,52:25.2187424731):11.615479804,(((33:7.280507806,34:7.280507806):3.513438611,35:10.793946417):8.133792342,((43:13.7979824,(45:10.4173052156,44:10.417305217):3.380677189):1.978215983,((((37:8.8067833391,36:8.8067833389):2.202705155,38:11.009488493500001):2.570590104,(41:9.085299061,(39:7.180854668,40:7.180854668):1.904444393):4.494779537):1.295740947,42:14.8758195439):0.9003788439):3.1515403706000003):17.906483521000002):2.9444987654,(((64:11.7308563,65:11.7308562982):2.788434006,((53:11.33033971,(54:10.53182068,(56:5.412855508500001,55:5.412855509):5.11896517):0.7985190314):0.09939146938,(((57:4.956534527,58:4.956534527600001):2.597364894,59:7.553899422000001):1.606476364,((60:7.26389685,(61:3.725787996,62:3.7257879959999998):3.538108854):0.2354047137,63:7.499301563):1.661074222):2.269355395):3.089559122):21.8272208764,((((((24:10.89354792,23:10.893547921):7.315961732,(21:6.944548653,22:6.944548653):11.264960999):0.3770051145,(19:3.953117149,20:3.9531171485):14.63339762):2.266136024,(26:10.75150446,25:10.7515044611):10.101146330399999):6.369681831,27:27.222332623):1.420365028,28:28.642697651):7.70381353):3.432209855):0.2533086054,((30:22.123503936,(32:18.447869437,31:18.4478694375):3.6756344999):4.2283652753,29:26.351869214):13.680160427):7.9594005789999995,(66:39.21352354,(((71:18.018233463,72:18.018233463):0.7017491324,(((69:12.706279583999999,(68:10.31445581,67:10.314455814):2.3918237711000003):2.766523158,70:15.47280274):2.575945427,((75:7.517998004,74:7.517998004):4.51723246,(77:6.004293624,76:6.004293624):6.0309368403):6.013517706):0.67123442554):1.084999524,73:19.804982124):19.40854142):8.77790668):5.544519039,(((80:4.496503401,79:4.496503401):23.94725516,(((85:20.76390186,84:20.763901855):0.4905049087,(83:19.2177764261,(82:16.904945472,81:16.904945475):2.3128309582999997):2.036630334):2.619064191,(86:9.113169017,87:9.113169017):14.760301937):4.570287605):20.9360887066,78:49.379847276):4.1561019902):1.013263692,((((((((102:25.5066256194,(101:23.55561023,(100:20.52244712,(99:19.83701151,((93:13.768998229000001,((97:7.675307412,(95:4.756567017,96:4.756567017):2.918740395):2.755387406,98:10.430694818):3.338303413):0.1660719918,94:13.935070222):5.901941286):0.6854356137):3.0331631072):1.95101539373):15.102906477,(126:30.877059287,(((((((((107:14.4008476795,106:14.400847683):5.844154195,(((111:7.865060991,112:7.8650609901):4.651227631,(108:10.47206569,(109:0.4117838449,110:0.4117838449):10.0602818475):2.04422293):2.628344167,113:15.144632789150002):5.100369085400001):2.257944908,(115:4.002698568,114:4.002698568):18.500248215):1.246803267,(117:10.682496549100001,116:10.682496549):13.0672535):0.9447642998,118:24.694514348):1.007024552,((119:2.960264028,120:2.96026402803):10.2884074422,121:13.2486714704):12.452867430400001):1.3009433599,((105:4.813354628,104:4.813354628):15.56274348,103:20.3760981028):6.626384155):0.4991222597,122:27.501604524):2.236983889,((125:13.4536267994,124:13.4536267998):11.703502644030001,123:25.157129441400002):4.5814589666):1.1384708711):9.73247282):2.908678211,127:43.518210309):0.5152794237,(129:13.2354833148,128:13.2354833138):30.7980064154):5.334561374,(((89:33.818341099,(90:12.37839287,91:12.378392872000001):21.4399482326):5.251737574,88:39.0700786739):4.292135568,92:43.362214243):6.005836864):1.23390145,130:50.601952564):0.8593825656,(131:36.62155904,132:36.621559045):14.839776073):0.6893149824,((((((143:9.687636567,144:9.687636567):21.05308938,(((((((156:5.55826453,155:5.55826453):4.394944494,157:9.953209023):0.8227969028,158:10.776005925700002):0.6504170765,(((154:1.213036533,153:1.21303653304):6.667260856,(152:2.6049972342,151:2.6049972342):5.2753001551):2.427872503,(150:6.975266615,149:6.975266615):3.3329032774000003):1.118253111):4.011650952,159:15.4380739578):8.036852851,(((145:15.2370289802,146:15.237028976799998):5.591987864,160:20.829016836999998):2.148271577,(147:19.227790458599998,148:19.227790458599998):3.7494979600400002):0.4976383872):1.436167712,161:24.911094518):5.829631430999999):1.172556991,((162:25.046136424,163:25.04613642119):2.584430079,(((171:12.87853355,(((167:3.282733631,168:3.282733631):5.932610026,(164:5.95924305,(165:0.1360360559,166:0.1360360559):5.8232069939999995):3.2561006068):1.150593246,(169:4.732453999,170:4.7324539986):5.6334829039):2.512596645):2.159070978,(173:3.276624468,172:3.276624468):11.760980057):0.361189896,((179:9.858073459,(((176:5.817671438,(174:4.841597499,175:4.8415975):0.9760739383570001):0.1860298283,177:6.0037012662):0.9765600179,178:6.980261284000001):2.8778121751):1.279531109,(181:4.467930799,180:4.467930799):6.669673768999999):4.261189854):12.2317720782443):4.282716448):2.283608684,((((141:6.01982853583,(139:4.6334169082,140:4.6334169083):1.3864116275):6.630667147,142:12.650495681999999):6.863831831200001,(137:7.042306605499999,138:7.0423066064):12.472020906900001):4.5533184193,((135:4.119430954,(133:3.332296611,134:3.332296611):0.7871343428):1.444081022,136:5.563511976):18.50413396):10.1292457):2.002923552,((183:15.79637203,182:15.796372027):7.271207274,(186:7.069859331,(184:2.3033319633000002,185:2.3033319627999997):4.7665273692):15.997719970000002):13.132235877):4.448659241,(188:0.02091701702,187:0.02091701702):40.627557402758):11.502175683699999):2.398562847):5.319030232999999);

TREE tree_0323 = ((18:31.260783320199995,(((11:10.02463959,12:10.02463959):11.896840942,((1:16.78721677,2:16.787216774):1.225109901,(3:15.940718899,((4:11.929551474,(((8:6.841382824369999,7:6.8413828243):0.9334857333,9:7.77486855801):1.56651118,(6:3.571644247,5:3.571644247):5.7697354907):2.5881717361):3.206775416,10:15.136326894):0.80439200995):2.0716077772):3.909153851):4.186279438,(((15:5.863072154,14:5.863072154):3.887658336,13:9.750730489):1.195246203,(16:9.9939496778,17:9.993949676800002):0.9520270154999999):15.1617832708):5.1530233545):15.8544071561,((((66:24.74209555,(((((77:3.816127664,(75:0.469398127,76:0.469398127):3.346729537):3.718710264,74:7.534837928):5.02000362,(((68:3.858495683,67:3.858495683):5.6092433239999995,69:9.467739007):2.011993938,70:11.47973294):1.07510860231):1.057080584,(71:9.054506142000001,72:9.054506142000001):4.557415988):3.159632684,73:16.771554818):7.9705407353):11.282922473,((((((((23:7.209425614,24:7.2094256133000005):4.568048988,(21:4.929341969,22:4.929341967999999):6.848132633500001):1.335729502,(20:4.264660841,19:4.2646608406999995):8.848543264):1.963557867,(25:8.673386228,26:8.673386228):6.403375743350001):6.896622843,27:21.973384812000003):0.6152183889,28:22.588603202999998):7.9421451561,((30:15.551962197999998,(32:13.997447704999999,31:13.997447703299999):1.5545144931):3.596967372,29:19.1489295704):11.3818187899):2.030176339,((((((((36:5.2101148504,37:5.2101148509):0.3930108498,38:5.6031257007):2.243911263,((39:3.962884194,40:3.962884194):1.279090576,41:5.241974770000001):2.605062194):0.8407085919,42:8.687745555):0.9523407264,((44:6.469936532,45:6.469936531):1.714724923,43:8.184661455):1.4554248277000001):0.3009850739,((34:4.017769499,33:4.017769499):2.149248505,35:6.167018004):3.774053351):11.351381787,((((49:4.599499398,48:4.599499398):3.54653858,(51:7.822718273,50:7.8227182732000005):0.3233197047):2.835870999,(46:7.845307461,47:7.845307460499999):3.1366015162):2.4367841754,52:13.4186931519):7.873759989):2.803389458,((64:9.052296297,65:9.052296297):3.334658333,((((((61:3.453906085,62:3.453906085):0.6806225582,60:4.134528643):1.478022831,((57:3.27969727,58:3.2796972693):1.825231173,59:5.104928442):0.507623032):0.06702111042,63:5.679572585):2.034335686,53:7.713908271):2.075243176,(54:8.988422773,(55:4.984249905,56:4.9842499046):4.0041728689):0.800728673):2.597803184):11.70888797):8.4650821):3.4640933289999998):2.184638109,(78:34.508923962,((((85:12.97218745666,84:12.972187457):0.658376522,(83:11.207891357000001,(81:9.634609893,82:9.6346098926):1.5732814639100001):2.422672622):0.3268324457,(86:6.598469752,87:6.598469752):7.358926671800001):3.57571614,(80:2.057681229,79:2.057681229):15.47543134):16.975811395):3.70073216752):2.959280232,((((((((90:5.705586729,91:5.705586729):17.2727896508,89:22.978376378):0.3713511257,88:23.3497275037):4.68726206,92:28.036989561000002):5.917254278,(((((101:18.5518819215,((99:14.18323029,((93:10.51930941642,94:10.5193094163):0.05679054604,(((95:4.514728031,96:4.514728031000001):2.600495904,97:7.1152239345):0.4056369334,98:7.5208608681):3.0552390947):3.607130326):1.033954546,100:15.21718483):3.334697091):0.1733526846,102:18.725234609900003):7.3900249301,(126:19.381594429,((123:14.4621894927,(125:8.415772286973,124:8.4157722868):6.0464172045):4.323233674,((((((((106:9.028937758,107:9.028937759099998):3.053360313,(((108:6.6087133204,(109:0.3345703888,110:0.3345703888):6.2741429315):1.52121565,(111:5.079442609,112:5.0794426092):3.050486361):0.4315558759,113:8.5614848466):3.5208132244000003):0.4416069489,(114:2.975588992,115:2.975588992):9.5483160283):1.065489961,(117:6.67448723,116:6.6744872299):6.9149077514999995):1.673897251,118:15.263292232000001):1.099814679,((120:1.200611324,119:1.200611324):6.6153529761,121:7.815964298999999):8.5471426113):0.45923650190000004,((105:2.684263049,104:2.684263049):10.66035846,103:13.344621507):3.477721905):0.5458537034,122:17.368197117199998):1.4172260495):0.5961712634):6.733665110914999):1.345769392,127:27.461028945):0.7583978342,(128:7.538829016,129:7.5388290162):20.6805977537):5.734817072):4.027770973,130:37.982014823):0.6902903924,(132:29.001583088,131:29.001583088):9.670722123):2.02633732,((((((143:8.9406143,144:8.940614301):12.52528819,(((((((156:2.992792866,155:2.992792866):3.454964458,157:6.447757324):0.574000272,158:7.02175759638):0.3114173787,((150:5.882229208,149:5.882229207):0.4912172104,((153:0.9046878092,154:0.9046878092):4.953447808,(152:1.4437883177000002,151:1.4437883176000001):4.414347299):0.5153108011):0.9597285567):2.473967246,159:9.80714222):5.136022175,(((146:10.8611408262,145:10.86114082437):1.53917635,160:12.400317177000002):1.622929194,(147:11.1728007552,148:11.1728007565):2.8504456143):0.9199180257):1.944742216,161:16.887906611):4.577995875):1.517999504,((162:19.673008131000003,163:19.673008137):1.458161507,(((171:5.948396066,((((166:0.2105120984,165:0.2105120984):3.2652265397999995,164:3.475738638):1.149591081,(168:1.779368956,167:1.779368956):2.845960763):0.9253442376,(170:0.93960988,169:0.93960988):4.6110640773):0.3977221094):1.55885166,(172:2.480960707,173:2.480960707):5.026287019):1.391255639,(((((174:3.82906957,(175:3.236358339,176:3.236358339):0.5927112305):0.3148491781,177:4.14391874736):0.1832292113,178:4.32714795891):0.7162679042,179:5.043415863):1.020085158,(181:2.842040627,180:2.8420406270000003):3.221460394):2.8350023434000002):12.23266627713861):1.852732351):2.193747811,(((137:3.5453210853,138:3.545321086):13.8127089972,(((140:3.992146732,139:3.9921467325999997):2.6960821446,141:6.688228877):5.705175519,142:12.393404395000001):4.964625685550001):1.03563299183,((135:2.745685207,(133:1.431172895,134:1.431172895):1.314512313):0.5822687828,136:3.3279539901999997):15.065709083000002):6.783986726):1.944426078,((182:13.945148959800001,183:13.945148964):2.859744675,(186:5.108209275,(185:1.208177007,184:1.2081770065):3.900032269):11.696684358799999):10.317182245000001):2.527609675,(187:0.5715841545,188:0.5715841545):29.078101407653698):11.048956972100001):0.4702938389):5.9462541062);

TREE tree_6050 = (((((11:11.28886739,12:11.28886739):11.578782998,((1:18.60284091,2:18.602840918):1.117244383,(((4:12.994762147,(((7:5.6471223051,8:5.6471223052):1.36858882476,9:7.01571113081):1.69019208,(5:2.2546095086999998,6:2.2546095079999997):6.451293702):4.2888589370000005):2.135930917,10:15.130693064999999):1.8731763559999999,3:17.00386942):2.716215877):3.147565095):4.89369248,((16:10.87264406,((14:4.451090196,15:4.451090196):5.625344478,13:10.076434673):0.7962093868):0.1709869849,17:11.043631044900001):16.7177118258):3.86105928783,18:31.6224021670445):22.297564895,(((((((((((19:5.676610122,20:5.6766101225):9.663151636,((21:5.265251636,22:5.265251635):9.828012386000001,(24:7.8546135838,23:7.854613584):7.2386504359999995):0.2464977373):2.109265681,(25:10.918081903,26:10.918081903000001):6.530945537399999):6.108669747,27:23.557697186000002):1.572923447,28:25.130620633999996):6.093394196,((52:18.7052281524,(((51:9.486285261999999,50:9.486285261999999):0.4022308236,(49:5.908904518,48:5.908904518):3.979611568):4.886506519579999,(47:9.549790224,46:9.549790224999999):5.225232382):3.930205547):11.640433627,(((34:4.59847381,33:4.59847381):3.29093514,35:7.889408951):7.515515423,(((44:9.625106343,45:9.625106342):3.253577824,43:12.87868417):1.108967626,((((39:3.609149282,40:3.609149282):4.656761828,41:8.265911111000001):2.261690895,((36:6.184619536,37:6.184619537):1.260924169,38:7.4455437060000005):3.0820583):0.4808272716,42:11.008429277):2.979222515):1.41727258):14.9407374051):0.8783530498483):3.815033387,((30:16.358837893,(32:14.466814017810002,31:14.466814017499999):1.8920238726):2.458033572,29:18.81687146008):16.222176746000002):3.222624433,((65:11.671930939149998,64:11.671930941):6.2636045,(((((63:6.400677233,(60:5.943326696,(61:4.414213062,62:4.4142130625):1.529113634):0.4573505364):0.8539972986,59:7.2546745314):1.01067615,(57:6.578406341,58:6.578406341):1.68694434):1.469156766,53:9.734507448):4.132993097,((55:7.247600855,56:7.2476008541):5.2197739259,54:12.46737478):1.400125764):4.068034897):20.3261372057):1.8277605324,(66:34.53775468,((((72:10.4186031728,71:10.418603172200001):1.589953788,(74:9.160904551,((75:1.836688913,77:1.836688913):2.482625405,76:4.3193143182):4.841590232):2.8476524101000003):0.3854613642,73:12.394018325000001):2.082154848,((69:9.71029775,(67:4.222515671,68:4.222515671):5.4877820795000005):1.774210697,70:11.48450845):2.9916647259999998):20.061581506):5.551678503000001):3.92528833,(((80:3.57647133,79:3.57647133):19.42340585,((85:17.302745934999997,(84:15.083549386000001,(83:11.57174223,(81:9.367584854,82:9.367584854999999):2.2041573712):3.5118071595):2.2191965497):0.7543342524,(87:7.915547757,86:7.915547757):10.1415324301):4.942796987):18.669442662999998,78:41.6693198444):2.3454016725):1.942830217,(((((((((101:17.58425504,((100:12.130797,99:12.130797):2.333879073,((((97:5.708558527,(95:2.596002727,96:2.5960027263):3.1125558001):1.170022651,98:6.878581177499999):3.4860641753399997,94:10.364645352):0.31638865116000003,93:10.68103400391):3.783642072):3.1195789689):1.0239915054,102:18.60824654863):9.71851717,((((((((((107:8.585573371330002,106:8.585573370999999):3.571261897,(((108:6.1399565178,(109:0.1467516016,110:0.1467516016):5.9932049164):1.324078875,(111:4.623571582,112:4.623571582):2.840463811):1.788867095,113:9.2529024884):2.9039327793):0.5661510464,(115:2.32169802,114:2.32169802):10.4012882937):0.9844516248,(117:5.3627195904,116:5.3627195902):8.344718348):0.6823078578,118:14.389745797):0.5980796422,((119:1.089293498,120:1.089293498):6.4140123327,121:7.5033058305):7.4845196089999995):0.7070486847999999,((105:3.351239913,104:3.351239913):9.898991303,103:13.250231216500001):2.4446429077):0.4845732939,122:16.17944742):1.544378448,(123:14.0347115199,(125:7.843271670699999,124:7.843271670289999):6.191439850169999):3.689114345):0.8882489726,126:18.612074838999998):9.714688881999999):2.787085583,127:31.113849306):0.6575826927,(129:9.5200493078,128:9.5200493075):22.25138268197):7.430233126,((((91:7.230506318,90:7.2305063191):15.610628002999999,89:22.841134317999998):8.403425875,88:31.2445601959):3.05924134,92:34.303801540399995):4.897863582):1.027985421,130:40.229650539000005):4.778717279,(131:27.234682040000003,132:27.234682036000002):17.773685787):0.5695662755,((((((135:2.85203492,(133:1.322275133,134:1.322275133):1.529759788):0.839939447,136:3.6919743671):12.343596756,(((141:3.9447387320000002,(140:2.4499575949,139:2.4499575941):1.4947811368000001):3.957510346,142:7.9022490781000005):6.5044718029999995,(137:3.6432218314,138:3.6432218314000004):10.7634990498):1.6288502412499999):13.62602946,((182:15.4324023872,183:15.432402388):3.769819149,(186:5.758936477,(185:1.719150931,184:1.7191509313):4.0397855464):13.4432850583):10.459379043):0.0002638735222,(((143:6.877989863,144:6.877989863):15.85655446,(((((((151:1.4628823865,152:1.4628823865):2.688685051,(154:0.9826842888,153:0.9826842888):3.168883148):3.430487688,(150:6.484736218,149:6.484736217):1.0973189073):0.6125127417,(((156:3.890375739,155:3.89037574):2.380701254,157:6.271076993):1.48734882,158:7.75842581301):0.4361420534):1.679787193,159:9.874355059):5.427621268,((145:10.066241437999999,146:10.066241437750001):4.814842715,(160:13.5089490865,(147:12.7477858911,148:12.747785891000001):0.7611631954):1.3721350662):0.4208921749):3.129790598,161:18.4317669269):4.302777391999999):0.4598954415,((162:12.4932225912,163:12.493222584000002):9.040665112,(((171:10.09011231,(((164:6.25167251,(166:0.1771868016,165:0.1771868016):6.074485708999999):2.41504067,(168:3.5502147,167:3.5502147):5.1164984801):0.2577924987,(170:3.034860456,169:3.034860456):5.889645222):1.165606635):3.416016503,(172:5.070273362,173:5.070273362):8.435855454):0.1949531598,((179:8.210380115,((((174:4.114578972,175:4.114578972):2.222503765,177:6.337082736):0.1464293911,176:6.483512127):0.1561431627,178:6.63965529):1.570724825):2.521109713,(180:6.007910896,181:6.007910896):4.7235789318):2.969592149):7.8328057268):1.66055206):6.46742469):8.7289923,(188:0.1777760088,187:0.1777760088):38.2130807439):7.1870773418):0.3796176325):7.962415329);

TREE tree_1876 = ((18:33.3584709330988,((((1:20.24629942,2:20.246299422):0.9759080848,(((4:14.2163525,((6:2.487124632,5:2.4871246323):8.115087469,((8:6.28213037317,7:6.282130372999999):1.587419119,9:7.869549492000001):2.7326626093):3.61414039776):1.973984214,10:16.190336714):0.8999512433,3:17.090287953):4.13191955):1.6341907243,(11:10.31989657,12:10.31989657):12.536501658999999):4.743348027,((((15:6.269426246,14:6.269426245):1.89996779,13:8.169394035):2.541491997,16:10.7108860299):0.5096879703,17:11.2205740017):16.3791722563):5.758724671):26.730013403900003,((((((((((((24:8.240310322600001,23:8.240310322000001):6.12681893,(21:5.17174988,22:5.17174988):9.1953793728):0.6729696489,(19:3.356455905,20:3.3564559054):11.683643):1.428502301,(25:10.152348139,26:10.152348138999999):6.3162530638):3.684380045,27:20.1529812508):0.8461061422,28:20.999087389999996):6.912519500499999,((30:15.163778919,(32:12.935383792,31:12.9353837931):2.2283951276000002):2.049572028,29:17.213350947800002):10.69825594245):5.193501834,(((((45:8.804104648000001,44:8.804104647):2.446637478,43:11.25074213):1.75405779,((((37:6.313219671000001,36:6.313219670700001):1.093378195,38:7.406597866):3.033349153,((40:3.763596729,39:3.7635967288):4.00126675,41:7.7648634795):2.675083539):0.7900136846,42:11.229960703700002):1.7748392125999999):1.358081324,((33:3.406524944,34:3.406524944):3.988176113,35:7.394701057):6.968180182):13.691917416,(((47:9.805058601,46:9.805058599999999):4.785588152,(((49:6.652886903000001,48:6.652886903):3.051521153,50:9.7044080556):1.184891031,51:10.889299086):3.701347667):5.137880279,52:19.7285270323):8.326271626):5.0503100632):0.2304652895,((((54:9.471612563,(55:5.754848037,56:5.754848037):3.7167645269999996):1.328707628,(((61:4.667343961,62:4.667343961):1.915271205,(63:5.915300932,60:5.915300932):0.6673142339):1.119481011,((57:5.954735743,58:5.9547357430000005):1.11404722,59:7.068782963):0.633313213):3.098224015):0.5952796386,53:11.39559983):2.625219126,(64:11.46360021,65:11.4636002136):2.557218746):19.314755059):4.293724987,(66:32.89554073,((((70:11.68018884,((68:4.87103741642,67:4.8710374173):4.860357249,69:9.731394666):1.948794174):4.514933858,((75:2.672331978,(74:0.4851107399,76:0.4851107399):2.1872212381):9.58259925,77:12.254931227):3.9401914717000004):0.1347574784,(71:11.9162115389,72:11.916211539):4.4136686383):3.364163649,73:19.694043827):13.20149690283):4.733758268):15.25502118,(((79:2.436071254,80:2.436071254):21.5533749,((((83:14.339763075999999,(81:12.796296836,82:12.796296836000002):1.5434662443):4.36820007,85:18.707963149599998):0.3799220343,84:19.087885188699996):1.7441238538000001,(86:12.186494681,87:12.186494686):8.645514353):3.157437117):21.775436844,78:45.764882998):7.1194371830000005):1.691697369,(((((((((((99:17.05133677,100:17.051336771):0.6414781785,((93:13.728029323000001,((97:8.876864817,(95:6.114332346,96:6.114332346):2.762532470844):1.843406632,98:10.72027144883):3.0077578737):0.03611497603,94:13.764144301):3.9286706469400006):5.058486865,101:22.751301807999997):3.066352403,102:25.817654213599997):9.343708846999998,((((((((((106:11.51903717,107:11.519037165199999):4.743236194,(((111:4.817928121,112:4.8179281216):5.633328466,(108:8.205882787,(109:0.107732298,110:0.107732298):8.0981504881):2.245373801):0.5537193658,113:11.0049759534):5.257297406):0.5749209355,(114:2.870593059,115:2.870593059):13.966601235600002):1.160455025,(117:7.007930933399999,116:7.007930933):10.9897183871):0.5991712428,(121:10.951765573,(120:1.904107516,119:1.904107516):9.0476580552):7.6450549902):0.7900447238,118:19.386865287):0.8897877236,122:20.276653011599997):0.7492523467,((104:3.281537083,105:3.281537083):11.07880307,103:14.360340157):6.6655652000000005):0.9851418887,((125:10.7035137964,124:10.7035137955):5.600942481400001,123:16.3044562769):5.706590967):1.4154409397999999,126:23.426488186):11.734874875):0.4957194668,127:35.657082534):0.361897203,(128:10.423573530899999,129:10.423573531999999):25.595406200569997):11.51724468,((((91:8.386564273000001,90:8.3865642729):27.27222606,89:35.658790327):2.101734353,88:37.76052468339):5.288176738,92:43.048701418):4.487522992):5.373697923,130:52.909922338200005):0.4239304074,(132:35.146382423,131:35.146382412):18.187470337):0.3041706228,((((((135:3.500099673,(133:2.27122001,134:2.27122001):1.228879662):1.353254446,136:4.8533541190000005):17.128381112,(((141:7.626567012,(140:1.777210905,139:1.7772109048):5.8493561074):2.065335131,142:9.6919021425):9.868448874,(138:5.908422797,137:5.908422796):13.651928220270001):2.42138421745):10.30957562,(((143:8.350388888,144:8.350388888):18.18017891,((((145:15.486128173,146:15.4861281726):2.901813264,(160:18.089611426,(147:16.436185148790003,148:16.436185149):1.6534262781):0.2983300091):0.3906710598,(((((153:0.9017656662,154:0.9017656662):5.236390994,(151:1.344805785,152:1.3448057850000001):4.79335087496):2.33891525,(149:6.923030022,150:6.923030022):1.5540418882):1.388539568,(((155:4.392508762,156:4.3925087613):2.325892208,157:6.7184009689):2.640114875,158:9.358515844800001):0.5070956325):2.18501623,159:12.050627707699999):6.727984788):1.821200134,161:20.599812629):5.930755169):0.8058683711,((162:20.222141058,163:20.222141053999998):1.729466042,(((172:4.717988527,173:4.717988527):12.118027083000001,((((164:8.279908044,(165:0.4258617377,166:0.4258617377):7.854046306):1.098125123,(167:2.940365007,168:2.940365007):6.437668159999999):2.81987148,(169:3.5397614712,170:3.53976147):8.658143176):0.5796653517,171:12.77757):4.05844561):0.1227092191,(((((174:7.008916269,177:7.008916269):0.5292920892,(175:6.544037545,176:6.544037545):0.9941708132):1.114707316,178:8.652915673999999):0.732195526,179:9.3851112):2.625852896,(180:7.835835449,181:7.835835449):4.175128647):4.947760733):4.9928822681):5.384829072):4.95487468):1.439164459,((183:16.238625695,182:16.2386256934):7.298740068,((184:1.768574542,185:1.768574541895):5.914514505,186:7.683089047):15.85427671538):10.193109547):9.443837164,(187:0.4233453251,188:0.4233453251):42.75096715953):10.463710892):0.9379941756):5.512466787999999);

TREE tree_2653 = (((((11:6.241330764,12:6.241330764):9.292067696,((1:13.91733881,2:13.917338812):0.41150048,(((4:9.092999119,((6:1.326435581,5:1.3264355807000001):5.2702500829,((7:3.8897054016,8:3.8897054006999996):0.8882423417199999,9:4.7779477435):1.8187379209999999):2.4963134545):2.227229771,10:11.32022889):0.5984733337,3:11.918702223):2.4101370679):1.2045591688):3.723237211,((13:6.3493200157,(15:3.787218972,14:3.7872189718000002):2.56210104437):0.8298233351,(16:6.353568432,17:6.3535684324999995):0.8255749189):12.077492320900001):5.9144616986,18:25.1710973665):26.0609043686,((((((29:17.7189554451,(30:15.462763892999998,(31:14.055364624100001,32:14.055364624):1.4073992684):2.2561915525):9.055603544,((((((21:5.391839653,22:5.391839653):5.981916627500001,(24:7.0143583901,23:7.014358389):4.359397891):1.470186432,(19:4.618792539,20:4.61879253942):8.225150174):1.66595947,(25:9.8685126173,26:9.8685126183):4.641389566):5.80567995,27:20.315582134814):1.602902268,28:21.918484402):4.8560745883):6.79643184,(((((46:8.93602701246,47:8.9360270127):2.301327745,((51:7.5095157271,50:7.5095157271):0.3952956574,(48:5.541204952,49:5.541204952):2.363606432):3.332543373):4.419309839,52:15.656664596999999):8.719349316999999,(((34:4.14495735,33:4.14495735):3.552040765,35:7.6969981149999995):3.64700453,(((45:7.183439836100001,44:7.183439836):2.8306958929999997,43:10.0141357288):0.9034871966,((((40:4.712002474,39:4.712002474):2.870298136,41:7.582300609000001):1.498343085,((36:5.6823396906,37:5.6823396911):1.012534805,38:6.6948744961):2.385769199):0.5588068581,42:9.6394505537):1.278172372):0.4263797197):13.0320112647):1.7267565361,(((54:13.22985797,(55:7.581165049,56:7.581165049):5.648692915450001):0.9760701706,(53:11.78332109,(((57:5.872841588,58:5.872841587):1.418155276,59:7.290996864):0.5423379724,((63:5.431550948,(61:4.394776892,62:4.394776891):1.036774056):0.2892889793,60:5.720839927):2.112494909):3.949986251):2.422607049):1.54052519,(64:13.05672764,65:13.056727638):2.689725683):10.3563171184):7.468220383):3.391744114,(66:27.98134271,((((74:0.4898011743,75:0.4898011743):10.12532157,(77:1.359962238,76:1.359962238):9.255160502699999):4.721809055,73:15.336931796):0.5580934248,((72:11.8058902754,71:11.805890277):2.166297361,(70:11.00978486,(69:10.224595645,(68:6.815256357,67:6.815256357999999):3.4093392878):0.785189215):2.962402778):1.922837583):12.086317488999999):8.981392237):4.381425491,(78:35.448762531,((((85:13.315940031,84:13.31594003):0.3425367678,((82:10.467695871,81:10.467695872):1.838532672,83:12.306228543000001):1.3522482541):1.196926917,(87:6.936734093,86:6.936734093):7.9186696224):3.661047534,(80:3.015277667,79:3.015277667):15.50117358):16.932311282):5.8953979080000005):2.640514037,(((((((((101:16.6540081978,(((((98:5.3729164477,(97:3.970050553,(95:3.297398496,96:3.297398496):0.6726520564):1.402865896):3.6456404790000003,93:9.0185569278):0.1897875593,94:9.2083444875):3.649535266,100:12.85787975):1.075522961,99:13.93340271):2.720605483):1.302442337,102:17.956450534):6.7273493056,((((122:15.0150164943,((((((106:9.035325781,107:9.035325780400001):3.009671047,(((111:4.710447792,112:4.7104477913):1.605876216,(108:4.732807739,(109:0.1894008932,110:0.1894008932):4.5434068457):1.583516269):2.590991093,113:8.907315101):3.1376817264):0.5377777097,(115:2.47362352,114:2.47362352):10.109151016):0.9092457455,(117:5.9630231323,116:5.9630231325):7.5289971509999996):0.7645396384,118:14.256559921000001):0.05809989204,(121:8.189906313,(120:1.623951462,119:1.623951462):6.5659548517):6.124753500180001):0.7003566832):1.23023451,((105:2.461135882,104:2.461135882):9.471731856,103:11.932867735999999):4.312383269):0.4786538509,((125:7.525346035300001,124:7.525346035799999):5.827412187139999,123:13.35275822245):3.3711466346999996):0.4945039018,126:17.2184087587):7.46539108081):3.196505058,127:27.880304895):2.075381547,(128:9.0093710544,129:9.009371054899999):20.9463153955):8.410371546,((((90:7.068275611,91:7.068275611000001):19.540775999999997,89:26.609051608):1.217509637,88:27.826561247100003):3.994265244,92:31.82082649):6.545231499):4.148665883,130:42.514723883):0.6608771869,(132:34.52133087,131:34.52133086):8.654270194):0.1816980709,(((((((133:1.912529332,134:1.912529332):2.603817272,135:4.516346605):1.124976437,136:5.6413230416):17.099906198,((137:5.458085860500001,138:5.458085861):14.6138494128,(((140:4.024388248999999,139:4.024388249):1.0420598009,141:5.0664480502):3.1185626248,142:8.185010674):11.8869246):2.669293965):10.08445018,(((143:8.203940014,144:8.203940014):18.94823199,(((((((152:2.112183327,151:2.1121833265):3.324405207,(153:0.6435360848,154:0.6435360847):4.79305244807):2.878239263,(150:5.585628094,149:5.585628094):2.729199701):1.351700004,(((156:4.734089428,155:4.734089428100001):2.878813633,157:7.612903061):1.628432236,158:9.241335297700001):0.4251925029):2.527582413,159:12.194110213000002):8.759063636,(((146:12.5883308191,145:12.588330819):4.869884932,160:17.45821575):1.653849334,(148:16.693959794,147:16.6939597941):2.4181052905):1.841108764):2.48738689,161:23.440560739):3.71161127):2.675274192,((162:20.0749701472,163:20.0749701446):2.881461367,((((((164:5.824224048,(166:0.3241894547,165:0.3241894547):5.500034593):3.156496844,(168:2.73355891,167:2.73355891):6.247161981):0.7011946383,(169:3.279701632,170:3.279701632):6.4022138967):0.6009481413,171:10.282863671000001):0.8049247555,(172:3.349864685,173:3.349864685):7.737923742):0.6184876375,((179:6.075418546,(((175:2.37500555,176:2.37500555):1.710783789,(174:3.4109718110380003,177:3.4109718118):0.6748175277999999):0.1192755161,178:4.205064855):1.870353691):1.812069718,(181:4.919981573,180:4.919981573):2.9675066915999997):3.818787799):11.2501554485):6.871014692):2.998233222):0.3617523243,((182:14.652971276999999,183:14.652971277):6.25644507,(186:4.466345326,(185:3.039190622,184:3.039190622):1.4271547046):16.4430710205):12.278015404000001):3.518793489,(187:0.4956976815,188:0.4956976815):36.21052755520001):6.651073895600001):0.627375339):7.247327267499999);

TREE tree_4162 = ((18:29.340878420699998,(((11:7.732859756,12:7.732859756):13.498691207,((1:15.44666991,2:15.446669913000001):3.584633337,((4:12.456630419,((5:2.661178451,6:2.661178451):6.752056842,((7:5.537712334,8:5.5377123343):0.4848855447,9:6.02259787897):3.390637414):3.0433951268):2.502096407,(10:14.390157806,3:14.390157810100002):0.56856901625):4.072576422):2.2002477138):4.564601661,(((13:8.59243815,(14:5.5789756839999995,15:5.5789756849000005):3.013462466):0.8432718597,16:9.43571001):1.118360518,17:10.554070527099999):15.2420820965):3.5447257959):21.696892115,((((66:31.71420217,(((74:10.05148552,(77:9.268836818,(76:0.477064112,75:0.477064112):8.791772706):0.7826487016):2.959142122,((72:11.434603114,71:11.434603113000001):0.8005674971,(((68:7.6483703599999995,67:7.64837036):1.847333261,69:9.49570362):1.979292557,70:11.47499618):0.7601744329):0.7754570302):0.8397182221,73:13.850345867):17.863856305600002):5.3405948930000005,((((((((21:6.234782451,22:6.234782450000001):9.6207850626,(23:8.075663734,24:8.075663734):7.77990378):0.02134564318,(19:4.978043814,20:4.9780438145):10.898869343):3.352161643,(25:10.567735273700002,26:10.567735274):8.661339525999999):5.105088343,27:24.334163144):1.6118383573,28:25.9460015):5.29216206,((30:17.057723409,(31:14.321924018299999,32:14.321924017):2.7357993904):2.197044369,29:19.2547677785):11.983395781):3.28734433,(((52:12.4384183049,((((49:4.9445736848,48:4.944573685):2.124289256,51:7.068862941):0.3953271816,50:7.464190123):2.297366012,(47:7.5992320453,46:7.599232045):2.1623240899):2.6768621718):7.6996582689999995,(((34:4.33489501,33:4.33489501):2.345486226,35:6.6803812360000006):2.970971463,((43:7.549910667,(45:6.5783552181,44:6.5783552180000004):0.9715554487):1.630072795,((((40:2.587331317,39:2.587331317):2.01958255,41:4.6069138667):2.49376419,((36:5.5078035354,37:5.507803536):0.6355654424,38:6.143368978):0.957309079):1.700341274,42:8.801019331700001):0.37896413001):0.4713692382):10.486723876):3.998147324,((64:11.187661499,65:11.1876615006):3.90414729,((53:11.24840027,(((60:6.230564835,63:6.2305648355):0.05009830053,(61:3.561128784,62:3.561128784):2.719534352):0.2093522687,((57:4.089564028,58:4.089564028):2.153880236,59:6.243444264):0.2465711405):4.758384868):1.026595868,(54:10.47432743,(55:6.869919782,56:6.869919782):3.6044076470000004):1.800668711):2.816812649):9.044415112900001):10.389283990000001):2.5292891697):5.565429969,(78:34.331106051000006,((((85:15.049055121999999,((81:10.984727838000001,82:10.984727838000001):1.375684694,83:12.3604125269):2.6886425905):0.7057604615,84:15.754815581499997):0.7717964333,(86:6.717731512,87:6.71773151203):9.8088805045):2.315102134,(80:2.696172585,79:2.696172585):16.14554157):15.489391901000001):8.289120979):1.66795382,(((((((89:23.8634553683,(90:6.921387733,91:6.921387732):16.942067642):3.149703535,88:27.01315890518):1.615504964,92:28.6286638721):6.86304362,((((102:16.188833864400003,((99:11.93883585,(100:11.42364806,((94:8.598124066,93:8.598124066):0.6320868,((97:5.693314345,(95:3.709152254,96:3.7091522538999997):1.9841620909):1.974410747,98:7.6677250923999996):1.562485774):2.193437193):0.5151877938):3.343746339,101:15.2825821928):0.90625167242):6.830743655,((((124:7.188715806,125:7.188715805599999):5.83861558375,123:13.0273313901):2.34532621,((((((((106:8.694801048,107:8.694801049099999):2.794039574,(((108:6.0430553795,(109:0.04320059826,110:0.04320059826):5.9998547819):1.441819553,(111:3.109798376,112:3.1097983752):4.375076557):0.9059848846,113:8.390859817):3.0979808055999998):0.6428436987,(115:2.135350798,114:2.135350798):9.9963335244):0.6465353266,(117:5.59460654,116:5.5946065398):7.183613107999999):1.003886562,((120:1.306833953,119:1.3068339528000001):5.8828395679,121:7.1896735207999996):6.592432688900001):0.09938509825,118:13.8814913071):0.37166124584,122:14.253152551000001):0.1840818511,((105:3.510826468,104:3.510826468):8.046734564,103:11.557561033):2.8796733723):0.9354231956):1.53710101766,126:16.9097586179):6.109818902200001):6.350228414,127:29.369805936000002):0.07652914174,(128:7.561199842300001,129:7.5611998418):21.885135237601):6.045372416):4.640333589,130:40.132041085):2.024146226,(131:28.56321569,132:28.563215698):13.592971618):0.7930057697,(((((((140:2.711343237,139:2.7113432374):3.1314008565,141:5.8427440932):4.4369855004,142:10.279729593999999):6.790992692,(137:4.6012714382999995,138:4.601271438):12.4694508475):3.2917487518,((135:2.530220507,(134:1.3572202,133:1.3572202):1.173000307):0.3372662569,136:2.8674867630999996):17.4949842728):9.405208713,((((143:7.081884476,144:7.081884477):15.63634863,(((((((155:4.733085276,156:4.733085276):1.232946466,157:5.966031742):1.208533673,158:7.17456541535):0.5164498102,(((152:1.5797715632,151:1.5797715637):3.367108778,(154:0.528210637,153:0.528210637):4.418669705):2.44186677,(149:3.268425383,150:3.2684253827):4.1203217296):0.3022681137):1.663954871,159:9.3549700969):5.758981146,(((146:9.8846884196,145:9.88468842):2.63243566,160:12.51712408):1.5307380133000001,(148:12.2615716594,147:12.261571659000001):1.7862904340999999):1.066089149):2.55464419,161:17.668595431):5.049637671999999):0.6188520754,((162:17.9898715022,163:17.989871504):3.418021653,(((171:9.928204275,(((164:6.390820451,(165:0.1214128788,166:0.1214128788):6.269407573):1.90341685,(168:3.50254422,167:3.50254422):4.791693082):0.5990154736,(169:2.133394422,170:2.1333944216100003):6.759858353):1.0349515):2.407647516,(172:4.995306753,173:4.995306753):7.340545038):0.288232134,(((178:5.79771883,((174:4.76092015,(175:4.271855474,176:4.271855474):0.4890646759):0.0397248531,177:4.8006450027000005):0.997073828):1.535785762,179:7.333504593):1.551949232,(180:4.441350102,181:4.441350102):4.444103723):3.7386301002):8.783809227399999):1.929192026):3.270899997,((183:16.07665216,182:16.0766521603):5.45033766,(186:5.91050683,(185:2.889396518,184:2.8893965181):3.0211103117):15.616482989760001):5.08099535973):3.159694571):6.929930644,(187:0.1458120609,188:0.1458120609):36.551798325):6.251582686000001):1.33898777):6.749589686);

TREE tree_4905 = ((18:37.520279508600005,(((11:8.940655628,12:8.940655628):17.87249138,((1:20.60462631,2:20.604626312):2.260783266,(3:19.946957107000003,((((5:3.0937210754999995,6:3.0937210751000004):9.335180245,(9:8.4787372455,(8:6.6751107046,7:6.675110705):1.8036265406):3.950164075):1.164349068,4:13.593250389000001):4.972077434,10:18.5653278225):1.3816292801999999):2.9184524759999997):3.947737428):4.070746489,((16:12.83699864,((15:6.859300596,14:6.859300596):3.629549124,13:10.488849721000001):2.3481489178999997):0.2683325152,17:13.10533115407):17.778562343):6.636386013999999):11.961296506199998,((((((28:30.087475598,(((((21:5.947045657,22:5.947045656):11.272910354,(24:10.1880230118,23:10.1880230109):7.031932999):0.871737585,(19:6.904089886,20:6.9040898869000005):11.18760371):1.377654802,(25:13.400824083,26:13.400824083099998):6.068524314299999):6.294715895,27:25.764064291):4.3234113047800005):3.175080482,((30:16.209959631,(31:13.498255005419999,32:13.498255003799999):2.7117046275999996):3.6991460022,29:19.909105634):13.353450446):2.369674579,(((((54:9.486915922,(55:7.5495982456,56:7.549598246):1.9373176769):1.78796596,(((57:5.365067978,58:5.365067978):1.695203348,59:7.0602713260000005):1.450231154,((61:5.64971544,62:5.649715441):1.045718094,(63:5.891255937,60:5.891255937):0.8041775975):1.8150689461999998):2.764379403):0.8675523783,53:12.14243426):3.098777212,(64:12.48113912,65:12.481139122):2.760072353):14.4169348414,(((35:6.711944831,(34:4.827551585,33:4.827551585):1.8843932453):5.90069313,(((((37:6.150020312400001,36:6.1500203117):0.2460298966,38:6.396050209):2.219920913,((40:5.251479682399999,39:5.251479681999999):1.69121735,41:6.942697032):1.673274089):1.184309711,42:9.800280831999999):2.172777506,((45:6.8147925048,44:6.814792505):3.283586609,43:10.098379114):1.8746792245000001):0.6395796228):14.800634828,(52:17.4912983543,((((49:6.462017125,48:6.462017126):2.284928347,51:8.746945472):0.4027722006,50:9.149717673):4.331321009,(47:9.724997485,46:9.724997485):3.756041198):4.0102596688):9.921974442):2.2448735177000003):5.974084345):1.5524933356,(66:27.90309305,(73:16.247684726,((((69:9.035471255600001,(67:4.8938788599,68:4.89387886):4.141592396):0.693984579,70:9.729455835):3.140307271,(77:7.835081622000001,((75:2.552458293,76:2.552458293):0.6934567774,74:3.24591507):4.589166551):5.0346814842):0.5137420318,(72:11.275128095,71:11.275128095):2.108377043):2.864179588):11.655408324):9.281630949):4.219308542,(78:36.944488875,((((84:15.037495659000001,85:15.037495659000001):0.1242613064,((82:11.26264195132,81:11.262641950999999):0.3968888643,83:11.659530817603):3.50222615):1.08494631492,(86:9.634024705,87:9.634024705):6.6126785759999995):2.081467753,(79:1.936249871,80:1.936249871):16.391921162):18.616317845999998):4.459543661):2.261503153,(((132:29.612644680000002,131:29.612644676000002):13.20131228,(((((((((((105:2.538039392,104:2.538039392):9.174709438,103:11.712748827999999):3.978347245,((((((106:8.304176718,107:8.304176718599999):3.973187274,(((108:6.0635723,(109:0.3486647123,110:0.3486647123):5.714907587):0.5431435012,(111:3.013499713,112:3.0134997129):3.593216088):2.780720633,113:9.3874364332):2.8899275584999997):1.269484763,(114:2.924363185,115:2.924363185):10.62248557):0.7674114247,(117:5.873083534,116:5.873083533970001):8.441176645999999):0.6108172449,118:14.925077423999998):0.09904104436,((119:1.297405914,120:1.2974059141):5.7429161304,121:7.0403220455):7.9837964248):0.66697760486):0.2537584057,122:15.9448544826):1.810054376,((124:8.00244602175,125:8.002446021999999):5.989738195599999,123:13.9921842185):3.7627246383000004):0.6038818249090001,126:18.3587906809):5.801190809,(102:17.4452322954,(101:16.44628922,(100:13.29277902,(99:13.21438627,((93:9.4695711196,(98:6.3538854778,(97:4.839681965,(95:3.574270909,96:3.574270909):1.265411056):1.5142035143):3.11568564183):0.3214436071,94:9.7910147269):3.423371543):0.07839274869):3.1535102029999997):0.9989430736):6.7147491939999995):1.50016669,127:25.660148176):1.503940441,(128:7.401638108899999,129:7.4016381083):19.762450511039997):13.22961939,(((89:24.629169909,(90:6.453453843,91:6.453453842):18.175716065):1.810127974,88:26.439297881399998):7.771193933,92:34.210491816099996):6.183216194):1.165464623,130:41.5591726266):1.254784328):0.3731438447,((((((135:2.348819668,(134:0.9990262126,133:0.9990262126):1.349793455):2.090046071,136:4.4388657385):16.8956225447,((138:6.417454300999999,137:6.417454301299999):13.456611273,(142:11.105416684,(141:7.610673338,(140:4.913947343,139:4.91394734298):2.696725996):3.494743347):8.768648894):1.46042270147729):6.908287211,(((143:6.630907617,144:6.630907617):19.54343547,(((145:12.699397087000001,146:12.6993970873):5.682952696,(((147:13.9646829746,148:13.96468297584):2.1673112903,160:16.131994265):0.9066017888,((((150:6.658001892,149:6.658001893):0.2477041165,((151:1.3112809903,152:1.3112809902):3.584087708,(154:0.611083486,153:0.611083486):4.284285213):2.0103373104999998):1.100940319,(((155:4.610734412,156:4.610734412):2.133307035,157:6.744041446500001):1.002958328,158:7.7469997737):0.2596465527):3.042153363,159:11.0487996894):5.9897963635):1.343753729):1.671960447,161:20.05431023):6.120032861):0.6580731786,((162:20.49137114,163:20.491371148):4.873600355,(((171:8.349006323,(((164:4.743046468,(165:0.3004973568,166:0.3004973568):4.4425491115):1.137708619,(168:1.357753756,167:1.357753756):4.5230013307):0.9130536465,(170:1.69132477,169:1.6913247702):5.102483963):1.555197589):0.8974311736,(172:3.128641541,173:3.128641541):6.117795955):0.7902865115,(((((176:2.608624547,175:2.6086245466999998):1.135768258,(174:3.419344307,177:3.4193443069000002):0.32504849776):0.7173821107,178:4.461774916):1.012052716,179:5.473827632):1.324453265,(180:3.547904145,181:3.547904145):3.2503767515):3.2384431116):15.3282474911161):1.467444766):1.410359225):3.247357415,((183:15.12658916,182:15.126589161):7.098272088,(186:6.8744253636,(184:2.8086336586,185:2.8086336584):4.0657917046000005):15.350435886):9.26527166):2.825259515,(188:0.1186900657,187:0.1186900657):34.1967023526):8.8717083812):0.4784348863):5.8160403254999995);

TREE tree_4098 = ((18:33.2307725005666,(((11:9.607910637,12:9.607910637):13.214006665,((1:20.07442931,2:20.074429309):1.0007267,(((4:14.537249991,((5:3.7729045839,6:3.772904584):8.656289303,((7:7.06141279388,8:7.0614127946):0.7282972682,9:7.789710062):4.639483825199999):2.1080561029):2.80877739,10:17.346027385):0.2644660392,3:17.610493419799997):3.4646625909999997):1.7467612919):5.155157677,((13:9.524514962000001,(16:8.70153383,(14:5.659748636,15:5.65974863617):3.041785195):0.82298113066):1.715611264,17:11.2401262257):16.736948752300002):5.253697529699999):17.689291030030002,((((((((((((37:6.250677307799999,36:6.250677307):1.252353446,38:7.503030752500001):2.894946333,((39:4.885694606,40:4.885694606):1.944257248,41:6.829951854):3.5680252318299996):1.336266868,42:11.734243953):1.336025827,((44:7.306597268000001,45:7.306597267999999):2.538527581,43:9.845124849):3.2251449316):0.8933189361,((33:5.619213621,34:5.619213621):4.369705228,35:9.988918849000001):3.974669867):11.648847835000002,(((47:10.97384281,46:10.973842808):3.212943045,((48:6.396395623,49:6.396395623):4.750053499,(50:10.7863204335,51:10.786320434):0.3601286884):3.04033673):3.856341007,52:18.0431268613):7.5693096870000005):4.4176301726672005,((30:17.250822566,(31:16.5504740151,32:16.5504740171):0.7003485511):2.207171188,29:19.4579937532):10.57207296708):3.186411541,(((64:8.107724572,65:8.1077245723):3.898312347,((((57:3.096498532,58:3.0964985319):2.449043673,59:5.545542204699999):0.464086515,((63:3.963293967,(61:2.860985274,62:2.8609852745):1.102308692):1.312830118,60:5.276124084):0.7335046355):1.925589858,((54:7.289778032,(56:3.7240020153,55:3.7240020155):3.565776017):0.4206757688,53:7.710453801):0.2247647766):4.070818341):16.25520727,((((((21:6.627616453,22:6.627616453):9.784110584,(24:7.1999111385,23:7.199911138999999):9.211815898):0.4577577802,(19:5.111453031,20:5.111453030790001):11.75803179):1.045038734,(25:12.1608236593,26:12.160823658830001):5.7536998916):5.606335473,27:23.520859025000004):1.902094139,28:25.422953164):2.8382910260000003):4.955234075):5.783875603,(66:23.57369083,((((71:10.313547760999999,72:10.313547759999999):1.579103176,(70:11.11650413,(69:9.6217842525,(68:5.124186329,67:5.1241863290000005):4.497597924):1.494719876):0.7761468069999999):0.5111372547,73:12.403788187):1.020574965,(74:10.12293723,((75:4.82088972,77:4.8208897204):1.009874087,76:5.830763807):4.292173428):3.3014259196999998):10.1493276749083):15.4266630356):3.542623605,(78:36.6186315357,((80:3.920768331,79:3.920768331):15.26979354,(((((82:11.4577202946,81:11.457720295000001):0.9495398056,83:12.4072601):2.5811435878999998,84:14.988403686200002):0.4360612265,85:15.424464915599998):0.934924914,(86:9.767725435,87:9.767725435):6.5916643944):2.8311720409):17.428069666):5.92434593376):1.80819131,(((((((((101:7.451362345100001,((99:6.498189062,((94:4.7551532636000005,93:4.755153263883):0.05308438198,((97:2.670774978,(95:1.328880712,96:1.3288807118000001):1.34189426706):0.2803478249,98:2.9511228033000005):1.85711484287):1.689951416):0.1888864767,100:6.687075539):0.7642868058):0.08685601723,102:7.538218362399999):4.9842562178400005,(((((103:5.034756005,(104:1.26090361,105:1.26090361):3.77385239517):2.3462173560000004,(((((106:3.9849621829,107:3.9849621834):1.640847185,((((109:0.07912224518,110:0.07912224518):2.5062364802,108:2.585358725):0.5887494163,(111:2.292770681,112:2.2927706816):0.8813374603):1.046042385,113:4.2201505273):1.4056588409):0.3496569061,(114:1.194952983,115:1.194952983):4.7805132906):0.2660560797,(117:3.0558218413000002,116:3.0558218417100003):3.1857005117):0.7508046394,(118:6.915401102000001,((120:0.8738321906,119:0.8738321906):2.4753339691,121:3.3491661599):3.5662349416500003):0.07692589098):0.3886463682):0.07356123892,122:7.4545345996000005):0.4769902753,((125:4.8438920412,124:4.8438920411):1.50611936787,123:6.3500114098000005):1.5815134665):0.49140767234,126:8.422932547):4.0995420324000005):7.626438915,127:20.148913489):0.7981448447,(128:6.2463877729999995,129:6.246387772499999):14.70067056731):15.68515437,(((89:26.213096966,(90:4.98491365,91:4.98491365):21.2281833234):2.368580524,88:28.5816774934):1.061936539,92:29.643614037):6.988598677):3.642834668,130:40.275047372):1.426744858,(132:20.994042621000002,131:20.994042620000002):20.707749617):0.860594352,((((((143:9.21907195,144:9.21907195):16.7832912,(((((((151:1.8767089609,152:1.8767089611999999):3.370017813,(154:0.498199026,153:0.498199026):4.748527748):2.180024725,(149:6.270745084,150:6.270745084):1.1560064152999998):0.287063683,(((155:3.987075289,156:3.987075289):2.266899013,157:6.25397430187):1.188942464,158:7.4429167651):0.270898417):3.997154513,159:11.710969696):5.794709253,((160:16.594355187,(147:13.935589871,148:13.935589871600001):2.6587653156099997):0.4678877066,(146:11.0489916213,145:11.0489916177):6.013251271):0.4434360567):2.058305767,161:19.5639847164):6.438378438000001):0.906269596,((162:19.0581021417,163:19.058102141):5.711255379,(((171:11.99749736,((((165:0.3829339612,166:0.3829339612):6.677512529,164:7.06044649078):2.213606451,(168:2.009937426,167:2.009937426):7.264115516):1.944014081,(169:2.553536074,170:2.5535360739):8.664530948):0.779430333):1.42085369,(173:5.828310236,172:5.828310236):7.59004081):0.479995295,((((((174:5.608173373,175:5.608173373):0.2956452742,177:5.9038186478):0.238039253,176:6.1418579):1.302954186,178:7.444812086):0.9611500267,179:8.405962113):1.121877768,(180:6.836258499,181:6.836258499):2.6915813823000003):4.370506458):10.871011185):2.13927523):3.979015732,(((((140:4.78992647,139:4.7899264705):0.3118691375,141:5.101795607899999):5.646759808,142:10.748555416):6.325784953,(137:4.7070711671,138:4.7070711675000005):12.3672692056):2.600531492,((135:1.747562848,(134:0.7931279268,133:0.7931279268):0.9544349209):0.9640564774,136:2.7116193248):16.963252536):11.21277662):1.761843055,((183:19.530316250000002,182:19.5303162527):3.033655067,(186:7.341229642,(185:2.866633438,184:2.866633438):4.4745962044):15.222741681):10.085520221):2.905402334,(187:0.4802054971,188:0.48020549710000004):35.0746883674453):7.0074927129999995):1.788782195):6.568894756);

TREE tree_5796 = ((18:38.6579090869215,((((1:20.81329492,2:20.813294921999997):2.738999026,((((((7:6.166650855,8:6.1666508544):2.326579447,9:8.4932303018):4.8741685345,(5:3.498712061,6:3.498712061):9.8686867747):3.849570712,4:17.216969552000002):2.114660201,10:19.33162975):0.5586123933,3:19.890242142520002):3.662051801):2.780548682,(11:12.21487355,12:12.21487355):14.117969068999999):6.333811436,((((14:5.11677965,15:5.11677965):5.803060442,13:10.919840092):2.0821833344,16:13.002023425500001):0.4451350832,17:13.447158509400001):19.2194955537):5.9912550287999995):17.399989845,((((66:29.92757331,(((71:15.61695142,72:15.6169514202):2.037186658,(((68:6.308570824,67:6.308570824):5.4121030774,69:11.7206739015):3.467593323,70:15.188267225):2.4658708531):0.4755925374,(((76:4.446091427,74:4.446091427):6.278135574,(75:0.6636203114,77:0.6636203114):10.060606689):5.942006874,73:16.666233871):1.46349674):11.797842695100002):14.690801364499999,((((30:21.368833157,(32:19.276899302,31:19.276899298800004):2.0919338539):1.845300563,29:23.2141337198):14.248697944,((52:20.482478751,((47:12.063604631,46:12.063604634):4.389548634,((48:8.305031601,49:8.3050316003):4.155312454,(50:11.480856743,51:11.480856742):0.9794873125):3.992809212):4.0293254809):13.264827036,((((((39:7.606621947,40:7.606621947):3.023457946,41:10.630079893):2.805901974,((37:8.210874305,36:8.2108743048):1.201303144,38:9.4121774488):4.023804418):0.9067723161,42:14.3427541839):1.23280022,(43:14.39177125,(45:11.443235108,44:11.443235108):2.948536143):1.18378315216):1.594016678,((34:7.305898801,33:7.3058988004000005):2.338421597,35:9.644320397):7.525250683):16.577734704):3.7155258748):1.165212133,(((((((24:8.5960199853,23:8.596019986):6.9761352819999995,(21:6.715892348,22:6.715892348):8.856262919999999):0.2451951577,(19:3.953012494,20:3.9530124935):11.86433793):3.100755792,(25:13.007229871,26:13.0072298708):5.9108763457):5.544543723,27:24.462649935199998):2.714946642,28:27.177596582):8.7250514978,((64:11.45718196,65:11.457181963):4.404434258,(((54:9.336780701,(55:5.622786564,56:5.6227865632):3.713994138):0.7835002455,(((57:4.000109247,58:4.000109247):3.371138288,59:7.371247535):0.294197244,((61:3.412596501,62:3.412596501):1.518924924,(63:4.008075999,60:4.008075999):0.9234454266):2.733923354):2.454836167):1.773517797,53:11.89379874):3.967817476):20.0410318644):2.725395708):5.990330882):5.314100082,(((((((82:11.318933002,81:11.318933002):1.020121456,83:12.3390544631):3.329720076,85:15.668774534299999):0.5971218701,84:16.265896405499998):0.3063643877,(86:8.621067259,87:8.62106726):7.9511935330000005):3.846959689,(80:2.512125018,79:2.512125018):17.907095462):19.081284963999998,78:39.500505446999995):10.431969314):1.523629182,(((((((89:30.826244371,(90:8.68795712,91:8.68795712):22.138287251):2.260320782,88:33.0865651571):6.089348494,92:39.175913646):7.783826081,(((((101:18.9769245854,((100:15.43152986,99:15.431529858000001):0.8977969931,((94:10.419004664,93:10.419004665300001):1.397520413,((97:7.577290744,(95:4.918617796,96:4.9186177959999995):2.6586729475):1.64413953,98:9.221430274300001):2.59509480436):4.512801775):2.647597737):1.325787168,102:20.302711756700003):6.370045462,(126:20.5577797778,(((((105:4.451685509,104:4.451685509):10.06763538,103:14.519320889):2.658086816,((((((106:10.04913494,107:10.04913493811):3.575461964,(((108:6.95966066734,(109:0.2096079826,110:0.2096079826):6.7500526847):2.109252629,(111:4.085192184,112:4.0851921839):4.983721113):2.303490313,113:11.372403608699999):2.2521932934):0.4574948638,(115:2.940362096,114:2.940362096):11.14172967):1.060387736,(117:6.571959918,116:6.5719599183):8.570519583780001):1.36346112,118:16.505940622):0.09448306796,((120:1.572254301,119:1.572254301):7.5849609854999995,121:9.157215286100001):7.443208403):0.5769840185):0.9815124895,122:18.158920200700003):1.030681841,(123:15.210837103,(124:7.9219776957,125:7.9219776956):7.2888594087800005):3.9787649345):1.3681777356):6.1149774462):6.493656245,127:33.166413463):1.011042498,(128:9.2509283963,129:9.2509283971):24.926527563000004):12.78228377):1.395458715,130:48.355198451999996):0.6696275948,(131:43.91034956,132:43.910349561000004):5.114476483):0.4547044837,((((((138:7.4596128010000005,137:7.4596128014000005):11.4761868448,(((139:6.396181468,140:6.3961814682):2.320106164,141:8.7162876326):5.663123313,142:14.3794109451):4.55638869895):2.2099231513,((135:3.431695843,(133:0.6029918419,134:0.6029918419400001):2.828704001):0.9957916793,136:4.4274875222):16.718235279599998):7.406286423,(((143:5.890922538,144:5.890922538):17.34299879,(((((((156:4.740082685,155:4.740082685):1.904021825,157:6.644104511):1.610993963,158:8.25509847394):0.3270536066,((150:7.734047561000001,149:7.7340475608):0.4432264094,((152:1.1744331035,151:1.1744331035):4.764111396,(153:1.194607787,154:1.194607787):4.743936713):2.238729471):0.4048781093):2.206697538,159:10.7888496176):5.042876687,(((145:10.958253619309,146:10.9582536173):3.432636545,160:14.390890162000002):1.192019508,(147:13.5619994853,148:13.561999483400001):2.02091018605):0.2488166343):2.55972476,161:18.391451064600002):4.842470259):1.542040946,((162:19.124196371,163:19.1241963767):2.760565325,(((171:7.212265607,(((164:5.112096444,(166:1.056117033,165:1.056117033):4.055979411):1.000183685,(167:1.892108312,168:1.892108312):4.220171818):0.8119851702,(169:1.68023178,170:1.6802317808):5.244033519):0.2880003076):1.671239208,(172:2.898167958,173:2.898167958):5.985336856):0.6751048372,(((((174:3.861377295,(176:3.658912643,175:3.6589126423):0.2024646524):0.2982088528,177:4.159586147650001):0.05504285608,178:4.2146290036):1.094700164,179:5.309329168):0.743481173,(180:3.01973398,181:3.01973398):3.03307636):3.5057993119999997):12.3261520417):2.891200568):3.776046949):4.311373597,((183:19.55373062,182:19.553730615950002):3.370409082,(186:7.584558723000001,(185:2.6683768118,184:2.668376812):4.916181911):15.33958097418):9.9392431118):7.559130007,(188:0.2085400553,187:0.2085400553):40.213972770000005):9.057017708):1.97657341):4.601794996000001);

TREE tree_4001 = ((18:26.9654301096975,(((11:9.038171421,12:9.038171421):10.759034842,((1:15.88944419,2:15.889444188):1.569148208,(((4:12.745612563,(((8:5.4185868372199995,7:5.4185868373):0.6082177842,9:6.02680462189):3.433576001,(6:2.511892264,5:2.5118922642):6.948488357999999):3.2852319408):1.580014111,10:14.325626672):0.7666052923,3:15.092231967):2.3663604298):2.3386138674):4.96934515,(((13:8.269235736,(15:4.937850702,14:4.937850702):3.331385034):0.3560518597,16:8.625287596):0.3089715788,17:8.9342591744):15.832292238):2.1988786973):21.7758027974,((((((((((((19:4.668043742,20:4.6680437423):10.12342864,(21:5.426794399,22:5.426794399):9.364677981):0.09470332873,(23:8.495825769,24:8.49582576961):6.39034994):2.285976209,(25:11.9894026343,26:11.9894026346):5.1827492834):5.476189399,27:22.648341314):1.592712484,28:24.241053801):6.894906699,(29:18.8354333852,((32:12.5043544,31:12.5043544):3.0342182485,30:15.538572647999999):3.2968607369000003):12.300527115):0.0138051329,(((((((37:7.593371448999999,36:7.5933714491):1.042752153,38:8.63612360138):3.863327907,((39:6.248729569,40:6.248729569):2.293912926,41:8.5426424947):3.956809014):0.9996197366,42:13.499071246):0.9963417208,(43:12.6998604,(44:8.077052893,45:8.0770528931):4.622807502500001):1.7955525705):0.9346041764,((33:5.302475225,34:5.302475225):3.864073981,35:9.166549207):6.263467936):13.762716315599999,(52:19.1389636418,(((50:10.145354709100001,51:10.1453547094):0.4598929748,(48:7.245932982,49:7.245932982):3.359314702):4.96776048,(47:12.1273212109,46:12.12732121):3.445686954):3.5659554731):10.05376982):1.9570321826564):1.731478449,((53:13.06248274,((((58:5.6395219569999995,57:5.639521957):1.600590452,59:7.240112409):1.544203549,((63:6.798797986,(61:4.797292134,62:4.797292134):2.001505852):0.9758793566,60:7.774677342):1.009638616):3.320792359,(54:11.59908809,(55:10.06090202,56:10.060902023659999):1.5381860686):0.5060202259):0.9573744215):4.224497917,(64:15.1304037,65:15.1304036927):2.15657696):15.5942634324):3.879978287,(66:29.08527485,((((((67:6.112648232000001,68:6.1126482314):3.1040052549999997,69:9.216653488):3.159750812,70:12.3764043):1.439116082,(77:5.9371936729999994,((75:1.608380584,76:1.608380584):1.46795922,74:3.076339804):2.860853869):7.8783267089999995):2.462595038,(72:15.067839410000001,71:15.067839411000001):1.210276011):0.4983667854,73:16.776482203):12.3087926439):7.675947529):5.05077663,(78:37.161633503999994,((79:2.319406129,80:2.319406129):16.80265647,((((87:9.034332468,86:9.034332468):7.1760904197,((82:11.299131924920001,81:11.2991319251):0.8887408215,83:12.187872750999999):4.0225501412):0.164413249,84:16.374836137):0.2362737899,85:16.6111099273):2.510952667):18.03957091):4.6503654959):2.030003388,((((((((90:7.65300537,91:7.65300537):19.519635854,89:27.17264122):5.156341471,88:32.328982693330005):3.608846551,92:35.937829252600004):4.715381202,((((102:16.0528773271,(101:15.4169888884,((100:11.87365705,99:11.87365705):1.128305913,(((((95:3.14275188,96:3.1427518801):2.940513902,97:6.0832657815):0.6977690394,98:6.7810348212000005):1.8697307939,94:8.650765614900001):0.03882555484,93:8.68959117):4.3123717930000005):2.41502592507):0.6358884401):9.188556673,((((((((((106:10.27302888,107:10.27302888136):2.741798644,(((112:4.1521205116,111:4.1521205100000005):3.782215409,((109:0.1848583605,110:0.1848583605):7.097381458499999,108:7.282239818000001):0.652096101):1.507350527,113:9.4416864459):3.5731410783):1.12564859,(115:2.383930685,114:2.383930685):11.75654543):0.8622410399,(117:6.369733123,116:6.3697331230000005):8.632984031700001):0.2996191649,118:15.302336319):0.6093311082,((120:1.172688326,119:1.1726883263999999):5.759626434399999,121:6.932314760400001):8.9793526675):0.31757409744,122:16.229241527):0.3393062237,((105:3.788421308,104:3.788421308):10.69921297,103:14.487634273000001):2.0809134756):1.080399731,(123:14.356126061700001,(124:8.6395560785,125:8.6395560793):5.716569982):3.292821418):0.290667991305,126:17.93961547197):7.3018185283):1.422751156,127:26.664185163):0.7780845514,(128:7.927044595,129:7.9270445948):19.51522511606):13.21094074):0.09973877553,130:40.752949234):1.515729947,(132:27.338757270000002,131:27.33875726):14.92992191):0.1394922101,((((((143:7.665673287,144:7.665673287000001):16.48323519,((((160:16.204787808,(148:15.15149612321,147:15.151496124786):1.0532916839):0.4701007724,(146:12.10080236,145:12.100802359):4.57408622):0.8721899385,((((((151:1.9220136579,152:1.9220136577):3.536891302,(154:0.8881899065,153:0.8881899065):4.570715054):3.048909429,(150:5.535203437,149:5.535203437):2.9726109530000002):0.4906480715,((155:4.86960699,156:4.8696069895):2.721266972,157:7.590873962):1.407588499):0.05488306292,158:9.0533455242):1.912884932,159:10.966230456000002):6.5808480639999996):2.524752029,161:20.071830548):4.077077931):0.1906549807,((162:16.6287120302,163:16.6287120312):2.068607148,(((171:8.176709386,(((164:5.263376953,(165:0.09999369454,166:0.09999369454):5.1633832580000005):1.567623509,(168:2.884639095,167:2.884639095):3.9463613669999997):0.4933395113,(169:1.757029339,170:1.7570293393):5.567310634):0.8523694125):1.982899777,(172:4.114054312,173:4.114054312):6.045554849999999):0.2373833505,(((178:4.774907996,((174:3.921017275,(175:3.518231472,176:3.518231472):0.4027858024):0.0327167502,177:3.9537340253):0.8211739708400001):1.264848457,179:6.039756453):1.278160431,(180:3.657824523,181:3.657824523):3.6600923610000002):3.0790756299999997):8.3003266709671):5.642244282):3.736692632,(((137:6.7694575129,138:6.769457512800001):10.643855959,(142:10.7802290415,(141:5.797688967999999,(140:2.362801412,139:2.3628014121):3.434887556):4.982540074699999):6.633084429):3.1999474450000003,(((134:1.529050691,133:1.5290506911):1.501045957,135:3.030096648):0.7047767665,136:3.7348734143):16.878387503):7.462995172):1.370127686,((183:18.574756903999997,182:18.5747569079):0.9044824686,(186:7.198717899,(185:3.93244760167,184:3.932447601):3.266270297):12.280521477):9.967144404):5.384110252,(187:0.04065803207,188:0.04065803207):34.7898360001):7.577677354):1.433831006):4.8992305222);

TREE tree_8222 = ((18:36.6626789939951,(((11:13.55804131,12:13.55804131):12.493474862,((1:22.50336454,2:22.503364547000004):0.7357786094,(3:19.200899472,((((5:2.1738398687,6:2.1738398685):10.974271706,((8:6.16703901892,7:6.16703902):2.265742577,9:8.432781596):4.71532997889):3.848893182,4:16.997004761):0.9766704035,10:17.973675159000003):1.227224311):4.03824368):2.8123730174):5.032239711,(((16:9.358093121,(14:6.498184244,15:6.498184244000001):2.859908877):0.7302133487,13:10.088306470300001):0.8116676361,17:10.8999741056):20.183781772):5.578923111):18.048593527,((((((((64:13.30648804,65:13.3064880346):2.212069526,((54:11.59794012,(55:5.9884753258000005,56:5.9884753260000005):5.6094647964):0.7378479214,(((((61:3.111022547,62:3.111022547):2.816296144,60:5.92731869):0.3401194412,63:6.267438132):1.970540889,((57:4.195572767,58:4.195572767):2.990670546,59:7.1862433119):1.051735708):1.932387238,53:10.17036626):2.165421785):3.182769522):18.3866186873,((((((23:9.90574036,24:9.9057403596):6.487431784,(22:7.551339557,21:7.55133955768):8.8418325869):1.598961577,(19:5.698636614,20:5.6986366144):12.29349711):2.153854711,(26:13.2742126902,25:13.274212685):6.8717757409999995):7.388629536,27:27.534617963000002):2.315222188,28:29.849840155):4.055336095):4.626760053,(29:23.4070175154,(30:19.063192196,(31:16.8937341722,32:16.893734168199998):2.1694580229):4.3438253197):15.124918781):0.05059550291,((((47:14.58089891,46:14.580898914):3.679377501,((50:13.5213989704,51:13.5213989668):1.051735285,(48:8.470725337,49:8.470725337):6.102408919):3.687142156):5.670397814999999,52:23.9306742306):13.024084177999999,(((34:7.614360558,33:7.614360558):2.677774328,35:10.292134885):7.821855248,((43:15.15671592,(45:12.335915777010001,44:12.335915776):2.820800146):1.837354215,((((39:7.672793814,40:7.6727938136):3.795405029,41:11.468198842):2.977451443,((37:9.304437331,36:9.304437331):1.481256493,38:10.785693823999999):3.659956462):0.8346587331,42:15.280309019999999):1.7137611188000001):1.119919995):18.84076826755):1.6277734053000001):3.9142528719999996,(66:34.17216188,((74:14.24156343,((75:3.633175598,76:3.6331755974999997):1.936422876,77:5.569598474):8.671964956):5.085652232,(((71:14.038092358,72:14.038092358):2.757925592,73:16.796017951):1.102586974,(((68:5.799388918,67:5.7993889186):6.584923324,69:12.384312242):0.5778529645,70:12.96216521):4.936439718):1.428610738):14.844946217):8.3246228):3.572378054,(78:39.858977865,((((((81:12.12465850947,82:12.124658509):0.866050392,83:12.9907088999):3.41030055,85:16.401009451):0.3495092874,84:16.7505187344):0.7503049826,(86:10.60135906,87:10.60135906):6.899464663600001):2.185045093,(80:2.022803628,79:2.022803628):17.663065189):20.1731090462):6.210184869000001):1.587336313,(((((((89:36.21183549,88:36.2118354981):0.3937025289,(90:8.965156195,91:8.9651561949):27.640381830000003):4.181415797,92:40.7869538186):1.115343607,(((((101:20.597297982999997,((100:13.95351143,99:13.95351143):3.357633962,((93:11.4040520754,(((95:4.358562437,96:4.358562437):2.027491519,97:6.38605395554):1.089924577,98:7.475978532000001):3.92807354201):0.3791876672,94:11.7832397452):5.527905651):3.286152593):0.8657277863,102:21.4630257726):7.883304539,(126:22.452408905000002,(((((105:3.488299142,104:3.488299142):11.36625774,103:14.854556880999999):5.111161201,((((((106:9.580023988,107:9.5800239868):6.44929484,((((109:0.1302773559,110:0.1302773559):7.5238522396,108:7.654129596):1.656248111,(111:5.653372157,112:5.6533721572):3.657005549):1.815957085,113:11.126334790799998):4.902984037):0.9167393072,(115:4.172113025,114:4.172113025):12.773945109299998):1.047876408,(116:8.3468853476,117:8.346885347):9.647049196000001):0.8380704709,118:18.832005013):0.2657837756,((120:1.73048353,119:1.73048353):8.372502798100001,121:10.102986329):8.9948024609):0.8679292948999999):0.3097871938,122:20.2755052769):1.063057211,((125:10.1038219085,124:10.1038219078):7.297205458614,123:17.4010273668):3.9375351213):1.1138464165):6.893921405):3.034319589,127:32.380649904):0.03115252344,(128:9.244145463,129:9.244145463):23.167656956000002):9.490495003):1.815916865,130:43.71821429):3.3284591,(132:26.389586070999997,131:26.38958607):20.657087318):0.09706487985,((((((143:5.867833873,144:5.867833873):17.48785734,(((((((156:3.6884425910000003,155:3.6884425910000003):3.034559894,157:6.723002485):1.401401828,158:8.124404313):0.05399766617,((150:5.722491564,149:5.722491564):1.350663794,((154:0.4248209893,153:0.4248209893):4.241778888,(151:1.0516887453000001,152:1.0516887453):3.614911132):2.406555481):1.1052466209):3.348825571,159:11.52722755):5.124704978,((147:12.4604719054,148:12.4604719057):2.7629991084000003,((146:10.0021926274,145:10.002192628):4.12543241,160:14.127625038):1.0958459765600002):1.428461514):3.316546008,161:19.968478537):3.387212676):1.886081358,((162:12.247692453,163:12.2476924495):9.960819238,(((171:8.192086096,(((164:4.898663703,(165:0.561699583,166:0.561699583):4.336964119619999):0.9441596272,(167:2.094551957,168:2.094551957):3.7482713739999998):1.514894884,(169:0.8733950348,170:0.8733950348):6.48432318):0.8343678819):0.8699146253,(172:2.5882494830000002,173:2.5882494830000002):6.473751239):0.04325331755,(((178:4.427885367,(((174:2.550437797,175:2.5504377971):0.6282521703,176:3.178689968):0.007302579863,177:3.1859925479999998):1.2418928189):0.5583820744,179:4.986267441):0.3957831863,(181:3.858726052,180:3.858726052):1.5233245753):3.723203412):13.1032576461103):3.033260886):4.156563598,((182:13.1653364837,183:13.1653364856):7.6687315,(186:5.309818133,(185:1.482030744,184:1.4820307437):3.8277873894):15.524249849499999):8.5642681873):0.8496643163,((((141:7.599755076000001,(140:4.047406533,139:4.0474065333):3.552348543):2.478526719,142:10.078281795399999):8.1426100462,(137:5.67090687502,138:5.6709068755):12.54998496596):3.2553713773,((135:3.2984598,(134:2.255370866,133:2.255370866):1.043088934):0.3787226964,136:3.6771824966):17.799080722000003):8.771737273):4.516498872,(188:0.2182695315,187:0.2182695315):34.546229823892205):12.379238913000002):0.5127607746):7.0547734764);

TREE tree_9144 = ((18:34.785619219299996,(((11:12.42977901,12:12.42977901):11.682818967,((1:21.35047435,2:21.350474351):0.131990332,(3:18.317452826,((4:15.459384808,(((7:5.64379242,8:5.6437924189):2.881916231,9:8.525708651399999):2.169303395,(6:3.7985186090000003,5:3.7985186090000003):6.8964934375):4.7643727619):2.413278542,10:17.872663354):0.4447894735):3.165011859):2.6301332917):6.39075228,(((13:9.16707078,(15:5.348269904,14:5.3482699040499995):3.8188008765):1.098085455,17:10.265156236700001):0.5869951042,16:10.852151340999999):19.651198909399998):4.2822689651):19.1793778548,(((((((((((23:9.410550305000001,24:9.410550305000001):5.407439131,(21:6.805315847,22:6.805315847999999):8.012673589):1.166147318,(19:5.116383235,20:5.116383234699999):10.867753521):2.038296017,(25:11.029973579,26:11.029973579):6.99245919338):5.341131655,27:23.363564426000003):1.686915137,28:25.050479564):8.441932145,((30:18.009123705,(31:16.2773715525,32:16.2773715506):1.7317521529400002):2.547738588,29:20.556862293000002):12.9355494169):1.729879497,(((64:10.708877589,65:10.708877589):3.632417391,(((54:9.200138499,(55:6.059908917,56:6.0599089167):3.140229582):0.9639327031,(((57:4.456595073,58:4.4565950739):1.325727451,59:5.782322524):0.4635285007,((63:4.955877209,60:4.955877209):0.02309943234,(61:4.486629716,62:4.4866297155):0.4923469253):1.266874384):3.918220177):0.719253043,53:10.88332425):3.457970735):10.361685589699999,((((33:2.990817252,34:2.9908172515):2.591218142,35:5.582035394):5.402497383,(((45:7.3852863629,44:7.3852863630000005):1.793244065,43:9.178530428):1.1821408502000001,((((40:4.402266833,39:4.402266833):2.844283661,41:7.246550493299999):1.404010694,((36:6.102599752,37:6.1025997515):0.8257266828,38:6.928326435):1.722234752):0.9682690447,42:9.618830231999999):0.7418410461):0.6238614984):11.051204063,(52:13.42845848452,((((48:4.293700779,49:4.2937007782):2.827881683,51:7.121582461):0.7387695721,50:7.8603520333):3.504699129,(47:8.717154777,46:8.717154777000001):2.647896385):2.0634073217):8.607278355):2.667243731):10.519310637):2.6120506789,(66:31.57901719,(((71:11.401443779000001,72:11.401443777999999):2.341940366,((70:10.9744153,((68:4.865246068,67:4.865246067199999):5.5627304092,69:10.427976477000001):0.5464388252):1.5175987,((74:5.320307522,76:5.320307522):3.615535102,(75:1.790341405,77:1.790341405):7.14550121873):3.5561713787):1.251370141):1.203452859,73:14.946837007):16.632180187):6.2553246935999995):7.558952526,(78:37.0832507453,((79:2.415327217,80:2.415327217):16.80328546,(((85:17.01337142,84:17.013371419):0.3591227197,((81:10.994536694,82:10.994536694):3.970719676,83:14.9652563696):2.40723777):0.2483555557,(86:7.022060898,87:7.022060896999999):10.598788797900001):1.597762982):17.864638069999998):8.310043663):2.796737253,(((((((((101:16.600573131,((100:13.01295502,99:13.01295502):0.7302386564,((93:9.3540928141,((97:6.664695432,(95:3.145123072,96:3.1451230717):3.5195723599088):0.7597574607,98:7.4244528932):1.9296399217):0.5519504634,94:9.9060432771):3.8371503946):2.8573794550000002):1.56853595,102:18.16910907721):4.645160082,(126:16.479333432,(((((105:2.431783047,104:2.431783047):8.820200684,103:11.251983731):2.642663725,((((((106:7.820370992,107:7.8203709924):3.383184221,(((108:5.371949561,(109:0.2666006797,110:0.2666006797):5.1053488816999995):1.250905498,(111:3.554636128,112:3.5546361286000003):3.068218931):1.907197518,113:8.5300525772):2.6735026358):0.2992207579,(114:2.248751407,115:2.248751407):9.2540245641):0.3292410197,(117:5.323799275,116:5.3237992761):6.508217714999999):1.190670812,118:13.0226878026):0.3029184392,((119:1.725251539,120:1.725251539):4.8769791654,121:6.602230703399999):6.7233755382):0.5690412143):0.1955832027,122:14.090230659249999):1.947915849,(123:13.2993319216,(124:6.8347190500999995,125:6.8347190505):6.4646128709):2.7388145859):0.44118692586):6.3349357271999995):3.246495413,127:26.0607645703):0.4602090881,(128:7.0663859736,129:7.0663859735):19.454587687700002):14.14288642,((((90:8.565244862,91:8.565244862):20.107907592,89:28.673152448):3.482697125,88:32.15584957442):5.82318484,92:37.9790344119):2.684825665):5.922706847,130:46.586566938000004):0.6184205339,(132:31.852722936,131:31.85272293):15.352264521):0.2890980803,((((((138:2.8314740097,137:2.8314740095600004):17.5688935244,(((140:5.8384252943999995,139:5.8384252953):0.8413928598,141:6.6798181539999995):5.445622603,142:12.125440758):8.274926776):1.4068686213000001,(((133:3.09782655,134:3.09782655001):0.7654618323,135:3.863288382):0.294784695,136:4.1580730779):17.649163078):9.464299595,(((143:5.524247596,144:5.524247596):19.49328019,(((((146:12.63751116359,145:12.637511164):2.422297459,160:15.059808623999999):1.361887517,(147:14.044028743,148:14.044028743):2.37766739896):0.6544599832,((((153:0.5497212531,154:0.5497212531):4.37821883,(151:1.6457153375,152:1.6457153374):3.2822247465):3.808147243,((149:6.977887036,150:6.977887036):1.560676402,(158:7.8666270059,((156:4.296488665,155:4.296488665):2.51479879,157:6.811287454):1.05533955145):0.67193643212):0.1975238882):1.638796529,159:10.3748838562):6.701272268):2.158237978,161:19.234394102):5.7831336890000005):1.89655962,((162:16.5835967942,163:16.583596795):6.236295905,((((((164:4.587668612,(165:0.6583746122,166:0.6583746122):3.929294):0.8620575104,(168:1.990626402,167:1.9906264025999998):3.4590997210000003):1.311587034,(170:2.393558452,169:2.3935584515199):4.3677547055):0.57107476,171:7.332387917):3.303908189,(173:2.981626055,172:2.981626055):7.6546700508):0.6360855409,((179:6.256776226,(((174:5.111925263,177:5.1119252632):0.6076073351,(175:4.230163809,176:4.230163809):1.4893687896):0.3390239441,178:6.058556542):0.1982196838):1.121947808,(181:5.456411979,180:5.456411978):1.922312056):3.8936576121):11.547511056028398):4.094194707):4.35744834):1.722134117,((182:15.459690389,183:15.459690386):6.12610676,(186:7.615982769,(184:3.396052256,185:3.3960522562):4.2199305123):13.969814381399999):11.40787272):4.39893168,(188:0.04705453374,187:0.04705453374):37.345547015600005):10.10148399296):0.6959461265):5.774965408);

TREE tree_3732 = ((18:40.5201669198,(((11:15.39614664,12:15.39614664):10.526550643,((1:22.90344897,2:22.903448968):1.463506417,((10:18.363277947,(4:13.609909137999999,((6:2.42805606,5:2.4280560601):7.589590296000001,((8:6.382871487,7:6.3828714871):1.262275706,9:7.645147193900001):2.3724991624):3.5922627825):4.753368807):1.2659287956,3:19.629206742):4.7377486446):1.5557419003000001):7.71977112,((16:11.27858858,((15:5.315284134,14:5.315284134):5.064045659,13:10.379329794):0.8992587884):0.5068869674,17:11.785475548700001):21.856992857):6.877698519242):17.852801789711002,(((((((64:13.88085886,65:13.8808588577):3.421104034,((((60:6.968415495,63:6.968415495):0.7405354035,(61:4.13458743,62:4.134587429):3.574363469):0.3028945043,((57:4.715675305,58:4.715675305):2.04357779,59:6.759253095):1.252592308):6.270684174,(53:14.13452282,(54:10.50846961,(55:6.602181186,56:6.6021811861):3.9062884259999997):3.626053211):0.1480067529):3.019433312):21.3863187981,((((((24:10.628130447,23:10.628130447):9.218222309,(22:7.7284156280000005,21:7.7284156280000005):12.117937128000001):0.1818496019,(19:6.141788018,20:6.141788017800001):13.88641434):1.404567307,(25:14.6073609238,26:14.6073609239):6.825408741):5.862321794,27:27.295091461000002):2.542937927,28:29.838029386000002):8.850252293):1.53436113,((((32:20.537866202,31:20.537866206300002):3.7673642736999997,30:24.30523048):2.9042353663,29:27.2094658409):12.397997445000001,((52:23.1705580078,((47:13.12807522,46:13.128075222):3.471820309,((51:12.283588906,50:12.283588905):0.913208392,(49:6.520050945,48:6.520050945):6.676746353):3.403098233):6.5706624757):11.704926803,((((((37:6.5463303633,36:6.5463303631):1.724266102,38:8.270596465):4.633191521,((39:5.14612802,40:5.14612802):4.639934872,41:9.786062891):3.117725094):2.060413501,42:14.964201487):1.699140731,((44:11.120016172,45:11.120016172):3.556663168,43:14.67667934):1.9866628778000002):2.021139621,((33:6.899307063,34:6.899307063):4.035796208,35:10.935103271000001):7.749378569):16.191002971):4.7319784799999995):0.6151795214):2.673706203,(66:35.73525166,(((71:14.013131866999998,72:14.013131868):1.894957304,(((69:12.195243017,(68:4.890567231,67:4.890567231):7.304675786):0.7428231453,70:12.93806616):1.803253908,(((75:4.103032448,77:4.1030324478999995):1.388843779,76:5.491876227):7.498547399,74:12.99042363):1.7508964445):1.1667691):0.1663931824,73:16.074482351):19.660769307000002):7.1610973520000005):2.139347483,(78:42.010619917,((((85:15.311520756,(83:11.45359458,(81:10.655378858999999,82:10.6553788593):0.7982157184):3.857926178):0.5588925614,84:15.870413317):0.4576070389,(87:9.26912192,86:9.26912192):7.0588984363):4.54893046,(80:5.007336692,79:5.007336692):15.86961412):21.133669097):3.025076589):2.367067108,(((((((((((100:13.2973196,99:13.2973196):0.1292317419,(((98:7.054398302300001,(97:5.61963717,(95:4.187987412,96:4.187987412):1.431649758):1.4347611317):2.9003668322,93:9.954765134):0.3025500036,94:10.2573151384):3.1692362079):4.516005225,101:17.942556574810002):0.44852410825,102:18.391080678599998):9.561443921,((((((105:3.149857079,104:3.149857079):8.510319034,103:11.6601761117):5.165328907,(((((106:8.725508826,107:8.72550882639):4.195827715,((((109:0.1855466782,110:0.1855466782):6.4921006576,108:6.6776473350000005):2.046184195,(111:4.525032833,112:4.5250328328):4.1987986974):1.07014061,113:9.793972140400001):3.1273644014):0.9049631582,(115:2.625564635,114:2.625564635):11.200735065):0.8694264569,(117:5.414456737,116:5.4144567369):9.281269419900001):1.152393502,((121:9.0891114905,(120:1.711835166,119:1.711835166):7.377276323000001):6.682052500568999,118:15.771163991):0.0769556684):0.9773853615):0.5934154592,122:17.4189204838):1.62677338,(123:15.375699568000002,(124:7.598687657499999,125:7.59868765819):7.7770119086000005):3.6699942916999997):0.2248189949,126:19.27051285447):8.6820117462):0.7859497994,127:28.738474398):0.1588882551,(129:7.5843658317,128:7.5843658314):21.312996818360002):11.03961451,(((89:25.413949004000003,(90:7.133194175,91:7.133194175000001):18.28075483):4.978160578,88:30.392109583529997):3.137567781,92:33.529677363):6.407299797):1.972716491,130:41.909693653000005):1.351631578,(131:27.003313730000002,132:27.003313721):16.2580115):2.200769729,((((((143:8.044132529,144:8.044132529):14.32378261,((((((149:5.91913875,150:5.91913875):1.6413617885999998,((153:0.5075869863,154:0.5075869863):3.982573848,(152:2.264433694597,151:2.2644336948):2.22572714):3.070339703):0.6834373402,(((156:4.660226237,155:4.660226237):2.214495412,157:6.874721649):0.9050554897,158:7.7797771375):0.4641607403):2.354929745,159:10.598867623999999):5.337651622,(((146:11.498529631970001,145:11.498529631):2.037556358,160:13.536085989):1.594005703,(148:13.3946898223,147:13.3946898226):1.73540187063):0.8064275527):1.201043193,161:17.137562437):5.230352699999999):2.141372311,((162:16.3469114731,163:16.346911476):4.866378688,((((((164:5.464028321,(166:0.2631921469,165:0.2631921469):5.200836174):0.9819891977,(168:1.862901886,167:1.862901886):4.583115633):1.026499647,(169:3.031154109,170:3.0311541093):4.441363057):0.5258821417,171:7.998399307):2.039689611,(172:4.428688479,173:4.428688479):5.609400439700001):0.2890654858,((((((176:3.630336169,174:3.630336169):0.04540151999,175:3.6757376897):1.079423094,177:4.7551607838):0.5624223678,178:5.317583151000001):0.7816568692,179:6.09924002):1.696731868,(181:3.855775071,180:3.855775071):3.940196817):2.531182516):10.8861357549):3.295997288):4.467011225,(((142:13.522720558,(141:4.739956556,(140:2.116170472,139:2.1161704719000003):2.6237860843):8.78276400167):5.428607895,(138:4.89947247098,137:4.899472470999999):14.051855981):3.09387889,((135:3.940497522,(133:2.308543773,134:2.3085437729000002):1.631953749):0.6678660542,136:4.6083635763):17.436843766):6.931091327):0.8253256394,((182:13.1897387779,183:13.189738781):5.447013242,(186:5.8516293119,(184:2.36616824,185:2.3661682399):3.4854610727999997):12.7851227081):11.164872290000002):6.689083006,(187:0.1512419726,188:0.1512419726):36.33946534735):8.971387634500001):1.940668652):10.970205103);

TREE tree_3619 = (((((11:10.74468797,12:10.74468797):12.624258009,((1:19.46954055,2:19.469540547):2.267920363,(3:18.510196262,((4:13.80375021,((5:2.9916304859,6:2.99163048606):7.231106176,((7:7.149640547200001,8:7.14964054705):0.5180265882,9:7.6676671352):2.555069526):3.581013548585):3.321871685,10:17.125621892999998):1.3845743687):3.2272646452):1.6314850678000001):5.646299556,(((16:8.333384093,(15:5.001567792,14:5.0015677921):3.3318163007000003):0.7746503629,13:9.108034456):1.510892401,17:10.6189268576):18.396318676):5.847480378575,18:34.86272591):17.1814062101,((((((((((((24:11.2852193813,23:11.285219382000001):6.7107124350000005,(21:7.139531308,22:7.139531307):10.856400509):1.098609323,(19:7.161473501,20:7.1614735012):11.93306764):2.886171717,(26:14.483877753399998,25:14.483877758):7.4968351028):6.915156649,27:28.89586951):2.78201130987,28:31.677880813999998):5.194291124,(((53:12.96368154,(((60:6.871123225,(61:3.892941328,62:3.892941328):2.978181897):1.042053315,63:7.913176539):0.3355359583,((57:5.068471913,58:5.068471912):2.236329033,59:7.304800945):0.9439115519):4.714969043):2.708761727,(54:14.06807516,(55:7.212525764,56:7.212525764):6.855549397):1.604368106):3.627983021,(64:12.9864881,65:12.986488103000001):6.313938185):17.571745652999997):1.314283939,(((32:18.256732669,31:18.256732673400002):2.7429113537,30:20.999644029000002):3.463817445,29:24.46346147605):13.722994408):0.7979846742,((52:25.5805699,((47:13.977846494000001,46:13.977846493):6.606938716,((48:7.505586622,49:7.505586622):4.940837415,(51:12.2695798,50:12.2695797957):0.1768442412):8.138361171):4.995784693):10.398899922,((((((39:7.75938791,40:7.75938791):2.294126142,41:10.053514051999999):4.049939897,((37:9.145511291,36:9.1455112911):1.015573456,38:10.161084747):3.942369202):1.881115369,42:15.9845693171):1.237470571,((44:11.2686650668,45:11.268665066):4.209587578,43:15.47825264):1.7437872424):1.30155708,((34:6.927564172,33:6.927564172):5.576056026,35:12.503620198):6.019976769):17.455872856):3.0049707261000003):0.8201882207,(66:23.67385037,(((71:7.16694365,72:7.1669436515):0.8374882445,((((76:1.866725153,74:1.866725153):3.986103997,77:5.85282915):0.006982525446,75:5.8598116758):0.7011779465,73:6.560989623):1.443442273):2.530270141,((69:7.188038863609,(68:3.6049195923000004,67:3.604919592):3.5831192712):0.5547957016,70:7.7428345652):2.7918674726):13.139148333000001):16.130778404):4.056305382,(78:38.010904103,((80:4.347584269,79:4.347584269):16.3803189,(((((81:9.775791668,82:9.77579166814):2.074919906,83:11.8507115734):2.239731027,85:14.0904426001):0.9667357646,84:15.057178364):2.026453604,(86:8.210489405,87:8.210489405):8.8731425638):3.644271196):17.2830009363):5.850030059000001):2.112921365,(((((((((101:18.466460758479997,((((((95:3.643448167,96:3.643448167):3.417816027,97:7.0612641936):1.850584561,98:8.911848755000001):1.9933256094,94:10.905174363099999):0.6219886086,93:11.527162972600001):3.038484512,(99:12.11447486,100:12.11447486):2.451172626):3.900813279):1.269636653,102:19.7360974154):11.002158563730001,((((((((((106:10.75528188,107:10.7552818835):4.958977171,(((111:4.666450347,112:4.666450347):5.227813275,((109:0.2246667586,110:0.2246667586):8.5624100832,108:8.787076842):1.107186781):1.44026606,113:11.3345296822):4.3797293715):1.062616045,(115:1.913731563,114:1.913731563):14.8631435372):1.312821922,(116:7.047668714,117:7.0476687136):11.042028308999999):1.182137122,((120:2.236355234,119:2.2363552345):7.6718708678,121:9.908226103):9.36360804177):0.2340673708,118:19.505901514999998):0.8235374505999999,122:20.329438967):0.1145923309,((105:2.805184322,104:2.805184322):12.3147426,103:15.119926922):5.324104371):1.606937799,(123:16.782016626900003,(124:9.6942728058,125:9.694272805699999):7.0877438209):5.2689524677):0.8427663269,126:22.893735422):7.8445205585):3.260990356,127:33.999246344979994):1.407215314,(129:11.757728487300001,128:11.757728487000001):23.6487331628):3.905299974,((((91:4.881822313,90:4.8818223127):16.058684823,89:20.940507139):7.767829013,88:28.708336149100003):3.659332317,92:32.367668463):6.944093163):3.268277249,130:42.5800388746):2.280181822,(131:34.26780013,132:34.267800125):10.592420573):0.05417694203,((((((143:6.741286202,144:6.7412862019999995):20.14522688,((((160:17.042071586,(148:16.109988509,147:16.10998850863):0.9320830767000001):1.626465422,((((153:0.982189782,154:0.9821897820000001):4.608600988,(151:1.883808472,152:1.8838084715):3.706982298):3.619514732,((((156:3.878353766,155:3.878353766):2.182071071,157:6.0604248386999995):1.407289447,158:7.46771428406):0.5272255956,(150:5.025881222,149:5.025881222):2.9690586586):1.215365621):1.974928869,159:11.1852343704):7.483302637):0.6005882995,(146:12.263722444799999,145:12.2637224407):7.005402861):1.350865261,161:20.6199905673):6.266522519):0.9872641228,((162:17.1754267202,163:17.1754267232):8.821431261,(((171:11.37559253,(((164:6.531837205,(166:0.2634943181,165:0.2634943181):6.268342886999999):2.6384925,(168:3.620250811,167:3.620250811):5.550078894):1.563049463,(170:2.540643169,169:2.5406431686):8.192736):0.6422133571):1.056408817,(172:4.801864173,173:4.801864173):7.63013717):0.6028532026,((180:4.194601441,181:4.194601441):3.027805936,(((((174:3.131327654,175:3.131327654):0.2254149876,176:3.3567426415):0.9377572547,177:4.29449989643):1.26238081,178:5.55688070582):0.9893910824,179:6.5462717881):0.6761355898):5.812447168):12.9620034388489):1.87691923):4.593605754,(((135:4.290597671,(133:2.517302937,134:2.517302937):1.7732947334):0.7250686977,136:5.015666369):20.557503343,((137:6.726211432,138:6.72621143175):13.861250767500001,(142:11.824635892200002,((140:3.656830256,139:3.656830256):2.32257523,141:5.97940548675):5.845230407):8.762826306200001):4.985707513):6.894213258):1.075097659,((182:16.0682647773,183:16.0682647725):5.073538709,(186:6.846523587,(185:2.688215303,184:2.6882153029):4.15830828402):14.295279901948):12.400677144):4.412941264,(188:0.1690493713,187:0.1690493713):37.786372515900005):6.958975749):1.059457881):6.0702765999);

TREE tree_7207 = ((18:34.9671638893,(((11:10.63523194,12:10.63523194):14.760594407,((1:22.92208693,2:22.922086931):0.6416460821,(((4:14.345805447,((6:3.215739136,5:3.215739136):7.630894243,((7:5.587472550399999,8:5.587472550199999):2.850465238,9:8.4379377891):2.408695591):3.4991720708):2.441967109,10:16.787772558):2.077337974,3:18.8651105362):4.698622479):1.8320933318000001):5.934265712,((((14:5.573600602,15:5.573600602):3.079473854,16:8.653074455):1.7596318226999998,13:10.4127062774):1.632557976,17:12.0452642542):19.284827809):3.6370718330000003):19.2475119809,((((((((((44:7.15933876,45:7.1593387598):2.457937736,43:9.61727649529):2.976066271,((((36:7.1931685496,37:7.19316855):0.9534282106,38:8.146596760000001):2.098312441,((39:5.507505948,40:5.5075059490000005):2.985107933,41:8.4926138814):1.75229532):0.9321863393,42:11.177095542):1.4162472262):1.325729025,(35:7.799095916400001,(33:4.668881135,34:4.668881135):3.130214781):6.119975876):15.3200118335,(((((49:5.898049887,48:5.8980498873):4.595282317,51:10.4933322):0.06539898915,50:10.55873119302):4.059881125,(46:11.353338882000001,47:11.353338882):3.265273437):4.876172836,52:19.4947851573):9.744298472):2.2868626934632,((30:17.766993999,(32:15.424352600199999,31:15.4243526004):2.3426413959):3.6899139099999996,29:21.4569079016):10.069038414000001):4.182118291,(((((((19:4.925394829,20:4.9253948286):13.4960057,(21:7.345219358,22:7.345219358):11.076181167):0.03705185999,(24:9.372919408,23:9.372919408):9.085532977):3.005396943,(26:13.7682060669,25:13.768206068):7.6956432614):6.891632985,27:28.355482314):2.9669587495000003,28:31.322441063):3.003015827,((64:8.784205913,65:8.784205912000001):3.934390364,((54:8.420986229,(55:4.21911288509,56:4.2191128849999995):4.201873344):0.3807830899,(53:7.861433483,(63:6.040134499,(((57:4.051674145,58:4.051674145):0.4660220075,59:4.5176961533):0.6731068362,(60:5.184164674,(61:2.352607906,62:2.3526079059):2.831556768):0.006638315115):0.84933151):1.821298983):0.9403358361):3.916826958):21.606860613099997):1.382607716):4.061483073,(66:33.18006936,((((70:11.58496477,((68:4.461090283,67:4.461090283):5.048934752,69:9.510025034):2.074939736):0.6281907836,(((77:4.114057202,76:4.114057202):0.9718878883,75:5.0859450901):3.788155927,74:8.874101017000001):3.339054536):0.5085913659,(72:12.2085946,71:12.2085946005):0.5131523196):0.6276430888,73:13.349390007):19.830679351999997):6.58947832):6.38798277,((((((83:14.985510390999998,(81:10.798297484999999,82:10.7982974846):4.1872129101):0.6765298118,85:15.662040207):0.3689847737,84:16.031024979999998):1.456027077,(87:8.472837429,86:8.472837429):9.0142146285):1.518904649,(80:3.447500902,79:3.447500902):15.5584558):19.747452321,78:38.753409033):7.404121421999999):1.551034275,(((((((89:22.692355588,(91:7.323943213000001,90:7.323943212):15.3684123759):2.42769639,88:25.12005197837):3.557928302,92:28.677980279):7.0971849,(((((((100:10.40858465,99:10.40858465):2.247291837,(((((95:2.797551366,96:2.797551366):2.136767127,97:4.93431849301):0.555063684,98:5.4893821764):4.1827634535,94:9.672145630900001):0.1041367835,93:9.776282414699999):2.879594074):3.877023339,101:16.5328998247):0.6014082,102:17.1343080265):6.843796884,((((((105:2.190001041,104:2.190001041):8.206729969,103:10.3967310097):2.4046544613,((((((106:7.351886315,107:7.3518863152):2.4866011877,(((108:5.345144665,(109:0.1140919096,110:0.1140919096):5.2310527558):1.140901967,(111:3.48028388,112:3.4802838799):3.005762752):0.4750569014,113:6.96110353284):2.8773839686000002):0.5938708107,(115:1.221819046,114:1.221819046):9.2105392677):0.2767443962,(117:4.49191990941,116:4.4919199097):6.2171828):1.323893104,118:12.0329958138):0.3741357884,(121:6.222620213500001,(119:1.443664163,120:1.443664163):4.7789560493):6.184511389):0.39425386873):0.4716483036,122:13.273033770000001):0.9804857666,((125:6.7643588934,124:6.764358893440001):5.552819195300001,123:12.3171780885):1.9363414518000002):0.5797277027,126:14.833247244):9.1448576673):1.725232307,127:25.703337223000002):0.7572213985,(129:7.401344002,128:7.401344002):19.05921461778):9.314606562):10.13021256,130:45.905377740999995):1.296614769,(131:27.235441209999998,132:27.235441212999998):19.966551297000002):0.05338666861,((((((143:8.317387424,144:8.317387424):18.34368677,(((145:12.385724552,146:12.385724554523001):5.705560748,((((((155:5.232490923,156:5.232490923):2.050582278,157:7.2830732010000006):1.616688506,158:8.8997617072):0.2804003137,(((152:2.1166025549,151:2.1166025547):5.05301829,(154:0.5162975254,153:0.5162975254):6.653323319):1.248413341,(149:5.559917319,150:5.559917318):2.8581168669999997):0.7621278336):1.190883413,159:10.371045432999999):6.606793805,((148:15.3554906651,147:15.3554906657):0.98125921106,160:16.336749878):0.6410893608):1.113446065):3.970465957,161:22.061751259999998):4.599322939):0.9533213117,((162:18.980292293,163:18.9802922925):1.125139557,((((((164:6.835731189,(165:0.4446598884,166:0.4446598884):6.391071300999999):1.630305593,(167:2.04080858,168:2.04080858):6.4252282019999996):0.2583615303,(170:2.133658756,169:2.133658756):6.590739557238):1.273325089,171:9.997723402):2.386537102,(173:3.652327497,172:3.652327497):8.731933007):0.4690516563,((179:7.758905006,((((174:5.928564841,176:5.928564841):0.4391561956,175:6.367721037000001):0.8493628982,177:7.217083935):0.2579522387,178:7.4750361729999995):0.2838688319):0.9568212722,(180:6.296100955,181:6.2961009549):2.419625323):4.137585882):7.2521196876143):7.50896366):3.831690176,((((134:1.82513007,133:1.82513007):1.55069433,135:3.3758244):1.180399694,136:4.556224094):22.0623674637,((137:6.31744301,138:6.31744300967):15.4514410452,((141:10.12695684,(140:7.05832763,139:7.05832763):3.0686292056999998):2.950377249,142:13.077334085):8.691549972):4.8497075036):4.82749412):2.106791974,((183:17.275589153,182:17.275589149):7.904986129,(186:6.7867121919999995,(185:3.7559085897999998,184:3.7559085891):3.0308036024000002):18.393863088699998):8.372302382):5.837830446,(188:0.1830778292,187:0.1830778292):39.20763026681799):7.864671073):0.4531855464):6.5061111491);

TREE tree_6165 = (((((11:9.638040449,12:9.638040449):10.919887518,((1:17.05202819,2:17.052028190999998):1.081281998,(((4:13.688900247,((6:3.453870859,5:3.4538708591):8.4800660907,((8:5.8271465514430005,7:5.8271465509):1.536037615,9:7.3631841668):4.5707527834):1.7549632977):1.078564862,10:14.767465113):0.3245884336,3:15.0920535433):3.0412566458000003):2.424617778):4.485211382,(17:9.3584467861,(16:8.316359493,((14:4.301283651,15:4.301283651):3.079646053,13:7.380929705000001):0.935429788):1.0420872928):15.6846925637):3.809241151,18:28.8523804993):23.8013666668,(((((((28:30.040909173999996,(((((21:6.703048379,22:6.703048378):8.824435563,(24:9.984514131000001,23:9.984514131000001):5.542969810000001):1.534778886,(19:5.181234181,20:5.181234181200001):11.88102865):3.166430908,(26:11.0064200933,25:11.006420094):9.222273641400001):5.462567287,27:25.691261022):4.3496481506):4.519768367,((52:20.8216492841,((46:11.544651154199999,47:11.544651154):4.193265911,((50:10.620384678,51:10.620384677):0.06753696369,(49:7.057942881,48:7.0579428805):3.62997876):5.049995424):5.0837322196):10.966388516999999,((((((39:5.688076932,40:5.688076932):1.99632019,41:7.6843971225):2.364858514,((36:6.0208753375,37:6.0208753374):0.9434824702,38:6.964357808):3.084897828):0.3381785075,42:10.387434145):3.07172618,((44:10.161419518999999,45:10.161419519999999):2.568133271,43:12.729552791):0.7296075328):1.146152276,((34:4.680117933,33:4.680117933):3.278326671,35:7.958444603499999):6.646867996):17.182725202):2.7726397387754):1.111882502,(29:22.98423019,((31:16.128021577400002,32:16.128021580400002):2.55285684383,30:18.680878424):4.30335176746):12.688329851):1.073917793,((65:14.81401601,64:14.814016012):3.464774382,((((57:6.693172802,58:6.693172801999999):3.762466538,(((60:7.071567808,(61:4.467357068,62:4.467357068):2.60421074):0.3370717932,63:7.408639601):2.151593201,59:9.560232802):0.8954065374):2.346601214,53:12.80224055):1.410117932,(54:13.84250422,(55:7.784884716,56:7.784884715):6.0576195054):0.3698542653):4.066431907):18.467687439):4.4000903835,(66:26.11533889,(((71:13.547673905,72:13.547673906):2.25238566,((((76:0.1755361443,75:0.1755361443):6.282510806,74:6.458046951):2.335672207,77:8.793719157):4.037415184,((69:10.465641521,(68:4.327040371,67:4.3270403716):6.138601148599999):1.259523627,70:11.725165146):1.105969194):2.9689252253):4.192616464,73:19.992676026999998):6.1226628602889):15.031229325000002):4.196361784,(((((((82:12.09885391871,81:12.098853922):2.3838017247,83:14.482655646000001):2.12178097,84:16.6044366148):0.4447184808,85:17.049155096):1.620123924,(86:9.1306095,87:9.1306095):9.538669518799999):2.235673092,(80:2.895693405,79:2.895693405):18.00925871):20.59303406,78:41.497986171):3.8449438287):1.774628208,(((((((((((100:7.532934294,99:7.532934294):2.895043543,((93:7.4551146704,94:7.4551146710000005):0.9184074345,(((95:2.260666855,96:2.260666855):2.129019927,97:4.389686783):0.8733062415,98:5.2629930241):3.110529081):2.054455732):3.2441384192,101:13.672116256999999):0.5730317387,102:14.24514799494):10.506661065,((((((105:2.019693022,104:2.019693022):10.3488689,103:12.368561922000001):2.755138943,((((((106:8.7985732208,107:8.7985732214):2.803387733,((((109:0.5512030805,110:0.5512030805):6.212213548,108:6.763416629):0.6644553029,(111:4.028195058,112:4.0281950577):3.399676874):1.484612935,113:8.9124848667):2.689476088):0.5247607653,(115:1.999236095,114:1.9992360944):10.1274856249):0.7443958129,(116:6.7355290463,117:6.735529046):6.1355884861):1.328747206,118:14.199864739999999):0.4019775692,((120:1.909278631,119:1.9092786314999999):5.346988415799999,121:7.2562670471):7.3455752608000004):0.5218585567):0.2004570843,122:15.3241579455):1.154861603,((125:8.4902548261,124:8.4902548257):4.737454845449999,123:13.227709672189999):3.251309881):0.1991382053,126:16.678157757):8.073651301830001):1.556282953,127:26.308092009):0.4229723139,(128:7.5732010698,129:7.5732010691):19.1578632505):10.1495168,(((89:24.048174845,(90:6.472726756,91:6.472726755):17.575448095):0.6369951028,88:24.68516995089):4.958445806,92:29.64361575503):7.236965373):8.273513373,130:45.154094500999996):0.9097648989,(131:32.68510619,132:32.685106188000006):13.378753209):0.373828997,((((((138:6.3299660942,137:6.329966095):14.4805059722,((141:7.223053417999999,(140:3.862150767,139:3.862150767):3.3609026503):3.833689094,142:11.05674251):9.753729557):2.8717051152999997,(((134:2.624742279,133:2.624742279):1.724374862,135:4.349117141):1.436450184,136:5.785567325):17.8966098622):9.151505897,(((143:9.20628152,144:9.206281520000001):19.99018095,(((((((151:2.271601467,152:2.2716014668):3.077769547,(153:0.2453782093,154:0.2453782093):5.103992804):2.797330538,(149:6.604777408,150:6.604777408):1.5419241433):0.6868402872,(((156:3.838910085,155:3.838910085):3.802770775,157:7.641680861):0.492952463,158:8.1346333228):0.6989085147):2.819020227,159:11.652562065):7.321038715,((146:13.1974749729,145:13.197474978):4.646149323,(160:16.978563693999998,(148:15.337106582,147:15.337106581):1.64145711263):0.8650606023):1.129976484):2.312010999,161:21.285611779):7.9108506940000005):1.443800556,((162:17.796596612,163:17.796596613):7.755410056,(((171:9.73958487,(((164:6.807177795,(165:0.7648590506,166:0.7648590506):6.042318745):2.121275268,(168:2.162997563,167:2.1629975625):6.7654555):0.1872651604,(169:1.518217268,170:1.518217268):7.5975009551):0.6238666463):2.121115492,(172:5.789310377,173:5.789310378):6.0713899844):1.297835501,((180:6.102289536,181:6.102289536):2.090245297,(179:6.623260469,((((174:2.698363863,177:2.6983638631):1.089059905,175:3.787423767):0.7845682793,176:4.571992047):0.870318483,178:5.44231053):1.18094994):1.569274364):4.9660010304):12.393470804584402):5.088256359):2.193420059):3.174619041,((183:14.72432182,182:14.724321821):7.941906606,(186:7.02065884,(185:2.483800127,184:2.4838001263000002):4.5368587132):15.645569586999999):13.342073692):4.142930446,(188:0.7566774726,187:0.7566774726):39.39455510400379):6.286455824):0.6798698128):5.5361889592);

TREE tree_1211 = ((18:38.8534260407528,((((1:24.62193282,2:24.621932819):0.6071645121,(((4:18.498948206,((9:8.2154942203,(8:6.981635629000001,7:6.981635629):1.233858591):3.284837826,(5:4.273983001,6:4.2739830005999995):7.226349045):6.998616158999999):2.36116684,10:20.860115044900002):0.4422562043,3:21.302371249):3.9267260859):3.318776766,(11:14.2010342,12:14.2010342):14.346839904):5.848567206,((((14:7.733112438,15:7.733112438):4.300312937,13:12.033425375):0.385577971,16:12.41900335):0.1708903392,17:12.589893684700002):21.8065476263):4.456984733):16.261175708,((((((((64:10.00533125,65:10.005331249):1.684270826,((54:7.229253125,(55:4.940669996,56:4.9406699962000005):2.288583129):1.079691685,((((63:6.139173291,((61:2.245466263,62:2.2454662638):3.711948911,60:5.957415175):0.1817581167):0.2198113481,59:6.358984639):0.01570125359,(58:4.4649475022,57:4.4649475027):1.909738391):1.071085767,53:7.44577166):0.8631731498):3.380657265):8.406006434,((52:13.016885433899999,((47:7.6794915,46:7.679491501):1.723067694,((49:4.626676103,48:4.6266761024):2.350577417,(50:6.1666476561,51:6.166647657):0.8106058632):2.425305675):3.6143262399999996):5.6258360849999995,(((34:4.048370709,33:4.0483707086):2.378318912,35:6.4266896209999995):4.471765173,(((((40:4.206741901,39:4.206741901):1.509325147,41:5.716067047999999):2.335806018,((37:4.7536844276,36:4.7536844267000005):1.216703243,38:5.97038767):2.081485396):0.87252651,42:8.924399575999999):0.6607657507,((44:6.279354856885,45:6.279354855699999):1.988368053,43:8.267722909):1.3174424181):1.313289467):7.744266724799999):1.4528869913):16.086304546,((((((24:11.1647041166,23:11.164704116):7.9336326319,(21:7.454938097,22:7.454938096999999):11.643398650999998):0.3232499202,(19:5.34685128,20:5.3468512798):14.07473539):2.399787673,(25:12.443149638700001,26:12.443149639000001):9.3782247021):4.964526323,27:26.785900662):2.663579267,28:29.449479931):6.7324331191):1.401868053,(((31:18.3194049057,32:18.319404906):3.0191899383,30:21.338594844):2.571204024,29:23.9097988689):13.673982241000001):4.300075604,(66:29.3475728,((((71:9.142103599,72:9.142103598):0.7408930043,(((76:1.822617126,74:1.822617126):6.396881849,77:8.219498975):0.7985898795,75:9.018088854):0.8649077486):1.830631503,73:11.713628106):0.642373563,(((68:4.304792606,67:4.304792605599999):5.670096408999999,69:9.974889015):1.117823145,70:11.09271216):1.2632895092):16.99157113):12.53628391):3.903315611,(((80:3.044129901,79:3.044129901):18.72635159,(((85:16.050294092,84:16.050294092999998):0.1079251499,(83:12.52469135,(82:11.795870065999999,81:11.795870071):0.7288212875):3.6335278886999998):0.3527757554,(86:10.13125425,87:10.13125425):6.379740744999999):5.2594864889999995):18.809470042,78:40.5799515204):5.207220791299999):3.753913712,(((((((((((((((97:5.942700162,(95:4.135846811,96:4.1358468109999995):1.80685335095):1.008175186,98:6.950875348499999):2.966734701,93:9.917610048670001):0.1494351424,94:10.067045192):4.4708121946,99:14.537857384499999):1.441536586,100:15.97939397):3.2865406249999998,101:19.2659346003):0.725794222,102:19.9917288201):9.852722601,(126:22.744996374,(((((105:3.307803563,104:3.307803563):12.40565587,103:15.713459431):4.36699739,((((((106:10.8844903297,107:10.8844903285):5.33599539,(((108:8.426000673,(109:0.3622400271,110:0.3622400271):8.0637606457):1.130261485,(111:5.141906648,112:5.141906647):4.41435551):3.565621229,113:13.121883387):3.0986023310000004):0.8661925229,(114:3.206103807,115:3.206103807):13.880574434):0.4878585089,(117:8.117658324,116:8.1176583238):9.456878426):1.521306894,118:19.095843644):0.5757124653,((119:2.161138071,120:2.161138071):9.476090594999999,121:11.6372286666):8.0343274419):0.4089007157):0.6554985778,122:20.735955398999998):0.9063837027,(123:16.622994414700003,(124:10.3534584061,125:10.3534584063):6.269536008139999):5.0193446903800005):1.1026572682):7.099455047):5.086011528,127:34.930462954):1.097009323,(129:10.9489831915,128:10.9489831949):25.0784890785):8.146676275,((((91:8.673057242999999,90:8.673057243):22.076382479,89:30.74943971):0.9584461069,88:31.7078858295):3.918985726,92:35.62687155417):8.547276988):2.997935757,130:47.172084298):1.47558151,(131:36.26682463,132:36.266824627999995):12.380841185000001):0.8025960483,((((((143:8.817623863,144:8.817623863):18.09943532,((((146:13.2486851233,145:13.248685128):3.171205494,160:16.419890617):0.314069591,((((((151:1.1549813974,152:1.1549813974):4.105975581,(153:0.297617955,154:0.297617955):4.963339023):3.428038507,(((155:2.810521087,156:2.8105210863999996):3.50765262,157:6.3181737076):1.221612335,158:7.539786042699999):1.149209443):0.3242974026,(149:5.85917053,150:5.85917053):3.1541223579):2.926451183,159:11.939744071):3.489116523,(148:13.873296314200001,147:13.873296314200001):1.5555642795):1.3050996138):3.933882332,161:20.66784254):6.249216646):0.3147740637,((162:24.810424344500003,163:24.810424341999997):0.5908061156,((((((164:6.910951153,(165:0.5066181108,166:0.5066181108):6.404333041):1.48086838,(167:2.388902239,168:2.388902239):6.0029172941):2.367445581,(169:1.541101722,170:1.5411017223):9.218163392000001):0.9240096388,171:11.6832747511):2.498068299,(173:3.986555799,172:3.9865558):10.194787253000001):0.09017692763,((179:9.366404311,((((174:4.587877764,175:4.587877764):0.8415173079,176:5.429395072):0.89718558,177:6.3265806520000005):1.254189239,178:7.58076989):1.78563442):1.239660086,(181:5.384049261,180:5.384049261):5.222015135):3.6654555837):11.1297104799):1.830602792):2.366254552,(((138:6.7895594382799995,137:6.78955943821):17.768923631,(142:13.564937220000001,(141:8.832460881,(140:5.594354938,139:5.5943549388):3.2381059420000002):4.73247633794):10.99354585):4.80856936397,(((133:2.639279359,134:2.6392793592):0.9248966826,135:3.564176042):0.9428577445,136:4.5070337858):24.860018647999997):0.2310353672):6.606776444,((183:15.926157036000001,182:15.92615704):9.397870344,(186:9.774709569,(185:5.307703216,184:5.3077032161):4.4670063539):15.549317813799998):10.880836867):3.139122181,(188:0.3490952098,187:0.3490952098):38.9948912161571):10.106275432):0.09082417455):5.573515709);

TREE tree_5992 = ((18:25.5072757616442,((((1:14.02786952,2:14.027869515999999):1.161850519,(3:12.957897366,((4:9.037855065999999,((6:1.190230668,5:1.1902306675):6.276172538,((8:4.45173806732,7:4.4517380675):1.468187476,9:5.919925543):1.5464776622):1.5714518606999999):2.945989834,10:11.983844900000001):0.9740524657):2.231822669):2.3252550522,(11:8.317239403,12:8.317239403):9.197735684000001):4.811532087,((16:7.339943256,((14:3.728153507,15:3.728153507):2.6252961712,13:6.353449679500001):0.9864935769000001):0.2764522867,17:7.6163955418):14.710111632199999):3.18076858656):25.151381068299997,((((66:27.24876315,(((((74:7.43968224,75:7.439682240000001):0.9299231341,77:8.369605373999999):1.245773717,76:9.615379091):1.412657616,((71:8.809494125,72:8.809494124):0.2596105885,73:9.069104713):1.958931994):2.372922551,(70:11.91302717,((68:6.2646553647,67:6.2646553652):2.5864495084000003,69:8.851104872499999):3.061922296):1.48793208908):13.8478038923):7.052640262,((((((((24:6.2040383364,23:6.2040383374):6.367645233,(21:4.486378919,22:4.486378919):8.0853046506559):1.458997939,(19:4.889490871,20:4.8894908708600004):9.141190638):2.098135227,(26:9.425448563,25:9.425448562):6.703368174):4.541078052,27:20.6698947903):2.64323556442,28:23.313130353000002):5.447686867,(((31:13.8119605557,32:13.811960555999999):1.84444922479,30:15.656409781):2.213087484,29:17.8694972629):10.8913199564):0.7733284217,(((52:12.70746102881,((47:7.4796700128,46:7.4796700141):1.57921082,((51:6.764075607,50:6.7640756059000005):0.0808493398,(48:3.994670282,49:3.994670282):2.850254664):2.213955888):3.648580199):6.035247411,(((34:2.6916833656,33:2.691683366):3.017055532,35:5.7087388973):3.816073925,(((45:5.9193957559,44:5.919395755):2.062197178,43:7.981592933):0.6677640163,((((36:4.6412797539,37:4.641279753):0.7481238507,38:5.389403604):1.532571236,((39:3.607837719,40:3.607837719):2.333996362,41:5.9418340809):0.9801407593):0.4511762384,42:7.373151077999999):1.2762058707000001):0.8754558725):9.2178956207):1.3339542168,((65:9.983889365000001,64:9.983889365):2.860426993,(((((57:3.755137219,58:3.755137219):2.144435852,59:5.899573071):0.1931885594,((63:3.979909455,60:3.979909455):0.891618966,(61:2.970862702,62:2.9708627019):1.900665719):1.221233209):1.553271236,53:7.6460328660000005):1.094362652,(54:8.472127498,(56:5.589296088,55:5.5892960883):2.8828314099999997):0.2682680205):4.103920839):7.232346302799999):9.4574829828):4.767257772):5.50327485,((((((83:11.5262938743,(82:9.43716029014,81:9.437160291):2.0891335812299996):1.569734041,85:13.096027913):0.08802898563,84:13.1840568981):0.3921812006,(87:6.488107767000001,86:6.488107766900001):7.0881303325):3.93843223,(79:2.201153433,80:2.201153433):15.313516890999999):16.8943267543,78:34.408997085):5.395681177):4.070344319,(((((((89:25.91638893,(90:7.807392816099999,91:7.807392815):18.1089961178):1.294566625,88:27.210955554399998):3.742488843,92:30.953444396000002):4.126540943,(((((101:17.0531871867,((99:10.7528501,100:10.7528501):1.317778068,((93:8.201855351699999,(((95:3.309133754,96:3.3091337541000003):1.348293095,97:4.65742684842):0.9996519602,98:5.6570788089):2.5447765428999998):0.2382474591,94:8.4401028108):3.630525358):4.98255902):0.2056909039,102:17.258878093):8.9535916981,((((((((((106:9.429469437,107:9.4294694371):3.446158064,(113:9.966210395200001,((111:4.476730516,112:4.4767305159):4.040070253,(108:6.0614395177,(109:0.2495493724,110:0.2495493724):5.8118901443899995):2.455361252):1.4494096251):2.9094171059000002):1.319565853,(115:2.608005786,114:2.608005786):11.587187568500001):0.7310486388,(116:6.1739810774,117:6.1739810761000005):8.752260916000001):0.4111736316,118:15.337415624):0.1231804527,(121:7.547391612999999,(120:1.110861526,119:1.110861526):6.4365300867):7.913204464230001):1.1404866243,((105:2.980806954,104:2.980806954):9.9487072468,103:12.929514201):3.6715685005000003):0.09986234998,122:16.700945055):1.439359582,((125:8.9687979827,124:8.968797984):5.31394847686,123:14.282746460199998):3.8575581727):0.7926742014,126:18.9329788336):7.2794909552):1.36977106,127:27.582240854000002):0.985547884,(128:8.2319261813,129:8.2319261814):20.335862558928):6.512196604):7.039632794,130:42.1196181446):0.9149291047,(131:25.291076812999997,132:25.291076816999997):17.743470418999998):0.5611429989,((((((143:7.29243468,144:7.292434681):20.3349433,(((((((156:5.116498665,155:5.116498665):3.97193611,157:9.088434775):0.4448985134,158:9.533333289089999):0.5069296306,(((153:0.6891602528,154:0.6891602528):4.11186984,(151:2.375884243,152:2.3758842431):2.425145849):3.143305233,(149:6.967830873,150:6.9678308730000005):0.9765044527310001):2.0959275937):1.435444671,159:11.475707590999999):7.789674991,(((146:13.803293356,145:13.803293354000001):3.602589101,160:17.405882454):0.9903145668,(148:17.039488603,147:17.039488601389998):1.3567084222):0.8691855565):1.502829525,161:20.7682121083):6.859165872):0.5490411517,((162:24.334424461,163:24.334424458):1.147217468,(((171:12.58436401,(((164:6.442017997,(165:1.351910101,166:1.351910101):5.090107896):2.852666955,(167:3.072035416,168:3.072035416):6.2226495360000005):2.662683179,(170:2.443936686,169:2.4439366865):9.513431445000002):0.6269958836):2.003546273,(172:3.006299575,173:3.006299575):11.581610712):1.329222377,(((((176:6.916708216,175:6.916708216):0.03733615524,(174:6.179003388,177:6.179003388):0.7750409837):0.6174109139,178:7.571455285):0.8959344505,179:8.467389736):0.4989097292,(180:4.764807906,181:4.764807906):4.2014915587):6.9508331988):9.5645092711613):2.694777191):1.786509411,(((137:5.348280628,138:5.3482806278999995):13.084377074499999,(((140:5.1695810823,139:5.169581082500001):2.2937296152,141:7.463310697000001):5.792106217,142:13.255416918):5.177240788):2.8765583502,((135:3.611194499,(133:2.139218544,134:2.1392185447000003):1.471975954):0.7935742946,136:4.404768793200001):16.9044472583):8.653712488):0.7452847835,((183:16.33526409,182:16.3352640899):5.443139897,(186:7.433420452,(184:2.57092353,185:2.57092353):4.8624969215):14.3449835344):8.929809333900002):4.971934934,(187:0.183440099,188:0.18344009906):35.496708161300006):7.9155419851):0.2793323439):6.7836342485);

TREE tree_4738 = ((18:36.862843333399994,(((11:13.18057969,12:13.18057969):11.336159921,((1:21.62013993,2:21.620139930999997):0.6276489742,(((4:14.569166238,((6:3.719610575,5:3.719610576):8.745850296,((8:7.312696644900001,7:7.312696645999999):0.7579150451,9:8.0706116907):4.3948491804):2.1037053669):2.217147534,10:16.786313777):1.385586413,3:18.171900185):4.075888723):2.2689506996):6.012241782,((((14:6.566544913,15:6.5665449128999995):4.0087698851999995,13:10.575314798):0.5726053762,16:11.14792017):0.7093512515,17:11.8572714255):18.6717099681):6.3338619497):21.853061204000003,(((((((((54:7.813188557,(55:5.415171265,56:5.415171265):2.3980172916):0.7820822465,(((((61:2.220545401,62:2.2205454004):2.221147815,60:4.441693216):0.08588243408,63:4.52757565):1.514521079,((57:3.992637317,58:3.992637317):1.922448933,59:5.9150862504):0.1270104788):2.005494629,53:8.0475913575):0.5476794461):3.484919827,(64:9.216958849,65:9.21695884854):2.863231782):26.716582610800003,((((((21:7.401472295,22:7.401472295):10.7548019979,(24:10.5284114734,23:10.528411474):7.6278628181000006):0.3923587339,(19:5.095461613,20:5.0954616127):13.45317141):5.43645548,(25:14.571864487860001,26:14.571864488700001):9.413224018):5.757228742,27:29.74231725):0.8721051561,28:30.614422405):8.182350835200001):2.338198653,((30:19.780660476999998,(32:17.453707845,31:17.4537078462):2.3269526360999997):2.85743548,29:22.638095963000005):18.496875935000002):0.05939307873,(((43:14.33921565,(45:12.446549869999998,44:12.446549871):1.892665777):3.202044366,(((34:6.238627136,33:6.238627136):2.736825491,35:8.975452627):7.811836732,((((39:7.315836132,40:7.315836132):2.373536149,41:9.689372281):4.199466554,((36:8.197336728,37:8.197336729):0.4171767872,38:8.614513515999999):5.274325319):0.5622501321,42:14.451088966700002):2.336200391):0.7539706546):16.760374495,(((51:12.55175331,((49:7.506037445,48:7.5060374450000005):3.73224357,50:11.238281016):1.3134722896):4.670256574,(47:13.752517668,46:13.752517671):3.4694922111000004):4.2264513175,52:21.448461196300002):12.853173312):6.892730471):5.600452967,(66:33.03973862,(((((69:13.173223310800001,(68:6.172206835,67:6.172206835):7.001016476):1.637705368,70:14.81092868):2.31954808,((74:1.396659844,76:1.396659844):7.400602602,(77:4.956258974,75:4.9562589739):3.841003472):8.333214313400001):1.188594922,(72:16.209744817999997,71:16.209744813):2.1093268639):1.507484935,73:19.826556617999998):13.213182004299998):13.755079329):5.167225073,(78:43.149051975,(((87:10.06737814,86:10.067378143):12.9047350277,(((83:15.65928222,(82:14.931019419000002,81:14.931019418999998):0.7282628043):3.3909620888000003,85:19.050244312):0.5704815363,84:19.620725849):3.35138732083):2.533602979,(80:3.993932156,79:3.993932156):21.511783989):17.643335827):8.812991044):2.235880442,(((((((((101:18.925840184000002,((99:13.72679028,((93:9.897579931700001,(98:7.479000860699999,(97:5.517881952,(95:4.673104133,96:4.673104133):0.8447778198):1.961118908769):2.418579071161):0.4731494991,94:10.370729432000001):3.356060852):0.6312968939,100:14.35808718):4.567753003):0.2490789083,102:19.174919089045):7.871373741999999,(126:20.266850321,(((((105:3.740002099,104:3.740002099):10.3704967,103:14.110498797):2.807527831,((((((106:8.748593279,107:8.74859327926):3.923732024,((((109:0.488066642,110:0.488066642):6.2770955513999995,108:6.765162194):1.401141504,(111:3.874066496,112:3.8740664965):4.292237201):0.9134909296,113:9.0797946276):3.5925306755):0.328856784,(115:2.496086798,114:2.496086798):10.505095288):1.598249062,(116:5.9510749878,117:5.951074988):8.648356160999999):0.8016437725,118:15.401074921000001):0.2605397188,((120:1.385527924,119:1.385527924):6.8980873041,121:8.2836152281):7.3779994121):1.2564119865999999):0.5024531846,122:17.420479806499998):2.332585115,(123:14.425448298449998,(124:8.469829229,125:8.4698292295):5.95561906968):5.3276166289999995):0.5137853941999999):6.779442509):2.412172239,127:29.458465067000002):1.162328109,(128:9.0045350958,129:9.0045350958):21.6162580839):16.16496505,((((90:7.800363701,91:7.800363701626):25.110298342,88:32.91066204081):0.630624629,89:33.54128667):5.299725496,92:38.841012162):7.944746065):3.00678196,130:49.7925401942):3.215573567,(132:32.778784687000005,131:32.77878469):20.229329059):0.2064358896,((((((138:8.869654260699999,137:8.8696542596):15.530391659,(((140:6.221804124,139:6.2218041234):1.598066702,141:7.819870825301):8.691188126,142:16.511058951):7.8889869664):2.9320623499,(((134:3.052309097,133:3.052309097):1.19879142,135:4.251100517):2.27183794,136:6.522938457):20.8091698072):10.551282,(((143:11.42267169,144:11.422671686000001):22.75839431,(((((146:13.9929632122,145:13.992963212000001):5.228819494,160:19.221782705):1.932077237,(148:18.0579365103,147:18.057936509399998):3.0959234333):1.64083685,(((((155:5.122006105,156:5.1220061050000005):4.35988107,157:9.481887174999999):2.409770748,158:11.891657924099999):0.01128392982,(((154:0.759532587,153:0.759532587):7.26518831,(152:2.159947877,151:2.1599478769):5.86477302):2.720474938,(150:9.361389588,149:9.3613895884):1.383806246):1.157746018):2.666951538,159:14.569893391):8.224803402):3.432059411,161:26.226756203999997):7.954309793):0.04403854374,((162:27.183532091,163:27.183532091):3.345797886,((((((164:6.411590502,(166:0.1019988668,165:0.1019988668):6.309591634999999):3.104682826,(168:3.145709052,167:3.145709052):6.370564276):3.15284773,(169:2.462654309,170:2.4626543093):10.206466749):0.636334361,171:13.305455423):1.701653136,(173:2.451809037,172:2.451809037):12.555299518):0.9605957428,((((((176:4.909511621,175:4.909511621):0.9860689955,174:5.895580617):0.9254964562,177:6.8210770728):0.4061660589,178:7.227243132):1.042039413,179:8.269282545):2.619032218,(181:5.742794564,180:5.742794564):5.145520199):5.079389534000001):14.561625676999999):3.695774559):3.658285735):1.680070011,((183:18.76329271,182:18.763292708999998):7.569161148,(186:7.416465106,(184:2.832844984,185:2.8328449841):4.5836201226):18.915988751):13.231006423):4.100474838,(188:0.2832313894,187:0.2832313894):43.3807037318218):9.550614525):0.9833738148):4.5179810838);

TREE tree_3768 = ((((((1:23.18395098,(3:18.6651724025,((4:15.570553467,((5:5.750644058,6:5.750644058):5.786108164800001,((8:5.948332409,7:5.948332409400001):1.1739592152,9:7.122291624100001):4.414460598700001):4.0338012469999995):2.351627678,10:17.92218115):0.7429912535000001):4.518778585):0.2083633433,2:23.392314329):2.4887566017,(11:11.51958357,12:11.51958357):14.361487357):5.859486477,((((15:4.340990401,14:4.340990401):4.758928328,13:9.099918728999999):1.7935527641,16:10.893471492):1.126891571,17:12.0203630639):19.720194343000003):3.8737892443999997,18:35.6143466509865):17.781433708,((((66:29.0195673,(((((76:5.28521519,(75:0.7866120783,77:0.7866120783):4.4986031116):3.739596308,74:9.024811498):4.956283309,((69:10.0109237255,(68:7.4267810050000005,67:7.4267810049):2.584142721):1.033079965,70:11.04400369):2.9370911150000003):0.01044936921,(72:13.088370535000001,71:13.088370531):0.9031736444):2.527123307,73:16.518667486):12.500899818):10.784519427,((((28:23.228965469,(((((24:7.7026037295,23:7.70260373):6.90995565254,(21:5.224028366,22:5.224028366):9.388531016):0.7527759444,(19:5.201848141,20:5.201848140299999):10.16348719):1.496088682,(26:10.7235603524,25:10.723560351669999):6.1378636574000005):4.531580021,27:21.393004028):1.83596143881):5.779250511,(((31:14.0357123765,32:14.035712377):2.0785282364,30:16.114240613):2.332755039,29:18.4469956515):10.561220328000001):1.558551518,((52:17.2903067996,((46:9.661604052,47:9.661604052):4.553469792,((51:9.7995111869,50:9.7995111867):0.1833100933,(48:6.853367126,49:6.853367126):3.129454153):4.232252564):3.075232957):12.166903219,((((((36:6.03887797,37:6.0388779690000005):0.9834543969,38:7.022332367000001):3.14364169115,((40:3.748811582,39:3.7488115822):2.453561836,41:6.2023734180000005):3.9636006398999997):0.4141968392,42:10.5801708971):1.563932651,((45:8.103707331799999,44:8.103707331999999):3.171644503,43:11.27535183):0.8687517133):0.7391844021,((33:5.538624017,34:5.538624017):1.911113528,35:7.449737545):5.433550405):16.573922072):1.109557481675):1.234009822,((64:8.628096073,65:8.628096074):3.049666028,((54:8.459840786,(55:4.680320848,56:4.6803208478):3.779519939):1.477052894,(53:9.664503991,(((60:2.953580225,63:2.953580225):1.612747166,(61:3.230721059,62:3.230721059):1.335606332):1.832197049,((57:2.900946726,58:2.9009467259):2.666367854,59:5.56731458):0.8312098604):3.265979551):0.2723896888):1.740868422):20.123015219000003):8.003309407):5.80746095,(((80:2.205339078,79:2.2053390789000002):16.3259742,((((82:10.61630755575,81:10.616307554999999):1.7817626877000001,83:12.398070239):2.530603455,(85:13.800572178,84:13.80057218):1.128101521):1.834936738,(86:7.248873545,87:7.248873545):9.514736892399998):1.767702838):22.455197296999998,78:40.986510571):4.625037102):3.725539764,((((((((((100:16.6816631,(99:16.02989093,((93:14.664369869999998,((97:7.623818868,(95:3.718119002,96:3.7181190024):3.905699866):2.0033812835,98:9.6272001512):5.0371697213):0.02755917038,94:14.6919290448):1.33796188398):0.6517721691):6.924285971,101:23.605949062799997):0.694452688,102:24.300401755600003):10.247719785000001,(126:25.142364465,(((((104:3.464057829,105:3.464057829):13.47740684,103:16.9414646649):3.8537523240000002,((((((106:12.26964066,107:12.2696406652):5.018917915,((((109:0.1580126176,110:0.1580126176):8.5036111316,108:8.661623749):1.134726111,(111:4.89195599,112:4.8919559905000005):4.90439387):1.984599582,113:11.780949440899999):5.507609137999999):1.01044934,(114:2.896935531,115:2.896935531):15.402072388):0.5116887442,(116:7.632773517,117:7.632773517):11.177923147):1.352776957,118:20.163473617999998):0.30624699,((119:2.769785544,120:2.769785544):7.5886280631,121:10.3584136076):10.1113070034):0.325496381):0.437891282,122:21.233108272):3.108188454,((124:13.203775248986,125:13.2037752497):5.935807080300001,123:19.1395823291):5.2017143986):0.8010677361999999):9.405757075):1.134972289,127:35.683093834000005):0.102777288,(128:9.53742743,129:9.537427430400001):26.248443690900004):6.756572723,(((89:29.30686005,(90:7.600073436,91:7.6000734363680005):21.706786616):2.917051552,88:32.2239116012):5.482696985,92:37.706608584099996):4.835835253):3.366581464,130:45.909025299999996):1.281910803,(131:32.46235722,132:32.462357215):14.728578888):0.2134943559,((((((143:8.958900872,144:8.958900872000001):18.95412274,((((160:17.6994757017,(148:17.472032748100002,147:17.472032747980002):0.22744295352):1.635466382,(146:14.1290732902,145:14.129073287999999):5.205868792):1.466847309,(((((156:5.368296477,155:5.36829647733):3.513564393,157:8.881860871):2.037427181,(((152:2.4697060705999996,151:2.469706071):4.111472196,(153:0.6143425962,154:0.6143425962):5.96683567):3.704245124,(150:8.081845822,149:8.081845822):2.2035775686):0.6338646605):0.26536844,158:11.1846564911):2.699178179,159:13.88383467):6.917954722):2.747134672,161:23.548924059999997):4.364099551):2.003576896,((162:19.3296200248,163:19.329620018299998):4.936004139,((((((164:7.993940992,(165:1.023307751,166:1.023307751):6.970633241):1.242452844,(168:3.858192881,167:3.858192881):5.3782009548000005):2.571004514,(169:2.798149179,170:2.7981491791):9.00924917):0.9135138442,171:12.720912199):3.662474742,(172:7.678639579,173:7.6786395794999995):8.704747356):0.7496008561,((179:9.724357024,(((176:6.184325608,(175:5.907390373,174:5.907390373):0.2769352351):0.3814051946,177:6.565730802799999):0.7833383955,178:7.3490691980000005):2.375287826):3.075529395,(180:6.514858102,181:6.514858103):6.285028316):4.333101373):7.132636375060001):5.650976344):2.277386071,((183:18.75478011,182:18.754780112):8.161085991,(186:7.0816242504,(185:3.794408495,184:3.794408495):3.2872157547):19.83424184695):5.2781204781):2.715599307,((((134:2.16409857,133:2.16409857):0.9723692768,135:3.136467847):0.3857049663,136:3.5221728129):20.703515481,((((140:4.111374301,139:4.111374301300001):2.2068251335,141:6.318199434):4.3739680073,142:10.692167440999999):7.9366551201,(138:5.0274020660000005,137:5.0274020655000005):13.6014204935):5.5968657319999995):10.6838976):4.264277531,(188:0.3319506371,187:0.3319506371):38.841912784):8.230567046100001):1.932656974):4.0586929154);

TREE tree_8264 = ((18:23.9839788304,((((1:13.79354243,2:13.793542426):0.6256941875,(((4:9.766093407,((6:2.220865495,5:2.220865495):4.7432849487,((8:3.8183521529,7:3.8183521527):1.2405440943556,9:5.05889624811):1.9052541965):2.801942963):2.16849208,10:11.9345854872):0.10806608404000001,3:12.042651572):2.3765850421000003):2.032999029,(11:6.830573337,12:6.830573337):9.621662305000001):3.295309831,((16:6.476077918,((15:4.0799483371,14:4.0799483379999995):2.137190724,13:6.2171390613):0.2589388569):0.8363868068,17:7.3124647257):12.4350807491):4.2364333565):26.430206753,((((((((32:10.782307484,31:10.7823074838):2.3167589621999998,30:13.099066447000002):2.091660205,29:15.190726652299999):11.452952827899999,(((((19:3.980368019,20:3.9803680191):8.426434258,((23:6.681480732,24:6.6814807317):5.5748713751,(21:5.124504501,22:5.1245045010000005):7.13184760606):0.1504501703):3.384915915,(26:9.191089293000001,25:9.191089293800001):6.6006288985):3.181952997,27:18.973671189):2.630449467,28:21.6041206557):5.03955882408):3.372905736,((((((44:5.8342294,45:5.8342294002):1.60749012,43:7.44171952):0.6579185451,((((36:4.7584917901,37:4.7584917899):0.5025641423,38:5.261055932):1.60539463,((40:3.549127864,39:3.549127863):0.9675724353,41:4.5167002992):2.349750263):0.266316243,42:7.132766804099999):0.9668712606000001):0.463148518,((34:3.820502332,33:3.820502332):3.216004086,35:7.036506418):1.526280166):9.371855838999998,(52:13.386722223860001,(((49:3.846502729,48:3.846502729):2.358320916,(51:5.8924082079,50:5.892408207099999):0.3124154371):2.409924938,(46:6.8449029239999994,47:6.8449029239999994):1.769845659):4.771973637):4.547920202):2.4623851687,((65:10.699562364999998,64:10.699562365):2.663446936,((54:10.38362133,(55:3.650998444,56:3.6509984444):6.7326228881):1.99430106,((((57:3.923024469,58:3.9230244684):1.105865309,59:5.028889778):0.09280466986,((63:4.278958105,60:4.278958105):0.178873646,(61:3.228537111,62:3.2285371110000005):1.22929464):0.6638626967):4.782355721,53:9.904050168):2.473872225):0.9850869085):7.034018291960001):9.619557622):5.044609145000001,(66:31.13596434,(73:13.361005079,((72:11.359062349,71:11.359062349400002):0.3463643657,((((68:3.328832395,67:3.3288323947):2.711685444,69:6.0405178398):3.55514938,70:9.595667219):1.662267525,((77:4.915944183,75:4.9159441823000005):4.971849088,(76:2.903981211,74:2.903981211):6.983812059):1.3701414726999999):0.4474919712):1.6555783686):17.774959257):3.925230026):7.740754755,(78:40.014642677,((80:1.429257991,79:1.429257991):21.31707393,(((((81:11.8006479057,82:11.8006479068):3.311413424,83:15.112061328):0.5002328569,85:15.6122941868):0.1500010736,84:15.762295261999999):3.421040443,(87:9.735777269,86:9.735777268):9.4475584355):3.562996221):17.268310749):2.7873064469999997):2.770854746,(((((((89:25.121464326599998,(91:8.447107479,90:8.447107478):16.674356846000002):2.792127779,88:27.913592106963):0.80018531,92:28.713777418000003):10.79826912,(((((101:17.787986222,(100:14.04306549,(99:13.81652983,((93:10.654763953,(98:6.8859524703999995,((95:3.989913439,96:3.9899134391):2.291900521,97:6.281813961):0.6041385096):3.768811482):0.12410049,94:10.778864443300002):3.037665384):0.2265356633):3.7449207309999997):1.304320738,102:19.092306959400002):7.5145069609999995,((((((105:3.113757776,104:3.113757776):11.61146493,103:14.725222704):4.0894205239999994,((((((106:10.76573678,107:10.76573677576):2.492326447,((((109:0.3283379982,110:0.3283379982):6.9341528256,108:7.262490824):0.9792886065,(111:4.439710106,112:4.4397101066):3.802069324):2.548667804,113:10.790447235490001):2.4676159899):1.913138788,(115:1.584127824,114:1.584127824):13.587074188999999):1.689855533,(116:7.656265157,117:7.656265157):9.204792388400001):1.020958624,118:17.882016169):0.3715710887,((120:1.765759561,119:1.765759561):8.996021067600001,121:10.761780627):7.4918066303):0.5610559678):0.1095080389,122:18.9241512655):1.002986361,(123:16.2198351823,(124:9.5231138525,125:9.523113852):6.696721328880001):3.7073024445000002):0.5582006402,126:20.4853382653):6.121475653):3.834141698,127:30.44095562):0.06057571252,(128:8.748365678,129:8.7483656776):21.7531656537):9.010515211):2.848343279,130:42.3603898156):1.985141756,(131:30.985402475,132:30.98540248):13.360129103):0.46294741,((((((143:8.002704701,144:8.002704700999999):22.20426598,(((((146:11.8258505356,145:11.825850535):6.721446665,160:18.547297195):0.5156088541,(147:16.2901935677,148:16.2901935669):2.772712487204):1.140745875,((((((154:0.8886491415,153:0.8886491415):4.971515803,(152:1.8671623221,151:1.8671623216):3.993002623):3.08736703,(150:7.599066449,149:7.5990664500000005):1.348465525):0.5502288006,((156:6.104349267,155:6.104349267):1.270377926,157:7.3747271928):2.1230335824999997):0.367390103,158:9.8651508778):3.235947955,159:13.10109883246):7.102553097):2.378294133,161:22.5819460635):7.625024616999999):0.8581476105,((162:22.966375997,163:22.966375991000003):1.849512375,((((((164:7.726076697,(165:0.4944625451,166:0.4944625451):7.231614152900001):1.226206998,(167:2.931002164,168:2.931002164):6.0212815306):1.708484316,(169:3.72487191,170:3.7248719096):6.935896101):1.365221063,171:12.02598907):2.07764084,(172:5.60303778,173:5.60303778):8.500592136):1.001369741,(((((174:4.996078318,177:4.9960783183):2.043428719,(175:5.664902036,176:5.664902036):1.3746050008):0.3232917173,178:7.362798754):0.7280421757,179:8.09084093):1.809790613,(181:5.660435242,180:5.660435242):4.240196302):5.204368112):9.7108887189):6.249229919):1.991802412,(((135:3.988305833,(133:3.457938635,134:3.457938635):0.5303671974):0.3649644504,136:4.3532702830000005):19.810069121,(((141:7.079015652999999,(139:5.097585477000001,140:5.097585476200001):1.9814301763):5.7646406733,142:12.843656326):8.1067004037,(138:5.9566025975,137:5.956602597):14.993754132000001):3.2129826735):8.893581296):1.481809291,((183:20.227771792,182:20.2277717885):4.694209362,((184:4.4278655744,185:4.427865575):3.440793138,186:7.8686587133):17.053322437):9.616748849):1.79108223,(188:0.1432923094,187:0.1432923094):36.1865199178):8.478666763):0.7643248736):4.841381727);

TREE tree_8017 = ((18:28.3026815898,(((11:8.09245734,12:8.09245734):12.368632136,((1:17.02181688,2:17.021816881):1.619493565,(((((9:5.9488194366,(7:4.852509365,8:4.8525093643699995):1.0963100721):4.922852976044999,(6:2.5711363164,5:2.5711363167):8.300536096):2.185759128,4:13.057431541):1.549346726,10:14.606778268):0.4413386409,3:15.048116908):3.5931935371):1.8197790294):3.745631344,((16:8.714435084,(13:8.078653428299999,(14:4.749017678,15:4.749017678):3.3296357506):0.6357816549):0.51058061,17:9.2250156945):14.9817051264):4.0959607771587):26.095625611000003,((((((((30:15.446017492,(32:13.117317608,31:13.1173176074):2.3286998831):4.2010382907,29:19.647055782):11.269477858,((((((22:5.353575407,21:5.353575407):8.3407849608,(23:7.55965116545,24:7.559651164299):6.134709203):1.679988663,(19:5.86616011,20:5.866160110500001):9.50818892):1.633107457,(25:9.810499549,26:9.810499548):7.196956939700001):5.491885885,27:22.4993423788):1.893216701,28:24.392559075):6.5239745653):2.617735005,(((((((40:4.840976555,39:4.840976555):4.173838647,41:9.014815202000001):2.406072256,((37:6.45277815,36:6.4527781505999995):1.642445372,38:8.095223522):3.325663935):0.6226730986,42:12.043560556):2.40744831956,((45:9.035525000999998,44:9.035525001):2.952450998,43:11.9879760013):2.463032877):1.522146194,((34:5.038937008,33:5.038937008):6.342062008,35:11.380999016):4.592156053):14.961364745000001,(52:19.912370197899996,(((48:6.848421615,49:6.848421615):4.161091933,(51:10.58529896,50:10.585298959):0.4242145883):3.75802916,(47:11.781000653,46:11.781000654):2.986542054):5.144827492999999):11.022149612):2.5997488266863997):0.7967971222,((64:12.83952939,65:12.839529390100001):2.480167958,((53:10.78792002,(((61:3.166909577,62:3.1669095772):2.925337372,(63:5.257561239,60:5.257561239):0.8346857098):0.9617286839,((57:3.813871908,58:3.8138719080000003):2.76588329,59:6.579755198):0.4742204346):3.733944386):0.2475044795,(54:10.62127254,(55:6.191821677,56:6.1918216774):4.4294508664):0.4141519542):4.284272849):19.0113684124):2.6681170642999996,(66:24.56430452,((((71:10.388002871,72:10.388002871000001):1.8523628,73:12.240365671):0.2833826256,(((74:0.3416679608,76:0.3416679608):11.17840574,77:11.520073702000001):0.4165672344,75:11.93664094):0.5871073603):0.4570853033,((69:9.437821102700001,(67:3.974090662,68:3.9740906612):5.463730443):1.203576596,70:10.641397699999999):2.3394359001000002):11.583470919945201):12.434878309):11.03891402,(78:41.11170494899999,((((85:17.0661791052,84:17.066179105):0.1250865296,((82:13.370928192000001,81:13.370928192000001):1.083709867,83:14.454638061099999):2.736627576):0.8625800375,(86:7.9826314410000005,87:7.9826314411):10.071214230799999):3.895432961,(80:2.712300905,79:2.712300905):19.23697773):19.162426318):6.926391897499999):0.6879104749,((((((((90:8.2216980696,91:8.221698069):23.237378491999998,89:31.45907656):1.793162064,88:33.252238624099995):2.195864306,92:35.448102923):7.779940871,(((((101:19.02776529,((99:13.45751427,100:13.45751427):2.019810423,((93:10.815433343900002,(((95:4.25446066,96:4.2544606599999995):1.935191681,97:6.18965234):1.9419424029,98:8.131594742499999):2.68383859953):0.3291190446,94:11.144552387):4.33277230902):3.550440591):1.4071327999,102:20.43489808684):9.687902253,((((124:11.8323969033,125:11.8323969034):5.1971536226,123:17.0295505267):3.1235044764,((((105:2.783076574,104:2.783076574):10.27355251,103:13.05662909):5.259298505,((((((106:9.963137459,107:9.9631374579):3.23056409,(((108:7.115740481,(109:0.1677820547,110:0.1677820547):6.9479584264):2.038546318,(111:5.680483493,112:5.6804834936):3.473803306):1.017765708,113:10.172052508199998):3.0216490416):1.262635287,(115:2.320312338,114:2.3203123382):12.136024498):1.856765068,(117:8.043263631,116:8.043263631):8.2698382721):1.18541023,118:17.498512132400002):0.4204421818,((119:2.038081648,120:2.038081648):7.6961583004000005,121:9.734239948599999):8.184714367):0.39697327497):0.2691955164,122:18.5851231055):1.567931895):0.4482625899,126:20.6013175929):9.521482747799999):2.564466706,127:32.6872670462):1.467805499,(128:8.567101248899998,129:8.567101249199998):25.587971303899998):9.072971253):1.42662569,130:44.654669495):3.05394846,(132:30.497002876999996,131:30.497002878):17.21161507):0.3912964305,((((((143:9.007719646,144:9.007719647):19.80966711,((((160:19.1149957571,(148:15.8544982832,147:15.854498282):3.2604974786):0.3193677685,(146:12.801360732820001,145:12.801360733):6.633002796):0.1873015427,(((((155:5.295553496,156:5.295553496):3.674018649,157:8.969572146):0.8870478089,158:9.856619954):0.2894412094,(((154:0.2943412902,153:0.2943412902):6.281636382,(151:1.519360871,152:1.5193608713):5.056616801750001):2.141373826,(149:7.424001093,150:7.424001093):1.2933504058):1.428709665):3.281895166,159:13.427956329999999):6.193708742):2.362763448,161:21.98442852):6.832958231999999):0.4841033885,((162:24.954474601,163:24.954474604):1.177129368,(((171:9.181536693,(((164:5.559062129,(165:0.3385955557,166:0.3385955557):5.220466573):2.167515003,(167:1.485452558,168:1.485452558):6.2411245740000005):1.314709836,(169:2.106773336,170:2.1067733357000002):6.934513632):0.1402497257):2.772587219,(172:4.752708833,173:4.752708832):7.2014150789):0.7171982009,(((178:5.270751821999999,(174:5.118086688,((176:3.279050412,175:3.279050412):1.365357903,177:4.644408316):0.4736783726):0.1526651338):1.24227065,179:6.513022471):2.04792103,(180:5.815089275,181:5.815089275):2.7458542262999996):4.110378612):13.4602818560432):3.169886171):3.366140892,((((141:8.157390382039999,(140:4.9507996289000005,139:4.9507996289000005):3.20659075248):6.019091766,142:14.1764821475):6.268068400000001,(137:5.7008583680000005,138:5.7008583674):14.743692178):7.5121297047,(((133:1.489073295,134:1.4890732955):3.410886317,135:4.899959612):0.3233436998,136:5.2233033119000005):22.733376940000003):4.710950788):1.844603194,((183:15.387784193,182:15.387784193000002):3.256227376,(186:7.6512201089000005,(184:3.251498433,185:3.2514984333):4.3997216751999995):10.9927914599):15.868222660999999):3.540822144,(187:0.01534446535,188:0.01534446535):38.0377119034835):10.046858005299999):0.6260929467):5.6722998776);

TREE tree_7774 = (((((11:7.708470817,12:7.708470817):11.530371354,((1:17.19289974,2:17.192899736):0.8059668753,(((((6:2.9124770746,5:2.9124770749):7.227604159,(9:6.0970421787,(7:4.734091109,8:4.7340911092599995):1.3629510703):4.0430390549999995):1.827775211,4:11.967856444999999):1.917051544,10:13.88490799):1.595204028,3:15.4801120167):2.5187545939000002):1.2399755606):6.228004195,((((15:4.188129547,14:4.188129547):3.67728208,13:7.8654116268):0.8721432873,16:8.737554914):1.012544093,17:9.7500990067):15.716747355899999):5.647660053,18:31.114506428219702):19.465414833900002,((((66:30.36665185,(((70:12.76283307,(69:9.6999073732,(68:5.407388368,67:5.4073883679999994):4.292519006):3.062925699):0.5588068547,(((76:2.95245319,74:2.95245319):1.845856639,75:4.79830982974):3.078409478,77:7.876719307):5.4449206205100005):2.747315661,((72:11.743839068,71:11.743839068):1.13825828,73:12.882097347999999):3.18685824):14.297696260999999):5.768449597,((((((((19:5.244034804,20:5.2440348032):8.612698433,(24:8.2789891782,23:8.278989179):5.577744057):0.06171901625,(21:5.381896942,22:5.3818969415):8.53655531):1.519342352,(26:10.7400654988,25:10.7400654995):4.6977291054000005):6.179117161,27:21.6169117698):1.89231097,28:23.509222734999998):4.8294205247,(((32:13.916053212,31:13.9160532121):2.4119359511000003,30:16.327989162):1.668832301,29:17.9968214633):10.341821797000001):2.841393605,(((64:10.04886108,65:10.0488610833):3.798336873,((53:9.377081736,(((60:5.403387879,(61:2.511890687,62:2.5118906868):2.891497193):0.2330158296,63:5.636403709):1.368854686,(59:6.697694,(57:3.413166595,58:3.4131665939999998):3.2845274062):0.3075643944):2.371823341):2.122790592,(54:10.57404159,(55:6.690405472,56:6.6904054719):3.883636122):0.9258307345):2.347325629):10.348274584999999,((((33:2.97833163,34:2.97833163):3.360465473,35:6.338797102799999):4.820274941,((43:7.880241394,(44:6.315324199,45:6.3153241986):1.564917195):2.293249122,((((40:4.777150213,39:4.777150213):1.711589355,41:6.488739568000001):2.092089827,((37:5.110832574,36:5.1108325741):0.8491724912,38:5.960005065000001):2.62082433):0.4032162626,42:8.984045658):1.1894448579999999):0.9855815281):11.206583859800002,(52:15.02173189362,((47:8.575006134,46:8.5750061347):3.203905787,((48:5.680187603,49:5.680187603):3.074666569,(51:8.626299691,50:8.62629969):0.1285544818):3.0240577495000003):3.2428199732):7.343924009):1.8298166359999999):6.984564324):4.9550645840000005):5.188063455,(78:34.31569822575,((79:2.764419695,80:2.764419695):16.31887811,((((83:13.23954836,(82:9.5566374379,81:9.556637437):3.682910923):0.8818905762,84:14.121438936):0.6425662729,85:14.76400520915):2.117573677,(86:8.506015783,87:8.506015783):8.375563103):2.201718922):15.2324004139):7.0074666787):4.650707305,((((((((((((93:12.478639414000002,((97:6.618964757,(95:3.260059446,96:3.260059446):3.3589053113):1.061747116,98:7.6807118725):4.7979275402):1.105163039,94:13.583802454699999):1.991174163,(100:11.74314581,99:11.74314581):3.831830806):6.3043421528,101:21.879318763):1.390945509,102:23.2702642758):5.710126544,((((((((((106:10.51455775,107:10.514557745200001):3.851020155,(((108:7.035449658,(109:0.3506955879,110:0.3506955879):6.6847540699):1.305963266,(111:5.167318105,112:5.167318105):3.17409482):2.964097291,113:11.305510215200002):3.0600676851000004):1.987896842,(115:3.126562673,114:3.126562673):13.226912068499999):0.3957928644,(117:7.653492343,116:7.6534923432):9.095775264):1.195751275,(121:9.0110852459,(120:2.008395724,119:2.0083957234):7.002689522599999):8.933933635599999):0.114043963,118:18.059062845):0.6701195503,((105:3.922355972,104:3.922355972):10.50453204,103:14.4268880196):4.302294379):0.6175539725,122:19.34673636664):0.95345241,((125:9.4460631713,124:9.446063171199999):6.334621773249999,123:15.780684944299997):4.5195038331):0.419764548,126:20.7199533255):8.2604374943):3.152962644,127:32.133353471999996):0.1486747129,(128:8.986863827,129:8.9868638273):23.29516435247):7.206775535,(((89:30.704529281,88:30.7045292781):2.58845158,(90:7.957426083,91:7.9574260830000005):25.3355547783):5.217206249,92:38.510187110000004):0.9786166111):3.496423354,130:42.985227068360004):2.041344693,(131:28.311624305,132:28.31162431):16.714947455):0.1339154335,(((((((141:10.64586167,(140:5.631909408,139:5.631909408):5.0139522578):1.212520526,142:11.858382192):6.633910063,(137:6.356278961,138:6.356278961199999):12.136013293393999):2.3452361313,((135:2.154692901,(134:1.654208934,133:1.654208934):0.5004839668):1.723264799,136:3.8779577005):16.959570685):9.346782554,(((143:7.246965651,144:7.246965651):18.59736156,(((((147:14.378955560600001,148:14.378955561):1.7302700351,160:16.109225597):2.152132863,(((((156:4.1021979624,155:4.1021979623):3.453038587,157:7.555236549):1.185413648,158:8.7406501963):0.1091577276,((150:6.578050307,149:6.578050307):2.1177600385,((152:2.3332656531000002,151:2.3332656534):2.84393794,(154:1.245955279,153:1.245955279):3.931248314):3.518606752):0.1539975792):3.076039854,159:11.9258477783):6.33551068):0.5228569926,(145:14.597602528,146:14.5976025237):4.186612927):1.936618833,161:20.720834285000002):5.123492927):2.695452378,((162:15.697128461450001,163:15.697128469099999):7.338086074,((((((164:4.309413714,(166:0.1559562859,165:0.1559562859):4.1534574279):1.334523237,(167:1.4696213,168:1.4696213):4.174315651000001):1.008638308,(170:0.9168716985,169:0.9168716985):5.735703561399999):1.093739809,171:7.746315069):3.393996284,(172:3.042832244,173:3.042832244):8.097479109):0.7852707242,((179:6.784791685,((((175:2.495959288,174:2.495959288):0.7400687194,176:3.236028007):0.9057452795,177:4.1417732870999995):0.7981928703,178:4.939966156900001):1.8448255269):1.413161484,(181:5.276341658,180:5.276341659):2.921611511):3.727628908):11.1096324616068):5.504565047):1.644531356):0.5685605443,((183:17.309230076,182:17.30923008046):7.04529083,((184:3.3104381368999998,185:3.3104381368):3.259999595,186:6.5704377318):17.784083174299997):6.39835058):5.155958958,(187:0.3082923833,188:0.3082923833):35.600538061514904):9.251656751):0.8133850128):4.6060490528);

TREE tree_1858 = ((18:32.9219903006,((((1:21.47120112,2:21.471201115):0.9960301139,(3:19.498608027,((4:15.63087501,((5:2.803615165,6:2.8036151652):8.182091054,((8:5.980122553499999,7:5.980122552999999):1.630912971,9:7.6110355226):3.3746706939999997):4.645168791):2.668759305,10:18.29963432):1.1989737139):2.968623204):1.9834243752999998,(11:11.46971214,12:11.46971214):12.980943476):4.953897476,((((15:5.453588764,14:5.453588764):3.5736779629999997,13:9.027266728153):0.4878205062,16:9.515087234):1.246628627,17:10.7617158595):18.642837224799997):3.5174372157000002):26.946252886000003,((((((((((((37:7.302518003,36:7.3025180034):0.9707753454,38:8.273293349):3.904179952,((39:5.604846463,40:5.6048464633999995):4.363881378,41:9.968727841):2.20874546):1.441975871,42:13.619449172):0.8393042838,((44:10.687992612999999,45:10.687992612999999):1.85593331,43:12.54392592):1.9148275315999999):1.192288856,((34:4.108086448,33:4.108086448):4.305506514,35:8.413592962):7.237449349):17.4928601178,((((51:11.84994349,50:11.8499434947):0.3288423193,(49:6.671754259,48:6.671754258):5.507031555):3.809287078,(47:14.237538319,46:14.2375383147):1.750534578):6.192876198,52:22.18094908995):10.962953341):6.6348186083,(((((((21:6.711724137,22:6.711724137):12.620091367999999,(23:10.424927253,24:10.424927253):8.906888251):0.8898052882,(19:5.60867152,20:5.6086715205):14.61294927):1.791320028,(25:17.2713398697,26:17.2713398698):4.74160095):4.779487621,27:26.792428439):6.1418207738,28:32.934249215):3.412261965,(((53:11.11030081,(((57:4.804736797969,58:4.8047367981):3.013256708,59:7.8179935060000005):0.1888537722,(60:6.329340463,((61:3.990419439,62:3.990419439):1.328498691,63:5.318918131):1.010422332):1.677506815):3.103453529):1.741173231,(54:12.7279987,(55:7.833527716,56:7.833527715000001):4.894470984):0.123475338):5.789514077,(64:14.34500761,65:14.345007605):4.295980505):17.7055230647):3.432209855):0.2533086054,((30:20.316280149,(32:18.0275295553,31:18.027529556399998):2.2887505892599997):4.195640973,29:24.511921119):15.520108524):7.9594005789999995,(66:39.21352354,((((72:12.7340401553,71:12.734040154999999):1.145430865,((76:4.148853514,(74:1.024093231,75:1.024093231):3.124760283):7.833511534,77:11.982365048):1.897105971):0.01957472447,(((68:7.3008929357,67:7.3008929362999995):4.015628723,69:11.316521658):0.7799455338,70:12.09646719):1.802578552):0.7945973797,73:14.693643119):24.519880413000003):8.77790668):5.544519039,(78:49.166905831,((((((81:13.654875127,82:13.6548751255):0.6721970342,83:14.327072159):1.215449727,84:15.542521889):1.61601783,85:17.158539716):2.379314752,(87:10.11143331,86:10.11143331):9.426421156999998):7.639103507,(79:2.879717413,80:2.879717413):24.297240563000003):21.989947854):4.3690434316):1.013263692,(((((((((101:26.5999527429,((99:20.78353738,100:20.783537387000003):0.8679105845,((((97:9.000549059,(95:6.644761129,96:6.644761129):2.35578793):2.116455927,98:11.117004984900001):3.7803291615,94:14.897334143):0.1682309186,93:15.0655650639):6.585882903):4.948504775):0.7520427036,102:27.351995446990003):13.257536653,((((((((((106:13.60185136,107:13.6018513647):5.971471978,(((111:5.30159408,112:5.3015940801):7.497333678,(108:10.91770789,(109:0.3697550078,110:0.3697550078):10.547952885299999):1.881219864):0.4480389975,113:13.2469667548):6.326356588):1.116821552,(114:3.515358005,115:3.515358005):17.174786889099998):1.490368391,(117:8.7198271015624,116:8.719827102):13.4606861843):0.8273402524,118:23.00785353923):0.5804603416,(121:12.5728261671,(120:2.400520839,119:2.400520839):10.1723053277):11.01548771334):0.9443332283,((104:3.291612939,105:3.291612939):13.58948994,103:16.881102873):7.651544232000001):0.7770077061,122:25.3096548171):1.948337824,((125:13.412399817699999,124:13.412399816499999):6.539677694650001,123:19.952077511200002):7.3059151273000005):0.02697108796,126:27.284963728):13.324568373800002):2.908678211,127:43.518210311):0.5152794237,(128:11.5136375873,129:11.5136375876):32.5198521456):5.334561374,(((89:39.153152652,(90:12.561021660999998,91:12.56102166):26.592130988999997):6.341642302,88:45.494794946599995):3.607344873,92:49.102139824661):0.2659112862):1.23390145,130:50.6019525576):0.8593825656,(131:35.76600298,132:35.766002973):15.695332145):0.6893149824,((((((143:6.944778576,144:6.944778575):23.39225127,((((((((156:4.9624634885,155:4.962463488):3.591461268,157:8.553924755):1.294097523,158:9.848022279299999):0.4914770997,((151:2.6058371251,152:2.6058371258):5.183466493,(154:1.312776605,153:1.3127766048999998):6.476527013999999):2.55019576):0.2326599625,(150:8.026332423,149:8.026332423):2.5458269187):2.079221383,159:12.651380725):7.973260926,(((148:16.826460320099997,147:16.826460319):2.3498072946999997,160:19.176267614):0.8988577958,(146:14.759977453000001,145:14.759977451000001):5.315147956):0.5495162402):2.029438041,161:22.654079691):7.682950157):1.576253092,((162:26.9989072617,163:26.998907262):0.631659241,((((((164:7.164593093,(165:1.091707231,166:1.091707231):6.072885862):2.070608734,(167:1.684981593,168:1.684981593):7.550220234):2.663944976,(169:2.839331844,170:2.83933184429):9.05981496):1.221901,171:13.1210478):2.77557231,(173:4.374242322,172:4.374242322):11.522377792):0.3194843209,((179:10.42371792,((177:7.791017672,((175:5.261777732,176:5.261777732):1.410948898,174:6.67272663):1.1182910426000001):0.6082648616,178:8.399282534000001):2.024435386):0.391162741,(180:6.748853548,181:6.748853548):4.066027113):5.401223773):11.4144620651743):4.282716448):2.283608684,((((133:0.7909314783,134:0.7909314783):0.8966514519,135:1.68758293):1.298131698,136:2.9857146284000002):19.757282875,((138:5.038976972,137:5.03897697277):14.859261979299998,((141:5.263908316,(140:4.594098069,139:4.594098068):0.6698102468):4.78523013,142:10.0491384449):9.849100508):2.8447585502):11.45389413):2.002923552,((183:17.223364612,182:17.223364613):6.717257156,((185:2.431093273,184:2.4310932734):6.3621155713,186:8.7932088435):15.1474129238):12.259193412):4.448659241,(187:0.01069644312,188:0.01069644312):40.637777970757995):11.502175683699999):2.398562847):5.319030232999999);

TREE tree_7595 = ((18:23.749404940999998,(((11:9.279623311,12:9.279623311):9.103965916,((1:14.56183094,2:14.561830937):0.9403538102,(3:13.083792833,((((5:1.6619063,6:1.6619062999):6.856316537,((7:4.4837953965,8:4.483795396064):0.9562888220000001,9:5.44008421853):3.0781386193999998):2.23371369,4:10.751936528):1.849322998,10:12.601259525):0.48253330818):2.4183919126999998):2.8814044805999997):2.443994001,((16:7.234318602,((14:4.1806134144,15:4.1806134143):2.241807099,13:6.4224205131000005):0.8118980893):0.5563454568999999,17:7.790664058500001):13.0369191702):2.9218217116):27.329624561,((((66:24.61356978,((((((76:4.335734197,75:4.335734197):0.8720038352,74:5.207738033):2.365074197,77:7.572812229):3.543498073,(70:9.435664921,((68:4.478819236,67:4.4788192358800005):3.879132813,69:8.357952049000001):1.077712872):1.6806453819):0.1419149401,(71:10.423274292,72:10.4232742921):0.8349509505):0.9155437863,73:12.173769024):12.4398007516):14.799276924,(((((((((21:5.038513499,22:5.0385134990000005):6.8560666463999995,(23:6.702196001,24:6.702195999800001):5.192384146):0.536886048,(20:3.7744334387,19:3.7744334386):8.657032754):1.890078406,(25:9.106141049,26:9.106141049):5.2154035504000005):4.345420595,27:18.666965194899998):0.9722860133,28:19.639251208):6.9938092115999995,(((32:12.594944027,31:12.5949440266):1.3952861783400001,30:13.990230206):3.184296232,29:17.174526438):9.4585339822):6.178560895,((52:20.433283919049998,((46:13.428750578999999,47:13.428750578999999):2.360688424,(((49:7.412462999000001,48:7.412462999000001):3.253298495,51:10.665761494100002):0.3412176856,50:11.0069791794):4.782459823):4.643844919):11.239691126,(((33:4.944651962,34:4.944651962):4.482981173,35:9.427633134):5.676472383,(((45:9.105167523,44:9.105167521999999):4.322885403,43:13.428052925):0.4999525593,((((37:7.3997207785,36:7.3997207786):1.519162453,38:8.918883232):2.7485871,(41:9.098455295,(40:6.054796714,39:6.054796715):3.043658581):2.569015037):0.840628223,42:12.508098554):1.4199069298):1.176100033):16.5688695271):1.1386462612130999):1.342297626,((64:13.40530446,65:13.405304464):2.812362266,((54:11.64670603,(55:6.92573043,56:6.9257304309):4.7209756039999995):2.0880145,(53:10.26755219,(((61:3.017218524,62:3.0172185249):1.474672747,(63:4.222182613,60:4.222182613):0.2697086586):4.732659445,((57:6.275395517,58:6.275395517):2.14143295,59:8.416828468):0.8077222492):1.043001469):3.467168348):2.482946197):17.9362522124):5.258927766999999):5.524161165,(78:38.997946997,((((85:15.3190924099,84:15.319092413999998):0.3334220434,((82:10.648970427,81:10.648970428):0.8140295724,83:11.4630000002):4.189514453):0.600590159,(86:7.808176152,87:7.808176152):8.4449284586):2.846714738,(80:3.038047081,79:3.038047081):16.061772269000002):19.8981276456):5.939060873000001):1.694379077,(((((((89:30.86974744,(91:7.098305603,90:7.098305603):23.771441832):6.071353114,88:36.941100549299996):3.647703636,92:40.588804185):1.109758365,(((((101:16.8445994058,(((93:9.469271019099999,(((95:3.410364474,96:3.410364474):1.61103332,97:5.0213977945799995):0.308806808,98:5.3302046031):4.1390664159899995):0.1849900762,94:9.654261095599999):3.521999733,(100:11.98804838,99:11.98804838):1.188212445):3.6683385787000002):0.2045335114,102:17.04913291867):5.8289316509999995,(126:15.182764383999999,(((((105:2.893963159,104:2.893963159):8.936671686,103:11.8306348459):2.192489217,((((((106:7.879174218999999,107:7.8791742182):3.279011597,(((108:6.0458827142,(109:0.06628346937,110:0.06628346937):5.9795992447):1.48837057,(111:3.6602402973,112:3.6602402976):3.874012987):1.352734412,113:8.8869876962):2.2711981186):0.7644154121,(115:2.506270937,114:2.506270937):9.416330291600001):0.6661751348,(117:5.900517972,116:5.9005179723):6.6882583904):0.8604416832,((120:1.703560514,119:1.7035605137):6.4346328949,121:8.138193409300001):5.3110246372):0.1226317628,118:13.571849808):0.4512742544):0.4743459632,122:14.497470025970001):0.6357445384,((124:6.985564536799999,125:6.9855645368):5.2706815991,123:12.25624613578):2.8769684295999998):0.049549819352):7.6953001856):1.438055092,127:24.316119658799998):1.30055384,(128:7.1863422175,129:7.1863422169):18.4303312833):16.08188905):2.557919821,130:44.256482367000004):1.74431748,(131:17.8916939436,132:17.891693945):28.109105901999996):0.3713483214,((((((143:7.334098372,144:7.334098372000001):21.02538788,(((((148:14.6537321695,147:14.653732168500001):0.6850549081999999,160:15.338787077):0.3671110642,(((150:7.088154661,149:7.0881546615):1.922507608,((158:8.41301296548,((155:4.172192069,156:4.172192069):2.336735266,157:6.5089273352):1.9040856303):0.5474437857,((153:0.7617661043,154:0.76176610427):5.301285052,(152:2.3682861797,151:2.3682861791):3.694764977):2.897405596):0.05020551717):1.892824186,159:10.903486454):4.802411687):1.757861174,(146:13.7266197163,145:13.726619714):3.737139599):2.086353337,161:19.5501126542):8.8093736):0.4730246469,((162:13.260202402,163:13.260202397):11.49631549,((((((164:5.25309002,(166:0.2137550061,165:0.2137550061):5.039335015):2.737960227,(167:2.03544444,168:2.0354444398):5.955605808):1.355971218,(170:1.975388856,169:1.9753888556):7.371632611):0.582551668,171:9.929573134):2.043395352,(173:5.446439491,172:5.4464394912000005):6.5265289954000005):0.6537539512,((((177:5.572283848,((175:3.278293313,174:3.278293313):0.7445595978,176:4.02285291):1.5494309374):0.6286912838,178:6.200975132):1.080830112,179:7.281805244):1.094688351,(180:6.373420472,181:6.373420472):2.0030731226):4.250228843):12.1297954521048):4.075993015):3.943152821,((183:15.67774801,182:15.677748008):7.953938774,((184:2.846456267,185:2.846456267):5.505416705,186:8.351872972):15.2798138097):9.1439769379):0.7494835942,((((134:2.516156646,133:2.516156646):2.374341995,135:4.8904986414):1.667436296,136:6.557934937):17.638508072,(((141:6.176977,(140:2.323079013,139:2.3230790136):3.853897987):6.066429018899999,142:12.243406018970001):6.4405233655,(137:4.115788295,138:4.115788296):14.5681410882):5.5125136251):9.32870431):3.934310633,(187:1.688840561,188:1.688840561):35.770617385):8.91269022):0.2592387772):4.4476425524000005);

TREE tree_4171 = ((18:28.2495425811,(((11:9.973754177,12:9.973754177):8.695545438,((1:17.06977127,2:17.069771268):0.5790068797,(((((5:3.831766824,6:3.83176682408):5.472007682,(9:6.299239947,(8:5.6289261102,7:5.62892611086):0.6703138356):3.0045345599999997):1.773027139,4:11.076801645):2.702001103,10:13.7788027488):0.4320618929,3:14.210864642):3.437913507):1.0205214681):4.626853871,((((14:4.344563847,15:4.344563848):2.495750085,16:6.840313932):1.3960281675,13:8.23634209968):1.395037606,17:9.631379706499999):13.664773781800001):4.953389093399999):21.367228118200003,((((66:34.10418513,((((74:1.091888156,75:1.091888156):11.84270947,77:12.934597624):1.314803855,76:14.249401479):3.746684798,(((71:12.375852693999999,72:12.375852692399999):3.958766618,(70:15.36193557,(69:14.325384324,(68:7.669443007,67:7.669443007):6.655941317):1.03655125):0.9726837367000001):0.9783325161,73:17.312951827):0.6831344497):16.108098852399998):6.1087325261,((((((((24:9.0737931655,23:9.073793166):5.568009701,(19:5.797364961,20:5.7973649603999995):8.844437906):0.08192130371,(21:5.465147394,22:5.465147394000001):9.2585767765):2.261762479,(26:11.4135045988,25:11.413504599000001):5.57198205):5.775530627,27:22.761017277):3.1444469413,28:25.905464218000002):7.439615302,(((31:17.211177331600002,32:17.211177326999998):0.4646635181,30:17.67584085):2.674545307,29:20.350386158):12.994693364):2.982909795,(((52:17.9011208498,((51:10.16974965,((49:6.340583898,48:6.340583898):3.753131431,50:10.093715329):0.0760343229):4.196088912,(46:11.4003155769,47:11.400315577):2.965522987):3.5352822886):10.02046412,(((43:11.99456146,(44:8.614876109,45:8.6148761085):3.379685355):1.955867445,((((36:7.311127953,37:7.311127953):1.181348322,38:8.492476275000001):3.186176535,(41:8.4912610845,(40:5.399214392,39:5.399214392):3.0920466924):3.187391726):0.3370665645,42:12.0157193748):1.9347095336):1.065685499,((34:6.049252872,33:6.049252872):2.767871414,35:8.8171242858):6.198990122):12.905470562):4.5661828776,(((54:16.9838832,(55:9.1454161196,56:9.14541612):7.838467076000001):1.631006088,(((((61:5.068347535,62:5.0683475342):3.581760719,(63:8.444468532,60:8.444468532):0.2056397218):3.021309711,(57:5.886650769,58:5.886650769):5.784767196):1.349568314,59:13.0209862789):2.45255435,53:15.47354063):3.141348654):6.128322633,(64:17.83563358,65:17.835633584):6.907578335):7.7445559323):3.8402214658):3.884928346):3.739681945,(78:37.170206317,((79:3.267852881,80:3.26785288074):14.26335303,((((83:12.05094341,(81:9.4670684544,82:9.467068453):2.5838749545):2.104167594,85:14.155111003):0.219780627,84:14.374891629):1.195872957,(87:7.017408868,86:7.017408868):8.5533557197):1.9604413250000001):19.639000409999998):6.782393282):0.9869931851,(((((((89:26.193074419,(90:7.008803017,91:7.00880301697):19.18427139912):4.437703788,88:30.630778199000005):4.70102469,92:35.331802889):6.002312498,(((((101:16.0596936507,((((98:8.1571868539,((95:3.265545081,96:3.265545081):2.276098719,97:5.5416438):2.6155430529):2.167757811,94:10.324944663100002):0.1186628048,93:10.4436074685):2.848727328,(100:11.47599001,99:11.47599001):1.816344787):2.7673588531):0.33235165,102:16.39204530044):5.0656238416,((((((105:3.171736607,104:3.171736607):7.394964191,103:10.566700799):2.4105281610000002,((((((106:7.758858415,107:7.75885841617):2.916151279,(((108:4.6261645498,(109:0.1727970966,110:0.1727970966):4.4533674526):0.9930369314,(111:3.677502978,112:3.6775029772):1.941698503):1.913536684,113:7.5327381652000005):3.1422715295):0.264730633,(115:1.846735004,114:1.846735004):9.0930053239):0.6082675426,(117:5.1398951001,116:5.1398950996999995):6.408112770300001):0.8830053122,118:12.431013182000001):0.01345599798,(121:6.6680799467,(120:1.444119353,119:1.444119353):5.2239605924):5.776389233899999):0.5327597794):0.4961287712,122:13.4733577264):1.655516995,(123:11.793690247499999,(124:6.56368719335,125:6.5636871935):5.23000305349):3.3351844781000004):0.3381925819,126:15.4670673075):5.990601832099999):1.133895211,127:22.59156435):0.5554234295,(128:6.173188640199999,129:6.1731886404):16.97379913923):18.18712761):2.26325239,130:43.597367785):0.5900248124,(132:35.403488507,131:35.40348851):8.783904076):0.5056525151,((((((143:8.207753751,144:8.20775375):16.49962568,(((146:12.3396136225,145:12.339613622):5.136329751,(((148:14.782964193,147:14.782964192):1.6384824033999998,160:16.421446596):0.417335916,((((154:0.7827631028,153:0.7827631028):4.408566748,(152:1.4689095142,151:1.4689095140000001):3.722420337):3.882363155,((((155:4.898946834,156:4.898946834):2.3659950441,157:7.2649418777):0.8044289896,158:8.069370866722):0.510032807,(149:3.71121575,150:3.71121575):4.868187924):0.4942893322):1.79987953,159:10.873572536000001):5.965209977):0.6371608613):2.1755041,161:19.651447474):5.055931962):0.9044913344,((162:9.317113025000001,163:9.317113025000001):14.58035665,(((171:9.635586129,(((164:5.724607416,(165:0.6299924814,166:0.6299924814):5.094614935):2.5930894931,(167:2.95458277,168:2.95458277):5.3631141396):0.6872399143,(169:2.826564651,170:2.8265646503):6.178372173):0.6306493059):1.922074397,(172:4.515939308,173:4.515939308):7.041721217999999):2.330189787,((181:7.272935339,180:7.272935339):3.636031999,((((177:5.239449078,175:5.239449078300001):0.6069912547,(174:5.563763102,176:5.563763102):0.282677231):1.175295133,178:7.021735466):2.196615885,179:9.218351352):1.690615986):2.9788829758000004):10.0096193629):1.714401093):1.7882216,(((137:4.12890717136,138:4.128907171):9.736274805999999,((141:5.369009102,(140:3.240213027,139:3.2402130270000002):2.1287960754):5.0015684226,142:10.370577525000002):3.4946044515):1.21822652086,(((134:1.478332282,133:1.478332282):1.510784859,135:2.989117141):0.3704387087,136:3.3595558493):11.723852649):12.31668387):1.853863939,((183:15.596557605,182:15.596557599999999):2.991962983,(186:5.16084222,(184:2.461159258,185:2.461159258):2.6996829625):13.4276783626):10.665435727):8.983251786,(187:0.1838357279,188:0.1838357279):38.053372371702594):6.455837013):0.246547678):4.6771779121999995);

TREE tree_7322 = (((((11:8.808537444,12:8.808537444):17.405937059,((1:21.76756782,2:21.767567826):0.5514566559,(3:19.185253354,((4:16.437750931,(((7:6.8504313759999995,8:6.8504313762999995):1.332219794,9:8.1826511703):5.009060609,(6:4.249852943,5:4.2498529424):8.9418588371):3.2460391512499998):1.821918922,10:18.259669857000002):0.9255835017):3.133771123):3.895450027):4.515222655,((((15:5.982843424,14:5.982843424):4.67856446,13:10.661407883999999):0.5284777599,16:11.18988564):0.8346829576,17:12.0245686015):18.705128558760002):1.7936697314,18:32.5233668890252):15.448607637,((((66:32.10150855,(((72:11.106248412,71:11.1062484117):0.7583144936,((74:9.412099223,(75:2.057604858,(77:1.295063444,76:1.295063444):0.7625414133799999):7.354494365500001):1.599550614,(70:10.31084394,((68:6.135798421,67:6.1357984211000005):2.4139573585000003,69:8.549755780000002):1.761088156):0.7008059012):0.8529130685):1.155963005,73:13.020525905):19.080982640099997):4.205328477,((((((47:13.56273778,46:13.56273778436):2.777588891,((50:11.2841302,51:11.284130199):0.5039471938,(48:7.178989375,49:7.178989375):4.609088018):4.552249282):5.358011172,52:21.6983378519):9.921489486,(((33:4.022660614,34:4.022660614):4.527889979,35:8.550550594):8.312093986,(((((37:6.6247350350000005,36:6.6247350357):1.510184147,38:8.134919183000001):3.875397369,((40:6.219605527,39:6.219605527):1.274759519,41:7.4943650464):4.515951507):1.000307581,42:13.010624133099999):2.108712523,((44:9.546786819,45:9.546786818200001):3.557020584,43:13.103807402):2.0155292551):1.743307923):14.7571827568):1.829667447,((29:17.1656243891,((31:11.93297723528,32:11.9329772362):2.35649822425,30:14.289475459):2.8761489293):10.421199381,(((((19:5.809252752,20:5.8092527518):7.33098493,((23:6.6045793669999995,24:6.604579367):5.90053855284,(21:4.956983851,22:4.9569838504):7.5481340679999995):0.6351197628):4.157746669,(26:9.8960715164,25:9.896071516):7.4019128335):4.845596748,27:22.1435811021):1.136372967,28:23.2799540643):4.3068697051):5.86267101):0.6362345646,((64:13.6501861,65:13.650186095):2.656090692,((54:11.2648269135,(55:7.421043899,56:7.421043899200001):3.843783017):1.085855086,(53:11.1568455,(((57:4.381274009,58:4.3812740094):3.497615636,59:7.878889644):0.7786455904,(60:7.711830705,((61:5.588997892,62:5.588997892):1.648176145,63:7.237174037):0.4746566685):0.9457045295):2.49931027):1.193836497):3.955594788):17.7794525531):2.2211076839999997):4.703050648,(78:36.55014954799999,((((((82:10.49123591831,81:10.491235918000001):1.251246865,83:11.742482782):2.591138253,84:14.333621034700002):0.7569310074,85:15.0905520429):0.2617887802,(86:8.834305192499999,87:8.834305193):6.518035630900001):1.63111939,(80:3.539424844,79:3.539424844):13.44403537):19.566689332):4.4597381236):2.30119868,(((((((89:29.723307137,(91:9.886684003,90:9.886684003):19.836623139):0.8342382713,88:30.55754541037):3.618610034,92:34.176155441400006):5.208195106,(((((101:15.931037183599999,((100:10.90824968,99:10.90824968):1.988085266,((((97:5.801225575,(95:2.544310582,96:2.5443105819999996):3.2569149925):0.7330012918,98:6.53422686702):2.3849151761200003,93:8.919142043):0.2122714491,94:9.1314134916):3.764921451):3.0347022370000003):0.3419702861,102:16.273007467299998):8.081441293,((((((((((106:8.627956006,107:8.627956006299998):4.360520118,(((111:4.637364704,112:4.6373647048999995):3.775165698,(108:6.2571526512,(109:0.1076882513,110:0.1076882513):6.1494644007):2.15537775):2.08333988,113:10.495870282999999):2.4926058415999996):0.3471822838,(114:2.791360133,115:2.791360133):10.544298274):0.5930739997,(116:5.95869903931,117:5.95869903877):7.970033368):0.5966151343,118:14.525347542):0.299999898,((120:1.584684099,119:1.584684099):6.1596254151,121:7.7443095133):7.081037926):0.7085021434,(103:10.58354862,(104:2.671965099,105:2.671965099):7.911583525999999):4.950300958):0.09912402297,122:15.632973605):2.612539716,(123:14.50134789187,(125:8.222293303999999,124:8.222293304):6.2790545889):3.7441654305800003):0.289896033708,126:18.535409356):5.819039404):3.564608293,127:27.919057048):0.9857419015,(128:7.8104500518,129:7.810450052):21.094348899199996):10.47955159):0.5814459987,130:39.9657965477):1.897283887,(131:34.57118922,132:34.571189223000005):7.291891209000001):0.7235006864,((((((143:7.048960096,144:7.048960096):17.84022605,(((((147:14.48782402551,148:14.487824026):1.7712729681000001,160:16.259096994):0.3839172029,(145:11.904650177,146:11.904650172340002):4.738364025):0.7174234781,(((((155:4.82383513,156:4.82383513):2.953493733,157:7.777328862999999):1.330258852,158:9.107587714600001):0.01092482942,(((154:0.6719919394,153:0.6719919394):4.905840179,(152:1.4908680356,151:1.4908680356000001):4.0869640827):2.852581966,(149:5.357925254,150:5.357925254):3.072488831):0.6880984590399999):1.458312101,159:10.576824645999999):6.78361303):2.86200539,161:20.222443064):4.666743085):1.20438691,((162:19.71869575681,163:19.7186957557):2.335875681,((((((164:5.452698198,(166:0.3167896404,165:0.3167896404):5.1359085574000005):1.425616955,(167:3.254721153,168:3.254721152):3.6235939999999998):0.468697735,(170:1.597508103,169:1.5975081034):5.749504784):1.285268806,171:8.632281694):1.581285958,(172:4.4015011144,173:4.4015011137):5.8120665371):0.08080537569,((((((174:2.959456278,176:2.959456278):0.5348965077,175:3.4943527857000003):0.2425686796,177:3.7369214654):0.1433817811,178:3.880303247):1.375023615,179:5.255326861):1.178079263,(180:3.852062131,181:3.852062131):2.5813439935):3.8609669030000004):11.7601984077181):4.03900162):3.861512109,(((142:11.577152943,(141:5.912554326,(140:4.716100863,139:4.716100863):1.1964534636000002):5.664598616999999):8.068139191,(137:4.939777672100001,138:4.939777673):14.7055144608):3.87810286,((135:4.30460898,(134:2.100718238,133:2.100718238):2.203890742):0.4961846216,136:4.8007936010000005):18.722601395999998):6.431690176):1.435196689,((182:18.103706120299996,183:18.103706120000002):3.157213563,(186:6.204716873,(184:2.92114307,185:2.9211430694000002):3.2835738031):15.056202811):10.129362167):2.787804734,(188:1.458172118,187:1.4581721176):32.7199144782459):8.408494531):0.7245052327):4.6608881748);

TREE tree_4581 = ((18:32.4780950467209,(((11:12.7259511,12:12.7259511):11.650392672999999,((1:18.80758893,2:18.807588927):1.748002159,(((((5:3.6874482427,6:3.687448243):9.126407861,((7:6.90147169595,8:6.901471696330001):0.7118013249,9:7.6132730212999995):5.200583083):2.530399831,4:15.344255936):1.608883683,10:16.953139622):0.4948074165,3:17.4479470339):3.1076440520000004):3.820752688):3.371164428,((13:9.308784564,(16:8.504443968,(14:5.946596075,15:5.9465960742999995):2.557847893):0.80434059658):1.330028103,17:10.638812667400002):17.108695538):4.7305868465):20.493142544999998,(((((((((((21:7.547732827,22:7.547732827):8.868662110599999,(23:9.028527665999999,24:9.0285276653):7.387867271999999):0.9190712271,(19:5.160575236,20:5.1605752365):12.174890929):1.891592953,(25:11.857161665,26:11.8571616657):7.36989745295):4.990765388,27:24.217824505):1.223149044,28:25.440973551):6.586951688999999,((30:20.43573636,(32:17.701060281,31:17.70106027891):2.7346760861):2.8016480869000002,29:23.2373844491):8.790540789000001):2.643189553,((((((45:6.4323766556,44:6.4323766550000006):1.373047538,43:7.8054241928):0.9020420643,((((37:3.9737360167999998,36:3.9737360167999998):0.8010058134,38:4.7747418303):1.958746039,((40:3.908548513,39:3.908548512):1.819603118,41:5.7281516308):1.005336238):0.9121860902,42:7.64567396):1.0617922975):0.5189744251,((33:3.13824627,34:3.13824627):2.742171804,35:5.880418074):3.346022608):11.4328491263,(((47:8.1791134354,46:8.179113435):2.068440297,((50:7.191709426,51:7.191709425899999):0.270193204,(49:4.593818816,48:4.593818816):2.868083814):2.785651102):3.358449314,52:13.6060030461):7.053286762):1.3189323015999999,((53:9.903489264,((((57:4.591541328,58:4.5915413282):0.9455013979,59:5.5370427263):1.440025336,(((61:3.06485661,62:3.0648566099999996):2.088356456,60:5.153213066):1.506653765,63:6.659866831):0.3172012312):2.268993887,(54:7.573795001,(56:5.209380382,55:5.209380381200001):2.3644146193999998):1.672266949):0.6574273146):3.985236174,(64:9.777831478,65:9.777831478):4.110893961):8.0894966733):12.692892679):5.678843032,(66:32.04927435,((((72:12.886594689,71:12.886594691500001):0.8481781963,(((68:5.61363661,67:5.61363661):5.337081803299999,69:10.950718412699999):0.6576575526,70:11.60837597):2.1263969199):2.455751014,73:16.190523904):1.505733439,((76:4.076639009,74:4.076639009):2.852372136,(77:3.4285925327999998,75:3.4285925325):3.500418612):10.767246194):14.3530170115):8.300683471):3.960893564,(78:38.826249129,((80:1.338485489,79:1.338485489):18.08804513,((((82:9.928537961,81:9.928537961):3.3214938165000003,83:13.250031776):1.504676182,(85:14.612754471,84:14.612754470000002):0.1419534887):1.0820302285999999,(86:6.153076335,87:6.153076335):9.6836618515):3.589792429):19.3997185116):5.484602262999999):2.672874952,(((((((89:26.037447308,(90:7.664669945,91:7.664669945):18.372777367):4.110435971,88:30.1478832788):3.43577229,92:33.583655574):7.608757381,(((((101:19.7022889435,((99:14.87008381,100:14.87008381):1.047053972,((93:10.8422678911,((97:5.762319859,(95:4.753214757,96:4.753214757):1.0091051018800001):1.687678635,98:7.449998494000001):3.392269397):0.5653940489,94:11.407661939):4.509475843200001):3.7851511589999998):0.4183459476,102:20.120634889199998):8.1686950008,((((((105:3.477955568,104:3.477955568):10.21214787,103:13.690103431999999):4.500393539,(((((106:10.315389081229998,107:10.3153890796):4.088762038,(((108:8.221907940000001,(109:0.3585542237,110:0.3585542237):7.863353716400001):1.222124707,(111:5.819423522,112:5.819423521999999):3.624609124):1.505861282,113:10.94989393006):3.4542571888):0.4138692886,(115:2.954914216,114:2.954914216):11.863106189):0.4150797815,(117:7.1513537155,116:7.15135371529):8.081746471999999):1.98441113,(118:16.655427409,(121:8.3623755567,(120:1.452737684,119:1.452737684):6.909637871699999):8.2930518523):0.5620839081):0.9729856561):0.1798871896,122:18.370384163900003):0.97579094,(123:16.3349408164,(124:9.253622716,125:9.253622715999999):7.0813181003):3.011234287):0.1303771558,126:19.476552259199998):8.8127776313):2.306924509,127:30.5962544):0.2072005531,(128:8.553867113999999,129:8.553867113899999):22.2495878465):10.388958):3.78068359,130:44.9730965391):1.23615623,(131:20.915652144,132:20.915652144):25.29360063):0.3858783285,((((((135:4.280403456,(133:2.934696696,134:2.934696696):1.34570676):1.044287889,136:5.324691345):19.8832786309,((137:6.858973566,138:6.8589735656):16.4922791997,((141:6.840085568,(140:3.095219193,139:3.0952191931):3.744866375):6.195264061,142:13.035349629999999):10.315903130499999):1.8567172132):8.345051957,(((143:8.107841895,144:8.107841896):21.56575357,((((((((156:7.378694178,155:7.3786941778):1.13536302,157:8.514057198):1.733102131,(((152:2.515821222,151:2.5158212223):2.46985215,(154:0.3607661323,153:0.3607661323):4.62490724):3.891242338,(149:8.517526609,150:8.517526608):0.3593891024):1.370243619):0.1815022068,158:10.4286615353):2.366652131,159:12.795313666):8.021478061,((148:18.32673469,147:18.3267346905):1.7527183444,160:20.079453035):0.737338693):1.206550569,(146:15.17495570158,145:15.174955704):6.848386595):2.418122399,161:24.441464696):5.232130771):1.919542283,((162:13.1737512172,163:13.173751215000001):13.16160669,(((171:9.280834388,((((165:0.227222189,166:0.227222189):6.236643086,164:6.463865275):1.238906141,(167:2.297442458,168:2.297442458):5.405328959):0.5336802726,(169:0.9326141327,170:0.93261413275):7.303837557):1.044382699):1.610620306,(172:6.067647623,173:6.067647623):4.8238070708):0.4364939454,((179:6.62299618,((176:5.322688795,((174:5.023494817,177:5.0234948173000005):0.1381845851,175:5.1616794021):0.1610093931):0.2664420834,178:5.589130879):1.033865301):1.329678969,(181:3.272187883,180:3.272187883):4.680487265):3.375273491):15.007409263):5.257779854):1.959884178):2.85955214,((183:19.27008012,182:19.270080117200003):5.553391644,((185:2.9290292276,184:2.9290292276):5.580432308,186:8.5094615358):16.3140102251):11.58910231):2.46252523,(188:0.08183535538,187:0.08183535538):38.7932639395173):7.720031800799999):0.3885952369):5.9875112565999995);

TREE tree_1896 = (((((11:11.12420899,12:11.12420899):8.029321191,((1:16.42799262,2:16.427992625):1.087698193,((10:14.555718265,(4:12.215344471000002,((5:3.466116603,6:3.4661166029999997):6.162851915,((8:5.5715192254,7:5.57151922445):0.6587853713,9:6.230304597):3.3986639218):2.5863759533):2.3403737923):0.77466201629,3:15.330380281):2.1853105373):1.6378393659000001):4.029752291,((((14:4.164946302,15:4.1649463017):3.5468740579999998,13:7.711820359799999):0.4813556174,16:8.193175977):1.243417644,17:9.436593621689997):13.7466888518):5.2897839864,18:28.4730664566):19.967702859,((((66:31.84407314,(((71:12.369449502,72:12.369449502):1.450508986,((70:11.65837078,(69:10.1244185709,(68:5.865993629,67:5.865993629):4.2584249409999995):1.533952213):1.728853758,((75:5.656729487,74:5.656729487):1.080064999,(76:4.578532337,77:4.578532337):2.1582621492):6.6504300554):0.4327339474):1.496566596,73:15.316525084):16.5275480561):3.351130233,(((((53:7.976933113,((((57:3.775827694,58:3.7758276937999997):0.9335607869,59:4.7093884810999995):1.716488747,((63:4.670376792,60:4.670376792):0.872478878,(61:2.624249939,62:2.6242499394000003):2.918605731):0.8830215577):1.167083944,(54:6.788363443,(55:3.809759276,56:3.8097592762000003):2.978604166):0.8045977289):0.3839719418):3.926398957,(64:9.799832969,65:9.799832969999999):2.103499101):12.201245176,(((((19:4.213503153,20:4.2135031530500004):8.861782012,((23:7.926427214,24:7.9264272142):4.817510286,(21:5.152708119,22:5.152708119):7.5912293802):0.3313476653):2.594024771,(26:10.1029959724,25:10.1029959725):5.566313964):6.736200585,27:22.405510526):1.501581837,28:23.907092358999996):0.1974848916725):4.9215282078,((30:14.636340247,(32:11.476576079,31:11.476576079800001):3.1597641682):2.051561042,29:16.6879012891):12.338204171000001):0.1965016095,(((35:8.450913134,(33:6.216407855,34:6.2164078547):2.2345052784):6.614904172,(((((40:5.698890649,39:5.698890649):1.692804838,41:7.3916954877):3.674310079,((37:7.1219547190000005,36:7.1219547191):1.050812523,38:8.172767243):2.893238324):0.6880472424,42:11.754052810000001):2.233232782,((45:9.5103616693,44:9.510361669):1.882931997,43:11.393293666):2.593991924):1.078531715):11.549464665199999,(52:17.283483544,((47:10.72917763,46:10.72917763):2.862510371,((49:5.717038844,48:5.717038844469999):3.918348163,(51:9.3001807322,50:9.300180732):0.3352062747):3.956300995):3.691795542):9.331798426599999):2.6073250989999996):5.97259631):5.222417967,(78:33.698474786,((79:2.464392198,80:2.464392198):16.17495909,((((83:12.23884756,(82:9.6437565139,81:9.643756514):2.5950910475):1.566926981,85:13.805774542000002):0.2916053313,84:14.097379869300001):2.056492024,(86:7.555360589,87:7.555360589):8.598511307799999):2.485479395):15.0591234932):6.719146554):1.795552802,((((((((((((103:11.53131878,(105:2.06600636,104:2.06600636):9.465312421):1.5046983171,((((120:1.167028685,119:1.167028685):5.4149594219,121:6.5819881063):5.8169251606,118:12.398913267000001):0.04075799496,((((106:7.685753218,107:7.68575321832):3.153241872,((((109:0.1114160957,110:0.1114160957):4.994599162699999,108:5.106015258799999):1.058400795,(111:3.101106976,112:3.1011069757):3.063309077):1.519466356,113:7.683882409600001):3.1551126802000002):0.3830526588,(114:2.909513766,115:2.9095137665):8.312533982):0.1549581814,(117:4.4198874873000005,116:4.4198874876):6.9571184420000005):1.062665333):0.5963458358):0.2416932356,122:13.2777103333):1.060137516,(123:12.27787904134,(125:6.8320646174999995,124:6.8320646176699995):5.44581442436):2.0599688089):1.40480590544,126:15.742653756200001):4.689791775,(102:15.213277029599999,(101:14.808144777799999,(((93:8.353582894300002,(((95:3.005457743,96:3.005457744):2.500576319,97:5.5060340619999995):1.023808639,98:6.52984269982):1.8237401926999999):0.1363301621,94:8.489913056):2.773282714,(100:10.35048767,99:10.35048767):0.9127080947):3.5449490089999998):0.4051322518):5.2191685):0.639228811,127:21.07167434):0.6215899157,(129:6.4708693427999995,128:6.4708693422):15.22239491689):15.3053351,((((90:5.367321243,91:5.367321242399999):19.3498311838,89:24.71715243):3.987206163,88:28.7043585854):4.369556992,92:33.073915580299996):3.924683782):2.200307739,130:39.198907095):2.325821639,(132:29.762901944,131:29.762901945):11.76182679):0.2075789078,((((((143:6.964874736,144:6.964874736):18.2930681,(((((145:10.839094651,146:10.8390946503):4.238035323,160:15.077129974):0.6115640639,(148:13.261105909000001,147:13.261105909):2.4275881298):1.361406467,(159:12.199352892,(((150:6.970116676,149:6.970116676):1.342544043,((152:1.7518755744,151:1.7518755736):3.030249558,(153:0.3496104734,154:0.3496104734):4.432514659):3.530535586):0.9212150624,(((156:3.414748086,155:3.4147480867):4.088151819,157:7.502899905):0.9803299837,158:8.4832298888):0.7506458921):2.9654771098):4.850747614):2.165576173,161:19.215676682999998):6.042266156999999):0.3879519444,((162:15.291012632,163:15.291012631000001):6.792071209,(((172:3.700291286,173:3.700291286):7.9760431509,((((164:6.03067506,(165:0.2387656249,166:0.2387656249):5.791909436):2.227430104,(168:3.14124939,167:3.14124939):5.116855773999999):1.1555575617,(169:2.091419548,170:2.0914195482):7.322243178000001):0.9561092062,171:10.36977193):1.30656250438):0.9531302864,(((((176:4.956104008,(175:3.951697026,174:3.951697026):1.004406981):1.184275511,177:6.1403795183000005):0.6279615624,178:6.768341081):0.3630253898,179:7.131366471):2.110698496,(180:6.096802265,181:6.096802265):3.1452627019999997):3.3873997562):9.4536191196569):3.562810946):5.553120604,(((142:12.719330317,((140:3.519398662,139:3.5193986619999995):2.2276695217999998,141:5.747068183):6.972262133):5.124791334,(138:6.70907245099,137:6.709072451):11.135049198):4.5321796575,(((134:2.923142419,133:2.923142419):1.713464326,135:4.636606745):0.7097373897,136:5.346344134):17.029957174):8.822714082):0.5503032474,((183:16.1614375315,182:16.161437533):7.566734179,((184:2.169726165,185:2.169726165):5.741667324,186:7.911393489):15.816778222099998):8.021146918):2.300913651,(188:0.3659370019,187:0.3659370019):33.6842952859408):7.6820753580000005):0.4808665013):6.227595171);

TREE tree_4848 = ((18:36.3422779381155,((((1:18.9500913,2:18.950091304):3.408679161,((4:15.919920698,(((7:7.8072739061,8:7.807273906630002):1.288856775,9:9.0961306809):3.823833257,(6:3.164542432,5:3.1645424319999997):9.7554215052):2.999956755):2.335788873,(10:17.299070022,3:17.299070026):0.9566395441000001):4.1030608950000005):2.2383646352,(11:11.13275546,12:11.13275546):13.464379631):5.917852155,((((14:5.300557824,15:5.300557824599999):4.066721168,13:9.367278993):0.4065380093,16:9.773817001):0.825995189,17:10.5998121904):19.9151750613):5.827290678):15.7300252613,(((((((((34:5.269934695,33:5.269934695):2.988074622,35:8.2580093167):8.622335494,(((((40:6.906903015,39:6.906903014):2.488143567,41:9.395046581):3.248873383,((37:7.817280846300001,36:7.8172808461):0.7556708642,38:8.572951711):4.070968254):1.480120594,42:14.124040560000001):1.828608661,(43:14.18631719,(45:11.6369835478,44:11.636983548):2.549333646):1.766332026):0.9276955908000001):16.921630138,(((47:12.998273766,46:12.9982737656):2.994032632,(((49:6.330881111,48:6.33088111):5.321313801,51:11.65219491):0.1769712426,50:11.829166155):4.163140243):6.0301654521,52:22.0224718469):11.7795031):2.2553380805,(((32:15.9220915529,31:15.922091554800001):2.955652031,30:18.87774359):3.7544383297999997,29:22.632181915):13.425131114):0.5007115256,(((((((24:10.021519353299999,23:10.021519352999999):7.384316647,(21:7.146852983,22:7.1468529830000005):10.258983016):0.851054763,(19:6.234409491,20:6.2344094914):12.02248127):2.157819593,(26:12.4705438188,25:12.470543819):7.944166536500001):8.736837711,27:29.1515480665):2.082687423,28:31.234235488):4.263514511,((53:13.22182478,((54:12.03621246,(55:7.8660937032,56:7.8660937036359995):4.170118756):0.1178348986,(((57:4.036730388,58:4.0367303885):4.035553902,((61:5.490021516,62:5.4900215150000005):1.973126617,(60:7.310099055,63:7.310099055):0.1530490769):0.6091361579):1.409872859,59:9.482157148999999):2.67189021):1.067777425):3.589093842,(64:14.45168706,65:14.451687063):2.359231567):18.68683137146):1.060274559):2.5095246839999996,(66:32.33513431,(((71:14.187910938000002,72:14.187910937000002):1.776479968,(((76:5.01309601,77:5.013096011):3.94135544,(74:1.25819135,75:1.25819135):7.6962601):3.956205424,((69:9.736884506,(68:4.137608324,67:4.137608323799999):5.599276183):2.077031395,70:11.8139159):1.0967409727000001):3.0537340319):1.67393812,73:17.638329029):14.696805285):6.7324149283):5.327707072,(((((84:14.595324815,((82:11.55480371514,81:11.554803715):1.000760377,83:12.555564092000001):2.0397607222):0.8951542207,85:15.4904790355):2.681192007,(86:6.745086569,87:6.745086569):11.4265844736):1.876427563,(80:1.064856781,79:1.064856781):18.983241824):20.433137182000003,78:40.48123578304):3.9140205249999998):1.762162796,(((((((((((100:10.1176853,99:10.1176853):1.298146311,((94:7.60235224,93:7.60235224):1.056227842,((97:5.143779108,(95:3.630585981,96:3.6305859810000003):1.513193127):0.5595350004,98:5.703314108100001):2.9552659740900005):2.757251528):2.5190403599,101:13.934871970000001):1.1568965283,102:15.0917684973):9.108816902200001,((((((((((((108:6.3617517932,(109:0.1797340678,110:0.1797340678):6.1820177251):1.427533023,(111:3.720888603,112:3.7208886035999997):4.068396213):1.17718683,113:8.9664716446):2.9932060064,(106:7.549466408,107:7.54946640791):4.4102112434):0.4178198326,(115:2.256029803,114:2.2560298027):10.121467680999999):0.3861932642,(117:5.572928939,116:5.57292893861):7.1907618091000005):0.7452742749,118:13.508965023000002):0.4584491967,((120:1.221736035,119:1.221736035):4.5486825202,121:5.770418555050001):8.1969956648):0.7808228271,((105:2.261392915,104:2.261392915):9.984065998,103:12.2454589139):2.5027781338999997):0.5398887758,122:15.288125821600001):0.6791841211,((125:7.2323386126,124:7.2323386119999995):6.24631335441,123:13.4786519657):2.488657977):0.2823805252,126:16.249690468):7.950894931400001):6.15400927,127:30.354594674999998):0.3014223205,(128:9.280673406,129:9.280673406):21.375343585099998):12.61753217,(((89:27.797798057999998,(90:7.748534155,91:7.748534156):20.0492639078):4.903102713,88:32.7009007747):4.459662994,92:37.160563765999996):6.112985391):1.152586294,130:44.426135447):0.5008941972,(132:33.701986840000004,131:33.701986840000004):11.225042814):0.3453949404,((((((((139:3.764290043,140:3.764290043):1.441382572,141:5.2056726146):6.5771507317,142:11.782823347):6.394898532,(138:4.053216059,137:4.0532160583):14.1245058232):1.6914853415800002,((135:3.543882363,(134:1.790522085,133:1.790522085):1.753360278):0.1991499928,136:3.7430323563):16.12617486372):9.11525438,(((143:6.66896365,144:6.668963649):19.82957408,(((145:13.854061317000001,146:13.854061317100001):4.19086751,(((147:14.5445523677,148:14.544552367):2.1098817175,160:16.654434084000002):0.7523572917,((((150:7.01216502,149:7.012165021):0.4684908223,((151:1.5278127035,152:1.5278127036):3.758277419,(154:0.6079663737,153:0.6079663737):4.678123749):2.1945657199):0.8229478158,(((155:4.628060861,156:4.628060861):2.342646233,157:6.9707070943):0.8245036047,158:7.795210699409999):0.5083929592):2.897641357,159:11.201245015):6.205546361000001):0.6381374514):2.115918394,161:20.160847222):6.337690504):0.7040976137,((162:22.465143126,163:22.465143127):1.194024625,((((((164:6.625315951,(166:0.8582202957,165:0.8582202957):5.767095655):1.923566569,(168:2.363540097,167:2.363540097):6.185342422):1.022962941,(169:1.582876226,170:1.5828762252):7.988969235):1.003480314,171:10.575325774):2.64880072,(173:2.731938196,172:2.731938196):10.492188297999999):0.851343508,((181:6.077296406,180:6.077296406):3.133312378,(((176:5.638893227,((174:5.162613658,177:5.162613658300001):0.2813173957,175:5.443931054):0.1949621726):0.4526967193,178:6.091589946):1.23951816,179:7.331108106):1.879500678):4.8648612174):9.5836977437):3.543467588):1.78182626):1.605663048,((183:15.383561,182:15.3835610026):5.370183107,(186:6.9067375941,(184:3.4141954822000002,185:3.4141954820000002):3.49254211185):13.847006514):9.836380532):3.992866861,(187:1.273661726,188:1.2736617259999998):33.3093297756408):10.689433088):0.8849945183):5.914884084400001);

TREE tree_1879 = (((((11:13.75926126,12:13.75926126):11.52850822,((1:21.41788188,2:21.41788188):0.4802403713,(((4:17.168461049,((5:3.375906431,6:3.3759064309999998):9.716861193,((7:6.9176956999999994,8:6.917695699599999):2.3267517140000002,9:9.2444474138):3.84832021):4.0756934207999995):1.910170553,10:19.078631595999997):0.9691415288,3:20.047773125200003):1.8503491208999998):3.3896472299999996):5.263625088,((((14:4.961948361,15:4.96194836076):5.0094573553,16:9.9714057163):0.6102860438,13:10.581691761):1.567127782,17:12.148819542):18.402575021300002):7.5841977061,18:38.1355922709):13.6888206346,(((((((((((23:10.325124969,24:10.3251249679):4.5610801306,(21:6.955678368,22:6.955678368):7.930526732000001):1.932920495,(19:4.63785126,20:4.6378512602):12.18127433):2.0774886,(25:12.66044708,26:12.660447079999999):6.236167115900001):6.359989981,27:25.256604181):3.0936148011,28:28.350218977999997):6.938785672,(((64:11.71785586,65:11.717855856):4.539071516,((54:10.79537504,(55:7.09100937,56:7.0910093698):3.70436567):0.4250525354,(53:9.144872377,(((57:4.582041197,58:4.5820411975):2.269356093,59:6.8513972899999995):0.4598309392,(60:4.277267082,((61:2.605789664,62:2.6057896639):1.583908743,63:4.189698407):0.08756867467):3.033961148):1.833644147):2.075555198):5.036499796):9.0891844971,(((((48:5.515523415,49:5.515523415):3.203076208,(51:8.41155322,50:8.4115532203):0.3070464031):2.039791626,(46:8.7616570649,47:8.761657065):1.996734183):2.8124582845,52:13.57084953295):9.390038745,(((33:4.0449076,34:4.0449076):2.520643286,35:6.565550886):4.130880449,((43:8.849399232,(44:6.520560979,45:6.520560979):2.3288382526999998):0.7470145611,((((40:4.556785679,39:4.556785679):1.787324794,41:6.344110472700001):2.294661208,((37:5.639573449199999,36:5.63957345):0.5225221222,38:6.162095571):2.4766761099999997):0.08135309676,42:8.720124777):0.8762890148):1.10001754236):12.264456942999999):2.3852235814):9.942892782):0.07824339635,(((31:16.5313210856,32:16.531321087000002):3.2974516853000004,30:19.828772767):2.2671979285,29:22.095970699000002):13.271277341000001):2.85847433,(66:24.37905603,(((72:12.203965714999999,71:12.203965714):1.875539641,(((68:4.61575602,67:4.61575602):4.1129251478,69:8.7286811675):2.345991221,70:11.07467239):3.0048329663):3.128062249,(((76:6.228217194,(75:2.104206398,74:2.1042063975):4.124010796):2.623576749,77:8.851793942):6.30423019,73:15.156024133):2.051543472):7.1714884252603):13.846666352):5.31849191,(78:35.6305850727,((79:2.521272987,80:2.521272987):17.74279041,(((((82:13.472938190999997,81:13.472938191):0.5346438573,83:14.007582047699998):1.63762724,85:15.645209288):0.0154204794,84:15.660629768):0.4634945812,(87:8.658333065,86:8.6583330645):7.465791283):4.139939047):15.366521672400001):7.913629211):2.124421304,((((((((91:6.522393336,90:6.5223933361):20.264875056,89:26.787268400000002):5.778997436,88:32.5662658288):3.506098671,92:36.072364499):3.4953979,(((((101:16.8808121593,((100:11.4687124,99:11.4687124):2.343485094,(((((95:3.282672559,96:3.282672559):2.610567761,97:5.8932403199):1.425130648,98:7.3183709672):2.5152698326999996,94:9.833640801):0.08176143795,93:9.9154022387):3.896795252):3.0686146709999997):0.3778475876,102:17.2586597491):8.8453540107238,(126:19.452264242000002,((123:13.06355261985,(125:8.601643792099999,124:8.60164379168):4.4619088266):5.8695908683999996,((((((((106:9.361115876,107:9.361115877):4.228551922,((((109:0.1977558385,110:0.1977558385):6.7341996898000005,108:6.931955528):1.105557091,(111:4.648611013,112:4.648611013):3.388901607):1.271335684,113:9.308848303149999):4.2808194955):1.016289572,(115:3.851421913,114:3.851421913):10.754535459):1.118667798,(116:6.7216790814,117:6.7216790807):9.002946088):0.3072691257,118:16.031894296):0.506511974,((120:1.243103262,119:1.243103262):8.2549124327,121:9.498015695900001):7.0403905743):0.5907152904,((105:3.511430182,104:3.511430182):10.64210453,103:14.153534709999999):2.9755868486):0.5142251691,122:17.643346733):1.2897967593000002):0.519120753):6.6517495189):3.633621784,127:29.737635539000003):1.62819104,(128:9.621455673,129:9.621455673):21.7443709075):8.20193582):1.735982401,130:41.303744801):2.986068324,(132:29.736882236,131:29.736882233):14.552930901):0.7008058861,((((((135:4.033445278,(133:3.142206691,134:3.142206691):0.8912385876):0.8628826943,136:4.896327973):16.500307861,((137:3.3423734914,138:3.3423734909):16.443411709,((141:7.0543544329,(140:3.007413271,139:3.007413272):4.0469411627000005):3.2095142068,142:10.26386863929):9.521916560200001):1.6108506337000001):10.16745419,(((143:9.290119832,144:9.290119831):17.96265984,(((159:10.223318833,((((156:4.657834895,155:4.6578348945000005):2.51941374,157:7.177248634):1.516117774,(((153:0.7548637852,154:0.7548637852):6.65397907,(151:2.1217132678999997,152:2.12171326786):5.287129586):0.8828627202,(150:6.866812526,149:6.8668125269999996):1.424893048):0.4016608338):0.165670164,158:8.859036573380001):1.36428226037):7.64742486,(((146:10.726942423,145:10.726942424):4.461174869,160:15.188117293):0.8152756443,(148:14.349976228,147:14.349976227900001):1.6534167095999999):1.867350756):2.394626982,161:20.265370673):6.987408997999999):0.1912107675,((162:18.9815067302,163:18.9815067284):6.361610564,(((171:9.181102715,(((164:5.522961145,(165:0.3350073307,166:0.3350073307):5.187953814):2.627407505,(167:3.275212753,168:3.275212753):4.875155898):0.9688241001,(169:2.91051069,170:2.9105106899999997):6.208682061):0.06190996394):2.874217845,(173:3.208748362,172:3.2087483622000006):8.846572197):0.4546565766,(((180:3.493310888,181:3.493310888):1.723974856,179:5.217285744):3.040076944,(((175:4.19538511,174:4.19538511):1.73383689,(176:5.669138611,178:5.669138611):0.2600833898):0.6995825408,177:6.628804541000001):1.628558147):4.2526144482):12.8331401496472):2.100873154):4.120099575):1.413611015,((183:15.86006592,182:15.860065923799999):10.792854791,((185:2.202588999,184:2.2025889987999996):5.05899511,186:7.261584109):19.39133660629):6.3247803147):4.420837759,(188:0.5081378995,187:0.5081378995):36.890400897938704):7.5920802216):0.6780165736):6.1557773148);

TREE tree_7676 = ((((((1:15.03694401,2:15.036944010000003):2.830353618,(3:14.655738848999999,(10:13.613831177,(((6:2.985385178,5:2.9853851782999996):6.152038381,((7:5.70096384414,8:5.70096384408):1.195644224,9:6.8966080679):2.2408154903):2.638238718,4:11.775662277):1.8381688999999999):1.0419076724999998):3.2115587775999996):2.216432165,(11:10.54904186,12:10.54904186):9.534687934):4.16741489,(((16:8.217881804,(14:5.17890157,15:5.1789015699):3.0389802343):0.3751110658,13:8.5929928706):0.9334749786,17:9.526467849500001):14.724676833):3.9220657881,18:28.1732104711):23.675963023,((((((((((19:4.672666505,20:4.6726665045):10.24344546,((23:7.437112102,24:7.4371121016):6.86137448,(21:5.319128208,22:5.319128208):8.979358373):0.6176253871):2.300101345,(25:11.0923579017,26:11.09235790248):6.123855412):5.066180233,27:22.282393547399998):2.7202873509999996,28:25.002680898999998):5.430813712,(((31:13.462544990000001,32:13.46254499):2.751212814,30:16.213757803):3.35954105,29:19.5732988536):10.8601957567):3.150400186,(((52:16.755349601499997,((46:9.4835466334,47:9.4835466339):3.055404911,((51:8.314306852,50:8.3143068506):0.4973228614,(48:5.1668307974,49:5.166830797899999):3.644798915):3.727321832):4.2163980581):7.309604394000001,(((33:3.459592098,34:3.459592098):2.770449517,35:6.230041615):5.753374958,(((((36:5.323161117800001,37:5.3231611173):0.5649794235,38:5.888140541):3.05338825,((40:4.339700272,39:4.3397002724):1.912818748,41:6.2525190203):2.689009771):0.9652148851,42:9.906743676000001):1.621330514,((44:7.379106737,45:7.379106737400001):3.019426152,43:10.398532889):1.1295412997):0.4553423838):12.081537427):3.7266336716,((64:13.96742217,65:13.967422176000001):3.574955831,((54:14.46941359,(55:8.459911244,56:8.459911243):6.0095023493):0.2662669854,((((57:5.622947505,58:5.622947505):2.485054544,59:8.10800205):0.8355440067,(60:8.382441937,((61:4.5707053,62:4.570705299):2.203108274,63:6.773813574):1.608628364):0.561104119):2.690611223,53:11.63415728):3.1015233):2.806697424):10.249209671):5.792307125000001):6.752136352,(66:32.46198543,((((71:14.233272049,72:14.233272049):0.8157683011,73:15.049040351):0.3182790309,((74:0.9127693388,76:0.9127693388):9.706074389,(77:9.444389095,75:9.4443890955):1.174454633):4.748475654):1.376088499,(((68:6.062509491,67:6.062509492):2.836376166,69:8.898885657000001):4.477481824,70:13.37636748):3.3670403991000004):15.718577550000001):7.8740457159999995):5.384845364,(78:38.3239064875,(((((82:11.110350673,81:11.110350674):1.529939183,83:12.640289856799999):1.584036345,(84:12.872765838,85:12.872765839):1.351560363):0.7910189283,(86:7.908128232,87:7.9081282314):7.107216897499999):2.1649902937000003,(80:2.946240583,79:2.946240583):14.234094845000001):21.143571064):7.396970023):2.250971476,((((((((((((((97:6.984489111,(95:3.87064358,96:3.8706435810000004):3.113845531):1.2269848495,98:8.211473959100001):4.7864012773999995,93:12.997875236399999):0.02697381682,94:13.024849053):5.685721096,(99:17.08297571,100:17.08297571):1.627594436):3.6279806804,101:22.3385508289):1.438473594,102:23.7770244233):10.568275877,(126:25.458889684000003,((((((((106:11.96517125,107:11.96517125):4.58393387,(((111:5.715142942,112:5.7151429422):4.551008663,(108:8.114799923,(109:0.3570432066,110:0.3570432066):7.75775671563):2.151351683):1.869980466,113:12.136132071):4.4129730486):1.578380497,(114:3.484735135,115:3.484735135):14.642750481):1.606956041,(117:8.266196641,116:8.2661966406):11.4682450163):1.36609165,(118:20.762555843999998,((120:2.119202228,119:2.119202228):9.4898922648,121:11.609094492):9.153461351099999):0.3379774649):0.8805626761,((104:4.050875497,105:4.050875497):12.60331918,103:16.654194673):5.326901309):0.2585234291,122:22.2396194105):3.031875483,(123:20.235658869599998,(124:11.3037101817,125:11.303710181100001):8.93194868942):5.0358360257):0.18739479004):8.886410613):1.188818945,127:35.534119249):0.3474069314,(129:9.8027862482,128:9.8027862485):26.07873993155):7.438059725,(((89:27.051474136000003,(91:6.936079364,90:6.936079363899999):20.115394776000002):4.141151788,88:31.1926259334):2.194187271,92:33.3868131995):9.9327727):0.8318442485,130:44.151430149300005):2.59539968,(132:25.520256546,131:25.52025655):21.226573283):1.020836815,((((((135:4.005218555,(134:1.840523826,133:1.840523826):2.164694729):1.346584144,136:5.351802699):24.827060239,((138:6.9573239474,137:6.957323948):16.2653932148,(((139:5.5919328199,140:5.5919328202):3.604914805,141:9.196847626):7.164949258,142:16.361796888):6.860920277):6.956145782):3.185950598,(((143:8.468691139,144:8.468691139):21.42213115,(((((((155:5.562530172,156:5.562530172):2.435147288,157:7.997677461):1.626468951,158:9.624146411320002):0.1695422738,(((152:2.134028916,151:2.1340289165):3.805061554,(153:0.3126040108,154:0.3126040108):5.626486459):2.535824626,(150:7.392526707,149:7.3925267063):1.0823883886):1.31877359):2.868235395,159:12.661924078999998):7.580821525,(((146:13.339411920900002,145:13.33941192):3.637345001,160:16.976756923):2.011782505,(148:16.9555784719,147:16.955578473400003):2.0329609531):1.254206178):3.510320667,161:23.7530662743):6.137756019):1.84505515,((162:25.106982262,163:25.106982265000003):1.786287795,(((171:10.45297534,((((166:0.8303729838,165:0.8303729838):6.867882316999999,164:7.6982553010000006):1.555688631,(167:1.134192959,168:1.134192959):8.119750972):0.3568934894,(169:4.126560533,170:4.126560533):5.484276888):0.8421379197):3.099004992,(172:4.193117188,173:4.193117188):9.358863145):0.4539951506,((((((174:5.691556843,175:5.691556843):0.1093025672,176:5.80085941):0.2313468463,177:6.032206257):1.466535309,178:7.498741566):0.5319090843,179:8.030650649):2.33580488,(181:5.967175021,180:5.967175021):4.3992805090000004):3.6395199536):12.8872945749102):4.842607378):1.628936103):2.689670011,((183:20.39236701,182:20.392367013299996):5.789625192,(186:7.453808632,(184:1.646896871,185:1.6468968713):5.8069117596):18.7281835736):9.872491345):5.540016326,(188:0.5101876682,187:0.5101876681999999):41.084312213184695):6.173166769):0.2041813413):3.8773255073999997);

TREE tree_6626 = ((18:29.664769784700002,(((11:8.724018556,12:8.724018556):10.337405828,((1:14.51290368,2:14.512903683):1.874860825,(((4:11.647382845,((6:2.291734058,5:2.2917340577):6.637227218,((8:4.79380904035,7:4.79380904):1.002253171,9:5.7960622122):3.13289906418):2.7184215691):1.374320558,10:13.0217034006):1.024273215,3:14.045976616599999):2.3417878907):2.673659876):3.745695564,((((14:3.598670714,15:3.598670714):3.6836240047000004,13:7.2822947198):0.7110218095,16:7.993316528):0.5407161554,17:8.534032683):14.273087264199999):6.857649827205):20.6166038688,(((((((30:15.014860937,(32:12.827917802999998,31:12.8279178026):2.1869431348):2.4897075547,29:17.504568491999997):8.948591797999999,(((((19:3.9752036428000004,20:3.9752036426):8.257394948,((23:7.254400469,24:7.2544004694):4.592148969,(21:5.05814975,22:5.058149751):6.788399688):0.3860491525):2.377837828,(25:9.299429029499999,26:9.299429029799999):5.3110073898):4.027993047,27:18.6384294613):1.258186124,28:19.896615589):6.5565447006):5.484478576,(((64:6.150071769,65:6.1500717691):1.511465277,(((((57:3.218599945,58:3.2185999448000002):0.5869284227,59:3.805528367):0.4558344519,(63:3.314665047,(60:3.270941293,(61:1.601665342,62:1.6016653416):1.669275951):0.04372375411):0.9466977722):1.420405447,((55:3.710739319,56:3.7107393184):1.4948931887999999,54:5.205632508):0.4761357587):0.7568484271,53:6.438616693):1.222920352):15.6591373545,((52:14.741965283699999,(((49:4.970830601,48:4.970830601):2.466841148,(51:7.437040729,50:7.4370407290000005):0.000631019626):2.480483481,(47:8.645273409,46:8.645273408):1.27288182096):4.823810055):5.871171483,((((44:6.247363373,45:6.2473633739):2.043387834,43:8.2907512079):1.58265481,((((39:4.346595715,40:4.346595715):1.082555327,41:5.4291510421):2.730625438,((36:4.656191778,37:4.656191778):1.475968434,38:6.132160212):2.027616268):0.5312082646,42:8.6909847451):1.1824212722999998):1.126712284,((33:4.004971858,34:4.004971858):2.763554609,35:6.768526466999999):4.231591835):9.6130184652):2.7075376330000003):8.616964464):3.5970640951000004,(66:29.78223782,((((((68:5.562922361,67:5.5629223612):4.384449833,69:9.9473721939):0.6603250152,70:10.60769721):2.585751575,((74:1.560656341,76:1.560656341):6.265755057,(77:4.503484968,75:4.5034849682):3.32292643):5.367037387):0.9060247959,(71:10.994461952,72:10.994461953):3.105011629):0.4248941942,73:14.524367778000002):15.257870043799999):5.752465139):6.625695961,(((((83:11.945309865499999,(81:9.834658992000001,82:9.8346589922):2.1106508734):1.384867059,(84:12.945907684,85:12.945907682000001):0.3842692418):2.152791706,(87:8.107895649,86:8.107895649):7.3750729816):3.632602076,(80:1.932868539,79:1.932868539):17.182702164):19.170154255,78:38.285724962):3.8746739583000003):2.123108486,(((((((((101:19.31415381,(100:16.10474623,(((93:11.6199310072,((97:7.151637799,(95:5.22867708,96:5.22867708):1.922960718):1.2071241021999999,98:8.358761900400001):3.2611691065599997):0.2139504429,94:11.833881450029999):2.976985144,99:14.81086659):1.293879631):3.209407585):1.5885097624,102:20.902663573799998):6.301157837,((((((105:2.885288756,104:2.885288756):11.60220736,103:14.487496113):3.3285681032000003,((((((106:9.436950681,107:9.4369506809):4.788213382,(((108:7.400810571999999,(109:0.3590566455,110:0.3590566455):7.0417539273):1.276673296,(111:4.66141346,112:4.66141346):4.016070409):1.465504879,113:10.1429887472):4.082175315100001):1.032883197,(115:3.107461157,114:3.107461157):12.1505861031):0.8224265317,(117:7.114230518,116:7.1142305174):8.966243274):0.742702366,118:16.823176157):0.3244023327,(121:8.9235202847,(120:1.43219973,119:1.43219973):7.491320556):8.2240582045):0.6684857243):0.1497311927,122:17.9657954053):1.5694288,((125:8.3813326581,124:8.3813326585):7.455258795700001,123:15.8365914526):3.6986327544):0.6025364351,126:20.137760643):7.066060767):2.006643717,127:29.210465132000003):0.7638206671,(128:7.9334688612,129:7.9334688617):22.04081692816):8.906898981,(((88:29.676219247,(90:8.3769109213,91:8.376910921):21.299308326000002):0.2068970603,89:29.88311631):3.905133703,92:33.78825001):5.092934764):2.421370348,130:41.302555118):1.910198033,(132:28.258113028000004,131:28.25811303):14.954640118):0.2714158082,((((((135:2.928154454,(133:1.58522419,134:1.58522419):1.342930264):1.114020289,136:4.0421747429999995):13.369995963,(((141:5.6465131979,(140:3.98789833,139:3.9878983301999997):1.6586148674999999):4.966400867,142:10.612914064000002):4.268852064,(137:4.804792985700001,138:4.8047929855):10.076973143):2.530404578):13.8132849,(((143:8.368427456,144:8.368427456000001):19.55064077,(((145:14.426187841,146:14.426187837):5.454540678,(((147:15.210089227,148:15.2100892256):2.7039059341,160:17.91399516):0.5918330811,((((157:7.8841741988,(155:4.339593996,156:4.339593996):3.5445802027):1.498534237,158:9.3827084345):0.7011807783,(((151:1.5061796307000002,152:1.5061796309000002):4.814663098,(154:0.9149724634,153:0.9149724634):5.405870266):3.111924963,(150:6.052453267,149:6.052453267):3.380314425):0.6511215217):1.309924701,159:11.393813915):7.112014326):1.3749002730000002):3.125212387,161:23.005940898):4.913127321):0.4926071777,((162:15.270398492,163:15.270398496):10.17121617,(((171:6.576907249,(((164:3.77334224,(166:0.5813145562,165:0.5813145562):3.192027684):0.7530484368,(168:1.399111917,167:1.3991119160999999):3.1272787602000003):1.157376278,(170:1.206931695,169:1.2069316957):4.476835259):0.8931402942):0.9714173814,(172:1.5017929,173:1.5017929):6.04653173):0.8520705729,((179:4.261423924,(((176:2.402448512,(174:1.765295424,175:1.765295424):0.6371530889):0.79775766201,177:3.2002061745):0.5433208303,178:3.7435270043):0.5178969192):0.7683210015,(180:2.716146935,181:2.716146935):2.313597991):3.3706502784000003):17.04121946230514):2.970060739):2.813780213):0.8265348409,((183:17.80978053,182:17.8097805299):3.261954186,(186:5.6656217112,(185:2.975499502,184:2.975499502):2.69012220905):15.4061130055):10.980255744):3.378827931,(188:0.1954065089,187:0.1954065089):35.235411875800004):8.053350575):0.7993384477):5.9978662400000005);

TREE tree_4650 = (((((11:10.30562321,12:10.30562321):10.498197379,((1:17.26873771,2:17.26873771):0.8138910475,(((((5:3.156940707,6:3.15694070653):7.584255764,((8:6.030178467,7:6.030178467509999):0.5299391587,9:6.560117625499999):4.181078845):1.221310935,4:11.962507406):2.44066143,10:14.403168834):0.7748749397,3:15.1780437753):2.904584982):2.721191835):2.817630858,(((16:7.300432732,(15:4.108238845,14:4.108238845):3.1921938866999997):1.3198050207,13:8.6202377522):0.9588454859,17:9.579083238599999):14.0423682155):4.3321742696,18:27.953625720200005):23.879414673,((((66:23.65524843,(((72:10.32015372546,71:10.320153726):1.988163973,((74:6.904648006,((76:3.112585307,77:3.112585307):1.4903373923,75:4.602922700000001):2.301725306):4.845831514,((69:8.653430449,(68:4.380854453,67:4.380854452):4.2725759964):0.5865120895,70:9.239942539):2.5105369811):0.5578381783):1.252210984,73:13.560528681):10.0947197477):14.354396718,((((30:16.317658359,(31:14.6067337026,32:14.606733703300002):1.7109246553):1.873206131,29:18.1908644894):12.555540121,((((26:11.3275359501,25:11.32753595):6.7011789832,(((24:9.494104179499999,23:9.49410418):5.53149666,(21:6.750962902,22:6.7509629019999995):8.2746379392):0.7467215412,(19:5.038169986,20:5.0381699861):10.7341524):2.2563925520000003):5.213755803,27:23.242470741119998):2.507039223,28:25.7495099585):4.9968946508):3.224645183,(((((34:2.874158645,33:2.874158645):1.945935334,35:4.8200939787):4.184926582,((43:6.362769525,(45:5.261017477299999,44:5.261017477):1.101752048):2.103000307,((((39:3.529441136,40:3.529441136):1.935819138,41:5.465260274):1.596793589,((37:4.0157764961,36:4.0157764961):0.8145368925,38:4.8303133881):2.231740475):0.2853660403,42:7.3474199038):1.1183499284):0.5392507291):7.524667024,(52:10.857340646699999,((((49:3.102199421,48:3.1021994211000004):2.82966009,50:5.9318595111):0.1240064722,51:6.05586598433):2.853596495,(46:7.72173941,47:7.7217394100000005):1.1877230692):1.947878169):5.6723469369999995):2.1811905352,((64:6.054377921,65:6.0543779214):1.412884173,(((((60:3.868235318,(61:2.702353293,62:2.702353293):1.165882025):0.4998272884,63:4.368062607):0.2136208403,((57:2.227671664,58:2.2276716645):2.2684769,59:4.496148564):0.08553488226):1.081629369,(54:5.111631235,(55:3.038874961,56:3.038874961):2.0727562742):0.55168158):0.619174801,53:6.2824876166):1.184774478):11.2436160296):15.260171674299999):4.0385953489999995):4.474203102,(78:34.793861569,((80:3.501045546,79:3.501045546):16.85865732,(((((82:11.127341872999999,81:11.1273418727):1.609380584,83:12.736722458300001):1.935969508,85:14.672691966):0.5715719357,84:15.244263902):1.763418776,(86:7.127284256,87:7.127284256):9.8803984215):3.3520201894900006):14.434158702):7.689986682000001):1.725935163,((((((((90:7.8161402744,91:7.816140273999999):22.061975921,89:29.878116191):0.409662246,88:30.28777843855):4.229398372,92:34.5171768119):6.838767861,(((((101:16.9994968928,(((94:8.677999788,93:8.677999787000001):0.7432155836,(((95:3.470011577,96:3.470011577):2.148300676,97:5.6183122535):0.988078763,98:6.6063910159):2.8148243546000002):4.573311829,(99:12.55272056,100:12.55272056):1.441806643):3.0049696944999997):0.786154415,102:17.7856513093):9.2218402811,(126:19.312138659,((((103:13.63011868,(104:3.472815968,105:3.472815968):10.1573027103):3.4734943719999998,((((((106:10.350299731,107:10.3502997313):3.14309695,(((111:4.601684577,112:4.601684578):3.000663411,(108:6.692579248,(109:0.404480587,110:0.404480587):6.2880986611):0.9097687394):2.165485391,113:9.767833379599999):3.7255633023000003):1.133618326,(114:2.484099163,115:2.484099163):12.142915844000001):0.4491988402,(117:5.720042867,116:5.720042867099999):9.356170980800002):1.063007873,118:16.13922172):0.3372179641,((120:1.425239491,119:1.425239491):7.2523141741,121:8.6775536652):7.798886019):0.6271733671):0.2664325318,122:17.3700455792):1.487843664,(123:14.7467447951,(124:11.2945597091,125:11.2945597094):3.4521850855):4.1111444532):0.4542494112):7.695352932):9.389340553,127:36.39683214):0.133099828,(128:12.331973947,129:12.3319739508):24.19795802384):4.826012698):1.758583322,130:43.11452799400001):0.5194868348,(131:34.13811528,132:34.138115285):9.49589955):0.1065595084,((((((143:8.320562162,144:8.320562162):16.29742343,(((((145:11.77644194,146:11.7764419403):2.787114945,160:14.563556885):1.489701944,(147:14.8421965903,148:14.8421965897):1.2110622390999999):0.8479799424,(((((151:2.0880961901,152:2.08809618936):4.027967703,(154:0.442120766,153:0.442120766):5.673943127):1.032478,(150:6.024654765099999,149:6.024654765):1.123887128):1.269564211,(158:8.12002949557,((155:3.009825545,156:3.009825545):3.277619156,157:6.287444700999999):1.8325847941000002):0.2980766088):1.709351707,159:10.127457809900001):6.77378096):2.062850501,161:18.964089272000002):5.653896324):0.6658510439,((162:12.873077607,163:12.873077611800001):9.767162409,(((171:8.489752441,(((164:5.743172545,(165:0.556703377,166:0.556703377):5.1864691683):0.8057680705,(167:2.180346683,168:2.180346683):4.368593932):0.5933385283,(169:2.337042754,170:2.3370427538):4.805236389):1.347473297):1.600213139,(172:4.162348608,173:4.162348608):5.927616972999999):0.477512848,(((((174:4.264818066,(175:3.794971153,176:3.794971153):0.469846913):0.8447827355,177:5.109600802003):0.8699696556,178:5.9795704576999995):0.8519664439,179:6.831536901):1.543254428,(181:2.929605212,180:2.9296052119):5.445186117):2.1926870997):12.0727615878):2.643596625):3.04370276,((183:12.45468527,182:12.454685265):7.183847073,(186:5.295163115,(185:2.443361085,184:2.4433610851):2.8518020303):14.343369223700002):8.689007062):0.8993625558,(((138:9.2247816545,137:9.224781654800001):9.434965753,((141:4.671597994,(140:4.194086524,139:4.194086523):0.47751147101):6.165290105,142:10.836888099000001):7.822859308):2.2066215404,(((133:2.410778197,134:2.410778197):0.1523552946,135:2.563133491):0.6388478007,136:3.201981292):17.664387656):8.3605330118):6.2810533,(187:0.7578660582,188:0.7578660582):34.7500892027):8.232619076999999):0.4692090751):7.623256978000001);

TREE tree_2784 = ((18:33.010525067859994,((((1:19.48825242,2:19.488252422):1.345054923,(3:18.020415277999998,(((((8:7.6409271389,7:7.6409271401):0.7278410558599999,9:8.368768194669999):2.736799303,(6:2.747084635,5:2.747084635):8.358482864):3.209810586,4:14.315378085):2.128383953,10:16.443762042):1.5766532402):2.812892067):3.370512002,(11:11.29511515,12:11.29511515):12.908704200999999):5.281458325,((((14:4.584746874,15:4.584746874):5.2710241159999995,13:9.8557709896):0.3182946617,16:10.174065652):0.7493702495,17:10.923435901200001):18.561841771):3.5252473969):20.704670291999996,((((66:38.37829403,((((((76:5.608184085,77:5.6081840849999995):0.5683788118,75:6.176562897):2.199863168,74:8.376426064):3.641783062,((69:8.66449379493,(67:7.099743737600001,68:7.0997437377):1.5647500571):0.9166826787,70:9.581176473):2.4370326535):0.3837996857,(72:9.6372971435,71:9.637297142):2.7647116691):3.436373895,73:15.83838271):22.539911318999998):3.3354322804,(((28:21.765687768099998,(((((21:4.8562927,22:4.8562927):7.1783753101,(23:6.311299363,24:6.3112993629):5.723368648):0.2449000422,(19:4.168907655,20:4.1689076551):8.110660398):2.473915608,(25:9.594797298,26:9.594797298400001):5.1586863629):4.90708887,27:19.660572530400003):2.1051152387):3.931846761,((30:13.556295336,(31:11.8676054625,32:11.867605462):1.6886898750000001):2.933100261,29:16.4893955972):9.208138931699999):9.943034336,(((64:7.796435101,65:7.7964351012):2.115475308,(53:8.208921156,(((((61:2.596529856,62:2.596529856):0.9671823725,63:3.563712229):0.4025255216,60:3.96623775):0.436330982,((57:2.807306474,58:2.8073064734):0.8304708986,59:3.6377773724):0.7647913601):2.688790711,(54:6.36547875,(55:3.253839718,56:3.2538397182):3.1116390320000002):0.7258806935):1.117561713):1.702989254):10.74858325541,((((33:2.905979326,34:2.9059793261049998):2.327523507,35:5.2335028342):3.924947194,(((((40:3.437450163,39:3.437450163):2.39523171,41:5.8326818726):1.154004945,((36:4.9592196637,37:4.9592196644):0.2277663131,38:5.186985977):1.79970084):0.3071711673,42:7.293857983900001):1.069574399,((44:5.1843415212,45:5.1843415208):1.532347967,43:6.716689489):1.6467428952):0.7950176445):10.366801516999999,(52:12.1677793891,((47:6.9855982050000005,46:6.985598205):2.821587282,((49:3.496164256,48:3.49616425581):3.357935083,(51:6.7053318514,50:6.7053318520000005):0.1487674878):2.9530861484999997):2.3605938980000003):7.357472158):1.135242125051):14.980075201):6.073157442):3.034965242,(78:34.149601731,((((((81:10.1862006307,82:10.1862006305):0.459643104,83:10.6458437354):3.189862214,85:13.835705949000001):0.733272901,84:14.56897885):0.7410903012,(86:9.857710109,87:9.857710109):5.4523590414):0.8315376868,(80:1.698659741,79:1.6986597409000002):14.4429471):18.007994894):10.599089819):2.352305695,(((131:35.113364969,132:35.113364966999995):10.52899968,((((((((((((97:7.009994826,(95:5.317674687,96:5.317674688):1.692320139156):1.216425815,98:8.2264206411):2.5535288149,93:10.7799494564):0.417566282,94:11.1975157384):5.17343659,(100:14.21041512,99:14.21041512):2.160537208):3.2111330139999996,101:19.5820853436):1.36384632,102:20.9459316609):6.642638299,((((((105:4.235137788,104:4.235137788):9.82060535,103:14.055743141999999):2.876504181,(((((((((109:0.2495201322,110:0.2495201322):6.281961690999999,108:6.531481823):1.393016595,(111:3.904962778,112:3.9049627780000002):4.01953564):2.758404417,113:10.6829028362):3.3545026441,(106:10.92997765,107:10.9299776509):3.10742782715):0.4595853738,(114:3.924227856,115:3.9242278554000003):10.572762997):1.045596128,(117:7.3924571503,116:7.3924571496):8.150129832000001):0.9780157818,118:16.520602763):0.161081392,((120:1.195161859,119:1.195161859):8.2877632957,121:9.4829251544):7.19875900018):0.25056316453):0.7735526317,122:17.70579994655):1.364359941,(123:15.0651130782,(124:9.735156999,125:9.7351569993):5.32995607806):4.0050468143):0.3112867284,126:19.381446616999998):8.207123340099999):3.387731047,127:30.976300997):0.1094426124,(128:8.685158623,129:8.6851586219):22.4005849974):9.091344643,((((90:7.1631586181,91:7.163158618000001):17.25556555,89:24.4187241632):3.472447571,88:27.891171738999997):3.404540363,92:31.2957121011):8.881376161):2.597127884,130:42.774216141100005):2.868148506):0.1644595319,((((((143:6.179044091,144:6.1790440909):18.54777668,(((((147:14.17308042,148:14.173080419700002):1.0181031388900001,160:15.1911835598):0.6562115772,(146:12.170824824,145:12.170824828499999):3.676570312):0.8846498281,((((150:7.828448285,149:7.8284482851):0.9848065599,((152:2.0711642134,151:2.0711642126):2.995666092,(154:0.6708732965,153:0.6708732965):4.395957008):3.74642454):0.5564365561,((157:7.7513102629,(155:3.9372443249,156:3.937244324):3.8140659389000002):1.337654113,158:9.0889643767):0.2807270246):0.9125138377,159:10.282205238600001):6.449839726):2.09840243,161:18.830447395):5.896373375):1.509983951,((162:17.4051205962,163:17.4051205904):7.364140635,((((((164:4.349421287,(166:0.5076438866,165:0.5076438866):3.841777401):2.426487581,(168:1.895596395,167:1.895596395):4.880312473):1.12975063,(170:2.329330547,169:2.3293305469):5.576328952):0.5995632214,171:8.50522272):2.115639432,(172:3.719108037,173:3.719108037):6.901754115):0.2542190792,((179:5.50707589,((177:4.1094419252,(176:2.477565088,(175:2.359235187,174:2.359235187):0.1183299015):1.631876837):0.4657239151,178:4.57516584):0.9319100496):2.17075541,(180:4.069881972,181:4.069881972):3.607949328):3.197249931):13.8941799988):1.467543492):4.81278006,((183:21.65946208,182:21.659462083999998):1.433041535,(186:9.868104778,(184:3.8698543443999998,185:3.869854344):5.998250433299999):13.224398840700001):7.9570811608478):1.400655219,((136:6.508565478,(135:3.678159745,(134:1.769753637,133:1.769753637):1.908406108):2.8304057333):15.709568918999999,((137:7.565196158,138:7.565196158000001):12.3239266143,(142:13.165128259,((140:4.7805928908999995,139:4.7805928906999995):1.766742496,141:6.5473353863):6.6177928732):6.72399451):2.3290116274):10.2321056):5.439166504,(187:1.717317382,188:1.7173173823):36.172089132400004):7.9174176747):1.294173064):6.614198120099999);

TREE tree_8920 = ((((((1:14.13761851,2:14.137618514):0.7306124289,(((4:9.733469264,((5:2.248515312,6:2.248515312):5.4583125,((7:3.5856896223,8:3.58568962242):0.7037147474000001,9:4.289404370200001):3.4174234412):2.0266414527):1.666274065,10:11.39974333):0.5519402418999999,3:11.951683572):2.916547372):0.7769065744999999,(11:6.706495072,12:6.706495072):8.938642445):3.352548734,((16:7.191853648,((14:3.5307121849999996,15:3.5307121851):2.451063403,13:5.9817755883):1.2100780601):0.1960415101,17:7.387895158099999):11.6097910929):3.6756519191,18:22.67333816835):32.256214889,(((((((((43:14.30200001,(45:10.630010509,44:10.630010509):3.671989505):0.9373561583,((((40:4.52231075,39:4.522310751):2.393654376,41:6.915965126):3.948404576,((36:7.317062764999999,37:7.317062765999999):1.149070604,38:8.4661333698):2.398236332):1.902208725,42:12.76657842757):2.472777746):1.525529996,((33:4.673176452,34:4.673176452):4.223729121,35:8.896905573):7.867980596):15.0882550201,(52:22.771459646300002,(((51:10.297415768,50:10.297415767):0.1091747482,(49:5.438206125,48:5.438206126):4.968384391):5.451318428,(47:10.53694516,46:10.536945158999998):5.320963786):6.913550706):9.081681538):3.8726898315000002,(29:21.6281817823,(30:18.940174715,(32:16.557712383,31:16.557712383000002):2.3824623336000004):2.6880070642):14.097649238):0.01041099549,((((((19:5.221775901,20:5.2217759011):11.56345719,((21:5.609304947,22:5.609304946):9.419992309000001,(24:10.654767830199999,23:10.65476783):4.374529424):1.755935832):1.684576261,(25:10.142282402,26:10.142282401):8.327526946999999):7.514854251,27:25.9846635974):1.949691256,28:27.934354855):6.2152877250300005,((64:9.608429919,65:9.608429918):3.925836291,((54:9.488356601,(55:5.286748362,56:5.286748362000001):4.2016082393000005):1.206570144,(53:10.28939345,((63:6.757373912,((61:4.651513116,62:4.651513116449999):1.785800523,60:6.43731364):0.3200602727):1.463941538,((57:3.676326998,58:3.6763269973):3.647984031,59:7.324311029):0.8970044225):2.068078004):0.4055332898):2.839339464):20.615376371):1.586599432):5.1574151144,(66:35.07133682,((((70:11.08356708,((68:5.715225299,67:5.715225298):5.178933945,69:10.8941592427):0.1894078321):5.171780993,(((76:4.905276748,75:4.905276748):3.93252007,77:8.837796818000001):6.199688025,74:15.03748484):1.21786322438):0.2579344543,(72:13.374274606,71:13.374274604):3.139007916):0.6100519857,73:17.123334507000003):17.9480023123):5.822320305):3.743635597,(78:39.6146501282,((((((82:12.224465764789999,81:12.224465766):0.9163686164,83:13.1408343827):1.547646564,84:14.688480945699999):0.1391629366,85:14.827643882):1.398899665,(87:7.672798548,86:7.672798547999999):8.5537450004):1.6904244,(80:1.617507246,79:1.617507246):16.2994607):21.697682189):5.022642599):4.679075523,(((((((89:26.16311611,(90:8.361619752,91:8.361619752):17.8014963615):3.782653857,88:29.9457699659):3.97545373,92:33.9212236927):9.559135623,(((((101:20.550297166,((99:14.89090359,100:14.89090359):2.970288084,((93:10.4340524746,(((95:4.291234266,96:4.291234266):2.93641363,97:7.2276478959):0.128152198,98:7.355800094000001):3.07825238035):0.198597639,94:10.632650114):7.228541561):2.689105488):1.91265118,102:22.4629483427):6.365284877,((((((118:18.4969854975,(121:8.6709819465,(119:1.432066884,120:1.4320668833899999):7.238915064):9.8260035505):0.2086634723,((((106:11.00414237,107:11.004142367):4.025182097,(((111:5.103412817,112:5.103412818):3.738695417,(108:6.273904256,(109:0.4292803628,110:0.4292803628):5.8446238926000005):2.568203977888):2.864501307,113:11.7066095401):3.3227149238):0.9094040354,(115:2.036605095,114:2.036605095):13.902123405):0.6598969915,(117:7.48396813,116:7.4839681301):9.114657360999999):2.107023479):0.3428818057,122:19.0485307804):0.4370890258,(103:15.575931508,(104:3.130087726,105:3.130087726):12.44584378):3.909688296):1.411901464,((125:10.281385539999999,124:10.2813855397):6.19635590202,123:16.477741441974):4.419779824):0.5497945343999999,126:21.4473158):7.3809174201):2.625173061,127:31.4534062786):2.355303627,(128:10.106872877399999,129:10.10687287617):23.701837033):9.671649408):1.975136733,130:45.45549604599999):1.46190902,(131:29.155433191,132:29.155433195):17.76197188):1.251606638,((((((143:6.816024928,144:6.816024929):17.29533944,((((((((156:4.287052222,155:4.287052222):2.561452233,157:6.848504456):1.621990526,158:8.47049498078):0.3853436888,(149:5.883801889,150:5.883801889):2.972036781):0.09685687264,((154:0.1728226291,153:0.1728226291):6.029887333,(151:1.8259963235,152:1.8259963234):4.3767136386):2.749985581):2.035836811,159:10.9885323542):5.529931177,((145:10.954274028,146:10.954274028):4.957911354,((148:13.465692112,147:13.465692109999999):1.6144787407,160:15.080170852):0.832014529):0.6062781496):2.282184178,161:18.800647713100002):5.310716657):1.505459644,((162:15.964562079,163:15.96456207991):6.027238563,(((171:10.29759979,(((164:6.509662003,(166:0.7140437995,165:0.7140437996):5.795618203):0.444479738,(168:1.732545531,167:1.732545531):5.2215962099999995):2.010302395,(169:1.252958068,170:1.252958068):7.711486067299999):1.333155653):2.732837345,(172:4.135156488,173:4.135156488):8.895280645):0.2884772647,(((((174:5.709440986,177:5.709440986000001):0.3561853888,(176:4.696947379,175:4.696947379):1.3686789964):0.4940534269,178:6.559679802):1.105705866,179:7.665385668):1.898172911,(181:5.270782796,180:5.270782796):4.292775783):3.75535582):8.672886237):3.625023371):7.70636626,((183:19.12617114,182:19.1261711411):2.352040223,((185:1.930706232,184:1.930706232005):5.333712465,186:7.2644186976):14.213792665):11.844978907000002):2.63804814,(((135:4.600408349,(133:3.357153501,134:3.3571535007):1.243254848):0.3620465736,136:4.962454923):19.508605637000002,((138:7.3498253583,137:7.3498253572):15.1370512004,((141:7.8432460842000005,(139:4.42732385,140:4.42732385):3.4159222337):6.334104515,142:14.177350602999999):8.3095259612):1.98418399645):11.49017785):4.519322162,(187:0.03207192802,188:0.03207192802):40.4484886345):7.6884511396):1.147356543):5.613184802);

TREE tree_7753 = ((18:19.775468240339997,(((11:6.185828834,12:6.185828834):9.080384898,((1:12.9888525,2:12.988852498):1.043888841,(3:11.808358848000001,((((6:2.731725351,5:2.7317253511999997):5.6199141572,((8:4.2814400418,7:4.28144004172):1.5443366044000002,9:5.8257766453899995):2.5258628634):1.210545948,4:9.562185458):1.373922924,10:10.936108381999999):0.8722504667):2.2243824905):1.2334723928):2.759574587,(((13:6.2368482691,(15:2.953454884,14:2.953454884):3.28339338489):0.06597213347,16:6.302820403):0.9709922182,17:7.273812621100001):10.7519756969):1.749679921):25.156531092999998,(((((((64:6.652263842,65:6.6522638419):3.078800972,((53:4.941320791,((((61:2.405261305,62:2.4052613055):0.5836021811,63:2.988863486):0.4049205866,60:3.393784073):0.3626458284,((57:1.756612186,58:1.7566121863):1.179554499,59:2.9361666851):0.8202632161):1.18489089):2.426218209,(54:6.886074531,(56:4.619881196,55:4.6198811951000005):2.2661933358):0.4814644697):2.363525813):12.149631708343,(((((19:4.007570087,20:4.00757008748):9.010660531,((22:5.244750614,21:5.2447506141):6.920800451,(23:6.181795821,24:6.1817958206):5.983755246):0.8526795519):1.785866159,(25:8.648602753,26:8.648602752799999):6.155494023768):2.997579003,27:17.801675781):1.630187747,28:19.431863527999997):2.4488329920000003):2.497651438,(((((34:6.113570508,33:6.1135705080000005):1.557510896,35:7.6710814044):3.834807113,(((((40:5.566253032,39:5.566253032):0.7359438563,41:6.3021968887):2.014522229,((37:6.0404164399999996,36:6.04041644):0.2964343317,38:6.336850772):1.979868345):0.2337959698,42:8.550515087299999):1.251770674,((45:7.4528245956,44:7.452824595):1.340638532,43:8.793463127):1.0088226344):1.703602756):10.2270622556,(52:15.4168209132,((47:8.088925967,46:8.088925967):3.446598682,((51:7.395307336,(49:4.188535473,48:4.188535473):3.2067718624):0.8742655984,50:8.269572934000001):3.265951715):3.8812962607):6.316129864):2.0890888566,((30:12.352751293,(32:10.752413504,31:10.7524135044):1.6003377874):3.383728724,29:15.7364800171):8.085559613000001):0.5563083301):7.303961986,(66:23.08257485,(((71:9.2224673249,72:9.2224673248):2.205285485,((70:9.367526087,((67:3.8939780257,68:3.8939780261):3.1957368587,69:7.0897148845):2.277811203):1.564389334,((74:4.851446625,76:4.851446625):4.086134313,(75:2.189554094,77:2.1895540934):6.748026844):1.9943344843):0.4958373881):0.4570366374,73:11.884789443999999):11.1977854022):8.599735092):5.371372898,(((((((81:9.05441691,82:9.054416910099999):0.6454038907,83:9.699820801):1.513237671,84:11.213058472):0.5178238927,85:11.7308823644):1.156161299,(87:7.691928157,86:7.691928157):5.1951155057):2.743968371,(80:2.848404056,79:2.848404056):12.782607978):14.925831002099999,78:30.5568430412):6.496839804):2.090070496,(((((((((((((94:8.20193892,(((95:2.720902881,96:2.7209028809999998):1.982247403,97:4.703150283):1.3312898227,98:6.0344401071):2.1674988143):0.4353919088,93:8.6373308295):1.948730934,99:10.58606176):1.298155085,100:11.88421685):2.6952808474,101:14.579497694300002):1.0499372,102:15.629434894300001):5.374080226,((((((((((106:7.225689596,107:7.2256895963):3.3150334703,(((111:3.491419832,112:3.4914198317):2.145885121,(108:4.4773923058,(109:0.2138809663,110:0.2138809663):4.26351134032):1.159912647):1.240139774,113:6.8774447266):3.6632783399999997):0.3231651574,(114:2.086363074,115:2.086363074):8.777525149):0.9119454719,(117:4.9800190786,116:4.9800190781):6.795814618):0.9821684745,((120:1.30826851,119:1.30826851):5.9780878787,121:7.286356389):5.4716457819999995):0.04402477136,118:12.802026942):0.19725771704,((104:2.52964559,105:2.52964559):7.87178287,103:10.401428459):2.597856199):0.9446878592,122:13.943972519599999):0.9116326114,(123:12.100303059500002,(125:6.226435421,124:6.226435421):5.873867639193):2.7553020697):1.28832834378,126:16.143933474):4.8595816460000005):4.548774427,127:25.5522895498):0.8806581381,(129:7.2632455058000005,128:7.263245506):19.1697021838):8.390766175,((((90:6.153807631,91:6.153807631):15.393857736000001,89:21.547665363):2.026949636,88:23.5746150004):2.766003217,92:26.3406182152):8.483095642):2.26192705,130:37.085640909):0.6624949919,(132:29.036869175,131:29.03686918):8.711266723):0.4330741523,((((((143:6.775100182,144:6.775100182000001):13.6373278,(((((145:11.243700472999999,146:11.2437004782):1.966775656,160:13.210476133):0.829991102,(147:11.939008407,148:11.939008407000001):2.101458829):0.5755112489,((((((154:0.704899393,153:0.704899393):3.942603811,(152:1.5287625209,151:1.5287625211):3.1187406827):2.400362788,(150:3.926388806,149:3.926388806):3.121477187):0.5681471454,((156:2.401399326,155:2.4013993254):2.73846737,157:5.1398666959999995):2.4761464424):0.45244357,158:8.06845670792):0.7659833341,159:8.834440042):5.781538442):1.464567915,161:16.0805463988):4.331881581999999):1.881199499,((162:13.976636998,163:13.9766370032):3.361993629,((((((164:5.796352279,(165:0.5106725644,166:0.5106725644):5.2856797140000005):2.040996238,(167:2.676071431,168:2.676071431):5.161277087):0.7791608799,(169:1.73800226,170:1.73800226):6.8785071361):1.533173712,171:10.14968311):0.8752852145,(172:4.015795732,173:4.015795732):7.0091725913):0.4651697403,((179:6.928187858,(((176:4.034373365,(175:3.479616149,174:3.479616149):0.5547572151):0.5705220173,177:4.6048953817000005):1.206028266,178:5.810923647999999):1.11726421):1.228864712,(181:3.33364759,180:3.333647591):4.82340498):3.333085494):5.8484925634):4.954996855):3.109745788,((182:17.28119211,183:17.28119211):2.046477203,((185:3.18857122,184:3.1885712199):2.941591162,186:6.13016238165):13.1975069331):6.075703958):1.916027679,(((138:4.597198768,137:4.5971987678):13.4910796813,(((140:2.692699481,139:2.692699482):2.993851352,141:5.6865508338):5.565035405,142:11.2515862379):6.836692211000001):3.167450439,((135:2.549041801,(134:2.000913553,133:2.000913553):0.5481282478):0.9696846686,136:3.51872647):17.737002416000003):6.0636720609):5.513814921,(188:0.3180837393,187:0.3180837393):32.515132126):5.347994187):0.96254328):5.7882459943);

TREE tree_9126 = ((18:27.4147267426761,(((11:9.158433733,12:9.158433733):11.034348498,((1:17.76057582,2:17.760575827):0.6217609911,(((((8:4.97916847083,7:4.979168470499999):0.337249528,9:5.3164179991000005):4.987425284,(5:2.39039397,6:2.39039397):7.913449313599999):1.13958924,4:11.443432523):3.214943169,(10:13.927504521000001,3:13.927504520600001):0.7308711712):3.7239611229999996):1.8104454153999998):4.33365372,(((13:7.735783021,(15:4.465534348,14:4.465534348199999):3.2702486716):0.4319783227,16:8.167761343):0.268108015,17:8.4358693579):16.0905665897):2.8882907887):23.6794520548,((((66:36.18099657,(((((76:7.859143523,(77:7.030249087,75:7.030249087):0.8288944354):1.073287246,74:8.932430769):4.352109596,73:13.28454036762):0.3208645557,(72:12.460560665,71:12.460560663999999):1.144844255):3.364447528,((69:10.63628509718,(68:6.454735063,67:6.454735063):4.181550034):1.691428987,70:12.327714083):4.6421383639):19.211144123):3.3475479407,((((((((24:9.2878275054,23:9.287827505):6.954931818,(21:6.757381411,22:6.757381411000001):9.485377912099999):0.6214349593,(19:5.908988644,20:5.9089886443):10.955205637):1.577233416,(26:12.877156544760002,25:12.8771565436):5.564271155):6.209445395,27:24.650873099000002):2.98843294528,28:27.639306039):3.4885194504999997,(29:18.9682466868,((32:13.949814633,31:13.949814631999999):3.3089104544000003,30:17.258725087000002):1.7095216003):12.159578802999999):2.797653527,((((53:5.537353174,(((57:2.1667637645999998,58:2.1667637646999998):1.660118069,(63:3.197204394,((61:1.276874442,62:1.2768744421):1.356974475,60:2.633848917):0.5633554771):0.6296774394):0.2414810308,59:4.068362864):1.46899031):1.294209719,(54:4.380113217,(55:3.300419142,56:3.3004191425):1.0796940751):2.451449676):2.273796201,(64:6.689596055,65:6.689596055):2.41576304):10.151303341,(((((44:5.103309076,45:5.1033090759):1.850912128,43:6.954221204):1.02677543,((((37:4.055612741,36:4.0556127411):0.5107491586,38:4.566361899):2.283154242,(41:3.2642306,(40:2.279375991,39:2.279375991):0.9848546092):3.5852855419000003):0.2538892126,42:7.103405355):0.8775912788000001):0.7360313481,((34:3.300011362,33:3.300011362):2.15631588,35:5.4563272417):3.26070074):8.9663975951,(52:12.4810615119,(((51:6.148213921,(48:2.890531171,49:2.8905311706):3.2576827492700002):0.3824743789,50:6.5306882997999995):2.504831779,(47:6.227040457,46:6.227040457):2.808479622):3.445541436):5.202364062):1.5732368634):14.668816582000002):5.603065486):3.458251888,(78:38.428661324000004,((79:5.099161705,80:5.099161705):17.71905231,((((83:13.39804163,(82:11.9126824776,81:11.912682477):1.4853591489):3.198140437,85:16.596182063):0.2038681521,84:16.800050220000003):1.118627301,(87:8.62952027,86:8.6295202696):9.289157246):4.899536497):15.610447306200001):4.558135084):3.435573324,(((((((89:25.283017574,(91:7.503468789,90:7.503468788):17.779548788):6.211597952,88:31.4946155305):4.674692256,92:36.169307783):3.962383417,(((((((99:13.67886363,((93:9.665953788,(98:7.317954773,(97:5.982414214,(95:3.533356441,96:3.533356441):2.449057773):1.3355405579):2.3479990162999997):0.7091603116,94:10.3751141):3.3037495317000003):0.6140760165,100:14.29293965):2.9029776737999997,101:17.1959173273):1.052133215,102:18.248050537800005):8.293971702,((((((((((106:8.355260832,107:8.3552608318):3.750242798,(((108:7.4717935909,(109:0.2523312909,110:0.2523312909):7.2194622998):0.8481090402,(112:3.7085112047999997,111:3.708511204):4.611391426):1.069670828,113:9.389573458600001):2.7159301711):0.4949975711,(115:2.21119401,114:2.21119401):10.38930719):1.091406774,(117:6.282996936,116:6.282996935360001):7.40891104):1.639324019,118:15.331231994):0.3336215376,((119:0.9193713918,120:0.9193713918199999):6.825359614,121:7.744731005999999):7.920122526499999):1.0893675282,((104:2.877576185,105:2.8775761849999997):10.15635139,103:13.0339275752):3.720293487):0.3895192087,122:17.1437402709):1.671317466,((125:8.758849688999998,124:8.75884968798):5.4165303594,123:14.175380048730002):4.6396776869):0.51958114726,126:19.334638882):7.2073833575999995):1.460486628,127:28.002508871099998):0.7631321329,(128:8.6233757554,129:8.6233757552):20.142265244340003):11.36605021):3.139303099,130:43.270994305):1.987391475,(132:36.426206877,131:36.426206879999995):8.832178901999999):0.8546897466,(((((137:6.958905883,138:6.9589058837):11.937947813400001,((141:6.514201689,(140:4.235241893,139:4.2352418929999995):2.2789597957):4.936098363,142:11.450300051820001):7.446553648299999):1.651161767,((135:3.975674768,(133:1.683328135,134:1.683328135):2.292346633):1.115953329,136:5.091628097):15.45638737248):12.56708853,((((143:8.412428795,144:8.412428796):20.63550077,((((((((153:1.189950092,154:1.18995009188):4.754331759,(151:1.1197115492,152:1.1197115492999998):4.824570301900001):2.918800974,(149:6.07718709,150:6.07718709):2.785895735):0.3756498396,(((156:4.580661496,155:4.580661496):3.34683995,157:7.927501446):0.9394707277,158:8.86697217352):0.371760491):3.1937758791000004,159:12.4325085444):7.302451741,(160:18.648999736900002,(147:16.215509361200002,148:16.2155093626):2.43349037353):1.08596055):0.3952712579,(146:14.1686945487,145:14.16869455):5.961536994):2.725406059,161:22.8556376023):6.1922919620000005):1.463809817,((162:23.5971819621,163:23.5971819643):2.961375083,(((((((166:0.3202007583,165:0.3202007583):6.5202901974,164:6.840490956):1.645034669,(167:2.892899033,168:2.892899033):5.592626591):0.4939462675,(170:2.648001227,169:2.648001227):6.3314706659999995):1.211511686,171:10.19098358):1.987848125,(173:3.481516686,172:3.481516686):8.69731501809):0.9011129619,((179:7.857249293,((177:6.1286759102,(176:4.888781682,(175:4.330170282,174:4.330170282):0.5586114001):1.2398942281999998):0.9583175094,178:7.0869934197):0.770255873):1.721365758,(180:6.311736598,181:6.311736598):3.2668784524):3.5013296150000004):13.4786123841889):3.953182334):2.188287423,((183:16.217458698,182:16.2174587007):6.549398729,((185:5.147574912,184:5.147574912):5.4895457524,186:10.637120665):12.129736763999999):9.933169371):0.4150771935):2.3095857,(187:0.1019738248,188:0.1019738248):35.3227158703):10.6883858272):0.3092941951):4.6718090684);

TREE tree_7780 = ((18:31.373553788000002,(((11:11.19229503,12:11.19229503):11.91499716,((1:16.97659335,2:16.976593346):2.293825683,(((4:11.337210659,((6:1.980882345,5:1.9808823454):7.261833046,((7:5.337114821,8:5.3371148213):0.7884822612,9:6.1255970834):3.1171183092):2.0944952677):4.048455832,10:15.385666495):0.77230678,3:16.1579732705):3.112445759):3.8368731619):2.625740803,(((16:8.505399749399999,(15:6.003049183,14:6.003049183):2.5023505664):0.5266453759,13:9.0320451263):0.743592705,17:9.775637830600001):15.957395164200001):5.640520796900001):20.3537965274,(((((((((((24:7.4099464357,23:7.409946435599999):6.853932393,(21:6.453020749,22:6.45302075):7.810858080500001):0.2227560225,(19:3.451608263,20:3.4516082632):11.03502659):1.816277673,(26:9.446986504,25:9.446986503):6.8559260224999985):3.256443341,27:19.559355866300002):3.9711049791999997,28:23.530460845900002):6.302872594,(((31:13.4388975624,32:13.438897563):2.0635838921,30:15.502481454):3.5389273360000004,29:19.041408790189998):10.791924649):5.511853991,(((64:11.76992327,65:11.769923262599999):6.103048894,(((((63:6.82372645,(61:4.284032163,62:4.284032163):2.539694287):0.101324018,60:6.925050468):2.384343247,((58:5.820367372,57:5.8203673717):2.479962881,59:8.300330253):1.009063462):1.780253052,53:11.08964677):1.583147248,(54:11.84315304,(55:7.80828444,56:7.8082844395):4.0348685988):0.8296409756):5.200178146):9.39071611,((((33:5.148028965,34:5.148028965):3.008480039,35:8.1565090042):4.060296813,(((((40:5.673762912,39:5.673762912):1.588937934,41:7.2627008455999995):2.202341057,((37:6.644470489000001,36:6.6444704891):0.2028511789,38:6.847321668):2.617720235):0.560281425,42:10.025323327999999):1.425093367,(43:10.30694776,(44:7.469518141,45:7.469518141):2.837429617):1.1434689373):0.7663891233):12.925162914,(52:17.83302641253,((47:9.281784756,46:9.281784756179999):3.932606158,((48:5.619727172,49:5.619727172):3.825373614,(51:8.805247978,50:8.805247979099999):0.6398528067):3.769290129):4.6186354989):7.308942319):2.1217195385):8.081499159):3.682724756,(66:25.52922072,((((72:11.95180234,71:11.95180233986):1.527657154,(70:10.02261696,((68:3.096458325,67:3.0964583251):5.416660903,69:8.513119228039999):1.509497728):3.4568425383):1.282375223,((76:9.277874131,75:9.2778741314):3.40652724,(74:11.47210801,77:11.472108013):1.212293358):2.077433346):0.9836748662,73:15.745509583):9.7837111369072):13.4986914686):3.147192517,(((80:2.899552257,79:2.899552257):17.20450619,(((((81:8.6903369699,82:8.69033697):1.221711251,83:9.9120482206):3.81382177,85:13.72586999):0.4424701923,84:14.168340183):1.146135064,(86:9.135597428,87:9.135597428):6.1788778187):4.789583204):16.221752323,78:36.325810775499995):5.8492939372):4.54171814,(((((((89:24.71935249,88:24.7193524948):1.955077838,(91:8.042114169,90:8.042114168):18.6323161591):3.622452335,92:30.296882664800002):7.129532264,((((((((93:9.446752633,((97:5.618801163,(95:2.983012217,96:2.9830122169999997):2.6357889464):0.5703147218,98:6.189115885):3.2576367476000003):0.1106249542,94:9.557377587300001):5.276578426,(100:13.25299321,99:13.25299321):1.580962807):0.8846819732,101:15.718637985):1.6201146695,102:17.3387526571):4.058979304499999,(126:14.703224525500001,(((((((((106:7.793124021,107:7.79312402095):3.033363221,((((109:0.09518519303,110:0.09518519303):5.3009126279,108:5.39609782087):0.8375394398,(111:4.052908379,112:4.0529083785):2.180728882):2.141701897,113:8.3753391583):2.4511480847000002):1.167423396,(115:2.041883,114:2.041883):9.9520276378):0.3780542655,(117:5.460386897,116:5.460386897499999):6.911578005599999):0.4978309444,(121:6.448980518900001,(120:1.431429718,119:1.4314297182):5.0175508011):6.420815329800001):0.02812536425,118:12.897921213):0.5634682949500001,((105:2.646134045,104:2.646134045):7.342543875,103:9.9886779195):3.4727115884):0.2851394625,122:13.74652897062):0.691964522,((125:6.6645017248,124:6.6645017247):4.5577878510800005,123:11.222289575330002):3.2162039163):0.26473103347):6.694507434):3.002471373,127:24.400203335):0.009270142954,(128:6.5505984728,129:6.5505984728):17.858874996810002):13.01694146):5.254040856,130:42.6804557798):2.303984551,(132:36.145215483,131:36.145215480000005):8.839224843):0.803196164,((((((143:8.476543353,144:8.476543353):16.79188015,((((146:13.108475156499999,145:13.108475154999999):3.081354258,(160:15.922714615,(147:14.5692421091,148:14.569242109900001):1.3534725064):0.2671147991):0.4282653698,((((((155:3.899495161,156:3.899495161):2.319466446,157:6.218961606):1.325296305,158:7.54425791245):0.3613489735,(149:6.58247639,150:6.5824763895):1.323130496):0.7469243495,((154:0.7432131228,153:0.7432131229000001):4.301034868,(152:0.9679707917,151:0.9679707918):4.076277199):3.60828324414):2.075207651,159:10.7277388861):5.890355898):3.212160875,161:19.830255659000002):5.43816784):0.7557174705,((162:14.627561175,163:14.6275611778):5.626532632,((((((164:4.213912494,(166:0.5943907611,165:0.5943907611):3.6195217324):1.982635252,(167:1.664232513,168:1.664232513):4.532315232):1.116408033,(169:2.593526315,170:2.5935263152):4.719429462):0.9896484996,171:8.302604278):3.6778493,(172:4.065358722,173:4.065358722):7.9150948561000005):0.6529546973,((179:6.476179335,((177:4.719644657,(176:4.386987418,(175:3.420254412,174:3.420254412):0.9667330056):0.3326572392):0.5019068754,178:5.2215515329999995):1.254627802):1.48329657,(180:5.135573999,181:5.135573999):2.823901906):4.6739323705):7.6206855381):5.770047156):4.326193481,(((135:3.560117495,(134:2.221582152,133:2.221582151178):1.338535343):0.5452385148,136:4.10535601):20.277133368,((142:10.773830346490001,(141:5.930829397,(140:3.925675868,139:3.9256758691):2.0051535274):4.8430009499999995):9.587581738999999,(137:5.241269837,138:5.2412698376):15.120142250099999):4.0210772916):5.967845071):0.8886026419,((183:14.3592514673,182:14.359251468000002):3.77393498089,((184:2.890887908,185:2.8908879075):2.8844372923000003,186:5.7753252):12.3578612489):13.105750641):4.64684016,(187:2.465855864,188:2.465855864):33.419921390460004):9.901859246):0.9291863484):5.010527467999999);

TREE tree_0670 = ((18:28.402281317600004,(((11:10.12600533,12:10.12600533):11.469654001,((1:17.35201606,2:17.35201606):1.849041694,(((4:12.109089142,((6:3.2093767232,5:3.2093767229):5.556291123,((7:5.215426321,8:5.215426321400001):1.386754709,9:6.602181030400001):2.163486815):3.343421296253):3.108453223,10:15.217542363):0.9741488668,3:16.191691231):3.0093665214):2.3946015798999998):3.73484849,((((14:4.763046646,15:4.763046646):2.822071022,13:7.5851176672000005):0.8323580836,17:8.4174757514):0.03947659992,16:8.456952351):16.873555467):3.0717734976999997):18.671079632,((((((((((((36:6.5123387275999995,37:6.5123387269999995):0.8209422484,38:7.333280976):4.070437939,((39:5.361812806,40:5.361812806):2.438598759,41:7.800411565399999):3.60330735):0.8548197971,42:12.258538712):1.212925591,(43:12.1993997353,(45:8.5096606787,44:8.509660679):3.6897390569999997):1.2720645670700002):1.549685501,((33:5.751350798,34:5.751350799):3.347014189,35:9.098364987):5.9227848175):14.837806975,(((46:11.132433472999999,47:11.132433472999999):2.704876478,((51:10.71180177,50:10.7118017717):0.4014055019,(48:6.380657835,49:6.380657835):4.732549438):2.724102678):5.490298323999999,52:19.3276082723):10.5313485):1.4868195847635999,(29:18.951399262400002,((31:15.4875785946,32:15.487578596):1.567322752,30:17.054901344):1.89649791562):12.394377098):0.7664855846,(((((((21:6.29192594,22:6.29192594):7.988930107000001,(24:8.8359779418,23:8.8359779418):5.444878105):2.060465023,(19:4.835198858,20:4.8351988576):11.506122212):2.725666922,(25:10.1038060131,26:10.103806013):8.963181979200002):2.789162617,27:21.85615061):2.437164538,28:24.293315147):5.0126808622,(((54:11.64168954,(55:5.799449686,56:5.7994496862):5.842239854000001):0.2938741753,((((((61:2.816484403,62:2.8164844023):2.4050467,63:5.221531102):0.9611655636,60:6.182696666):1.880929707,(57:3.39819176,58:3.3981917599):4.665434613):1.155748812,59:9.219375185):1.221173882,53:10.4405490719):1.495014648):3.042177879,(64:11.16392796,65:11.1639279655):3.813813631):14.328254415899998):2.806265936):3.4121283929,(66:28.47823166,(((71:9.586308354,72:9.5863083536):0.8361328964,((((68:2.633681046,67:2.6336810453000004):4.676416236,69:7.310097280999999):1.082539819,70:8.3926371):1.8463110552,((75:5.177080571,(76:2.02972961,77:2.02972961):3.147350962):3.016987479,74:8.19406805):2.0448801045):0.1834930954):0.8616503378,73:11.2840915874):17.194140072000003):7.046158676999999):4.692168364,(78:35.2374927204,((((((81:8.826638758,82:8.8266387592):0.8942381998,83:9.720876958089999):2.99054035,85:12.711417308000001):0.4215610974,84:13.132978405560001):1.108953614,(87:7.328639655,86:7.328639656):6.913292364):2.323902321,(80:2.381858764,79:2.381858764327):14.183975577):18.6716583758):4.9790659836):1.13239014,(((((((89:21.831016885,(91:8.529605319,90:8.52960532):13.301411563):5.313083831,88:27.144100715199997):3.529699363,92:30.673800073200002):5.397337453,((((126:19.538668961,(((((((((106:9.948190436400001,107:9.9481904375):3.529383695,((((109:0.202849952,110:0.202849952):7.0973628983,108:7.3002128505999995):1.825349681,(111:4.152817297,112:4.1528172972):4.972745234):0.9099097544,113:10.0354722851):3.4421018462000004):0.5705267077,(114:2.575245822,115:2.575245822):11.4728550186):1.373622251,(117:6.3043409928,116:6.304340992):9.117382098):0.831519354,((120:1.223344644,119:1.223344644):6.4738161088,121:7.6971607530999995):8.5560816911):0.0003996830141,118:16.253642127):0.8470959296,((105:4.278938409,104:4.278938409):10.2254443,103:14.504382708000001):2.596355344):0.1535016993,122:17.254239758500002):1.479339431,(123:15.5923144231,(124:10.45959551438,125:10.4595955134):5.13271891029):3.1412647634200006):0.8050897734):4.324881349,(102:16.279610586700002,(((100:12.04734654,99:12.04734654):0.8974735292,(((98:5.5952249408,((95:3.142833942,96:3.142833941):0.9937000179,97:4.136533959):1.4586909813):3.02973509564,94:8.624960036000001):0.1359303362,93:8.7608903716):4.183929697):2.494976069,101:15.43979613746):0.8398144497):7.5839397232):0.7643290422,127:24.627879347):1.01268496,(129:6.868147737399999,128:6.8681477373):18.77241657229):10.43057322):1.562843275,130:37.6339808108):2.578220839,(132:21.227896234,131:21.227896228):18.984305422):0.1172367495,((((((137:3.943537175,138:3.9435371743000003):11.566039164000001,((141:5.382020024,(140:3.58801236,139:3.5880123601):1.7940076642):5.4310841089999995,142:10.813104133):4.696472206):2.8425509274999996,((135:2.488580692,(134:1.10412134,133:1.10412134):1.384459352):0.1753244982,136:2.6639051898):15.688222076299999):8.860846174,(((143:8.626135942,144:8.626135942):16.27303746,(((((148:15.533212946099999,147:15.533212946799999):1.1912579216,160:16.724470868):1.279148213,((((150:6.475359456,149:6.4753594564):1.73727618,((152:2.2835418784,151:2.2835418781000003):3.492775217,(153:0.3955537496,154:0.3955537496):5.380763345399999):2.436318542):0.3445065147,(((155:3.699460758,156:3.699460758):3.156463335,157:6.8559240930000005):1.339360307,158:8.19528440029):0.3618577516):2.571417673,159:11.128559824):6.875059257):0.8731545059,(146:13.789520219,145:13.789520219):5.087253367):1.482978259,161:20.3597518465):4.539421556):1.030802299,((162:15.6917940291,163:15.6917940262):6.77514157,(((171:9.959520342,((((166:0.7744882938,165:0.7744882938):4.867278156699999,164:5.6417664499999995):2.085846528,(168:1.789138011,167:1.789138011):5.938474966999999):0.6306000227,(170:2.760334614,169:2.7603346146):5.597878387):1.601307341):2.311759988,(172:2.770794935,173:2.770794935):9.500485395):1.603453495,((((((176:5.054499006,175:5.054499006):1.281263259,174:6.335762266):0.2146898428,177:6.5504521086):0.3968672655,178:6.947319374):1.123534106,179:8.07085348):0.8135511631,(181:5.06831527,180:5.06831527):3.8160893739999997):4.990329183):8.5922017675271):3.463040105):1.282997742):0.6164212356,((182:12.0511634406,183:12.051163441):9.587831782,((185:1.8025742047,184:1.8025742042100001):3.243195178,186:5.04576938259):16.5932258411):6.190399457):3.155970318,(188:0.0885781376,187:0.0885781376):30.896786853714598):9.3440734):1.019510449):5.724412108);

TREE tree_2217 = ((18:23.142325495799998,(((11:7.05457207,12:7.05457207):8.182492661,((1:13.03852116,2:13.038521156):0.9585243227,((((((8:3.7126825748299996,7:3.7126825748999996):1.300404877,9:5.0130874509000005):1.5086432523000002,(6:1.595056586,5:1.5950565863000001):4.926674117):2.609030054,4:9.130760758000001):0.9788508983,10:10.109611657):1.1305313606,3:11.240143016):2.7569024615):1.2400192522):3.298816104,(17:6.954230177,(16:6.754300769,(13:6.3771373017,(15:3.706513151,14:3.706513151):2.67062415):0.3771634676):0.19992940782):11.581650657):4.6064446666):24.950090498,((((66:25.79586162,(((72:12.159489277999999,71:12.159489277):3.429430776,(((69:9.677536690999998,(68:5.532569142,67:5.5325691417):4.1449675486):1.43047717,70:11.10801386):3.184450138,((75:3.566027413,77:3.5660274131999996):6.558223816,(76:1.836084336,74:1.836084336):8.2881668932):4.168212767590001):1.296456056):0.7484088737,73:16.33732893):9.458532692616199):13.838230359,(((((((55:4.305405562,56:4.305405561500001):4.77901874,54:9.084424301):2.194162716,((((57:3.564206928,58:3.5642069287):2.50120653,59:6.065413459):0.8580381784,((61:5.516176428,62:5.516176428):1.118757581,(63:6.485210625,60:6.485210625):0.1497233832):0.2885176284):3.871947278,53:10.79539892):0.4831881023):1.718054952,(64:8.719717208,65:8.719717207899999):4.276924761):13.995478542,((((((23:8.307208692,24:8.30720869234):6.824508509,(21:5.239810733,22:5.2398107330000006):9.891906468050001):1.21675159,(19:4.748137975,20:4.7481379742000005):11.60033082):2.411382581,(25:10.173131714,26:10.1731317146):8.5867196578):5.644795171,27:24.404646539100003):1.838724321,28:26.243370864000003):0.7487496465051):8.68665877,(((31:17.5691786772,32:17.569178679):2.3032728131,30:19.872451492):3.205758768,29:23.078210257900004):12.600569022):1.172137029,((((33:5.49450656,34:5.49450656):5.2920977593,35:10.786604319):4.726951886,(((((40:7.199589931,39:7.199589931):2.880878938,41:10.080468869):2.665502901,((36:7.0839379092999994,37:7.083937909):2.193463708,38:9.277401616999999):3.468570153):0.8319375913,42:13.577909361):1.512180865,((44:10.162666814,45:10.1626668141):1.796356978,43:11.959023792):3.131066434):0.4234659784):15.592486818000001,(((46:14.352404977,47:14.352404979000001):1.931527307,((49:8.419688518,48:8.419688518100001):4.514866109,(51:12.080271252,50:12.0802712506):0.8542833755):3.34937766):5.625114308000001,52:21.909046595099998):9.196996429):5.744873286000001):2.7831756674999997):4.129764323,(78:38.4635280782,((79:4.811021741,80:4.811021741):19.18852178,(((83:16.55873691,(82:12.890198362,81:12.890198363):3.6685385449):1.050786764,(85:17.212436247,84:17.212436247):0.3970874247):0.8025684155,(86:7.275357406,87:7.275357405299999):11.136734682):5.587451428):14.463984556):5.3003282266000005):1.098659575,(((((((89:26.63064204,(90:5.88248446,91:5.88248446):20.7481575864):1.726019231,88:28.3566612739):4.2457565109,92:32.602417779):4.977112086,(((((101:19.566999240799998,((99:16.73678217,((94:11.578204463999999,93:11.578204463999999):0.1358558852,(((95:3.376791623,96:3.376791623):3.045135941,97:6.421927564000001):1.363982438,98:7.7859100016):3.928150347):5.022721823):0.3725792954,100:17.10936147):2.4576377734):0.4633621222,102:20.030361363499996):8.425044916200001,((((((105:3.755621355,104:3.755621355):11.18301069,103:14.938632044):3.654428953,((((((106:9.755820673,107:9.755820671799999):4.9076374638999996,(((108:7.3304949672,(109:0.3613389348,110:0.3613389348):6.969156031700001):1.353905113,(111:4.783953051,112:4.78395305):3.900447029):2.85552959,113:11.539929669663):3.1235284668):0.871955381,(115:3.682411329,114:3.682411329):11.8530021888):0.5070547284,(116:7.0157138014000004,117:7.015713802):9.02675444431):1.359464347,118:17.401932592):0.8994495461,(121:8.7184342497,(120:2.377962404,119:2.377962404):6.3404718464):9.582947888900001):0.29167886204):0.8390560029,122:19.4321169994):1.014030788,((125:9.607304318799999,124:9.607304319):6.6520742035,123:16.2593785216):4.1867692685):0.1101343782,126:20.556282170000003):7.899124110300001):1.289296257,127:29.744702535000002):0.6351722001,(129:8.1686807838,128:8.1686807835):22.2111939557):7.199655136):3.693266491,130:41.27279637):1.363352232,(131:22.938106036,132:22.938106038999997):19.698042561):0.2555770367,((((((135:4.01132678,(133:2.825665715,134:2.8256657148):1.185661065):0.6534690696,136:4.66479585):15.901917715,((137:6.01838301839,138:6.0183830182):10.637470586000001,((141:5.8179525093999995,(140:4.616275375,139:4.616275375399999):1.2016771347):5.272004853,142:11.089957363):5.565896241):3.910859964):9.460290442,(((143:7.734558335,144:7.734558335):18.52510726,(((((146:11.55466991236,145:11.554669916):3.477381444,160:15.032051356):2.0667022858,(148:13.962776419999999,147:13.96277642051):3.13597722102):1.375793505,((((((154:0.6454691981,153:0.6454691981):3.938768323,(151:1.332931589,152:1.3329315891999998):3.2513059321):2.718048214,(149:5.53268804,150:5.53268804):1.769597695):1.041749091,((155:4.8844560363,156:4.884456036):1.64859693,157:6.5330529666):1.810981861):0.4263645716,158:8.770399399):3.52581563021,159:12.2962150301):6.178332117):2.861650283,161:21.3361974295):4.923468163):0.4545454377,((162:19.7116288821,163:19.7116288845):2.561448468,(((171:3.126977884,(((164:2.0843882163,(166:0.1722283029,165:0.1722283029):1.912159913):0.6179766418,(168:0.6888003539,167:0.6888003539):2.0135645045):0.1467955588,(169:0.8305164733,170:0.8305164733):2.018643944):0.2778174676):1.361194305,(172:1.036498454,173:1.036498454):3.451673735):0.3375831507,((((((175:2.001102734,176:2.0011027347):0.1593647427,174:2.160467477):0.1125148699,177:2.2729823471):0.2566017561,178:2.5295841030000004):0.5660256331,179:3.095609736):0.3034736369,(181:1.3275646,180:1.3275646):2.0715187732):1.4266719671):17.447322004483745):4.441133676):3.312792983):0.9191727315,((183:17.589754337000002,182:17.5897543416):3.319410649,((184:4.499436862,185:4.4994368631):4.34370286,186:8.843139722):12.0660252673):10.03701175):3.956373674,(188:0.1051579352,187:0.1051579352):34.7973924835435):7.98917522):1.97079024):3.2299001253);

TREE tree_3431 = ((((((1:18.82092941,2:18.820929414):1.36546304,(((4:14.191182979,(((7:5.949712363470001,8:5.9497123645):1.801817636,9:7.7515299994):1.329567766,(5:2.313085459,6:2.313085459):6.7680123074):5.1100852126000005):1.803018505,10:15.994201486000001):0.9504597563,3:16.944661240400002):3.241731214):1.4186210341999999,(11:9.346746532,12:9.346746532):12.258266956):4.635859009,(((13:9.256304125,(15:4.87631394,14:4.876313940699999):4.3799901841):0.8473452716,16:10.1036494):0.2727456093,17:10.3763950058):15.864477486400002):5.066664163,18:31.307536662549502):19.8746152348,((((66:32.51112784,((((((77:4.225159405,75:4.225159403999999):3.950501976,76:8.1756613806):2.150746355,74:10.32640774):1.207918786,(71:9.845789420000001,72:9.845789421):1.688537101):1.111330724,((69:8.9838353777,(68:6.17509655,67:6.175096549):2.8087388275):1.494170192,70:10.478005569):2.1676516774000003):1.155120699,73:13.800777949):18.7103498952):4.285875691,((((28:26.377186820000002,(((((21:5.374313754,22:5.374313753):8.740995089,(23:8.736684555,24:8.736684554690001):5.378624289):0.3522997386,(19:5.672852316,20:5.6728523157):8.794756267):3.463598046,(26:11.9167952625,25:11.916795262):6.0144113664):4.95163629,27:22.882842920199998):3.4943439016):5.33558849,(((32:14.4188307156,31:14.418830716199999):1.8389903437500001,30:16.257821059999998):3.710750377,29:19.968571438300003):11.744203872):0.3474131226,((((46:11.3538608426,47:11.353860842):3.115898928,((51:9.720833342,50:9.720833341999999):0.5569880489,(48:6.253704158,49:6.253704158):4.024117233):4.191938378400001):4.951591129,52:19.421350898300002):9.776563536000001,((((45:11.335168248000002,44:11.335168248):0.7189310651,43:12.05409931):1.83147521,(((41:7.602473915,(39:3.831847434,40:3.831847434):3.770626481):3.232722709,((36:5.8445907734,37:5.844590773):1.561455929,38:7.4060467029999995):3.4291499216809997):0.7988905922,42:11.634087216400001):2.2514873073):0.5775244297,((33:5.232551771,34:5.232551771):3.492162591,35:8.724714362):5.738384592):14.7348154837):2.8622739958843):0.8014907726,((64:12.7098195,65:12.709819495000001):1.916050662,(53:12.58433925,((((57:8.356968157,58:8.356968157):1.157390628,59:9.514358784999999):0.2248828037,(((61:4.847846576,62:4.847846576):3.702669274,63:8.55051585):0.4811962246,60:9.031712074):0.7075295142):1.957029161,(54:10.67519971,(55:7.788517134,56:7.788517133999999):2.8866825780000003):1.021071037):0.8880684972):2.0415309132):18.235809047):3.9353243310000003):3.66558002,(78:32.75045301204,((((((81:12.125294202,82:12.125294201300001):0.3990513404,83:12.524345542299999):2.31313262,84:14.837478161):0.1873333818,85:15.024811544):0.3371121494,(86:6.762927182,87:6.762927182):8.598996510900001):3.844083136,(79:3.125332549,80:3.1253325483000003):16.08067428):13.544446182):7.712130548):4.542107393,(((((((((101:20.405285035200002,(((94:11.361188342,93:11.3611883427):0.2758565537,(98:9.0555276444,((95:4.483141036,96:4.483141036):1.420308162,97:5.903449198):3.152078446):2.5815172517000002):2.837579045,(100:12.23364205,99:12.23364205):2.24098189):5.930661097):0.1400648865,102:20.5453499243):6.928145037,((((((((((107:10.53149054915,106:10.53149055):4.68997799,(((111:5.598177756,112:5.598177757):3.418550183,(108:7.882965173,(109:0.252856035,110:0.252856035):7.6301091374):1.1337627659):1.734501337,113:10.7512292753):4.4702392644):0.5795886198,(115:1.650376576,114:1.650376576):14.150680584):0.6783603326,(117:6.8920212440999995,116:6.8920212439):9.587396249):1.689835554,118:18.1692530487):0.7565238695,((119:1.567941817,120:1.567941817):6.671881645,121:8.239823462):10.6859534545):0.29299048949999995,122:19.218767408399998):0.4446590885,((105:4.624279934,104:4.624279934):11.14059714,103:15.764877076):3.898549416):1.04534447,(123:16.33726021454,(124:8.945967674,125:8.9459676739):7.39129254255):4.3715107479):0.004297249761,126:20.71306821293):6.7604267466):4.628701945,127:32.102196908):0.2964041384,(128:8.8238542451,129:8.8238542454):23.574746805300002):7.918513395,((((91:7.1330504586000005,90:7.133050459):21.6467174963,89:28.77976795):2.874078113,88:31.6538460639):2.013089735,92:33.666935798):6.65017864):2.912919217,130:43.230033661):0.9197225879,(131:29.602279204,132:29.602279202):14.547477046000001):0.1557630244,((((((143:7.634512787,144:7.634512786999999):17.89482504,((((146:10.630974819,145:10.630974819):5.692905163,(160:15.577645148,(148:14.045453674200001,147:14.0454536743):1.532191474):0.7462348339):1.087186961,(159:11.225882921,((((156:4.316161147,155:4.316161147):2.958015075,157:7.274176220999999):0.9252274479,158:8.1994036691):0.1543882598,(((154:0.4181094221,153:0.4181094221):4.387538214404,(151:1.130096894,152:1.130096894):3.6755507431):2.349615441,(149:5.604822784,150:5.604822784):1.550440294):1.198528851):2.8720909925000004):6.185184022):3.314749789,161:20.725816731000002):4.803521098):1.478721391,((162:15.846245257000001,163:15.846245256000001):7.93055921,((((((164:4.111750078,(165:0.8035473821,166:0.8035473821):3.3082026950000003):0.5030554418,(167:1.222152513,168:1.222152513):3.392653007):0.7817431217,(169:0.9975305369,170:0.997530537):4.399018105):0.7509465536,171:6.1474951943):1.585844547,(173:2.561974313,172:2.561974313):5.171365429):0.1971460532,((179:3.931246853,((((176:2.618230367,174:2.618230367):0.4327034839,175:3.0509338513):0.233108203,177:3.284042054):0.04581595351,178:3.3298580077):0.6013888457):0.5139439537,(181:1.376140996,180:1.376140996):3.0690498112):3.4852949879999997):15.846318673160642):3.231254754):2.039406596,(((137:3.573317669,138:3.5733176696999998):13.338073706,((141:9.731192939,(140:5.6284227510000004,139:5.6284227506):4.102770188099999):2.35509038,142:12.086283318):4.825108057):4.2874425425,(136:3.773384373,((133:1.540762831,134:1.540762831):0.4611868214,135:2.001949652):1.7714347207):17.4254495437):7.848631903):2.489743853,((182:15.372937825000001,183:15.372937826000001):7.613327564,(186:5.606754391,(184:2.767760154,185:2.7677601550000004):2.8389942370999997):17.379510999500003):8.550944281):5.989138761,(188:1.311463233,187:1.311463233):36.214885203248):6.77917084):0.6991716785):6.177460946);

TREE tree_4609 = ((18:27.1850597181,(((11:6.809655602,12:6.809655602):12.213095661,((1:15.70370829,2:15.703708292999998):0.8623552388,(3:13.873138430000001,((4:10.89543851,((5:2.5816997492000002,6:2.5816997494000002):5.7789668800000005,((7:5.7240299789,8:5.724029979200999):0.6162450649,9:6.3402750437):2.0203915857):2.5347718814999998):2.145582403,10:13.041020913):0.8321175163000001):2.6929251022):2.456687732):3.630512727,((16:9.021628423,((15:4.093373156,14:4.0933731568):3.174770732,13:7.268143889000001):1.7534845342):0.5108772921,17:9.5325057158):13.120758276):4.53179573):24.320669431300004,((((66:25.23442506,((((((67:5.259970753699999,68:5.259970754):4.257950864,69:9.517921619100001):0.9943188228,70:10.512240441):1.410887955,(((74:1.752945549,75:1.752945549):1.688115037,76:3.441060587):5.986082517,77:9.427143104):2.4959852928000004):0.2309619634,(72:9.272406585,71:9.272406585999999):2.881683774):1.593560768,73:13.747651128000001):11.48677393228):13.919702658999999,(((((30:14.871511792,(31:13.008652143099999,32:13.008652142999999):1.8628596477000001):3.4976603500000003,29:18.3691721412):12.083135359,((((((20:4.6423557272,19:4.6423557267):9.654960828,(24:10.3286839856,23:10.328683987):3.968632568):0.03217850228,(21:7.135158396,22:7.135158396):7.1943366610999995):3.51436367,(26:11.169909532,25:11.1699095329):6.6739491951000005):7.38248036,27:25.226339088):2.14355632,28:27.369895407):3.0824120933):3.317721575,((((47:13.029858253,46:13.029858253):2.224762313,((51:9.544376781,(49:6.551659224,48:6.551659224):2.992717557):0.5387771024,50:10.083153884200001):5.171466682):5.5511718112,52:20.80579238173):11.506802034,((35:8.917256024,(33:5.242916468,34:5.242916468):3.6743395564):5.8425609,(((((37:7.342044853999999,36:7.342044854):0.8119739018,38:8.15401875609):3.729760832,((40:6.57935383,39:6.5793538307):2.901369573,41:9.480723402599999):2.403056184):0.4587427218,42:12.342522309):1.667245329,(43:12.98169827,(44:9.041019197999999,45:9.04101919801):3.940679071):1.028069369):0.7500492863):17.552777487):1.4574346682908002):3.093552916,((64:14.00179325,65:14.0017932487):3.577539226,((((57:6.355779037,58:6.3557790369):4.389029038,((((61:5.434759911,62:5.434759911):2.206819502,63:7.641579412):2.053228467,60:9.694807879):1.002446025,59:10.697253904):0.04755417054):3.190729629,53:13.935537707):1.200255088,(54:15.00258083,(55:7.851443187,56:7.851443187):7.1511376470000005):0.133211958):2.443539683):19.284249513):2.2905457251):2.66892144,(78:35.38711076788,((((((82:10.522459348,81:10.5224593483):0.4576015727,83:10.9800609179):2.493170592,84:13.473231513):1.226766493,85:14.699998006000001):0.6916104845,(87:7.771635412,86:7.771635412):7.6199730788):1.649874676,(79:2.453352225,80:2.453352225):14.588130942100001):18.3456275931):6.435938395000001):2.198760198,((((((((90:8.131665722,91:8.131665721900001):22.075337644,89:30.207003364000002):3.305960528,88:33.5129638928):3.468269229,92:36.981233123):2.236873639,(((((101:16.90168099,((99:12.18873681,100:12.18873681):1.479240772,((94:9.437393267400001,(((95:3.295661772,96:3.295661772):2.330296765,97:5.625958537349):1.350786802,98:6.976745339200001):2.4606479282100002):0.1387040616,93:9.57609733028):4.09188025):3.2337034065):0.5398246633,102:17.4415056484):8.54908753,(126:18.551655335,(((((105:2.945468467,104:2.945468467):9.283242893,103:12.228711362):3.7404406249999997,((((((106:9.102350936,107:9.1023509357):3.65139115,(((108:5.4608456083,(109:0.2282204759,110:0.2282204759):5.2326251316):1.489134429,(111:4.846458683,112:4.846458683):2.103521354):2.097409913,113:9.04738994983):3.7063521352):0.2812422816,(115:2.187853039,114:2.187853039):10.847131328):0.6170300049,(117:5.8474202470000005,116:5.8474202464):7.8045941254999995):1.329088096,118:14.981102468):0.01041633154,((120:1.773061908,119:1.773061908):7.20503403,121:8.978095939300001):6.0134228601):0.9776331869999999):0.763801215,122:16.7329531982):1.376489499,(123:14.30597536742,(125:8.968611078,124:8.968611077):5.33736429003):3.8034673325000004):0.44221263426):7.438937845499999):1.80561627,127:27.79620945):1.125687602,(128:7.8156831101,129:7.8156831095):21.1062139396):10.29620971):2.79264141,130:42.0107481714):0.8028074161,(132:18.493264910999997,131:18.493264905):24.32029068):0.1636431803,((((((143:7.021388159,144:7.021388160000001):17.7949841,((((((((155:4.445225305,156:4.445225305):1.816677367,157:6.2619026709):1.936150074,158:8.198052745353):0.3791718476,(149:5.690242856,150:5.690242856):2.886981737):0.5290720433,((151:1.6050708245999998,152:1.6050708249999999):4.837923707,(153:1.261146356,154:1.261146356):5.1818481757):2.663302104):1.551449039,159:10.657745675000001):5.316942718,(((146:10.4937505274,145:10.493750527):2.131105129,160:12.624855655999998):2.262919399,(148:13.498088871100002,147:13.49808887248):1.38968618377):1.086913338):3.817786369,161:19.792474762):5.0238975):2.171905869,((162:19.3524659241,163:19.352465927):2.422307054,((((((164:5.762504129,(166:0.1399799821,165:0.1399799821):5.622524147):1.224898236,(168:1.90611114,167:1.9061111401000002):5.081291224):0.7774654439,(169:1.920930579,170:1.9209305789000002):5.843937229):1.775723801,171:9.540591609):1.696675689,(172:4.332949208,173:4.332949208):6.904318091):0.6000859468,(((((176:4.832194075,(174:4.404063273,175:4.404063273):0.4281308022):0.9548962854,177:5.7870903605):0.8964214645,178:6.683511825):0.30366761,179:6.987179435):2.082796655,(181:3.909135375,180:3.909135375):5.1608407154):2.7673771543999997):9.9374197347983):5.213505146):2.353156794,((((133:1.318305113,134:1.318305113):1.796614605,135:3.114919718):0.1458334492,136:3.2607531667000003):16.502508831,((((140:3.3505117759000003,139:3.3505117756000002):0.7256926868,141:4.076204463):4.243969356,142:8.320173819):6.891452019,(137:5.475486861,138:5.475486861399999):9.736138978):4.5516361611):9.57817292):0.728179158,((183:13.9898509,182:13.989850894999998):5.419234041,(186:5.569480592,(184:2.214135938,185:2.214135938):3.3553446543000005):13.8396043443):10.660529143):4.988481124,(187:0.1293556401,188:0.1293556401):34.9287395581011):7.919103568):1.044610586):7.483919791);

TREE tree_9455 = ((18:14.2347362204,(((11:4.996088975,12:4.996088975):4.696137817,((1:7.848156618,2:7.848156618):1.202483961,(((4:5.821702989,(((8:2.9536066807,7:2.95360668):0.4256459648,9:3.3792526455000003):0.9840864626,(6:1.5856769869,5:1.5856769870000003):2.7776621205):1.4583638811):1.2461387062,10:7.067841695):0.2915748832,3:7.359416577399999):1.6912240013):0.6415862130800001):2.243087439,((((15:2.063509121,14:2.063509121):1.9168495023,13:3.9803586237):0.2549974799,16:4.2353561036):0.3939406577,17:4.6292967615):7.3060174694999995):2.2994219892):39.122076901999996,((((66:32.40970807,((((74:8.25763608,76:8.25763608):4.51999669,(77:1.569527286,75:1.569527286):11.208105483):1.116411508,((72:9.954328047,71:9.95432804726):2.543378582,73:12.497706629):1.396337649):1.6725981765,((69:8.785005831000001,(67:6.0393502007999995,68:6.0393502007):2.7456556301):1.800995595,70:10.58600143):4.9806410286):16.8430656170807):6.329972378,(((((((((37:7.927341494,36:7.927341493):0.9168528129,38:8.844194306):3.581111304,(41:8.728033236,(40:7.019716021,39:7.019716021):1.708317215):3.6972723740999998):1.265505839,42:13.690811449):1.064811681,((45:9.157421253799999,44:9.157421253999999):4.32046583,43:13.47788708):1.2777360456):0.9795719959,((34:6.008155529,33:6.008155529):4.004498421,35:10.012653950299999):5.722541175):17.35726299,(52:21.776547787000002,(((51:11.81355616,(49:7.052480297,48:7.052480297):4.761075866):0.343951985,50:12.157508147999998):3.553589454,(46:14.279781337,47:14.279781334900001):1.431316269):6.065450184):11.315910326000001):0.9633932288134,(((31:14.860495191400002,32:14.860495191):3.939873791,30:18.800368978999998):3.714371122,29:22.51474010415):11.541111236999999):1.435212532,((((((19:5.768933763,20:5.7689337625):13.03242471,((24:10.955499097499999,23:10.955499098):7.3128034127,(21:4.417397899,22:4.417397899):13.8509046121):0.533055959):2.028204873,(25:13.7594283205,26:13.759428320200001):7.070135021):4.441092837,27:25.270656182):3.854893554,28:29.125549733):1.9951824863999998,((64:14.02557782,65:14.02557782):7.944423395,(53:16.59802122,((54:13.284492,(55:9.851964377,56:9.851964377):3.432527627):2.093976887,((60:7.004314061,(63:6.710642809,(61:2.814622154,62:2.814622155):3.896020655):0.2936712521):2.206343177,((57:6.288275466,58:6.288275466):2.599474335,59:8.887749801):0.3229074367):6.167811653):1.219552328):5.371979996):9.150731007801):4.370331654):3.248616568):8.427250683,(78:40.375528923,((79:3.324473976,80:3.324473976):15.91182523,(((85:15.3594176206,84:15.359417616999998):1.013476528,((81:14.247183854,82:14.247183854):0.1281937026,83:14.375377554):1.997516592):0.7423355202,(87:11.69470378,86:11.6947037777):5.4205258912):2.121069541):21.139229709):6.791402208):2.009868992,(((((((((101:16.95262662,((99:13.63511704,100:13.63511704):0.7866228436,((93:10.727434713900001,94:10.7274347143):0.04211085621,(((95:4.786573006,96:4.786573006):1.330980134,97:6.1175531405):1.645607478,98:7.7631606177):3.00638495306):3.652194314):2.5308867395):2.128687211,102:19.0813138351):9.2772678252,((((((((((106:10.7806883,107:10.780688301800001):4.690932795,(((111:4.813652651,112:4.8136526505):3.755566371,(108:7.24297121,(109:0.128526528,110:0.128526528):7.1144446819):1.326247812):2.180316892,113:10.749535913599999):4.7220851829):0.6051952987,(115:3.385313866,114:3.385313866):12.691502529000001):0.9804416302,(117:7.309992436,116:7.3099924367):9.74726558962):0.7783281103,118:17.835586135):0.1469010953,(121:10.298486545,(119:2.531468276,120:2.5314682756):7.7670182691):7.684000687000001):0.5230148333,((105:2.856825809,104:2.856825809):11.2808554,103:14.137681204):4.36782086):0.6470628245,122:19.152564887):1.083002857,((125:9.269691268999999,124:9.2696912694):6.5547638768,123:15.8244551432):4.411112601):0.9705112606999999,126:21.206079007):7.1525026525):1.10203742,127:29.460619080599997):3.573904064,(128:9.5516566906,129:9.5516566897):23.482866460300002):9.933999747,((((90:8.386773794,91:8.386773795):17.374044468999998,89:25.760818262):3.32339329,88:29.08421154906):7.197040676,92:36.28125222600001):6.687270664):1.541480368,130:44.510003259):1.146606006,(132:35.883684884000004,131:35.88368489):9.772924384):0.4447314569,(((((((133:3.020161299,134:3.020161299):1.443970446,135:4.4641317461000005):0.6755443094,136:5.1396760553):16.425503939400002,((142:10.728253257,(141:5.035339174,(139:4.034942221,140:4.0349422211):1.0003969538):5.692914082500001):7.089623857,(138:5.828726418,137:5.82872641766):11.989150695):3.7473028832):10.65784627,(((143:7.161581156,144:7.1615811570000005):19.01716066,(((146:12.905138068100001,145:12.905138069):4.502102219,((((((149:5.966573167,150:5.9665731672):1.021204219,((153:0.3169411386,154:0.3169411386):5.214572026,(152:1.6794547110480003,151:1.6794547108):3.852058454):1.456264222):0.8664246023,((155:3.393974133,156:3.3939741330000004):3.5324875687999997,157:6.926461702):0.9277402873):0.1701216127,158:8.02432360098):1.8067329416,159:9.831056544):7.253326324,(160:15.797721347,(148:14.375345719099998,147:14.375345718600002):1.4223756278):1.28666152):0.3228574203):2.729375814,161:20.136616097999998):6.042125714):1.384387974,((162:20.013941737,163:20.013941734):0.8517728234,((((((164:7.854647083,(165:0.8176024403,166:0.8176024403):7.0370446423):1.36798714,(167:4.773507037,168:4.773507037):4.4491271861):1.160520417,(169:3.142200831,170:3.1422008317000003):7.2409538090000005):1.962133022,171:12.34528766):0.7712359174,(173:5.164628342,172:5.164628342):7.951895236):0.6041781485,((179:9.414276231,(((176:4.540323858,(174:2.818103192,175:2.818103192):1.722220666):2.0734758396,177:6.613799698):0.5954524136,178:7.209252111):2.20502412):0.5258449819,(180:5.749765915,181:5.749765915):4.190355297):3.7805805142):7.1450128388):6.697415228):4.659896481):0.5465178983,((182:13.193943895,183:13.193943896):6.655497699,(186:6.921981027,(185:3.204266536,184:3.204266537):3.717714491):12.9274605681414):12.920102575):3.333255642,(188:0.2469659477,187:0.2469659477):35.85583386810001):9.998540913):3.075459395):4.180013001);

TREE tree_2933 = (((((11:9.824614406,12:9.824614406):9.94950601,((1:16.15014714,2:16.15014714):2.051056263,((3:14.277553714,10:14.277553716):1.215397077,(4:12.136193276,((6:2.420449801,5:2.420449801):6.656571835199999,((8:4.5635556079,7:4.563555609000001):1.26417285483,9:5.827728464):3.249293172):3.0591716404000002):3.356757516):2.708252611):1.5729170125):4.113739155,((13:8.3847495574,(16:7.770419129,(14:4.487720119,15:4.4877201190000005):3.282699011):0.6143304277):1.376045516,17:9.7607950738):14.12706449629):3.7198074193,18:27.607666991232996):26.6340037872,(((((((30:20.33451025,(31:18.0987890947,32:18.098789092):2.2357211528):2.848375648,29:23.182885894600002):15.024859635,((((47:15.331084891100001,46:15.331084894499998):2.849918759,((49:7.992836166,48:7.992836166):4.312799401,(51:11.88651813,50:11.8865181351):0.4191174323):5.875368083):4.805247439,52:22.986251089699998):12.244169924,((((45:11.352399896000001,44:11.352399896000001):2.308254394,43:13.66065429):1.438089065,((((40:6.004628434,39:6.004628434):4.814608873,41:10.819237306000002):2.451622727,((36:7.3570095747,37:7.357009574):0.5260800951,38:7.88308966956):5.3877703633):0.7745788023,42:14.045438834999999):1.053304520321):1.601727186,((34:5.867130958,33:5.867130958):2.902766922,35:8.7698978797):7.930572661):18.529950472):2.9773245164553):1.935566871,(((((((24:9.620208865099999,23:9.620208865):6.577396438,(21:5.305719231,22:5.305719231):10.891886072):1.289405245,(19:6.268475462,20:6.26847546231):11.21853509):1.892602197,(26:12.36876984,25:12.36876984):7.0108429050300005):6.080098174,27:25.45971092):2.829727701,28:28.289438620400002):9.8293596158,((53:9.162334041,(((((57:3.830500783,58:3.8305007827999997):1.696310698,59:5.5268114809):0.3034948279,(((61:2.441333639,62:2.4413336389999998):1.805088796,60:4.246422435):0.5051469046,63:4.751569339):1.078736969):2.527954573,54:8.358260881):0.5963700022,(56:4.787979710599999,55:4.7879797102):4.166651173):0.2077031575):3.932765236,(64:8.584776887,65:8.584776887):4.510322391):25.0236989641):2.024514165):2.3034453403999997,(66:32.95083423,(((74:11.22691651,(75:5.877917485,(77:5.366709427,76:5.3667094265):0.5112080582):5.348999022999999):4.213671676,73:15.440588187):0.65194075,((72:14.688051822,71:14.688051825999999):1.108233252,(((68:6.606670401,67:6.606670401000001):3.461001975,69:10.067672375999999):2.36673527,70:12.43440765):3.3618774289999998):0.2962438596):16.858305295999997):9.49592351):3.389731945,(78:38.190372765,((80:2.597829282,79:2.597829282):17.81106283,((((82:9.713722451999999,81:9.713722451999999):1.9419635215,83:11.655685973):3.127308975,(85:14.656631739,84:14.656631737800002):0.1263632099):0.7921687809,(86:7.346764292,87:7.346764292):8.2283994377):4.833728386):17.781480653):7.646116922):1.665080822,(((((((89:29.148608794999998,(90:8.643629345,91:8.643629345499999):20.504979445):4.536870024,88:33.6854788151):4.56431814,92:38.249796947):4.516961807,((((((((93:8.4579310023,94:8.4579310025):1.28495441,(98:6.282006024299999,((95:2.928848886,96:2.9288488859):2.095388583,97:5.024237469):1.2577685553):3.4608793880699995):4.420145305,(99:13.45493079,100:13.45493079):0.7080999283):2.4777222459,101:16.6407529585):0.395645444,102:17.036398407):9.49013329357,((((125:8.740376774275,124:8.7403767741):6.74245831924,123:15.4828350937):3.0059394767,((((105:4.156236022,104:4.156236022):10.97233932,103:15.1285753425):2.728138911,((((((106:9.21079479,107:9.2107947898):4.431330632,(((108:7.266584851999999,(109:0.4649171274,110:0.4649171274):6.80166772482):1.583027322,(111:4.134203051,112:4.1342030511):4.715409123):1.781892609,113:10.6315047852):3.0106206386000003):0.5655180643,(115:3.377282851,114:3.377282851):10.830360636):0.7898525722,(116:6.434384372999999,117:6.4343843722):8.56311168627):1.430442115,118:16.427938173999998):0.2496123283,(121:7.241623671599999,(120:2.184778777,119:2.184778777):5.0568448936):9.4359268314):1.1791637524):0.1298798545,122:17.9865941075):0.50218046017):0.9877614466,126:19.476536016):7.049995684):1.628523905,127:28.155055609999998):1.144564279,(129:8.4456370778,128:8.44563707772):20.853982813000002):13.46713887):2.747719553,130:45.514478309):0.8280759637,(132:27.21362757,131:27.21362758):19.128926699):0.4545534298,((((((143:8.054054157,144:8.054054157):15.32707923,(((((146:11.1189401547,145:11.118940155):4.715207269,160:15.834147420999999):1.2469784248,(148:13.9094943777,147:13.909494378):3.171631473):0.9331117033,(((158:8.068925875677,(157:6.226403475000001,(156:3.864605097,155:3.8646050962):2.361798378):1.8425224015000001):0.5288871994,(((152:2.1131957265,151:2.113195727):2.979073673,(154:0.4794501527,153:0.47945015262999996):4.6128192479):2.565178314,(150:6.009759288,149:6.009759288):1.647688426):0.9403653613):2.903399124,159:11.501212200000001):6.513025353):2.104571514,161:20.118809067):3.2623243229999996):2.089855189,((162:19.227311143,163:19.2273111428):3.622050419,(((171:11.39251092,(((164:7.5186397469,(166:1.008526316,165:1.008526316):6.5101134300000005):2.783697643,(168:2.691687352,167:2.691687352):7.610650036999999):0.6018928322,(169:2.386380297,170:2.3863802965):8.517849925):0.4882807019):2.805337797,(172:5.019281723,173:5.019281723):9.178566998):0.4437879811,((((((175:5.0780981597,176:5.078098159):0.4293615801,174:5.50745974):0.7258420662,177:6.233301806):0.701566031,178:6.934867837):1.851523285,179:8.786391122):1.987102131,(181:5.734880232,180:5.734880232):5.038613021):3.8681434489999997):8.2077248626628):2.621627021):8.942388348,(((((140:3.358407897,139:3.358407897):1.27368903,141:4.6320969272):3.513454396,142:8.145551322900001):11.455348628,(137:3.5064692303999996,138:3.5064692309999996):16.09443071962):6.2685507246,((135:4.840242738,(134:2.670610905,133:2.6706109048999997):2.169631833):0.8024603117,136:5.642703049):20.2267476237):8.543926255):1.424735446,(((185:7.189295775,184:7.189295775000001):3.3593603117,186:10.5486560864):12.065636092100002,(183:17.505808194,182:17.505808189):5.108483989):13.2238202):4.844589302,(187:0.06335428596,188:0.06335428596):40.6193473933762):6.114406023000001):0.7044628099):6.740100271999999);

TREE tree_6661 = ((18:19.20839935314,(((11:6.397950758,12:6.397950758):7.411449258,((1:10.82983859,2:10.829838593):0.9020512834,(((4:8.296893588,(((7:4.0290084771,8:4.0290084762):0.5388865075,9:4.567894985100001):1.616725714,(5:2.157573798,6:2.15757379763):4.0270469001):2.1122728895):1.535764893,10:9.832658481):0.30550791229999996,3:10.138166392999999):1.593723483):2.0775101393):3.17999563,((((14:3.357882511,15:3.357882511):1.9227768784000001,13:5.2806593899):0.1556347249,16:5.436294114):0.7660732248,17:6.2023673393):10.787028306):2.2190037142):32.7557982913,((((66:30.2709724,((((74:7.836242879,(75:6.482396532,(77:4.115035601,76:4.1150356007):2.367360931):1.3538463470900002):3.718329688,((69:8.452178539,(68:5.226622072,67:5.226622073):3.2255564669999996):1.788433232,70:10.24061177):1.3139607962):0.1404837101,(71:10.81316833,72:10.81316833):0.8818879475):0.9032750454,73:12.598331323):17.6726410786):7.6178679294,(((((52:14.806518261999999,((((48:3.815786242,49:3.815786242):3.3008274141,50:7.116613655199999):0.2336011252,51:7.350214781):2.359221197,(47:7.19949341,46:7.1994934099000005):2.509942568):5.09708228692):8.602340819,(((33:4.56981031,34:4.56981031):3.059567382,35:7.629377691):3.463853317,(((((40:3.863350508,39:3.8633505078):2.355678093,41:6.2190286008):2.086513512,((37:5.489844562,36:5.4898445616):0.2776341131,38:5.7674786749999996):2.538063439):0.724990893625,42:9.030533007639999):0.8117013063,((45:6.5731611022,44:6.573161101):1.525027179,43:8.09818828):1.744046033):1.250996695):12.315628076):2.1277706762976,(((((19:3.981244221,20:3.9812442201500002):9.036747687,((23:5.82402326,24:5.8240232602):6.1616739973,(21:4.95308336,22:4.95308336):7.032613896599999):1.032294651):1.889457124,(25:8.460796567,26:8.460796567000001):6.4466524649):4.149189057,27:19.056638085):1.913132451,28:20.96977054):4.5668592203):2.037601458,((30:14.962943732,(31:13.2799417879,32:13.279941787):1.6830019441):1.336987875,29:16.299931607):11.274299612730001):6.705734202,((65:13.428063089,64:13.428063090999999):4.928667316,((((57:4.249213427,58:4.249213427):3.250829754,59:7.5000431808000005):1.316689901,(((61:4.735032737,62:4.735032737):3.522343717,63:8.257376454):0.4518666613,60:8.709243115):0.107489967):4.624476745,(53:13.01771443,(54:11.29247864,(55:6.835983331,56:6.8359833308):4.456495309999999):1.725235792):0.4234953949):4.915520579):15.9232350097):3.60887491):4.579366679,(78:34.6253741717,((((84:15.823202376,((81:12.862908757000001,82:12.8629087566):0.6335155694,83:13.4964243302):2.3267780492):0.9674156704,85:16.790618046):1.35988293,(86:7.753980616,87:7.753980616):10.3965203593):2.581826174,(79:4.371222027,80:4.371222027):16.36110512):13.893047022):7.8428328377):3.783267131,((((((((90:8.124098222,91:8.124098222):15.286507819,89:23.410606047):9.064268783,88:32.47487482513):2.396273766,92:34.871148585):6.179067589,(((((((99:11.84339876,((94:7.5910571697,93:7.591057169389999):0.44880258907000004,(((95:2.25793663,96:2.2579366299):1.960103403,97:4.218040033):1.2903346255,98:5.5083746581):2.5314851010000003):3.803538996):0.2378372287,100:12.08123598):3.038393576,101:15.11962956017):0.5892931104,102:15.7089226705):8.988185149,(((((((((106:8.475466195,107:8.4754661947):4.462780631,(((108:5.3615833683,(109:0.3924852536,110:0.3924852536):4.9690981140999995):2.145572421,(111:3.854443945,112:3.8544439455):3.652711843):2.0914369336000003,113:9.598592722):3.3396541028):0.9677652626,(114:2.999381502,115:2.999381502):10.906630586):0.531271554,(117:4.9521173833,116:4.9521173832):9.485166259):0.8323236163,(((120:1.327670104,119:1.327670104):7.018492624,121:8.3461627288):6.334302532600001,118:14.680465262000002):0.5891419969):0.37849054468,((104:3.485527345,105:3.485527345):8.03878548,103:11.5243128205):4.123784978):0.3520538865,122:16.0001516884):1.274858356,(123:13.732480094000001,(125:9.1229952879,124:9.1229952871):4.60948480619):3.5425299521999998):0.501165604,126:17.77617565):6.9209321699999995):2.507656121,127:27.20476394):0.5495446897,(128:8.790081824,129:8.790081823100001):18.96422680664):13.29590756):1.268975651,130:42.3191918357):2.736837038,(131:25.327951650000003,132:25.327951654):19.728077219):0.5879429907,((((((143:8.005062165,144:8.005062165):18.19517968,(((((147:15.127919551,148:15.1279195507):2.12699539158,160:17.254914943):0.733674121,(((((150:6.664276179,149:6.6642761787):0.7750188741,((151:2.0642475912,152:2.0642475907):2.799198873,(154:0.332775662,153:0.332775662):4.530670802):2.575848589):1.578951666,158:9.01824671955):0.4353474801,((155:4.93900389,156:4.93900389):2.66343653,157:7.60244042):1.851153779):1.830543167,159:11.284137366):6.704451698):1.404419182,(145:14.2684554909,146:14.268455489199999):5.124552756):1.95962722,161:21.352635466000002):4.847606376):2.599408588,((162:15.052579057,163:15.052579053):10.89273207,(((171:15.54206812,(((164:10.31481816,(165:0.3236409259,166:0.3236409259):9.991177237):2.620865607,(168:4.33033517,167:4.33033517):8.605348600000001):0.5273940896,(169:2.846582661,170:2.8465826610000002):10.616495199):2.078990261):1.733632406,(173:6.255862311,172:6.255862311):11.019838216):0.5107464725,((179:9.269839025,(178:6.716385321,((174:5.275201124,177:5.2752011242):0.4647723618,(176:4.31661032,175:4.31661032):1.4233631657):0.9764118343):2.553453705):1.591085557,(181:6.957166986,180:6.957166986):3.903757595):6.925522418):8.1588641324743):2.854339302):4.035055553,((182:20.199992826,183:20.1999928224):2.147408043,((185:2.875542297,184:2.8755422974):3.070482916,186:5.94602521437):16.4013756548):10.487305107059798):2.51964153,(((137:6.6459130225,138:6.645913023):13.665187465,((141:8.520751674,(140:3.431142663,139:3.43114266304):5.089609011):3.294808337,142:11.815560012):8.495540471):4.010331195,(135:5.926171796,((134:4.30156639,133:4.30156639):1.480237633,136:5.7818040239999995):0.1443677726):18.395259881):11.03291584):2.417625847,(188:0.2968377522,187:0.2968377522):37.4751356186):7.871998501):0.6075022738):5.7127235138);

TREE tree_4795 = ((18:24.581217702560302,(((11:6.926677952,12:6.926677952):10.592082274,((1:14.08033258,2:14.080332576):0.7354388808,(((4:10.134299989999999,(((7:5.688560084100001,8:5.688560084200001):0.08796733876,9:5.776527422999999):1.283298271,(5:1.999953457,6:1.999953457):5.0598722363999995):3.074474297):1.09376984,10:11.2280698293):0.72113783981,3:11.949207669):2.8665637879999997):2.702988769):2.931224969,(((16:5.749893518,(14:2.679853183,15:2.6798531831):3.0700403356):0.2964505301,13:6.0463440493):1.221923775,17:7.2682678229):13.181717372000001):4.131232504810001):24.029059399,((((66:24.76775242,(((72:10.89566903,71:10.89566903):0.4100993304,(((69:7.1664646804,(68:4.64205614,67:4.642056141):2.5244085405):1.607937029,70:8.774401709):0.87991863,((77:3.94953675,75:3.94953675):3.829526877,(76:1.507203996,74:1.507203996):6.2718596302):1.87525671282):1.651448022):0.9660674674,73:12.271835828):12.495916591):10.756794163999999,(((((((19:5.809372605,20:5.809372605):9.091555061,((23:8.583623571,24:8.5836235719):5.7168352252000005,(21:5.64951691,22:5.64951691):8.6509418877):0.6004688703):1.667106511,(25:9.545756077,26:9.545756077):7.0222781012999995):4.47392579,27:21.0419599644):1.414777659,28:22.456737628):4.5517353125,(((32:12.593603417,31:12.5936034174):1.888000654,30:14.481604071):2.4872366206,29:16.9688406923):10.039632249):5.549451269,((((((44:10.032425657000001,45:10.032425657500001):2.145024309,43:12.177449967100001):1.743563369,(((38:7.202843977999999,(37:5.864970527,36:5.8649705267000005):1.3378734518000002):3.780112979,((39:6.075660711,40:6.075660711):1.629765897,41:7.7054266082):3.27753035):0.5503587017,42:11.533315659900001):2.3876976756):1.614888407,((34:5.361767419,33:5.361767419):2.293064084,35:7.654831504):7.881070239):13.527005404,(52:17.90196873942,(((50:9.986845930000001,51:9.986845930000001):0.08554418791,(49:6.51800032839,48:6.5180003289999995):3.554389789):4.286485853,(47:10.23311722,46:10.23311722):4.125758751):3.543092765):11.16093841):2.150722734,((64:10.42951156,65:10.429511558):2.920360475,(((54:8.689646867,(55:5.542386149,56:5.5423861493):3.147260717):1.14911862,(((57:3.491119913,58:3.4911199123):2.089517347,59:5.5806372600000005):0.7262532387,((60:3.45244759,(61:2.646018511,62:2.646018512):0.8064290787):1.052865634,63:4.505313224):1.801577274):3.531874989):0.6140966199,53:10.45286211):2.897009927):17.863757853000003):1.34429433):2.9666223743):4.170297799,(78:34.5480968859,((((85:15.402112915100002,((81:10.9170251995,82:10.91702519984):1.263709004,83:12.1807342):3.2213787104):0.1666226604,84:15.568735572000001):1.387319186,(86:8.549184766,87:8.549184766):8.406869995100001):2.946906551,(80:2.763897085,79:2.763897085):17.139064228000002):14.645135576):5.146747501):2.654238415,(((((((89:22.666633031,(90:5.660733714,91:5.6607337135):17.0058993212):4.374209614,88:27.04084264727):2.530637194,92:29.571479839200002):6.04278894,(((((101:19.709466510200002,(((98:9.5783242619,((95:4.900992151,96:4.900992152000001):2.227412926,97:7.128405077):2.4499191842999997):2.6331790533,(93:12.0718558072,94:12.071855808):0.13964750836):3.560108813,(100:14.97281731,99:14.97281731):0.7987948146):3.937854379):0.2798039679,102:19.9892704757):8.495379094637,((((125:10.8152259979,124:10.815225996999999):3.83665145281,123:14.651877449299999):3.4231592063000003,((((104:2.556714386,105:2.556714386):9.607348238,103:12.1640626296):4.037606513,((((((106:8.644357569,107:8.644357568999999):4.389031308,(((108:6.373675042,(109:0.2871972702,110:0.2871972702):6.0864777719):0.897108063,(111:4.032409052,112:4.0324090517):3.238374054):1.616151712,113:8.88693481728):4.1464540596):0.8163731901,(114:2.166450439,115:2.166450439):11.683311627):0.4630248759,(117:6.397154977,116:6.3971549769):7.9156319657):0.7647241453,118:15.077511088000001):0.1142309675,((120:2.347607913,119:2.347607913):6.8196880636,121:9.167295976599998):6.024446080000001):1.0099270814999999):0.08767932043,122:16.28934845358):1.785688198):0.1513574293,126:18.226394086):10.2582554848):2.337911538,127:30.8225611105):0.3859150501,(128:9.228386131,129:9.2283861311):21.980090029190002):4.405792616):5.293727415,130:40.907996186999995):0.7774920686,(132:27.883331862000002,131:27.88333186):13.802156394):0.1894760804,((((((143:7.00658499,144:7.00658499):16.92347294,(((((148:12.7966382609,147:12.796638260799998):1.54324645243,160:14.339884714):1.499277729,(((((156:6.685207926,155:6.685207926):0.8046036899,157:7.489811615):1.280896631,158:8.7707082452):0.7351844332,((((151:2.4128660718,152:2.4128660726):3.97634641,(153:1.055856836,154:1.055856836):5.333355646):2.893788609,150:9.283001091):0.06323708738,149:9.346238179):0.159654501):2.791757411,159:12.29765009):3.541512352):1.432030857,(146:12.21544342296,145:12.2154434265):5.055749876):2.882855451,161:20.15404875):3.776009181):2.936366608,((162:15.407265713,163:15.407265714000001):5.940954597,(((171:4.773946731,(((164:2.844123723,(165:0.2931092849,166:0.2931092849):2.551014438):0.7877497131,(168:0.9979916798,167:0.9979916797999999):2.6338817557):0.8484446554,(170:0.9123614876,169:0.9123614875999999):3.567956604):0.2936286398):1.725458361,(172:3.26782803,173:3.26782803):3.2315770626):0.7842866775,((181:3.576634225,180:3.5766342251):1.358400956,((((174:2.981457869,177:2.9814578693):0.1325181266,(175:2.651541469,176:2.6515414683):0.4624345273):0.5253675952929999,178:3.6393435912):0.658574498,179:4.297918089):0.6371170924):2.348656588):14.06452853845741):5.518204232):3.259957432,((183:16.3398352,182:16.339835198000003):4.735315655,((185:2.068656063,184:2.0686560631):5.19821038,186:7.26686644336):13.80828440645):9.051231117):0.9075818367,(((137:3.637636082,138:3.637636082):10.8488600399,(((140:1.685227771,139:1.6852277715000001):2.84446581,141:4.5296935813):4.079718402,142:8.609411983):5.877084139):4.378862529,((135:4.430356757,(134:1.470502646,133:1.470502646):2.959854111):0.4553121545,136:4.8856689106):13.979689742):12.16860516):4.535524913,(187:0.02193179061,188:0.02193179061):35.5475569292903):6.305475618):0.4741184628):6.2611942989);

TREE tree_2705 = ((((((1:17.23659783,2:17.236597827):0.3784520558,((10:15.014847284,((((7:6.0585727128,8:6.058572712809999):0.9637720425,9:7.02234475472):2.839216286,(6:1.784000531,5:1.784000531):8.077560511):2.326527539,4:12.188088580999999):2.82675870522):0.4203132974,3:15.435160584):2.1798892992):3.5634047339999997,(11:10.36538909,12:10.36538909):10.813065523999999):3.622550999,(((13:8.219310518,(14:4.865915046,15:4.8659150460000005):3.3533954713):0.3808458147,16:8.600156333000001):0.7437480169,17:9.3439043503):15.457101266999999):3.2645567128,18:28.06556233):18.7819217005,((((66:32.34968511,((((74:9.342422533,((77:4.02465278,76:4.02465278):2.413890439,75:6.438543219):2.9038793149):3.826804673,(70:11.35332804,(69:9.5333252276,(67:6.9054127990000005,68:6.9054128):2.627912429):1.820002812):1.8158991658):0.07051210844,(71:10.154659185,72:10.154659184):3.085080131):0.8663532204,73:14.106092539):18.243592574199997):3.5671048558000003,(((((((((39:4.419415645,40:4.4194156447):1.685710433,41:6.105126078):2.803368605,((36:5.4519771955,37:5.451977196):0.7932924618,38:6.245269657):2.663225026):0.2933045415,42:9.2017992247):0.9457703831,(43:9.245898879,(45:7.4383820219,44:7.438382022):1.807516857):0.9016707291):2.002063555,((34:3.867452958,33:3.867452958):2.838671975,35:6.706124933):5.443508231):13.366483394,(((((48:5.69539119,49:5.6953911899000005):2.909242906,51:8.604634096):0.2654800122,50:8.870114108400001):2.845270452,(47:8.706488269,46:8.7064882693):3.0088962912):6.896048944,52:18.611433504399997):6.904683052999999):2.388060141767,(((31:16.2253358719,32:16.225335869):2.3673993468999996,30:18.592735220999998):2.1866550808,29:20.7793902976):7.124786401000001):4.071531459,((28:21.873261845000002,(((((19:4.724323838,20:4.724323838):8.803211798,(21:4.75336593,22:4.75336593):8.774169707):0.08240714722,(23:7.206352237799999,24:7.2063522375):6.403590545999999):2.290631014,(26:9.088837562599998,25:9.088837562):6.811736235157):3.838912331,27:19.739486127600003):2.1337757165999998):6.8688375648057995,((64:11.2629797,65:11.262979696999999):5.160194605,(((63:7.287112318,(((61:3.137444948,62:3.137444949):1.709655661,60:4.847100609):1.529462469,((57:3.263050346,58:3.263050346):1.36183645,59:4.6248867967):1.751676282):0.9105492402):4.436909914,(54:10.66313181,(55:6.427536314,56:6.427536314999999):4.235595499):1.06089042):1.373520569,53:13.097542801):3.325631501):12.3189251057):3.233608744):3.941081811):1.555550726,(78:33.789920994,((((((82:7.510844794800001,81:7.510844794):2.153946855,83:9.66479165):3.147570634,84:12.812362283):0.4394849935,85:13.251847277):1.128334915,(87:7.015812,86:7.015812):7.364370191899999):1.864570038,(80:1.473315067,79:1.473315067):14.771437161):17.545168762):3.682419697):3.831571305,((((((((90:5.15184616,91:5.15184616):20.528120054,89:25.679966216):1.329072685,88:27.009038902800004):2.972199757,92:29.981238656200002):5.944617494,(((((101:16.0576263401,(((94:8.915718433,93:8.915718432699999):0.1141441661,(((95:3.698163182,96:3.698163182):2.304555748,97:6.00271893):0.35665211961000004,98:6.3593710496):2.67049154849):4.160925311,(99:12.31372594,100:12.31372594):0.877061972):2.8668384276000003):0.4905605363,102:16.5481868732):7.2486601757999995,(126:16.405104826,(((((((((107:7.5988606952,106:7.598860695400001):3.791975208,((((109:0.06098129401,110:0.06098129401):6.4607232836,108:6.5217045777):0.8626029911,(111:3.74938329,112:3.7493832903):3.634924278):1.739900325,113:9.12420789414):2.2666280099):0.9103585168,(114:2.915503042,115:2.915503042):9.385691378399999):0.6420612335,(117:5.0528378541,116:5.0528378548):7.890417799):1.412171734,118:14.355427388):0.1742587491,(121:7.214342070400001,(119:1.793707297,120:1.793707297):5.4206347745):7.3153440663):0.5552493455999999,((104:3.252542274,105:3.252542273):9.162747368,103:12.415289640000001):2.6696458400000003):0.1774969337,122:15.2624324171):0.8135392779,((124:9.674482305428999,125:9.67448230472):3.0597566171999997,123:12.734238921):3.3417327726):0.32913313134):7.391742225):2.784378968,127:26.5812260123):0.05324018555,(128:6.7059298759,129:6.7059298764):19.92853632413):9.291389951):3.103934856,130:39.029791014400004):0.6955538499,(131:27.6198856,132:27.619885594):12.105459267):0.7144506818,(((((((134:2.439377277,133:2.439377277):1.393702393,135:3.83307967):0.6406330543,136:4.4737127245199995):18.329154831,((142:12.603603991,((140:3.885460041,139:3.885460041):1.723994449,141:5.609454490999999):6.99414950098):5.1929029158999995,(138:5.488410996400001,137:5.488410997):12.308095910350001):5.006360652800001):5.68681189,(((143:7.003597779,144:7.003597779000001):19.7166308,(((((146:9.916848009999999,145:9.916848010999999):4.264619482,160:14.181467492):1.986502099,(148:13.27978265882,147:13.279782657950001):2.8881869342):1.035117083,((((149:6.408740504000001,150:6.4087405034):2.593961924,((152:2.1340411355,151:2.1340411357):2.143797647,(153:0.6171366844,154:0.6171366844):3.660702098):4.724863645):0.3068452488,((157:7.260086952,(155:3.726469218,156:3.7264692174):3.533617734):1.634927667,158:8.8950146181):0.4145330584):2.757574236,159:12.067121913000001):5.135964761):2.562255655,161:19.765342326):6.954886246):0.4302614448,((162:12.6998601724,163:12.699860172000001):12.37945672,((((((164:3.858578079,(166:0.3745611359,165:0.3745611359):3.484016944):2.290972995,(168:2.559371597,167:2.559371597):3.5901794770000004):0.9798340913,(169:1.964382113,170:1.9643821137):5.165003052):0.1167888029,171:7.246173968):1.608136327,(172:2.890943185,173:2.890943185):5.963367112):0.03368094817,((179:4.771497222,(178:3.6102848303,(((175:1.642756527,174:1.642756527):0.706496962,176:2.349253489):0.6163431429,177:2.9655966318):0.6446881989000001):1.161212392):2.248197729,(180:3.567051053,181:3.567051053):3.452643899):1.868296293):16.1913256409659):2.07117313):1.339189432):1.671597629,((182:12.587254362,183:12.5872543657):8.179549326,(186:7.920136221,(185:3.569809045,184:3.569809046):4.3503271761):12.846667466000001):9.3944733929):2.677283921,(187:0.1635460941,188:0.1635460941):32.6750149084):7.601234537):0.8641164585):5.5435720379);

TREE tree_7997 = (((((11:10.70902908,12:10.70902908):12.50391047,((1:17.81428249,2:17.81428249):1.637697113,(((((6:3.028401058,5:3.0284010588):7.969179084,((7:4.919888087,8:4.9198880874):1.77109682938,9:6.6909849167):4.3065952255):3.008651399,4:14.006231541):1.134710666,10:15.140942205):0.6492909003,3:15.790233106799999):3.661746497):3.7609599424999995):4.146479101,((((14:4.925506966,15:4.9255069661999995):3.0470521341000003,16:7.97255909928):0.578711955,13:8.5512710549):1.807867754,17:10.3591388085):17.000279839399997):5.735500051700001,18:33.0949187015):23.54682671334,((((66:33.5044739,(((71:16.862413446,72:16.862413441):2.019920658,(((69:13.5268231659,(68:7.1730254358,67:7.173025436):6.353797729):1.387794807,70:14.91461797):0.7787091845,(((75:6.126122357,76:6.126122357):4.873325284,77:10.999447641):1.49594929,74:12.49539693):3.1979302263):3.189006943):1.356186925,73:20.238521028999997):13.2659528748):12.434111415,((((29:21.0046870364,((32:15.079168927,31:15.079168927201998):1.5659853437,30:16.64515427):4.3595327659):9.498719614,(28:24.125247889999997,((((19:5.704639733,20:5.7046397338):9.035624095,((24:7.499500545,23:7.499500545):6.7508899509999996,(21:4.754626598,22:4.754626599000001):9.495763898):0.4898733322):3.113057317,(25:10.1950995768,26:10.195099578):7.6582215671):2.960384061,27:20.813705204999998):3.311542684):6.3781587599):4.239250549,((52:22.32901080079,((46:13.236590078999999,47:13.236590080000001):2.677838392,((50:11.22375946,51:11.22375946):0.2662481468,(48:7.702052702,49:7.702052702):3.787954905):4.424420865):6.414582333):11.016238207,(((33:4.69623622,34:4.69623622):4.183954874,35:8.880191093):7.633769662,(((((37:7.502474662,36:7.502474662):1.224536445,38:8.727011107):3.619611933,((40:5.718618953,39:5.718618953):3.07191425,41:8.7905332031):3.556089838):1.259846608,42:13.606469648400001):1.983197636,((45:10.447490500999999,44:10.447490501999999):2.764603745,43:13.2120942497):2.377573037):0.9242934708):16.8312882572):1.3974081875720001):3.261443594,((((((57:2.618203232,58:2.618203231946):4.402389772,59:7.02059300312):1.612976809,(63:7.758300614,((61:3.675535008,62:3.675535007):3.460570679,60:7.136105687):0.6221949273):0.8752691988):4.538805376,53:13.1723751917):0.1774781678,(54:11.31446661,(55:7.906417554,56:7.906417554):3.4080490534999996):2.03538675):9.490502633,(64:17.36995186,65:17.369951865):5.470404127):15.1637448052):7.93448452):2.567366681,(78:43.836633109,((((85:16.3684715455,84:16.368471545):0.04483618406,((81:11.700003936600002,82:11.7000039368):3.632554891,83:15.332558832):1.080748901):1.639565577,(87:9.800995872,86:9.800995872):8.251877433299999):3.453076881,(79:4.766547141,80:4.766547141):16.73940305):22.3306829183):4.669318881800001):3.422020743,((((((((90:11.090908227,91:11.090908227):21.7353956323,89:32.8263038595):4.208917045,88:37.035220904800006):4.681769424,92:41.716990333):2.90597957,(((((((100:14.70344476,99:14.70344476):2.221756021,(((98:7.4435706836,((95:3.775712492,96:3.7757124920000003):2.925254904,97:6.700967396):0.7426032873):4.8930814437,94:12.336652123):0.05664563618,93:12.393297762999998):4.531903022):4.087999386,101:21.0132001744):1.549791057,102:22.562991228):11.29482959173,((((((105:4.430368063,104:4.430368063):12.12153309,103:16.5519011567):5.669125621,(((((106:13.35203195,107:13.3520319545):3.319794569,(((108:8.860446137,(109:0.5876536526,110:0.5876536526):8.272792484):2.094956647,(111:6.423324034,112:6.4233240342):4.53207875):2.020692702,113:12.9760954865):3.6957310354):2.202231515,(114:3.523683231,115:3.523683231):15.350374806473898):0.5962878939,(117:8.544605528,116:8.5446055283):10.925740404):1.495332777,(((119:2.466478216,120:2.466478216):9.4276805106,121:11.894158727):8.8577616601,118:20.751920384):0.2137583217):1.2553480704):0.3412776112,122:22.5623043847):1.64993531,((125:14.313182551799999,124:14.313182552499999):6.5113338049000005,123:20.8245163589):3.3877233415999997):0.5652719446000001,126:24.777511642):9.0803091765):1.985460834,127:35.843281661):0.7094715936,(129:10.043965732799998,128:10.0439657319):26.5087875221):8.070216655):1.181227738,130:45.804197635):2.209603754,(131:24.738241525,132:24.738241526099998):23.275559881):2.143979184,((((((143:8.175846723,144:8.175846723):19.94826349,(((((((152:2.3610279837999997,151:2.3610279836):5.241896853,(153:0.5458859556,154:0.5458859556):7.0570388812000004):3.644811602,(149:8.627152632,150:8.6271526318):2.620583807):0.1479139218,(((155:5.54984722,156:5.54984722):2.857831227,157:8.407678447):2.313730121,158:10.7214085678):0.674241793):3.129124348,159:14.524774707999999):5.468880611,((160:17.0200257533,(147:16.086173455,148:16.0861734556):0.9338522979999999):1.22805254,(145:12.941548964,146:12.9415489587):5.306529334):1.745577026):2.668829009,161:22.6624843278):5.461625882):4.619381261,((162:21.15906587,163:21.1590658738):7.245714069,((((((164:7.338040735,(166:0.5303635465,165:0.5303635465):6.8076771879999995):1.662702908,(167:3.430217029,168:3.4302170283):5.570526614):2.835886448,(170:3.694007226,169:3.6940072258):8.142622865):2.503711881,171:14.34034197):1.464582081,(173:6.8888983573,172:6.8888983574):8.916025696):1.414101248,((((((174:4.218973266,176:4.218973266):1.562017193,177:5.780990459):0.5871518263,175:6.368142284999999):0.7788873269,178:7.147029612000001):2.032490769,179:9.179520381):2.378756215,(180:4.604546794,181:4.604546794):6.9537298023):5.660748705):11.1857546335):4.338711528):1.920180848,((((134:2.34157453,133:2.34157453):2.636657948,135:4.978232478):1.3991031,136:6.377335578299999):21.2081708481,((((140:8.5909818,139:8.590981801):2.1657039135,141:10.7566857134):6.164735278,142:16.921420986999998):8.324787477600001,(138:9.172810568,137:9.172810569):16.07339789612):2.3392979577):7.078165892):0.4101231716,((182:17.467123170999997,183:17.4671231712):6.2714423334700005,(186:6.8251624688999994,(184:2.710234663,185:2.7102346633):4.114927806):16.91340303573):11.335229989):4.41514327,(188:0.3979136229,187:0.3979136229):39.09102514):10.668841825):1.770192156):4.7137726755);

TREE tree_9885 = ((18:28.7575124489,((((1:16.52150923,2:16.521509234):0.7821053965,(((4:12.217674829,((5:2.763847073,6:2.763847073):7.033978916,(9:5.3665965459,(7:4.5854051699000005,8:4.58540516856):0.7811913764):4.4312294424):2.4198488401000002):2.027473045,10:14.2451478692):0.5449431506,3:14.7900910249):2.513523605):1.9093181743,(11:7.832381052,12:7.832381052):11.380551752):5.140376785,((16:7.975504297800001,((15:4.717121808,14:4.7171218074):2.304810389,13:7.021932197):0.9535721015999999):0.4983756248,17:8.4738799228):15.879429666):4.404202861):20.1782478323168,((((66:31.6909509,(((71:13.396870995999999,72:13.396870996):0.08968385411,((((77:1.19234419,75:1.19234419):4.050936378,76:5.243280568):3.895960101,74:9.139240668):3.854152827,(70:11.09817242,((68:4.243744382,67:4.243744382):4.373992980260001,69:8.6177373628):2.48043506):1.8952210725):0.493161355):0.9507164352,73:14.437271281):17.253679615000003):4.3391564201,(((((64:10.9228421618,65:10.9228421618):2.047203873,(53:10.28355364,((((57:3.803797533,58:3.8037975325):2.243342853,59:6.047140386000001):1.182630521,(((61:3.016610455,62:3.0166104549):2.647036847,63:5.663647301999999):0.4355735219,60:6.099220824):1.130550083):1.889894719,(54:8.352490912,(55:4.99524198,56:4.9952419794):3.357248932):0.7671747134):1.163888011):2.686492397):15.8064374067,((((((23:7.3022400697,24:7.3022400706):8.804440012,(22:5.5725436665,21:5.572543667):10.534136415999999):1.643059863,(19:4.895523972,20:4.8955239725):12.85421597):0.9929896258,(26:12.26554491849,25:12.265544918):6.477184653):4.940465033,27:23.683194605):1.722853647,28:25.406048249999998):3.3704351886):2.268930925,(((((((40:5.220230693,39:5.220230693):2.627299905,41:7.8475305982000005):3.359748429,((36:7.5600566771,37:7.5600566769999995):0.8178512253,38:8.3779079022):2.829371125):0.375539518,42:11.582818544):1.467630693,(43:11.40757491,(44:8.914582124,45:8.914582123999999):2.4929927882):1.6428743247000002):0.967541372,((34:4.124461706,33:4.124461705):4.121910595,35:8.246372301000001):5.7716183089):13.901220598,(((46:10.267908515,47:10.267908515):3.908420193,((49:6.003981221,48:6.003981221):4.803093165,(50:10.5329152573,51:10.5329152567):0.2741591293):3.369254321):4.159640709,52:18.335969413970002):9.583241791999999):3.1262031610000003):2.282992194,(((31:14.6925051457,32:14.692505146000002):2.9855328051,30:17.678037951):2.93360731,29:20.611645261):12.7167613):2.7017007509999997):4.163816215,(78:33.8827325837,((79:2.134550099,80:2.134550099):15.13529641,(((83:13.0230461,(82:10.1439263738,81:10.143926374000001):2.8791197273):1.650682608,(85:14.596438895,84:14.596438896):0.07728981294):0.4684891605,(86:6.25046715,87:6.25046715):8.8917507189):2.127628635):16.612886081):6.3111909456):3.05871102,((((((((((((((97:5.73980077,(95:3.027956045,96:3.0279560451):2.7118447251):0.5571308882,98:6.2969316585):3.0421430380000003,93:9.3390746972):0.1810034493,94:9.5200781466):3.145085047,(100:11.24332331,99:11.24332331):1.421839879):3.194929269,101:15.860092457299999):0.52341759428,102:16.383510056):7.589296084000001,(((((((((106:9.607067895,107:9.607067894):2.051458243,(((111:2.713056394,112:2.7130563938):5.15839896,((109:0.421727872,110:0.421727872):6.0735180791,108:6.495245951):1.376209403):0.5844726713,113:8.455928025):3.202598113):1.150002789,(114:2.216904363,115:2.216904363):10.591624565):0.727167705,(116:4.7487084345,117:4.74870843429):8.7869881978):0.898312469,((121:7.833960262500001,(120:1.528412952,119:1.528412952):6.3055473101):6.5672042044,118:14.401164467):0.03284463539):0.26721345173,((104:2.002727357,105:2.002727357):8.980436982,103:10.983164335200001):3.718058215):0.1647102988,122:14.8659328563):1.5947485843,((124:8.383513030400001,125:8.3835130306):4.58324516412,123:12.9667581941):3.4939232406):0.74321386143,126:17.203895298):6.7689108422):1.907458586,127:25.880264725):0.180950776,(128:7.9218432319000005,129:7.9218432314):18.139372267859997):11.02603616,((88:28.5357676318,(89:25.19365346,(90:8.701263015799999,91:8.701263016):16.492390444):3.3421141646):3.383640447,92:31.919408078399996):5.167843586):3.488127937,130:40.575379598):0.7057896727,(131:28.586060643,132:28.586060647):12.69510862):0.70971798,((((((143:7.322541088,144:7.322541088):16.51009831,((((146:10.900109559,145:10.900109559):4.904374032,((148:14.39137888263,147:14.3913788831):0.88229331965,160:15.273672202):0.5308113881):0.7479791673,(159:9.932876916000001,(((((153:0.3479103039,154:0.3479103039):4.336173113,(151:1.180440072,152:1.18044007241):3.5036433444000004):2.306836191,(150:4.773861899,149:4.773861899):2.217057709):0.2318495277,158:7.222769136710001):0.04001079641,((156:2.9307984721,155:2.930798473):3.523850401,157:6.454648873000001):0.8081310589):2.6700969851200003):6.619585841):2.176354417,161:18.728817175):5.103822221):3.206437204,((162:21.8669493384,163:21.866949337439998):2.282456169,((((((164:4.325043249,(165:0.3383775095,166:0.3383775095):3.9866657395000002):1.780369345,(167:1.961902971,168:1.961902971):4.143509624):1.155361249,(170:1.6932956761,169:1.6932956767):5.567478167):1.097905292,171:8.358679135):1.010274497,(173:2.974557685,172:2.974557685):6.394395947):0.4969865147,((179:6.67387361,((((175:4.047780357,174:4.0477803575):1.085021833,177:5.1328021904):0.06484250576,176:5.1976446957):1.3991858,178:6.5968304955):0.07704311376):0.6486701716,(180:3.445805342,181:3.445805342):3.8767384398):2.543396366):14.283465362889398):2.889671089):2.613548569,((((134:2.544222692,133:2.544222692):1.968799951,135:4.513022643):1.370088426,136:5.883111069):17.139229483,((((140:2.998181419,139:2.998181419):3.826500245,141:6.824681663000001):1.9434313418,142:8.7681130053):10.772202977100001,(138:6.815953659,137:6.8159536584):12.724362323999998):3.4820245694):6.630284618):1.083333721,((183:15.140581633,182:15.140581634999998):6.503319657,(186:5.6052059199999995,(185:2.137854865,184:2.137854865):3.4673510559):16.0386953709):9.092057599):2.792497873,(187:0.3691783262,188:0.3691783262):33.1592784319599):8.462430494):1.261747296):5.683125736);

TREE tree_7487 = ((((((1:15.62097566,2:15.620975657):1.779832484,(((4:12.337343028000001,((9:5.8158639782999995,(8:4.30675225373,7:4.306752254):1.5091117246999999):3.4061522254,(6:2.724990352,5:2.724990352):6.497025851):3.1153268238000003):1.687220932,10:14.0245639615):1.0536813981,3:15.078245358):2.3225627834):2.8662679708,(11:7.932215341,12:7.932215341):12.334860775):4.766519953,((16:8.054968745,((15:3.726969998,14:3.7269699979):3.8001080975800003,13:7.5270780959):0.5278906494):1.092933342,17:9.1479020879):15.885693977799999):4.799317466,18:29.8329135304):17.9646143235,(((((((((((19:6.353528834,20:6.353528833):8.99787451,(24:7.4968066391,23:7.496806639):7.8545967050000005):0.3825813021,(22:5.982719698,21:5.9827196979):9.751264948):1.481659445,(26:11.52140165,25:11.521401651):5.69424244):5.08238192,27:22.2980260141):2.433395205,28:24.731421216):4.668889294,((64:7.739888757,65:7.7398887573):4.908566682,(53:9.843155287,((54:8.779881785,(55:5.979438326,56:5.9794383256):2.80044346):0.4639626442,(((57:3.196121998,58:3.1961219969999997):1.589802708,59:4.785924706):0.6427606332,((63:4.238558169,60:4.238558169):1.013523737,(61:2.116161362,62:2.1161613620999997):3.135920543):0.1766034332):3.815159091):0.5993108573):2.805300152):16.7518550801):0.442906428,((((32:12.84111730088,31:12.841117300099999):2.2759602074,30:15.117077508):4.2357166184,29:19.3527941269):10.047519883,((((34:4.199882819,33:4.19988281904):3.429984133,35:7.629866951):5.499055919,(((((40:5.278034073,39:5.278034073):1.946019968,41:7.2240540407):2.664171781,((37:6.6886712710000005,36:6.688671271):0.331162357,38:7.019833628000001):2.868392195):0.7932374784,42:10.681463301):1.540243091,((45:7.1708010343,44:7.170801035):2.618946074,43:9.789747109):2.4319592830000003):0.9072164789):14.900976768,(52:17.816493510900003,(((48:5.834693157,49:5.834693157):3.472061273,(51:8.847125308,50:8.847125307299999):0.4596291217):3.842542909,(47:10.69450311,46:10.694503112):2.454794227):4.667196177):10.213406127999999):1.3704143671401):0.4429029375):4.888560271999999,(66:23.2647682,(((72:11.234853687,71:11.2348536879):0.575965796,(70:9.417245482,((68:3.968803918,67:3.968803918):5.1537950802,69:9.122598997599999):0.2946464841):2.3935740019000002):0.403161981,(((77:0.9577940475,75:0.9577940475):7.080040901,(74:2.811265582,76:2.811265582):5.226569367):3.753701626,73:11.791536574999999):0.4224448895):11.050786734999999):11.467009013):5.052930116,(((80:2.914553071,79:2.914553071):17.26966162,(((((81:11.4013592189,82:11.40135922):0.8882383738,83:12.289597592):1.304133156,85:13.593730749):0.9936286505,84:14.587359399):1.167675417,(86:10.09798962,87:10.09798962):5.6570452002):4.429179878):14.1556743892,78:34.339889083):5.444818244):2.070279788,(((((((89:23.885159897999998,(90:5.083240278,91:5.083240277):18.801919622):3.515177505,88:27.400337401599998):3.199308724,92:30.5996461263):8.079078813,(((((((100:11.75493008,99:11.75493008):2.404959514,((93:8.863717612810001,94:8.863717612000002):0.9861405404,(((95:2.241954142,96:2.2419541415):1.933863996,97:4.1758181375):2.38072964,98:6.5565477782):3.2933103756):4.310031438):3.9989984830000003,101:18.158888077):1.4226043046,102:19.581492379):5.534041723,(126:18.787095087,(((((105:3.576676538,104:3.576676538):9.409575227,103:12.986251769):3.406678674,((((((107:8.879156437999999,106:8.879156437):3.485735545,(((111:4.398383473,112:4.398383473):3.599079614,(108:6.40781142,(109:0.06441114685,110:0.06441114685):6.3434002729):1.5896516673):1.792901748,113:9.790364833800002):2.5745271473000004):0.6116553948,(115:1.686533415,114:1.686533415):11.29001396):0.2478136419,(116:6.505292559,117:6.505292559):6.719068459):1.842867,118:15.067228018):0.2502502559,(121:8.287132715,(120:1.958264915,119:1.958264915):6.328867799899999):7.0303455578):1.0754521653):0.6065786255,122:16.99950906):1.330052609,((125:8.3024963825,124:8.3024963827635):6.2661844139400005,123:14.568680793900002):3.7608808789000006):0.45753341300000006):6.328439013300001):2.23279751,127:27.348331610000002):0.3245698233,(128:7.7822575699,129:7.7822575708):19.890643869599998):11.00582351):1.780599861,130:40.4593248038):0.8267163718,(131:31.30744765,132:31.307447642000003):9.978593531000001):0.1946006153,((((((143:8.099888351,144:8.09988835):15.83571711,(((((146:12.7097381234,145:12.709738123000001):2.536528832,160:15.246266955):0.8042140487,(148:13.294925181900002,147:13.294925182):2.75555582194):0.7748341579,(((((150:7.278931549,149:7.2789315489):0.181559264,((153:0.8020911108,154:0.8020911108):3.84997267,(152:1.8491367766,151:1.8491367765):2.8029270047):2.808427032):0.3687108699,((155:3.023138437,156:3.023138437):3.208918393,157:6.232056829999999):1.597144853):0.2381172293,158:8.0673189129):2.680061783,159:10.747380695):6.077934466):2.030285424,161:18.85560058566):5.080004874):1.974807491,((162:15.1210006209,163:15.121000624):8.671908499,((((((164:6.948851458,(165:1.618865388,166:1.618865388):5.329986071):3.028210817,(167:1.765288635,168:1.765288635):8.21177364):1.490035781,(169:3.479299649,170:3.4792996489999997):7.987798408):1.169318106,171:12.63641616):2.222074653,(172:4.612615549,173:4.612615549):10.245875267999999):1.756670839,((179:8.123320086,(((176:4.151984645,(175:3.614766418,174:3.614766418):0.5372182274):0.7723326967,177:4.924317342199999):1.504781209,178:6.429098551):1.694221536):1.510116334,(181:3.197529492,180:3.1975294919):6.4359069284):6.981725235):7.177747473):2.11750383):5.304053783,((182:21.672637023,183:21.672637021):2.297278306,(186:7.4291274767,(185:2.4388171192000003,184:2.4388171192000003):4.990310357293):16.5407878536):7.244551409):1.137512075,(((135:2.527309473,(134:1.872737188,133:1.872737188):0.6545722841):0.2207569671,136:2.7480664400999997):17.5124959992,((137:4.71186051,138:4.711860510199999):13.013268124000001,(142:8.378287305299999,((139:3.3229462738,140:3.322946273):1.318386455,141:4.641332729):3.736954577):9.3468413282):2.5354338024):12.091416374000001):3.034376719,(188:0.1171970805,187:0.1171970805):35.2691584503415):6.094286259):0.3743453269):5.9425407324999995);

TREE tree_7750 = ((18:34.701427964570996,((((1:19.79945432,2:19.799454321000002):2.929452157,((4:17.879161623,((9:8.91761015432,(8:6.8074242294000005,7:6.8074242295000005):2.1101859246):3.649747148,(5:4.228969751,6:4.228969751):8.338387551):5.311804325000001):2.420749705,(10:18.461428055,3:18.4614280539):1.8384832791):2.428995144):1.3996816962,(11:12.34993574,12:12.34993574):11.778652426999999):5.098045054,((((15:6.269170407,14:6.269170407):4.626827426,13:10.8959978319):0.1559867327,16:11.05198457):0.2013396315,17:11.2533241975):17.973309028):5.4747947345000005):19.9894879638,((((66:29.64147817,((((76:6.409251208,77:6.409251208):4.349123865,(74:0.9928450085,75:0.9928450085):9.765530065):3.702995114,((((67:3.838154156,68:3.8381541558):3.7581466,69:7.596300757):3.38058406,70:10.97688482):2.671022573,(71:12.257315940000002,72:12.257315941000002):1.3905914493):0.8134627974099999):2.137521228,73:16.598891416):13.0425867556):11.910881486,((((64:11.05847755,65:11.058477547999999):3.135262422,(((((61:3.591926994,62:3.5919269934):3.800335158,(60:6.163436899,63:6.163436899000001):1.228825253):0.5273998521,((57:2.862768249,58:2.862768249):2.172574528,59:5.035342777):2.8843192259999997):2.870714881,(54:9.078046949,(55:6.533917742,56:6.533917741930001):2.5441292065):1.712329936):0.825334169,53:11.61571105):2.578028917):20.5630824023,((((33:5.061690738,34:5.061690738):3.901761116,35:8.963451854):7.156339309,(((((37:7.108580595,36:7.108580595):2.3153555947999997,38:9.42393619):2.591060528,((40:5.045934866,39:5.045934866):2.632382812,41:7.6783176782):4.33667904):1.169207772,42:13.184204491):1.13595207,((44:11.045434726,45:11.0454347257):1.873081655,43:12.9185163852):1.4016401797):1.799634602):16.553735372,(((47:12.8810914,46:12.881091398999999):2.419780007,((51:10.72303812,50:10.723038119):0.3910278482,(48:8.025202244,49:8.025202244):3.088863722):4.186805438):4.745870469,52:20.0467418701):12.626784664):2.0832958426):2.777666624,(((30:19.040375189,(32:15.6222041607,31:15.6222041579):3.4181710269):2.710501944,29:21.7508771284):13.7800804111,((((((21:6.875268158,22:6.875268159):9.794595244,(24:8.878581057,23:8.878581058):7.791282345):1.271518174,(19:5.261795676,20:5.2617956758):12.6795859):2.706423281,(26:13.0021207565,25:13.002120757):7.6456841013000005):4.942434281,27:25.590239138):4.790953055,28:30.381192193999997):5.149765346500001):2.003531467):4.0178706587):5.304056119,(78:37.683355297999995,((((85:17.541691655999998,84:17.541691661999998):0.8040453358,((82:11.712423892499999,81:11.712423891):1.659551427,83:13.371975324000001):4.973761677):0.368235486,(87:11.05775234,86:11.057752344):7.656220138):1.914645194,(80:1.898432114,79:1.898432114):18.730185567):17.054737625):9.173060479):1.384194497,((((((((90:7.254197949,91:7.254197949):19.2827831214,89:26.536981071):2.621461165,88:29.15844224):7.080286699,92:36.2387289379):5.272662402,(((((((((94:11.458619602,93:11.458619601099999):0.3189927453,(98:7.802185978400001,((95:3.295109519,96:3.295109519):3.04737406,97:6.34248358):1.4597023984):3.97542636869):2.556396823,99:14.33400917):0.1964519362,100:14.53046111):5.18722541,101:19.71768651156):0.9538189426000001,102:20.671505458000002):11.3922693116,(126:24.362549598,(((((((((106:11.00866208,107:11.008662082299999):5.883969832,(((111:6.446966731,112:6.446966731):4.583416205,(108:8.1846212607,(109:0.08679097853,110:0.08679097853):8.0978302819):2.845761675):2.241042285,113:13.27142522016):3.6212066934):0.6460623141,(114:2.43498668,115:2.43498668):15.103707548600001):1.05824345,(116:7.1126980248,117:7.1126980248):11.484239654):0.7398122482,118:19.336749926):0.1179838528,((120:1.936155032,119:1.936155032):8.133603132,121:10.069758165):9.384975614999998):0.8079718834,((104:3.8073236,105:3.8073236):10.32130957,103:14.12863317):6.1340724959):0.2827014112,122:20.5454070704):2.435041187,(123:17.29326119537,(125:9.9887177123,124:9.988717712):7.3045434843):5.6871870656):1.3821013393000001):7.701225170000001):3.409330272,127:35.47310505):0.004914339473,(128:9.890199182,129:9.890199182):25.5878201975):6.033371952):2.234155501,130:43.745546841999996):3.213398716,(131:37.93381086,132:37.933810861):9.025134692):0.5637155453,(((((((133:2.823601917,134:2.8236019177):1.580616038,135:4.404217954):1.1020341,136:5.506252055):15.987135523000001,(((141:4.53333222,(140:1.92550051,139:1.9255005098):2.6078317102999997):6.8046656003,142:11.337997821):8.8623325522,(137:5.612026578,138:5.612026577569999):14.588303793):1.2930572081):8.834311389,(((143:9.468112805,144:9.468112805):17.97901659,((((((((152:1.45991833172,151:1.459918332):3.581569947,(154:0.2426266378,153:0.2426266378):4.798861641):2.951124112,(150:7.937139838,((155:4.720350329,156:4.720350328):2.109571441,157:6.829921769):1.107218068):0.05547255258):0.3600930593,158:8.3527054494):0.1720523211,149:8.524757771):5.056454084,159:13.5812118554):6.457020818,((160:18.278211483,(147:14.357017695100001,148:14.357017695):3.9211937921):0.666538873,(145:12.260779359999999,146:12.2607793565):6.683971005):1.093482312):1.073062064,161:21.1112947331):6.33583466):0.276929863,((162:16.149321479,163:16.149321474):8.195909812,((((((164:4.15214152,(166:0.1198915745,165:0.1198915745):4.032249945):2.239426616,(167:1.710053493,168:1.710053493):4.681514644):2.312369929,(169:2.267057838,170:2.2670578386000004):6.436880228):0.3379405147,171:9.041878581):3.923463407,(172:4.39867945,173:4.3986794500999995):8.566662538000001):0.1951341489,((179:5.716341673,(178:5.426695677,((176:4.395717227,(175:3.394134791,174:3.394134791):1.001582436):0.01385509048,177:4.4095723173):1.01712336):0.2896459956):1.352792252,(181:3.524336367,180:3.524336367):3.544797558):6.091342211800001):11.1847551538278):3.378827978):2.603639703):2.805314519,((183:14.81261605,182:14.812616049999999):6.633975033,(186:4.27451375748,(185:2.559743077,184:2.5597430771000003):1.71477068061):17.1720773246):11.686422407):6.83425457,(187:0.1825147783,188:0.1825147783):39.7847532805):7.5553930422):0.7179491793):6.450305641);

TREE tree_0890 = ((((((1:21.20283255,2:21.202832557):0.7636946918,((10:16.911277591,(4:14.646314922000002,(((7:7.2540959349000005,8:7.2540959344):0.7563558281,9:8.0104517617):2.295532793,(6:3.069268679,5:3.069268679):7.236715876):4.3403303673):2.2649626696):2.715343499,3:19.626621087):2.3399061540000003):2.75794006,(11:9.189395072,12:9.189395072):15.535072228):5.221847355,((16:10.280589547,17:10.2805895466):1.17746678,(13:10.005461058,(14:5.017561234,15:5.017561234):4.987899824):1.452595269):18.4882583322):4.6713172012,18:34.61763186019999):20.393184242399997,((((66:25.45679677,((((72:9.786503357,71:9.786503356999999):0.8069396753,73:10.593443033):2.448665241,(((68:3.964808365,67:3.9648083654):4.9552063870000005,69:8.9200147519):0.6008238489,70:9.520838601):3.5212696735):1.370119906,((76:0.5942682833,74:0.5942682833):12.4071328,(75:7.541121544,77:7.541121543999999):5.4602795389):1.410827097):11.044568589999999):11.767076414,((((64:11.82687695,65:11.826876955000001):2.480853044,(53:10.86968357,((((60:6.024172141,63:6.024172141):0.3302552621,(61:3.218783937,62:3.218783937):3.135643466):2.009423663,((57:4.394775361,58:4.3947753606):3.268662136,59:7.663437497):0.7004135686):1.412432264,(54:8.68352355,(55:5.871555798,56:5.8715557976):2.8119677524):1.09275978):1.093400242):3.438046427):16.622052758,((((((19:5.889354609,20:5.889354608940001):7.96725044,(24:6.793426457900001,23:6.793426458):7.063178591):0.6014217855,(21:5.274314212,22:5.274314212):9.183712623):2.197404138,(26:9.3980887713,25:9.398088771000001):7.25734220248):4.87741936,27:21.532850333000003):1.079726773,28:22.612577105):8.317205654):3.038458096,((((((50:10.5270856344,(49:7.225326723,48:7.225326723):3.3017589114):0.3427239997,51:10.869809633):5.563572909,(47:12.264044066,46:12.264044065):4.169338478):3.9681047128999998,52:20.4014872614):11.367967181,((((((36:7.606136368,37:7.6061363678):0.8059691741,38:8.412105541999999):2.691198726,((39:7.109283261,40:7.109283261):1.763257659,41:8.8725409196):2.23076334846):0.8770019109,42:11.980306179):1.795924621,((44:8.315961567,45:8.315961567799999):3.933643877,43:12.24960544):1.5266253555999998):1.244721925,((34:6.037259277,33:6.037259277):2.45319833,35:8.490457608):6.530495118):16.748501713):1.7924912930999999,(((32:13.622994570300001,31:13.6229945701):2.6177118549,30:16.240706426):4.213712912,29:20.454419337):13.107526392):0.4062951306):3.25563233):7.52337461,(78:40.2607895904,((80:4.2558698539,79:4.255869854):14.86824032,(((85:15.021473671999999,84:15.021473671999999):0.1280751811,((81:10.069225883000001,82:10.06922588268):1.0849619605,83:11.154187843999999):3.995361009):1.408668567,(87:9.326888629,86:9.326888629):7.2313287912):2.565892752):21.136679415):4.486458213000001):2.931158721,(((((((89:30.384645791,(91:9.254279132999999,90:9.2542791329):21.1303666592):1.395519174,88:31.78016496947):5.455912608,92:37.2360775791):4.935119386,((((102:19.419544116999997,((((93:10.1218077521,(98:6.758483458400001,(97:5.770010631,(95:4.106880015,96:4.106880015):1.663130616):0.9884728271000001):3.3633242938):0.5693913468,94:10.6911990988):3.030441655,(99:11.48384414,100:11.48384414):2.237796612):4.1809117078,101:17.902552463400003):1.5169916546200002):9.928623413,(126:21.800807798,(((((105:3.206888662,104:3.206888662):11.56588301,103:14.7727716753):4.756711771,(((121:8.9892736206,(119:1.44858521,120:1.44858521):7.5406884096999995):9.38705455146,118:18.376328172):0.3856446142,((((106:8.902253239,107:8.90225323909):5.519233118,(((111:4.217062898,112:4.21706289665):4.532795294,(108:6.845055829,(109:0.1973659117,110:0.1973659117):6.6476899174):1.9048023624000001):2.329849886,113:11.07970807737):3.3417782784999996):0.3555492101,(114:3.129074049,115:3.129074049):11.647961516999999):2.366960435,(117:7.4886445815,116:7.4886445813):9.655351420999999):1.617976784):0.7675106611):0.7616081587,122:20.291091603799998):1.322367533,(123:16.40843103895,(124:11.5595472853,125:11.5595472856):4.84888375293):5.2050281005):0.18734865906):7.547359731999999):2.696462088,127:32.044629615199995):0.4954330633,(129:10.4818635051,128:10.4818635034):22.058199176800002):9.631134277):4.092511808,130:46.263708773999994):0.6449897596,(132:32.458514768,131:32.45851477):14.450183765):0.1783948397,((((((135:6.893694622,(133:4.415168733,134:4.415168733):2.478525889):3.482239429,136:10.375934051):21.624328120999998,((137:6.930959215,138:6.9309592147):18.7514734549,((141:7.325107095,(140:5.779317646,139:5.7793176458):1.5457894478):11.80878168,142:19.13388877):6.548543895):6.317829505):0.0003200058218,(((143:9.642767578,144:9.642767577):17.0913152,((((146:11.487758793,145:11.4877587886):5.223133417,160:16.710892209):1.741985015,((((((155:3.660488241,156:3.6604882406):2.834184046,157:6.494672287):2.2424541068,((149:5.05096511,150:5.0509651103):2.694995862,((151:1.247068718,152:1.24706871789):2.588329937,(153:0.1721038517,154:0.1721038517):3.663294804):3.910562316):0.9911654225):0.4408326436,158:9.1779590385):1.475455728,159:10.653414765):7.674494701,(147:14.210528666,148:14.210528667):4.1173808001):0.1249677593):3.475884259,161:21.9287614814):4.8053212940000005):0.7877112118,((162:17.387892452,163:17.387892447):4.82177422,((((((164:4.998859776,(166:0.3868499841,165:0.3868499841):4.612009792):1.5690342552,(168:2.422630332,167:2.422630332):4.1452636997):0.247563599,(169:0.8020435325,170:0.8020435324999999):6.013414099):1.179064335,171:7.994521965):1.122495737,(172:1.873628876,173:1.873628876):7.243388827):0.7722331681,((179:5.609819104,((((174:3.004983268,177:3.0049832682):0.6500044665,176:3.654987735):0.07949255571,175:3.734480291):0.6998833525,178:4.4343636426):1.175455461):0.7728466031,(181:2.992217281,180:2.992217281):3.3904484265):3.5065851640000005):12.320415798600001):5.312127315):4.478788194):2.143653122,((182:15.433386762800001,183:15.433386763):6.690372052,((184:3.468471188,185:3.468471188):5.354004069659999,186:8.822475257899999):13.3012835588):12.020476484):3.345191283,(188:0.1904625715,187:0.1904625715):37.298964014321):9.5976667804):0.5913131521):7.3324095853);

TREE tree_4815 = (((((11:9.124893331,12:9.124893331):12.933692927,((1:18.66391877,2:18.663918765):1.604166885,(((4:14.434011820999999,((6:3.432719802,5:3.4327198020000003):7.476241879,((7:6.84034626548,8:6.8403462662):1.36529338,9:8.205639646800002):2.7033220350000002):3.5250501400000003):1.100487741,10:15.534499563):1.3651294567,3:16.89962902):3.368456631):1.790500604):5.828803408,(((13:8.155790836,(14:3.432182564,15:3.432182564):4.7236082724):0.8462353843,16:9.002026220000001):1.310980685,17:10.3130069056):17.5743827523):5.575440949000001,18:33.462830611940205):24.512960714000002,((((66:32.29937315,((((70:13.2358767,(69:11.101546734,(67:6.069236752,68:6.069236752):5.032309983):2.134329965):2.293769445,((76:9.238373326,(77:7.5737865,75:7.5737865):1.6645868257999998):4.355837843,74:13.59421117):1.9354349755000002):2.092668275,(71:15.567762562999999,72:15.567762566999999):2.0545518524):0.3142149557,73:17.936529372):14.3628437747315):13.9642409,(((((((19:4.744990077,20:4.744990077400001):11.13514781,((21:5.502631315,22:5.5026313159999996):9.396079567,(23:6.640612701,24:6.6406127015):8.258098180000001):0.9814270012):2.501547351,(25:11.467885615,26:11.46788561501):6.913799619):6.754101025,27:25.135786262):2.350747335,28:27.486533594):5.8955503657,((30:16.134898865,(32:14.113339465,31:14.113339460959999):2.0215594052):3.759606665,29:19.8945055302):13.48757843):7.069022305,(((((54:13.58564699,(55:8.734236962,56:8.734236961999999):4.851410027):0.1037295216,(((57:4.724840376,58:4.7248403759999995):3.17661237,59:7.901452746):0.1270399282,(63:6.24404856,((61:4.306926629,62:4.3069266289999995):1.74566558,60:6.052592208):0.1914563517):1.784444114):5.660883837):0.4985739923,53:14.1879505):2.523001333,(64:12.96267922,65:12.962679212):3.74827262):17.6275254957,(((((((36:6.909796833,37:6.9097968322):0.807729659,38:7.7175264915):3.977733888,(41:9.097634004,(40:7.144602445,39:7.144602445):1.953031559):2.5976263762):0.5384250276,42:12.233685408):2.89303271,((45:8.3594197622,44:8.359419763):3.748461999,43:12.10788176):3.01883635642):1.531976549,((34:5.321602808,33:5.321602808):4.570368409,35:9.891971217):6.766723449):13.892872172,(52:21.041988384099998,((47:13.18768479,46:13.1876847936):2.9936433316,((49:6.722533368,48:6.7225333676):5.590952304,(51:12.131345051,50:12.13134505124):0.1821406213):3.8678424527):4.860660255):9.509578459):3.7869104918999996):6.1126289371):5.812507791):2.357678543,(78:42.054474764000005,((((85:17.944383043000002,((82:12.55914641,81:12.559146409):0.3725237731,83:12.9316701827):5.012712861):0.9192162202,84:18.863599265):0.279456964,(87:10.39311381,86:10.39311381):8.7499424171):2.129624007,(80:1.985832051,79:1.9858320513999999):19.28684818):20.781794532):6.566817833):1.631900912,((((((((90:7.982169584,91:7.982169583999999):21.1859541323,89:29.168123712):9.02448494,88:38.1926086502):6.017247253,92:44.209855908600005):2.474338775,((((102:21.30500335813,(101:20.48345918,(100:14.22499963,(((94:12.058216438999999,93:12.05821643897):0.3698024586,(((95:5.729195091,96:5.729195090999999):0.973428945,97:6.7026240364000005):2.838728389,98:9.5413524252):2.8866664726):1.282402248,99:13.71042115):0.514578489):6.258459541):0.8215441822):9.274159802,((((((((((106:10.66051466,107:10.6605146608):4.13807138813,(((108:7.9468520718999995,(109:0.4959672679,110:0.4959672679):7.450884804799999):1.386665392,(111:5.61498906,112:5.614989059999999):3.718528404):2.118443859,113:11.4519613223):3.3466247263):2.054751574,(115:3.104567182,114:3.104567182):13.748770441):1.11811845,(117:8.270276069000001,116:8.270276069):9.701180004000001):1.499369164,((120:1.480553929,119:1.480553929):7.789553635,121:9.27010756434):10.20071767293):0.4920232905,118:19.9628485309):0.8802506699,((105:3.145924579,104:3.145924579):13.71710587,103:16.863030452):3.980068747):0.4176520417,122:21.2607512421):2.0084767116,((124:9.4559222888,125:9.4559222883):7.6451260021,123:17.1010482925):6.168179659):0.6179837746,126:23.887211727):6.691951435):4.034033674,127:34.6131968314):0.5105461868,(128:11.365659953000002,129:11.36565994913):23.758083065900003):11.56045166):1.069376012,130:47.75357068674001):0.666308413,(132:40.99628462,131:40.99628462):7.4235944832000005):1.432410928,((((((135:3.037038948,(134:1.347965459,133:1.347965459):1.689073489):0.7421484676,136:3.7791874153):22.991878891,((142:11.436649079,((140:3.420361555,139:3.4203615554000004):2.000740049,141:5.421101602999999):6.0155474747):9.7655719765,(137:6.4472770310000005,138:6.4472770316000005):14.754944019):5.568845253959999):8.268422032,(((143:10.29932773,144:10.299327727):19.43789494,(((((146:13.854151311799999,145:13.854151310999999):4.229049283,160:18.083200593):1.755853918,(147:16.159711622,148:16.159711622):3.6793428902):0.6476497774,((((151:2.6629818080999996,152:2.6629818079):4.396632182,(154:0.4406346165,153:0.4406346165):6.618979373):4.137575278,((158:8.89269253742,((155:6.092735034,156:6.092735034):1.982337576,157:8.075072609500001):0.817619928087):0.9223820153,(149:7.060595635,150:7.0605956354999995):2.7544789175999997):1.382114714):2.413129034,159:13.610318301):6.87638599):3.49966605,161:23.98637034):5.7508523259999995):1.921702774,((162:22.997218416,163:22.997218424):4.13903094,((((((164:7.247440414,(166:0.5923562865,165:0.5923562865):6.655084127):4.508121912,(168:1.741172272,167:1.741172272):10.014390055):1.305996522,(169:2.22990679,170:2.2299067895):10.831652059):0.2590824489,171:13.3206413):2.573249212,(173:1.894168158,172:1.894168158):13.999722352):0.6647731334,((((((174:4.593619858,175:4.593619858):2.323984991,176:6.9176048487):0.7834013427,177:7.7010061920999995):1.339865455,178:9.040871647):1.018450255,179:10.0593219):1.884528934,(181:6.054536915,180:6.054536915):5.88931392):4.614812809):10.577585722):4.522676082):3.380562891):0.8032844729,((183:19.61036196,182:19.610361962000002):7.556515853,(186:9.050430453,(184:6.103351973,185:6.103351973):2.9470784807899997):18.1164473611):8.675894995):3.578907897,(188:0.8050467781,187:0.8050467781):38.616633927470104):10.430609325):0.4009034839):7.7225978091);

TREE tree_3775 = ((18:33.53062856990999,(((11:9.675821513,12:9.675821513):13.367429909999998,((1:19.80353824,2:19.803538237):1.721527686,(((4:13.413362328,((5:3.021366567,6:3.0213665671000003):7.242397955,((7:6.6846802284,8:6.6846802282399995):0.4210055837,9:7.105685811100001):3.158078711):3.1495978057):2.969414661,10:16.382776987):1.8297375662,3:18.212514556):3.3125513730000002):1.5181855004):5.180987004,(((16:8.488400378,(15:5.247153751,14:5.2471537506):3.2412466274):1.060238479,13:9.548638857):1.313407539,17:10.8620463967):17.3621920386):5.3063901393):25.108164642,((((((28:29.130923461000002,((((19:6.016519282,20:6.0165192829):11.987834,((24:10.871893230600001,23:10.871893231000001):6.683825689900001,(21:6.125613174,22:6.125613174):11.4301057459):0.4486343612):1.513171843,(26:14.112162567000002,25:14.112162566999999):5.4053625578):6.39899296,27:25.9165180818):3.2144053777):4.753927299,(29:19.763976899,((32:15.896564270999999,31:15.896564271299999):1.3571787022069999,30:17.25374297):2.5102339251999997):14.120873861):4.299055314,(((((33:3.195261836,34:3.1952618360000002):2.7629083850000002,35:5.958170222000001):3.948914396,(((44:5.970574787,45:5.970574786799999):2.230096084,43:8.200670871):1.09236632,((((39:3.91058003,40:3.91058003):2.129520879,41:6.0401009092):1.392907071,((37:4.3699237352,36:4.36992373526):1.074422714,38:5.444346449):1.988661532):0.5377715764,42:7.970779557):1.3222576337499998):0.6140474268):11.3967545463,(((((49:3.070635089,48:3.0706350884):3.104428864,51:6.175063952):0.08358294815,50:6.2586469006000005):3.06711197,(47:6.894542673,46:6.894542673):2.431216197):4.491033623,52:13.8167924955):7.48704667):1.2514486155,(((53:7.504432552,(((57:3.395198271,58:3.3951982712):1.657613896,59:5.052812167):0.6243243925,(60:4.399221144,(63:3.836860764,(61:2.011504886,62:2.0115048862):1.825355877):0.5623603803):1.277915415):1.827295993):1.350562641,(54:8.18272663,(55:4.655760176,56:4.65576017574):3.526966454):0.6722685641):3.29534929,(64:8.70997362,65:8.7099736201):3.440370863):10.4049432955):15.62861829):4.3138396245,(66:29.52653401,(((71:15.82190625,72:15.821906249469999):0.675788438,((70:14.1317783,(69:12.214084307499999,(68:7.588093125,67:7.588093125):4.625991181):1.917693992):1.617526954,((77:3.490739104,76:3.4907391048):6.216533574,(75:2.91611658,74:2.91611658):6.791156098):6.042032574):0.7483894356):0.6498946758,73:17.147589361):12.378944646340099):12.971211683):5.791213664,(78:40.6456327435,((((83:12.58665131,(81:11.680399893,82:11.680399892):0.90625141935):3.72384349,(85:15.051021374200001,84:15.051021369):1.259473428):0.9784366369,(87:8.478806381,86:8.478806381):8.8101250588):4.470253314,(80:3.381858641,79:3.381858641):18.377326112):18.886447982):7.643326625):2.543718054,(((((((((101:20.535374334,(((93:12.380594410099999,((97:7.078681289,(95:3.94858615,96:3.94858615):3.1300951382):0.9615795014,98:8.0402607898):4.3403336204):0.2142929711,94:12.594887382):2.53056987,(99:11.90299246,100:11.90299246):3.222464796):5.409917083):1.4630325126000001,102:21.998406847100004):9.299240093,(126:22.815165665,((123:17.665493093400002,(124:13.3330665197,125:13.333066518999999):4.332426573599999):4.6057522139,(((103:15.48165556,(104:3.923384918,105:3.923384918):11.5582706441):4.406786608,((((((106:10.53437181,107:10.534371814):4.813713117,((((109:0.23646019,110:0.23646019):8.0748711237,108:8.311331313):1.166640405,(111:4.753268469,112:4.753268469):4.724703249):1.939965833,113:11.417937551000001):3.9301473792):1.888495353,(114:3.440392137,115:3.440392137):13.796188146):1.016571084,(117:6.695772341,116:6.695772340802999):11.557379026200001):0.3431904837,118:18.596341851):0.2988491501,((120:1.587709101,119:1.5877091011):8.7566504492,121:10.344359551):8.5508314509):0.9932511694999999):0.8494103164,122:20.7378524838):1.53339282):0.5439203594):8.482481275):2.236143952,127:33.533790897):0.2652834168,(129:10.144086156299998,128:10.1440861564):23.654988153):10.44648066,((((90:9.689283131,91:9.68928313):20.2907581879,89:29.98004133):3.010304464,88:32.990345788199996):3.50702685,92:36.4973726416):7.748182334):1.820214509,130:46.065769481299995):3.521499454,(132:44.284215020000005,131:44.28421503):5.3030539026):0.2290081572,(((((((134:3.906560354,133:3.906560354):0.4290249427,135:4.3355852967):1.439008981,136:5.7745942775):23.588154408,((137:4.794018634,138:4.7940186344):20.3620041748,((141:4.49288549,(139:2.6079501560000002,140:2.6079501552):1.8849353345000002):7.325569884,142:11.818455374):13.337567435):4.2067258808):9.136170351,(((143:8.078837842,144:8.078837842):22.13036387,(((((148:17.7552981053,147:17.7552981041):1.8927871173000002,160:19.648085218200002):0.602916208,(146:14.581622838200001,145:14.581622834000001):5.669378591):0.9286194801,(((((156:4.237045257,155:4.237045257):4.763071032,157:9.00011629):1.028406154,158:10.028522443580002):0.3851175998,((150:8.842253576,149:8.842253575199999):0.4279790341,((151:2.888952391,152:2.8889523914999997):3.444578559,(154:0.6388389637,153:0.6388389637):5.6946919860000005):2.93670166):1.143407434):3.510756712,159:13.9243967564):7.255224154):4.349368312,161:25.528989227):4.680212486):3.646474042,((162:27.275923306899998,163:27.2759233064):3.950354105,((((((164:10.22478295,(165:0.9112753183,166:0.9112753183):9.313507636):2.525728636,(167:3.670163725,168:3.670163725):9.080347866):0.965594045,(169:3.544977607,170:3.544977607):10.171128028):2.088925406,171:15.805031041):3.854082304,(172:7.074915496,173:7.074915496):12.584197848999999):0.2857506401,((179:11.63368762,((177:8.443569835,((175:5.792898919,176:5.792898919):1.63512887,174:7.428027789):1.0155420462):0.7056164294,178:9.149186265):2.484501357):2.220957762,(181:3.146517717,180:3.146517717):10.708127667):6.090218601):11.281413427076899):2.629398341):4.643243289):1.930154368,((183:19.35917107,182:19.359171068000002):6.315986754,(186:8.577618088,(184:4.393077913,185:4.3930779131):4.1845401754):17.0975397349):14.753915585999998):3.272708985,(187:0.004706515931,188:0.004706515931):43.6970758766):6.1144947):1.016400324):7.806115802);
[truncated: 1,232,020 more chars]
