# Supplementary material for: Sexual selection, feather wear, and time constraints on the pre‐basic molt explain the acquisition of the pre‐alternate molt in European passerines
Source: Ecol Evol. 2022 Sep 6;12(9):e9260. doi: 10.1002/ece3.9260 (PMC9448967; doi:10.1002/ece3.9260)
Supplement: Supplementary file 2 — Figures S1–S6 [file ECE3-12-e9260-s005.docx]

Figure S1. Posterior distributions of rate parameters from the dependent model of correlated evolution between the occurrence of the pre-alternate molt and the strength of sexual selection in 188 European passerine species (corresponding to Figure 3A). The model was run 10 times over a sample of 100 phylogenetic trees (Appendix S1A), providing in each run 950 observations (9500 observations in total) drawn from 28,600,000 iterations of the Markov chain with a burn-in period of 100,000 and thinning interval of 30,000. Z-values represent the percentage of visits assigned as zero in the post-convergence portion of the model. Note that the x-axis range differs among graphs. Average (± SE) autocorrelation coefficient of the log likelihood for the 10 runs was 0.071 (± 0.014).

Figure S2. Posterior distributions of rate parameters from the dependent model of correlated evolution between the occurrence of the pre-alternate molt and migratory behavior in 188 European passerine species (corresponding to Figure 3B). The model was run 10 times over a sample of 100 phylogenetic trees (Appendix S1A), providing in each run 950 observations (9500 observations in total) drawn from 2,000,000 iterations of the Markov chain with a burn-in period of 100,000 and thinning interval of 2000. Z-values represent the percentage of visits assigned as zero in the post-convergence portion of the model. Average (± SE) autocorrelation coefficient of the log likelihood for the 10 runs was 0.009 (± 0.005).

Figure S3. Posterior distributions of rate parameters from the dependent model of correlated evolution between the occurrence of the pre-alternate molt and aerial foraging in 188 European passerine species (corresponding to Figure 3C). The model was run 10 times over a sample of 100 phylogenetic trees (Appendix S1A), providing in each run 950 observations (9500 observations in total) drawn from 2,000,000 iterations of the Markov chain with a burn-in period of 100,000 and thinning interval of 2000. Z-values represent the percentage of visits assigned as zero in the post-convergence portion of the model. Average (± SE) autocorrelation coefficient of the log likelihood for the 10 runs was 0.083 (± 0.014).

Figure S4. Posterior distributions of rate parameters from the dependent model of correlated evolution between the occurrence of the pre-alternate molt and winter territoriality in 188 European passerine species (corresponding to Figure 3D). The model was run 10 times over a sample of 100 phylogenetic trees (Appendix S1A), providing in each run 950 observations (9500 observations in total) drawn from 2,000,000 iterations of the Markov chain with a burn-in period of 100,000 and thinning interval of 2000. Z-values represent the percentage of visits assigned as zero in the post-convergence portion of the model. Average (± SE) autocorrelation coefficient of the log likelihood for the 10 runs was 0.011 (± 0.007).

Figure S5. Posterior distributions of rate parameters from the dependent model of correlated evolution between the extent of the pre-alternate molt (partial or complete) and sexual dichromatism in 83 European passerine species (corresponding to Figure 5A). The model was run 10 times over a sample of 100 phylogenetic trees (Appendix S1C), providing in each run 950 observations (9500 observations in total) drawn from 2,000,000 iterations of the Markov chain with a burn-in period of 100,000 and thinning interval of 2000. Z-values represent the percentage of visits assigned as zero in the post-convergence portion of the model. Average (± SE) autocorrelation coefficient of the log likelihood for the 10 runs was 0.033 (± 0.013).

Figure S6. Posterior distributions of rate parameters from the dependent model of correlated evolution between the extent of the pre-alternate molt (partial or complete) and migratory behavior in 83 European passerine species (corresponding to Figure 5B). The model was run 10 times over a sample of 100 phylogenetic trees (Appendix S1C), providing in each run 950 observations (9500 observations in total) drawn from 7,700,000 iterations of the Markov chain with a burn-in period of 100,000 and thinning interval of 8000. Z-values represent the percentage of visits assigned as zero in the post-convergence portion of the model. For *q*13, *q*21, *q*24, and *q*31 two plots are shown, one with the full x-axis range and the other showing only a small range close to zero so that the number of zero values can be visually checked. Average (± SE) autocorrelation coefficient of the log likelihood for the 10 runs was 0.069 (± 0.011).
