# Supplementary material for: Sexual selection, feather wear, and time constraints on the pre‐basic molt explain the acquisition of the pre‐alternate molt in European passerines
Source: Ecol Evol. 2022 Sep 6;12(9):e9260. doi: 10.1002/ece3.9260 (PMC9448967; doi:10.1002/ece3.9260)
Supplement: Supplementary file 3 — Table S1 [file ECE3-12-e9260-s004.docx]

Table S1. References (in addition to Birds of the Western Palearctic (Cramp and Perrins 1988-1994) and the Handbook of the Birds of the World Alive (del Hoyo et al. 2020)) consulted in search of information on winter gregariousness for some of the passerine species included in the study. Scientific species names follow Gill and Donsker (2018).

| Species | Reference |
| --- | --- |
| *Anthus richardi* | Atahan A, Gül O, Atahan M, Gül M. 2015. Unusual wintering records of pipits (Aves: Motacillidae) in Hatay, eastern Mediterranean region of Turkey. Turkish J Zool. 39:74-79. |
| *Arundinax aedon* | Baker K. 1997. Warblers of Europe, Asia and North Africa. London, UK: Christopher Helm. |
| *Carpodacus roseus* | Clement P, Harris A, Davis J. 1993. Finches and sparrows. An identification guide. London, UK: Christopher Helm. |
| *Cercotrichas galactotes* | Clement P, Rose C. 2015. Robins and chats. London, UK: Christopher Helm. |
| *Emberiza rutila* | Byers C, Olsson U, Curson J. 1995. Buntings and sparrows. A guide to the buntings and North American sparrows. Robertsbridge, UK: Pica Press. |
| *Locustella certhiola* | Baker K. 1997. Warblers of Europe, Asia and North Africa. London, UK: Christopher Helm. |
| *Locustella fasciolata* | Kennerley P, Pearson D. 2010. Reed and bush warblers. London, UK: Christopher Helm. |
| *Phylloscopus proregulus* | Baker K. 1997. Warblers of Europe, Asia and North Africa. London, UK: Christopher Helm. |
| *Sylvia hortensis* | Baker K. 1997. Warblers of Europe, Asia and North Africa. London, UK: Christopher Helm. |
| *Sylvia nana* | Baker K. 1997. Warblers of Europe, Asia and North Africa. London, UK: Christopher Helm. |
| *Sylvia nisoria* | Baker K. 1997. Warblers of Europe, Asia and North Africa. London, UK: Christopher Helm. |
| *Sylvia ruppeli* | Baker K. 1997. Warblers of Europe, Asia and North Africa. London, UK: Christopher Helm. |
| *Turdus obscurus* | Iqbal M, Ajiman, Noske RA, Setiawan D. 2014. Hunting of a very large aggregation of eyebrowed thrushes *Turdus obscurus* in Sumatra. Kukila 17: 68-71. |
| *Zoothera sibirica* | Andrew P. 1985. An annotated checklist of the birds of the Cibodas-Gunung Gede Nature Reserve. Kukila 2: 10-28. |

References

Cramp S, Perrins CM, editors. 1988-1994. Handbook of the birds of Europe, the Middle East and North Africa: the birds of the Western Palearctic. Vols. V-IX. Oxford, UK: Oxford University Press.

del Hoyo J, Elliott A, Sargatal J, Christie DA, de Juana E, editors. 2020. Handbook of the birds of the world alive. Barcelona, Spain: Lynx Edicions. https://birdsoftheworld.org/, retrieved on November 2020.

Gill F, Donsker D, editors. 2018. IOC world bird list (v8.1). http://www.worldbirdnames.org/.
