# Supplementary material for: Sexual selection, feather wear, and time constraints on the pre‐basic molt explain the acquisition of the pre‐alternate molt in European passerines
Source: Ecol Evol. 2022 Sep 6;12(9):e9260. doi: 10.1002/ece3.9260 (PMC9448967; doi:10.1002/ece3.9260)
Supplement: Supplementary file 4 — Table S2 [file ECE3-12-e9260-s001.docx]

Table S2. Occurrence of the pre-alternate molt when molt in November or December is considered pre-alternate (pre-alternate molt from November; 1 = yes, 0 = no; partial or complete when present) or when it is considered pre-basic (pre-alternate molt from January), log_10_-transformed body mass (g), sexual selection index, sexual dichromatism, migration (1 = resident, 2 = resident to short distance, resident to partially migratory, or resident to eruptive, 3 = altitudinal, short-distance, partial migrant, or resident to migratory, 4 = migratory to short-distance, or migratory to resident, 5 = migratory), aerial foraging (0 = unimportant, 1 = important, 2 = the most important foraging technique), type of habitat (1 = desert, 2 = savannah, steppe, cliffs, or high mountain, 3 = scrub, tundra, or grassland, 4 = riparian area, groves, wetland, 5 = open woodlands, 6 = forest), and winter gregariousness (1 = territorial, 2 = non-gregarious, 3 = moderately gregarious, 4 = gregarious) for the 188 passerine species included in the study.

| Species (Gill and Donsker 2018) | Species (Jetz et al. 2012) | Pre-alternate molt from November | Pre-alternate molt from January | Body mass | Sexual select. | Sexual dichrom. | Migrat. | Aerial foraging | Type of habitat | Winter gregar. |
| --- | --- | --- | --- | --- | --- | --- | --- | --- | --- | --- |
| *Acanthis flammea* | *Carduelis flammea* | 0 | 0 | 1.085 | 0.133 | 51.96 | 4 | 0 | 3 | 4 |
| *Acanthis hornemanni* | *Carduelis hornemanni* | 0 | 0 | 1.139 | -0.327 | 29.96 | 3 | 0 | 3 | 4 |
| *Acrocephalus agricola* | *Acrocephalus agricola* | 0 | 0 | 0.982 | -0.319 | 19.91 | 5 | 0 | 4 | 2 |
| *Acrocephalus arundinaceus* | *Acrocephalus arundinaceus* | 1 complete | 0 | 1.477 | 0.951 | 15.29 | 5 | 1 | 4 | 2 |
| *Acrocephalus dumetorum* | *Acrocephalus dumetorum* | 0 | 0 | 1.049 | 0.600 | 9.70 | 5 | 1 | 4 | 2 |
| *Acrocephalus melanopogon* | *Acrocephalus melanopogon* | 1 partial | 1 | 1.037 | -0.519 | 26.46 | 3 | 0 | 4 | 2 |
| *Acrocephalus paludicola* | *Acrocephalus paludicola* | 1 complete | 1 | 1.064 | 2.958 | 34.10 | 5 | 0 | 4 | 2 |
| *Acrocephalus palustris* | *Acrocephalus palustris* | 1 complete | 1 | 1.061 | 0.622 | 16.40 | 5 | 0 | 4 | 2 |
| *Acrocephalus schoenobaenus* | *Acrocephalus schoenobaenus* | 1 complete | 1 | 1.081 | 0.700 | 32.93 | 5 | 0 | 4 | 1 |
| *Acrocephalus scirpaceus* | *Acrocephalus scirpaceus* | 1 complete | 1 | 1.090 | 1.174 | 22.54 | 5 | 1 | 4 | 2 |
| *Aegithalos caudatus* | *Aegithalos caudatus* | 0 | 0 | 0.934 | -0.389 | 9.30 | 1 | 0 | 5 | 4 |
| *Alauda arvensis* | *Alauda arvensis* | 0 | 0 | 1.586 | -0.055 | 35.81 | 4 | 0 | 3 | 4 |
| *Alauda leucoptera* | *Melanocorypha leucoptera* | 0 | 0 | 1.656 | -0.102 | 39.88 | 3 | 0 | 2 | 4 |
| *Alaudala rufescens* | *Calandrella rufescens* | 0 | 0 | 1.382 | -0.200 | 21.12 | 2 | 0 | 2 | 4 |
| *Anthus campestris* | *Anthus campestris* | 1 complete | 1 | 1.362 | 0.398 | 8.32 | 5 | 0 | 2 | 2 |
| *Anthus cervinus* | *Anthus cervinus* | 1 partial | 1 | 1.320 | -0.328 | 46.77 | 5 | 0 | 3 | 4 |
| *Anthus pratensis* | *Anthus pratensis* | 1 partial | 1 | 1.265 | 0.245 | 33.99 | 2 | 0 | 3 | 3 |
| *Anthus richardi* | *Anthus richardi* | 1 complete | 1 | 1.508 | -0.339 | 16.62 | 5 | 0 | 3 | 3 |
| *Anthus spinoletta* | *Anthus spinoletta* | 1 partial | 1 | 1.378 | 0.315 | 23.58 | 4 | 0 | 2 | 1 |
| *Anthus trivialis* | *Anthus trivialis* | 1 partial | 1 | 1.369 | 0.162 | 31.71 | 5 | 0 | 5 | 2 |
| *Arundinax aedon* | *Acrocephalus aedon* | 1 complete | 0 | 1.350 | -0.127 | 37.47 | 5 | 0 | 4 | 2 |
| *Bombycilla garrulus* | *Bombycilla garrulus* | 0 | 0 | 1.735 | -0.559 | 14.05 | 3 | 1 | 6 | 4 |
| *Bucanetes githagineus* | *Bucanetes githagineus* | 0 | 0 | 1.292 | -0.368 | 32.68 | 2 | 0 | 1 | 4 |
| *Calandrella brachydactyla* | *Calandrella brachydactyla* | 0 | 0 | 1.317 | -0.269 | 31.37 | 5 | 0 | 2 | 4 |
| *Calcarius lapponicus* | *Calcarius lapponicus* | 1 partial | 1 | 1.445 | 0.964 | 90.56 | 5 | 0 | 3 | 4 |
| *Calliope calliope* | *Luscinia calliope* | 0 | 0 | 1.267 | -0.296 | 68.36 | 5 | 0 | 5 | 2 |
| *Carduelis carduelis* | *Carduelis carduelis* | 0 | 0 | 1.204 | -0.341 | 20.25 | 3 | 0 | 4 | 4 |
| *Carduelis citrinella* | *Carduelis citrinella* | 0 | 0 | 1.079 | -0.443 | 37.29 | 3 | 0 | 2 | 4 |
| *Carpodacus erythrinus* | *Carpodacus erythrinus* | 1 complete | 0 | 1.380 | 0.678 | 74.09 | 5 | 0 | 5 | 4 |
| *Carpodacus roseus* | *Carpodacus roseus* | 0 | 0 | 1.456 | -0.537 | 60.66 | 3 | 0 | 6 | 4 |
| *Cercotrichas galactotes* | *Erythropygia galactotes* | 0 | 0 | 1.307 | -0.230 | 10.96 | 5 | 0 | 4 | 2 |
| *Certhia brachydactyla* | *Certhia brachydactyla* | 0 | 0 | 0.914 | 0.157 | 23.84 | 1 | 0 | 5 | 2 |
| *Certhia familiaris* | *Certhia familiaris* | 0 | 0 | 0.954 | 0.148 | 12.92 | 2 | 0 | 6 | 2 |
| *Cettia cetti* | *Cettia cetti* | 1 partial | 1 | 1.125 | 1.502 | 20.69 | 4 | 0 | 3 | 1 |
| *Chloris chloris* | *Carduelis chloris* | 0 | 0 | 1.415 | 0.086 | 36.88 | 3 | 0 | 5 | 4 |
| *Cinclus cinclus* | *Cinclus cinclus* | 0 | 0 | 1.787 | 0.907 | 5.81 | 2 | 0 | 4 | 1 |
| *Coccothraustes coccothraustes* | *Coccothraustes coccothraustes* | 0 | 0 | 1.753 | -0.515 | 36.44 | 3 | 0 | 6 | 4 |
| *Coloeus dauuricus* | *Corvus dauuricus* | 0 | 0 | 2.318 | -0.379 | 26.87 | 3 | 0 | 5 | 4 |
| *Coloeus monedula* | *Corvus monedula* | 0 | 0 | 2.391 | -0.366 | 25.25 | 3 | 0 | 5 | 4 |
| *Corvus corax* | *Corvus corax* | 0 | 0 | 2.974 | -0.422 | 25.38 | 1 | 0 | 5 | 3 |
| *Corvus corone* | *Corvus corone* | 0 | 0 | 2.756 | -0.332 | 44.88 | 1 | 0 | 4 | 3 |
| *Corvus frugilegus* | *Corvus frugilegus* | 0 | 0 | 2.657 | -0.279 | 38.42 | 3 | 0 | 5 | 4 |
| *Cyanistes caeruleus* | *Parus caeruleus* | 0 | 0 | 1.023 | 0.726 | 10.06 | 1 | 0 | 5 | 4 |
| *Cyanistes cyanus* | *Parus cyanus* | 0 | 0 | 1.166 | -0.391 | 12.85 | 1 | 0 | 4 | 3 |
| *Cyanopica cyanus* | *Cyanopica cyanus* | 0 | 0 | 1.982 | -0.356 | 16.25 | 1 | 0 | 5 | 4 |
| *Delichon urbicum* | *Delichon urbicum* | 1 complete | 0 | 1.161 | -0.572 | 15.07 | 5 | 2 | 3 | 4 |
| *Emberiza aureola* | *Emberiza aureola* | 0 | 0 | 1.292 | -0.194 | 88.40 | 5 | 0 | 4 | 4 |
| *Emberiza bruniceps* | *Emberiza bruniceps* | 0 | 0 | 1.394 | 1.552 | 110.44 | 5 | 0 | 2 | 4 |
| *Emberiza caesia* | *Emberiza caesia* | 1 partial | 1 | 1.282 | -0.221 | 53.26 | 5 | 0 | 5 | 4 |
| *Emberiza calandra* | *Miliaria calandra* | 0 | 0 | 1.688 | 1.675 | 35.53 | 2 | 0 | 5 | 4 |
| *Emberiza chrysophrys* | *Emberiza chrysophrys* | 0 | 0 | 1.255 | -0.250 | 77.14 | 5 | 0 | 5 | 4 |
| *Emberiza cia* | *Emberiza cia* | 0 | 0 | 1.370 | -0.219 | 52.79 | 2 | 0 | 3 | 3 |
| *Emberiza cioides* | *Emberiza cioides* | 0 | 0 | 1.323 | -0.285 | 34.65 | 2 | 0 | 5 | 4 |
| *Emberiza cirlus* | *Emberiza cirlus* | 1 partial | 1 | 1.408 | -0.307 | 55.98 | 5 | 0 | 5 | 3 |
| *Emberiza citrinella* | *Emberiza citrinella* | 0 | 0 | 1.473 | -0.247 | 59.60 | 3 | 0 | 5 | 4 |
| *Emberiza hortulana* | *Emberiza hortulana* | 1 partial | 1 | 1.299 | 0.242 | 36.29 | 5 | 0 | 5 | 3 |
| *Emberiza leucocephalos* | *Emberiza leucocephalos* | 0 | 0 | 1.455 | -0.172 | 109.88 | 5 | 0 | 5 | 4 |
| *Emberiza melanocephala* | *Emberiza melanocephala* | 1 complete | 0 | 1.453 | 1.704 | 113.65 | 5 | 0 | 3 | 4 |
| *Emberiza pallasi* | *Emberiza pallasi* | 0 | 0 | 1.146 | -0.260 | 131.43 | 4 | 0 | 3 | 4 |
| *Emberiza pusilla* | *Emberiza pusilla* | 1 partial | 1 | 1.166 | -0.270 | 17.10 | 5 | 0 | 3 | 4 |
| *Emberiza rustica* | *Emberiza rustica* | 1 partial | 1 | 1.313 | -0.291 | 54.91 | 5 | 0 | 5 | 4 |
| *Emberiza rutila* | *Emberiza rutila* | 0 | 0 | 1.243 | -0.335 | 74.15 | 5 | 0 | 5 | 4 |
| *Emberiza schoeniclus* | *Emberiza schoeniclus* | 1 partial | 1 | 1.266 | 0.877 | 118.09 | 3 | 0 | 4 | 4 |
| *Emberiza spodocephala* | *Emberiza spodocephala* | 0 | 0 | 1.268 | -0.306 | 48.68 | 5 | 0 | 5 | 4 |
| *Eremophila alpestris* | *Eremophila alpestris* | 0 | 0 | 1.528 | -0.119 | 42.48 | 4 | 0 | 3 | 4 |
| *Erithacus rubecula* | *Erithacus rubecula* | 0 | 0 | 1.248 | -0.461 | 7.91 | 4 | 0 | 5 | 2 |
| *Ficedula albicollis* | *Ficedula albicollis* | 1 partial | 1 | 1.104 | 0.682 | 93.84 | 5 | 2 | 6 | 2 |
| *Ficedula hypoleuca* | *Ficedula hypoleuca* | 1 partial | 1 | 1.143 | 0.578 | 85.73 | 5 | 2 | 6 | 2 |
| *Ficedula parva* | *Ficedula parva* | 1 partial | 1 | 0.996 | -0.548 | 99.74 | 5 | 1 | 6 | 2 |
| *Fringilla coelebs* | *Fringilla coelebs* | 0 | 0 | 1.342 | -0.137 | 77.58 | 3 | 0 | 6 | 4 |
| *Fringilla montifringilla* | *Fringilla montifringilla* | 0 | 0 | 1.365 | -0.234 | 91.88 | 5 | 0 | 5 | 4 |
| *Galerida cristata* | *Galerida cristata* | 0 | 0 | 1.621 | -0.204 | 22.38 | 1 | 0 | 2 | 3 |
| *Galerida theklae* | *Galerida theklae* | 0 | 0 | 1.582 | -0.202 | 27.99 | 1 | 0 | 2 | 3 |
| *Garrulus glandarius* | *Garrulus glandarius* | 0 | 0 | 2.220 | -0.470 | 28.05 | 2 | 0 | 6 | 2 |
| *Geokichla sibirica* | *Zoothera sibirica* | 0 | 0 | 1.878 | -0.467 | 66.98 | 5 | 0 | 6 | 3 |
| *Helopsaltes certhiola* | *Locustella certhiola* | 1 complete | 1 | 1.158 | -0.262 | 24.92 | 5 | 0 | 3 | 2 |
| *Helopsaltes fasciolatus* | *Locustella fasciolata* | 1 complete | 1 | 1.434 | -0.344 | 18.58 | 5 | 0 | 3 | 2 |
| *Hippolais icterina* | *Hippolais icterina* | 1 complete | 1 | 1.121 | -0.292 | 11.36 | 5 | 0 | 5 | 1 |
| *Hippolais olivetorum* | *Hippolais olivetorum* | 1 complete | 0 | 1.243 | -0.387 | 20.01 | 5 | 0 | 4 | 1 |
| *Hippolais polyglotta* | *Hippolais polyglotta* | 1 complete | 0 | 1.041 | -0.353 | 14.36 | 5 | 0 | 5 | 1 |
| *Hirundo rustica* | *Hirundo rustica* | 1 complete | 0 | 1.246 | 0.657 | 15.37 | 5 | 2 | 3 | 4 |
| *Iduna caligata* | *Hippolais caligata* | 0 | 0 | 0.949 | -0.382 | 16.83 | 5 | 0 | 4 | 1 |
| *Iduna pallida* | *Hippolais pallida* | 1 complete | 0 | 0.954 | -0.327 | 20.00 | 5 | 0 | 3 | 1 |
| *Lanius collurio* | *Lanius collurio* | 1 complete | 1 | 1.454 | 0.046 | 48.09 | 5 | 1 | 5 | 2 |
| *Lanius excubitor* | *Lanius excubitor* | 1 partial | 1 | 1.796 | 0.084 | 6.83 | 4 | 1 | 5 | 1 |
| *Lanius minor* | *Lanius minor* | 1 complete | 1 | 1.667 | -0.499 | 25.57 | 5 | 1 | 5 | 1 |
| *Lanius nubicus* | *Lanius nubicus* | 1 partial | 0 | 1.294 | -0.658 | 28.73 | 5 | 1 | 5 | 1 |
| *Lanius senator* | *Lanius senator* | 1 complete | 0 | 1.556 | -0.425 | 37.54 | 5 | 1 | 5 | 1 |
| *Linaria cannabina* | *Carduelis cannabina* | 0 | 0 | 1.291 | 0.117 | 47.89 | 3 | 0 | 3 | 4 |
| *Linaria flavirostris* | *Carduelis flavirostris* | 0 | 0 | 1.188 | -0.376 | 42.07 | 3 | 0 | 3 | 4 |
| *Locustella fluviatilis* | *Locustella fluviatilis* | 1 complete | 1 | 1.207 | -0.536 | 20.37 | 5 | 0 | 4 | 2 |
| *Locustella lanceolata* | *Locustella lanceolata* | 1 complete | 1 | 1.025 | -0.484 | 24.47 | 5 | 0 | 4 | 2 |
| *Locustella luscinioides* | *Locustella luscinioides* | 1 complete | 0 | 1.142 | -0.231 | 18.76 | 5 | 0 | 4 | 2 |
| *Locustella naevia* | *Locustella naevia* | 1 complete | 1 | 1.124 | -0.412 | 29.70 | 5 | 0 | 3 | 2 |
| *Lophophanes cristatus* | *Parus cristatus* | 0 | 0 | 1.043 | -0.327 | 19.14 | 1 | 0 | 6 | 4 |
| *Loxia curvirostra* | *Loxia curvirostra* | 0 | 0 | 1.590 | -0.355 | 71.75 | 2 | 0 | 6 | 4 |
| *Loxia leucoptera* | *Loxia leucoptera* | 0 | 0 | 1.409 | 0.272 | 71.53 | 2 | 0 | 6 | 4 |
| *Lullula arborea* | *Lullula arborea* | 0 | 0 | 1.430 | -0.312 | 28.93 | 3 | 0 | 5 | 3 |
| *Luscinia luscinia* | *Luscinia luscinia* | 0 | 0 | 1.377 | -0.442 | 15.30 | 5 | 0 | 5 | 2 |
| *Luscinia megarhynchos* | *Luscinia megarhynchos* | 0 | 0 | 1.286 | -0.439 | 20.13 | 5 | 0 | 5 | 2 |
| *Luscinia svecica* | *Luscinia svecica* | 1 partial | 1 | 1.221 | 0.767 | 71.34 | 5 | 0 | 3 | 2 |
| *Melanocorypha bimaculata* | *Melanocorypha bimaculata* | 0 | 0 | 1.733 | -0.116 | 23.91 | 5 | 0 | 2 | 4 |
| *Melanocorypha calandra* | *Melanocorypha calandra* | 0 | 0 | 1.790 | 0.055 | 13.77 | 1 | 0 | 2 | 4 |
| *Melanocorypha yeltoniensis* | *Melanocorypha yeltoniensis* | 0 | 0 | 1.779 | 0.099 | 243.92 | 3 | 0 | 2 | 4 |
| *Monticola saxatilis* | *Monticola saxatilis* | 1 partial | 1 | 1.702 | -0.352 | 105.53 | 5 | 1 | 2 | 2 |
| *Monticola solitarius* | *Monticola solitarius* | 1 partial | 1 | 1.730 | -0.414 | 76.46 | 3 | 1 | 2 | 2 |
| *Montifringilla nivalis* | *Montifringilla nivalis* | 1 partial | 1 | 1.567 | -0.322 | 45.62 | 2 | 0 | 2 | 4 |
| *Motacilla alba* | *Motacilla alba* | 1 partial | 1 | 1.360 | -0.274 | 28.97 | 4 | 1 | 3 | 3 |
| *Motacilla cinerea* | *Motacilla cinerea* | 1 partial | 1 | 1.235 | -0.453 | 83.63 | 3 | 1 | 4 | 3 |
| *Motacilla citreola* | *Motacilla citreola* | 1 partial | 1 | 1.284 | -0.230 | 71.89 | 5 | 1 | 3 | 4 |
| *Motacilla flava* | *Motacilla flava* | 1 partial | 1 | 1.246 | -0.358 | 77.18 | 5 | 1 | 3 | 4 |
| *Muscicapa dauurica* | *Muscicapa dauurica* | 0 | 0 | 0.991 | -0.410 | 32.03 | 4 | 2 | 5 | 2 |
| *Muscicapa striata* | *Muscicapa striata* | 1 complete | 1 | 1.201 | -0.033 | 39.30 | 5 | 2 | 5 | 2 |
| *Nucifraga caryocatactes* | *Nucifraga caryocatactes* | 0 | 0 | 2.240 | -0.451 | 30.20 | 1 | 0 | 6 | 2 |
| *Oenanthe deserti* | *Oenanthe deserti* | 0 | 0 | 1.298 | -0.142 | 68.43 | 5 | 0 | 3 | 1 |
| *Oenanthe hispanica* | *Oenanthe hispanica* | 1 partial | 1 | 1.179 | 0.773 | 52.68 | 5 | 1 | 3 | 1 |
| *Oenanthe isabellina* | *Oenanthe isabellina* | 1 partial | 1 | 1.468 | 0.896 | 13.50 | 5 | 1 | 2 | 1 |
| *Oenanthe leucura* | *Oenanthe leucura* | 0 | 0 | 1.613 | -0.210 | 32.34 | 1 | 0 | 2 | 3 |
| *Oenanthe oenanthe* | *Oenanthe oenanthe* | 1 partial | 1 | 1.409 | 0.775 | 40.73 | 5 | 1 | 2 | 2 |
| *Oenanthe pleschanka* | *Oenanthe pleschanka* | 1 partial | 1 | 1.288 | -0.391 | 148.49 | 5 | 1 | 2 | 2 |
| *Oriolus oriolus* | *Oriolus oriolus* | 1 ♂com ♀par | 0 | 1.898 | -0.511 | 105.85 | 5 | 0 | 5 | 3 |
| *Panurus biarmicus* | *Panurus biarmicus* | 0 | 0 | 1.141 | 0.219 | 57.97 | 2 | 0 | 4 | 4 |
| *Parus major* | *Parus major* | 0 | 0 | 1.234 | 0.132 | 30.88 | 1 | 0 | 6 | 4 |
| *Passer domesticus* | *Passer domesticus* | 0 | 0 | 1.424 | -0.419 | 110.46 | 1 | 0 | 4 | 4 |
| *Passer hispaniolensis* | *Passer hispaniolensis* | 0 | 0 | 1.384 | -0.379 | 140.53 | 2 | 0 | 4 | 4 |
| *Passer montanus* | *Passer montanus* | 0 | 0 | 1.330 | -0.452 | 34.04 | 1 | 0 | 5 | 4 |
| *Pastor roseus* | *Sturnus roseus* | 0 | 0 | 1.865 | -0.439 | 19.08 | 5 | 0 | 2 | 4 |
| *Periparus ater* | *Parus ater* | 0 | 0 | 0.964 | -0.414 | 21.19 | 2 | 0 | 6 | 4 |
| *Perisoreus infaustus* | *Perisoreus infaustus* | 0 | 0 | 1.926 | -0.453 | 20.44 | 1 | 0 | 6 | 4 |
| *Petronia petronia* | *Petronia petronia* | 0 | 0 | 1.480 | -0.412 | 24.31 | 1 | 0 | 2 | 4 |
| *Phoenicurus ochruros* | *Phoenicurus ochruros* | 0 | 0 | 1.217 | -0.420 | 101.92 | 3 | 1 | 2 | 2 |
| *Phoenicurus phoenicurus* | *Phoenicurus phoenicurus* | 0 | 0 | 1.164 | 0.632 | 100.61 | 5 | 1 | 6 | 2 |
| *Phylloscopus bonelli* | *Phylloscopus bonelli* | 1 complete | 0 | 0.940 | -0.231 | 11.18 | 5 | 1 | 5 | 1 |
| *Phylloscopus borealis* | *Phylloscopus borealis* | 1 complete | 1 | 1.037 | 0.846 | 7.05 | 5 | 1 | 6 | 2 |
| *Phylloscopus collybita* | *Phylloscopus collybita* | 1 partial | 1 | 0.919 | 0.945 | 31.66 | 4 | 1 | 6 | 3 |
| *Phylloscopus inornatus* | *Phylloscopus inornatus* | 1 partial | 1 | 0.814 | -0.384 | 46.96 | 5 | 1 | 6 | 2 |
| *Phylloscopus proregulus* | *Phylloscopus proregulus* | 1 partial | 1 | 0.778 | 0.133 | 15.20 | 5 | 1 | 6 | 2 |
| *Phylloscopus sibilatrix* | *Phylloscopus sibilatrix* | 1 complete | 1 | 0.964 | 0.810 | 13.01 | 5 | 1 | 6 | 1 |
| *Phylloscopus trochiloides* | *Phylloscopus trochiloides* | 1 complete | 1 | 0.898 | -0.296 | 31.46 | 5 | 1 | 5 | 1 |
| *Phylloscopus trochilus* | *Phylloscopus trochilus* | 1 complete | 1 | 0.940 | 1.002 | 13.41 | 5 | 1 | 5 | 2 |
| *Pica pica* | *Pica pica* | 0 | 0 | 2.316 | -0.334 | 21.78 | 1 | 0 | 5 | 3 |
| *Pinicola enucleator* | *Pinicola enucleator* | 0 | 0 | 1.751 | -0.489 | 86.16 | 3 | 0 | 6 | 4 |
| *Plectrophenax nivalis* | *Plectrophenax nivalis* | 1 partial | 1 | 1.625 | -0.195 | 91.26 | 4 | 0 | 3 | 4 |
| *Poecile cinctus* | *Parus cinctus* | 0 | 0 | 1.057 | -0.408 | 10.72 | 1 | 0 | 6 | 4 |
| *Poecile lugubris* | *Parus lugubris* | 0 | 0 | 1.199 | -0.510 | 27.35 | 1 | 0 | 6 | 3 |
| *Poecile montanus* | *Parus montanus* | 0 | 0 | 1.045 | -0.480 | 10.30 | 1 | 0 | 6 | 4 |
| *Poecile palustris* | *Parus palustris* | 0 | 0 | 1.039 | -0.262 | 15.39 | 1 | 0 | 5 | 1 |
| *Prunella collaris* | *Prunella collaris* | 0 | 0 | 1.593 | -0.382 | 22.46 | 1 | 0 | 2 | 3 |
| *Prunella modularis* | *Prunella modularis* | 0 | 0 | 1.297 | -0.465 | 16.20 | 4 | 0 | 5 | 2 |
| *Ptyonoprogne rupestris* | *Hirundo rupestris* | 0 | 0 | 1.310 | -0.610 | 36.52 | 3 | 2 | 2 | 4 |
| *Pyrrhocorax graculus* | *Pyrrhocorax graculus* | 0 | 0 | 2.325 | -0.303 | 30.72 | 2 | 0 | 2 | 4 |
| *Pyrrhocorax pyrrhocorax* | *Pyrrhocorax pyrrhocorax* | 0 | 0 | 2.444 | -0.209 | 34.70 | 1 | 0 | 2 | 4 |
| *Pyrrhula pyrrhula* | *Pyrrhula pyrrhula* | 0 | 0 | 1.387 | -0.452 | 40.68 | 3 | 0 | 6 | 3 |
| *Regulus ignicapilla* | *Regulus ignicapilla* | 0 | 0 | 0.748 | -0.282 | 19.31 | 2 | 1 | 5 | 3 |
| *Regulus regulus* | *Regulus regulus* | 0 | 0 | 0.744 | -0.370 | 17.86 | 3 | 1 | 6 | 3 |
| *Remiz pendulinus* | *Remiz pendulinus* | 1 partial | 1 | 0.968 | 2.371 | 52.30 | 4 | 0 | 4 | 4 |
| *Riparia riparia* | *Riparia riparia* | 1 complete | 0 | 1.105 | -0.632 | 31.69 | 5 | 2 | 3 | 4 |
| *Saxicola rubetra* | *Saxicola rubetra* | 1 partial | 1 | 1.220 | -0.408 | 31.90 | 5 | 1 | 3 | 2 |
| *Saxicola torquatus* | *Saxicola torquatus* | 0 | 0 | 1.187 | 0.655 | 69.71 | 3 | 1 | 3 | 2 |
| *Serinus serinus* | *Serinus serinus* | 0 | 0 | 1.049 | -0.209 | 61.53 | 3 | 0 | 4 | 4 |
| *Sitta europaea* | *Sitta europaea* | 0 | 0 | 1.310 | -0.463 | 15.18 | 1 | 0 | 6 | 1 |
| *Spinus spinus* | *Carduelis spinus* | 0 | 0 | 1.122 | -0.516 | 104.35 | 4 | 0 | 6 | 4 |
| *Sturnus vulgaris* | *Sturnus vulgaris* | 0 | 0 | 1.892 | 0.677 | 29.40 | 5 | 0 | 5 | 4 |
| *Sylvia atricapilla* | *Sylvia atricapilla* | 1 partial | 1 | 1.223 | 0.346 | 63.10 | 3 | 0 | 5 | 3 |
| *Sylvia borin* | *Sylvia borin* | 1 complete | 0 | 1.260 | 0.554 | 7.25 | 5 | 0 | 5 | 3 |
| *Sylvia cantillans* | *Sylvia cantillans* | 1 partial | 1 | 0.982 | -0.447 | 49.35 | 5 | 0 | 3 | 2 |
| *Sylvia communis* | *Sylvia communis* | 1 partial | 1 | 1.179 | 0.639 | 29.00 | 5 | 0 | 5 | 1 |
| *Sylvia conspicillata* | *Sylvia conspicillata* | 1 partial | 1 | 0.954 | -0.576 | 28.62 | 3 | 0 | 3 | 2 |
| *Sylvia curruca* | *Sylvia curruca* | 1 partial | 1 | 1.060 | -0.518 | 10.82 | 5 | 0 | 4 | 3 |
| *Sylvia hortensis* | *Sylvia hortensis* | 1 complete | 1 | 1.340 | -0.528 | 46.00 | 5 | 0 | 4 | 3 |
| *Sylvia melanocephala* | *Sylvia melanocephala* | 1 partial | 1 | 1.068 | -0.468 | 61.94 | 2 | 0 | 3 | 2 |
| *Sylvia nana* | *Sylvia nana* | 0 | 0 | 0.940 | -0.577 | 17.59 | 3 | 0 | 1 | 2 |
| *Sylvia nisoria* | *Sylvia nisoria* | 1 partial | 1 | 1.352 | 1.220 | 31.75 | 5 | 0 | 4 | 2 |
| *Sylvia ruppeli* | *Sylvia rueppelli* | 1 partial | 1 | 1.109 | -0.504 | 108.02 | 5 | 0 | 3 | 2 |
| *Sylvia sarda* | *Sylvia sarda* | 1 partial | 1 | 0.991 | -0.341 | 13.33 | 2 | 0 | 3 | 1 |
| *Sylvia undata* | *Sylvia undata* | 1 partial | 1 | 1.033 | -0.493 | 36.07 | 3 | 0 | 3 | 1 |
| *Tarsiger cyanurus* | *Tarsiger cyanurus* | 0 | 0 | 1.129 | -0.300 | 70.10 | 5 | 1 | 6 | 2 |
| *Tichodroma muraria* | *Tichodroma muraria* | 1 partial | 1 | 1.246 | -0.347 | 109.20 | 2 | 1 | 2 | 1 |
| *Troglodytes troglodytes* | *Troglodytes troglodytes* | 0 | 0 | 0.968 | 0.783 | 14.09 | 4 | 0 | 5 | 1 |
| *Turdus iliacus* | *Turdus iliacus* | 0 | 0 | 1.787 | -0.400 | 26.44 | 4 | 0 | 6 | 4 |
| *Turdus merula* | *Turdus merula* | 0 | 0 | 2.051 | -0.327 | 51.81 | 3 | 0 | 5 | 3 |
| *Turdus obscurus* | *Turdus obscurus* | 0 | 0 | 1.797 | -0.255 | 61.00 | 5 | 0 | 6 | 3 |
| *Turdus philomelos* | *Turdus philomelos* | 0 | 0 | 1.831 | -0.487 | 25.79 | 3 | 0 | 6 | 2 |
| *Turdus pilaris* | *Turdus pilaris* | 0 | 0 | 2.025 | -0.395 | 16.32 | 5 | 0 | 6 | 4 |
| *Turdus ruficollis* | *Turdus ruficollis* | 0 | 0 | 1.919 | -0.436 | 39.30 | 5 | 0 | 6 | 4 |
| *Turdus torquatus* | *Turdus torquatus* | 0 | 0 | 2.037 | -0.443 | 40.49 | 4 | 0 | 6 | 4 |
| *Turdus viscivorus* | *Turdus viscivorus* | 1 partial | 1 | 2.070 | -0.540 | 26.98 | 4 | 0 | 6 | 3 |
| *Zoothera dauma* | *Zoothera dauma* | 0 | 0 | 2.141 | -0.515 | 47.98 | 4 | 0 | 6 | 2 |

References

Gill F, Donsker D, editors. 2018. IOC world bird list (v8.1). <http://www.worldbirdnames.org/>.

Jetz W, Thomas GH, Joy JB, Hartmann K, Mooers AO. 2012. The global diversity of birds in space and time. Nature. 491:444-448.
