# Supplementary material for: Sexual selection, feather wear, and time constraints on the pre‐basic molt explain the acquisition of the pre‐alternate molt in European passerines
Source: Ecol Evol. 2022 Sep 6;12(9):e9260. doi: 10.1002/ece3.9260 (PMC9448967; doi:10.1002/ece3.9260)
Supplement: Supplementary file 5 — Table S3 [file ECE3-12-e9260-s003.docx]

Table S3. Feather tracts molted in the pre-alternate (winter-spring) molt for the 83 passerine species with this molt included in the study. Species with pre-alternate molt occurring in November or December (*n* = 16) are shown in bold. Species that do not change any flight feather (primaries, secondaries, tertials, or rectrices) in the pre-alternate molt (*n* = 37) are denoted with an asterisk (*). Species with seasonal color change in males (according to McQueen et al. 2019; *n* = 15) are denoted with a hash (#).

| Species | Pre-alternate molt | Molted feathers |
| --- | --- | --- |
| ***Acrocephalus arundinaceus*** | Complete | All |
| *Acrocephalus melanopogon** | Partial | Body feathers |
| *Acrocephalus paludicola* | Complete | All |
| *Acrocephalus palustris* | Complete | All |
| *Acrocephalus schoenobaenus* | Complete | All |
| *Acrocephalus scirpaceus* | Complete | All |
| *Anthus campestris* | Complete | All (flight feathers variable) |
| *Anthus cervinus*#* | Partial | Greater coverts, body feathers |
| *Anthus pratensis* | Partial | Inner secondaries, outer greater coverts, body feathers |
| *Anthus richardi* | Complete | All (flight feathers variable) |
| *Anthus spinoletta*#* | Partial | Body feathers |
| *Anthus trivialis* | Partial | Central rectrices, body feathers |
| ***Arundinax aedon*** | Complete | All |
| *Calcarius lapponicus** | Partial | Head feathers (chin, throat and sides of head) |
| ***Carpodacus erythrinus*** | Complete | All |
| *Cettia cetti* | Partial | Inner secondaries, greater coverts, body feathers |
| ***Delichon urbicum*** | Complete | All |
| *Emberiza caesia** | Partial | Body feathers |
| *Emberiza cirlus** | Partial | Body feathers |
| *Emberiza hortulana** | Partial | Body feathers |
| ***Emberiza melanocephala*** | Complete | All |
| *Emberiza pusilla** | Partial | Head feathers |
| *Emberiza rustica** | Partial | Primary coverts, body feathers |
| *Emberiza schoeniclus** | Partial | Body feathers |
| *Ficedula albicollis*#* | Partial | Wing coverts, body feathers |
| *Ficedula hypoleuca#* | Partial | Inner secondaries, greater coverts, body feathers |
| *Ficedula parva* | Partial | Tertials, greater coverts, body feathers |
| *Helopsaltes certhiola* | Complete | All |
| *Helopsaltes fasciolatus* | Complete | All |
| *Hippolais icterina* | Complete | All |
| ***Hippolais olivetorum*** | Complete | All |
| ***Hippolais polyglotta*** | Complete | All |
| ***Hirundo rustica*** | Complete | All |
| ***Iduna pallida*** | Complete | All |
| *Lanius collurio* | Complete | All |
| *Lanius excubitor** | Partial | Greater coverts, body feathers |
| *Lanius minor* | Complete | All |
| ***Lanius nubicus*** | Partial | Primaries, outer secondaries, rectrices (variable) |
| ***Lanius senator*** | Complete | All (flight feathers variable) |
| *Locustella fluviatilis* | Complete | All |
| *Locustella lanceolata* | Complete | All |
| ***Locustella luscinioides*** | Complete | All (all feathers variable) |
| *Locustella naevia* | Complete | All |
| *Luscinia svecica*#* | Partial | Body feathers |
| *Monticola saxatilis** | Partial | Body feathers |
| *Monticola solitarius** | Partial | Outer greater coverts, body feathers |
| *Montifringilla nivalis** | Partial | Some body feathers |
| *Motacilla alba#* | Partial | Inner secondaries, greater coverts, body feathers |
| *Motacilla cinerea*#* | Partial | Body feathers |
| *Motacilla citreola*#* | Partial | Body feathers |
| *Motacilla flava*#* | Partial | Median coverts, greater coverts, body feathers |
| *Muscicapa striata* | Complete | All |
| *Oenanthe hispanica*#* | Partial | Body feathers |
| *Oenanthe isabellina** | Partial | Body feathers |
| *Oenanthe oenanthe*#* | Partial | Body feathers |
| *Oenanthe pleschanka*#* | Partial | Body feathers |
| ***Oriolus oriolus*** | ♂ com ♀ par | ♂ all, ♀ flight feathers |
| ***Phylloscopus bonelli*** | Complete | All |
| *Phylloscopus borealis* | Complete | All |
| *Phylloscopus collybita** | Partial | Outer greater coverts, body feathers |
| *Phylloscopus inornatus** | Partial | Body feathers |
| *Phylloscopus proregulus** | Partial | Body feathers |
| *Phylloscopus sibilatrix* | Complete | All |
| *Phylloscopus trochiloides* | Complete | All |
| *Phylloscopus trochilus* | Complete | All |
| *Plectrophenax nivalis*#* | Partial | Head feathers |
| *Remiz pendulinus** | Partial | Greater coverts, body feathers |
| ***Riparia riparia*** | Complete | All |
| *Saxicola rubetra*#* | Partial | Greater coverts, body feathers |
| *Sylvia atricapilla* | Partial | Tertials, inner greater coverts, body feathers |
| ***Sylvia borin*** | Complete | All (flight feathers variable) |
| *Sylvia cantillans** | Partial | Body feathers |
| *Sylvia communis* | Partial | Tertials, body feathers |
| *Sylvia conspicillata** | Partial | Body feathers |
| *Sylvia curruca* | Partial | Tertials, rectrices, body feathers |
| *Sylvia hortensis* | Complete | All (flight feathers variable) |
| *Sylvia melanocephala** | Partial | Body feathers |
| *Sylvia nisoria* | Partial | Tertials, rectrices, body feathers |
| *Sylvia ruppeli** | Partial | Body feathers |
| *Sylvia sarda** | Partial | Body feathers |
| *Sylvia undata** | Partial | Body feathers |
| *Tichodroma muraria*#* | Partial | Head feathers (head, throat, and neck) |
| *Turdus viscivorus** | Partial | Some body feathers |

References

McQueen A, Kempenaers B, Dale J, Valcu M, Emery ZT, Dey CJ, Peters A, Delhey K. 2019. Evolutionary drivers of seasonal plumage colours: colour change by moult correlates with sexual selection, predation risk and seasonality across passerines. Ecol Lett. 22:1838-1849.
