# Supplementary material for: Sexual selection, feather wear, and time constraints on the pre‐basic molt explain the acquisition of the pre‐alternate molt in European passerines
Source: Ecol Evol. 2022 Sep 6;12(9):e9260. doi: 10.1002/ece3.9260 (PMC9448967; doi:10.1002/ece3.9260)
Supplement: Supplementary file 6 — Table S4 [file ECE3-12-e9260-s006.docx]

Table S4. MCMCglmm models with the occurrence (Models 1, 2, 3, and 4) or the extent (Model 5) of the pre-alternate molt as the dichotomous response variable and sexual selection, sexual dichromatism, migratory behavior, aerial foraging, type of habitat, and winter gregariousness/territoriality as predictor variables. Molt occurring in November or December is considered pre-alternate in some models (Models 1 and 2) but pre-basic in others (Models 3, 4, and 5).

| Model | Estimate | 95% LCI | 95% UCI | ESS | Autocorrelation | z-score | pMCMC |
| --- | --- | --- | --- | --- | --- | --- | --- |
| Model 1 |  |  |  |  |  |  |  |
| (intercept) | 0.462 | -3.955 | 4.953 | 966 | -0.004 | -0.144 | 0.842 |
| Body mass | -0.471 | -2.686 | 1.700 | 950 | -0.002 | 0.127 | 0.693 |
| **Sexual selection** | 1.113 | 0.067 | 2.185 | 961 | -0.004 | 0.037 | **0.033** |
| Sexual dichromatism | 0.004 | -0.013 | 0.021 | 966 | 0.003 | 0.076 | 0.621 |
| **Migration** | 1.248 | 0.746 | 1.778 | 972 | -0.004 | 0.155 | **0.001** |
| Aerial foraging | 0.965 | -0.132 | 2.087 | 961 | 0.002 | 0.225 | 0.091 |
| **Type of Habitat** | -0.505 | -0.952 | -0.066 | 955 | -0.006 | -0.178 | **0.026** |
| **Gregariousness (three categories)** | -1.161 | -1.980 | -0.355 | 976 | -0.003 | 0.010 | **0.006** |
| Heritability (h^2^) | 0.517 | 0.450 | 0.591 |  |  |  |  |
|  |  |  |  |  |  |  |  |
| Model 2 |  |  |  |  |  |  |  |
| (intercept) | -0.829 | -4.547 | 2.918 | 955 | 0.000 | -0.065 | 0.667 |
| Body mass | -1.021 | -2.958 | 0.878 | 961 | -0.003 | 0.130 | 0.311 |
| Sexual selection | 0.880 | -0.060 | 1.820 | 954 | 0.003 | 0.285 | 0.062 |
| Sexual dichromatism | 0.002 | -0.014 | 0.017 | 965 | -0.002 | 0.011 | 0.824 |
| **Migration** | 1.255 | 0.801 | 1.725 | 990 | -0.007 | 0.045 | **0.001** |
| **Aerial foraging** | 1.516 | 0.434 | 2.635 | 956 | -0.003 | 0.039 | **0.006** |
| **Type of habitat** | -0.476 | -0.879 | -0.065 | 960 | -0.002 | -0.094 | **0.023** |
| **Territoriality** | -1.681 | -3.377 | -0.055 | 952 | -0.002 | -0.070 | **0.042** |
| Heritability (h^2^) | 0.520 | 0.454 | 0.597 |  |  |  |  |
|  |  |  |  |  |  |  |  |
| Model 3 |  |  |  |  |  |  |  |
| (intercept) | 3.307 | -1.180 | 7.822 | 971 | -0.001 | -0.132 | 0.151 |
| Body mass | -1.641 | -3.943 | 0.586 | 957 | 0.006 | 0.028 | 0.154 |
| Sexual selection | 0.584 | -0.304 | 1.493 | 961 | 0.003 | -0.010 | 0.206 |
| Sexual dichromatism | 0.009 | -0.008 | 0.025 | 974 | -0.003 | -0.057 | 0.310 |
| **Migration** | 0.868 | 0.386 | 1.359 | 945 | -0.002 | -0.122 | **0.001** |
| Aerial foraging | 0.187 | -0.843 | 1.203 | 961 | 0.002 | 0.051 | 0.720 |
| **Type of habitat** | -0.456 | -0.887 | -0.026 | 964 | -0.001 | 0.222 | **0.038** |
| **Gregariousness (three categories)** | -1.375 | -2.162 | -0.598 | 969 | -0.006 | 0.190 | **0.001** |
| Heritability (h^2^) | 0.514 | 0.447 | 0.588 |  |  |  |  |
|  |  |  |  |  |  |  |  |
| Model 4 |  |  |  |  |  |  |  |
| (intercept) | 0.197 | -3.348 | 3.769 | 983 | -0.002 | 0.091 | 0.914 |
| Body mass | -1.787 | -3.691 | 0.084 | 977 | -0.002 | -0.121 | 0.063 |
| **Sexual selection** | 0.824 | 0.029 | 1.621 | 950 | 0.003 | -0.120 | **0.041** |
| Sexual dichromatism | 0.004 | -0.010 | 0.019 | 978 | -0.007 | 0.055 | 0.567 |
| **Migration** | 0.791 | 0.377 | 1.206 | 949 | 0.006 | -0.105 | **0.001** |
| Aerial foraging | 0.629 | -0.283 | 1.544 | 952 | 0.005 | 0.004 | 0.182 |
| **Type of habitat** | -0.426 | -0.809 | -0.055 | 958 | -0.001 | 0.049 | **0.028** |
| Territoriality | -0.536 | -1.872 | 0.798 | 950 | 0.001 | -0.013 | 0.437 |
| Heritability (h^2^) | 0.523 | 0.453 | 0.597 |  |  |  |  |
|  |  |  |  |  |  |  |  |
| Model 5 |  |  |  |  |  |  |  |
| (intercept) | -3.093 | -8.087 | 1.821 | 976 | -0.009 | -0.021 | 0.223 |
| Body mass | -2.066 | -5.242 | 1.022 | 961 | -0.002 | -0.070 | 0.197 |
| Sexual selection | -0.330 | -1.583 | 0.897 | 929 | -0.001 | -0.067 | 0.607 |
| **Sexual dichromatism** | -0.088 | -0.149 | -0.030 | 992 | -0.003 | 0.096 | **0.001** |
| **Migration** | 2.126 | 0.825 | 3.508 | 954 | -0.001 | -0.025 | **0.001** |
| Aerial foraging | 0.064 | -1.752 | 1.860 | 958 | -0.002 | 0.082 | 0.937 |
| Type of habitat | -0.116 | -0.960 | 0.733 | 979 | -0.004 | 0.074 | 0.794 |
| Gregariousness | -0.993 | -2.263 | 0.249 | 946 | -0.001 | -0.062 | 0.116 |
| Heritability (h^2^) | 0.505 | 0.439 | 0.577 |  |  |  |  |

Model 1 (*n* = 162 species): gregariousness includes three categories (non-gregarious, moderately gregarious, and gregarious) because territorial species were excluded from the analysis; molt occurring in November or December is considered pre-alternate. Model 2 (*n* = 188 species): territoriality includes two categories (territorial and non-territorial); molt occurring in November or December is considered pre-alternate. Model 3 (*n* = 162 species): gregariousness includes three categories (non-gregarious, moderately gregarious, and gregarious) because territorial species were excluded from the analysis; molt occurring in November or December is considered pre-basic. Model 4 (*n* = 188 species): territoriality includes two categories (territorial and non-territorial); molt occurring in November or December is considered pre-basic. Model 5 (*n* = 67 species): only species with pre-alternate molt are included (molt occurring in November or December is considered pre-basic); gregariousness includes four categories (territorial, non-gregarious, moderately gregarious, and gregarious). Log_10_-transformed body mass was included in all models as a confounding factor. Models were run on 100 random phylogenetic trees and, for each independent variable, we show the average of the following parameters: estimate, lower (LCI) and upper (UCI) 95% credibility interval of the estimate, effective sample size (ESS), level of autocorrelation, z-score of the Geweke’s convergence diagnostic, and pMCMC value. Heritability (h^2^) represents the phylogenetic signal and we show the average of the estimate and the average of 95% LCI and UCI. pMCMC < 0.05 (shown in bold) denotes statistical significance.
